# Supplementary material for: Global and regional burden and inequalities of oral conditions in children, adolescents, and young adults (0–39 years), 1990 to 2021
Source: PLOS Glob Public Health. 2025 Oct 9;5(10):e0005274. doi: 10.1371/journal.pgph.0005274 (PMC12510561; doi:10.1371/journal.pgph.0005274)
Supplement: S1 Text — (DOCX) [file pgph.0005274.s001.docx]

# S1 Text: Supplementary Methods

Portions of the Supplementary Methods are reproduced, with permission, from the appendices of other GBD 2021 publications for analytic components that are common across them. Input data sources for all components of GBD estimations are viewable at the Global Health Data Exchange (http://ghdx.healthdata.org). GBD 2021 results are viewable online in an interactive visualization tool called GBD Compare (<http://vizhub.healthdata.org/gbd-compare>).

## The GBD 2021 methods for estimation of the burden of oral disorders

In this section, we outline the key steps in estimating oral disorders in GBD 2021 [1]. For a comprehensive explanation, please refer to the official GBD page for the complete estimation methodology: https://www.healthdata.org/sites/default/files/methods_appendices/2021/Oral_nonfatal_2020_final_jw_updated_atm_Apr_17_AC.pdf.

Data searching and systematic literature reviews were completed for all oral disorders due to the overlap in data types and sources used for modelling. The Expert Group for GBD 2010 conducted an initial literature review in PubMed, Embase, LILACS, and SciELO, encompassing published articles and national reports. For GBD 2021, a review of the Global Health Data Exchange (https://ghdx.healthdata.org) was undertaken to compile oral health surveys and national epidemiological reports. A total of 105 new sources were identified and integrated into the GBD 2021 models after eliminating redundant data points. Emphasis was placed on preserving data granularity while minimizing repetition, particularly in age, gender, and urbanicity descriptors, with priority given to age data, followed by gender and urbanicity. Many studies presented decayed, missing, and filled teeth (dmft) or DMFT scores, which denote the lifetime prevalence of dental caries. When assessing the disability burden from dental caries, only current prevalence data was deemed relevant, with lifetime prevalence data converted to current prevalence and incidence whenever feasible.

The modelling approach for untreated caries of deciduous and permanent teeth involved treating deciduous and permanent caries separately and then combining the estimates to determine the global burden of dental caries, consistently implemented since GBD 2010. The case definition for dental caries includes teeth with visible cavities at the dentin level, soft to probing, restorations, or missing teeth due to caries. Exclusions are cosmetic defects, stained enamel pits, fluorosis, and abrasion lesions. The dmft/DMFT index is commonly utilized by public health dentists, with lowercase letters for deciduous dentition and uppercase for permanent dentition. Lifetime prevalence data were converted to current prevalence using the d/D to dmf/DMF ratio for individuals aged 20 and under. Incremental dmf/DMF scores were converted to incidence values for permanent caries in individuals aged 20 and under and all ages for deciduous caries. Caries incidence in longitudinal studies was determined based on the difference in dmf/DMF scores between initial and subsequent examinations, while cross-sectional studies calculated incidence for age intervals ≤3 years. To address significant heterogeneity in caries incidence and prevalence data, age and sex were separated, and meta-regression—Bayesian, regularized, trimmed (MR-BRT) was applied. Within-study matches compared both methods of ascertainment in the same study population. The ratio of alternative to reference was logit-transformed, and the standard error was calculated using the delta method. Sex was included as a fixed effect, and for prevalence, the midpoint of age was treated as a spline. Adjustments were made for deciduous caries data derived from DMFS due to limited availability, reflecting an age-specific relationship between these measures. Similar adjustments were made for data on permanent teeth caries using the same crosswalks. Prevalence data for permanent teeth caries showed age-dependent variations between data from surface measurements and tooth measurements, necessitating adjustments of DMFS-derived prevalence data to align with tooth references. Conversion of D/DMF was focused on children under 13 years old, providing accurate measures on the relationship between D/DMFS and D/DMFT. Insufficient data hindered an assessment of differences between cohort-based caries incidence and incidence from DMF increment. The DisMod model development used similar settings for both caries types, with the primary difference in value priors. Zero incident caries were assumed for infants under 1-year-old and zero incident deciduous caries from age 11 onward. For permanent caries, zero-incident cases were assumed for children under 5. Location-level covariates were assigned separately for prevalence and incidence, with sugar availability affecting incidence and log-transformed LDI affecting prevalence to reflect dental care access. GBD estimates of edentulism prevalence were employed to adjust Years lost due to disability (YLD) estimates for permanent teeth caries. Since oral disorders hardly lead to death, the GBD 2021 study assumed years of life lost (YLLs) for them = 0. Therefore, the estimates of disability-adjusted life years, which consist of the sum of YLLs and YLDs, were equal to YLDs. After estimating standard severity distributions and disability weights, asymptomatic individuals were considered to experience no disability. Those with mild disease had one hour of pain per day throughout the disease duration, while those with severe disease had constant pain in the terminal phase after an initial phase. YLDs were calculated by multiplying the prevalence, disability duration, proportion with symptoms, and disability weight for each age, country, sex, and year.

The modelling approach for periodontal disease followed the GBD definition of disability as bad breath, a bad taste in the mouth, and occasional gum bleeding without disrupting daily activities. Attachment loss (AL) and pocket depth (PD) served as the primary metrics for assessing periodontal health. Using the Community Periodontal Index (CPI) method, only individuals with Class 2 pockets (6 mm or more) were considered. Loss of attachment was evaluated by sextants, with the highest score recorded in the absence of an index tooth. Studies reporting the number of sextants rather than individuals were excluded. Severe periodontal disease definitions included CPITN Class 4, CPI Class 3, Clinical AL >6 mm, Clinical AL >5 mm, Clinical AL >4 mm, and Gingival PD >5 mm, with preference given to CPITN = 4, followed by AL >6 mm and PD >5 mm. No additional crosswalking was conducted for these definitions. Crosswalks in MR-BRT were performed for alternative definitions after Age-sex splitting, using within-study matches. In the DisMod-MR 2.1 model, mortality and relative risk were held constant until age 30, with incidence and prevalence set to zero until age 8. Incidence began at age 9, based on the youngest age with a non-zero prevalence estimate. GBD estimates of edentulism prevalence were used to adjust YLD estimates for periodontal disease. All prevalent periodontal disease cases were assumed to have the described disability, with a uniform disability weight of 0.007 (0.003–0.014) regardless of pain experience.

The modelling approach for edentulism began with the case definition that includes individuals with no remaining permanent teeth, excluding toothlessness in infancy. Self-report data on toothlessness from the World Health Survey (WHS) for 47 countries and various national oral health surveys were utilised alongside published studies. Following age-sex division, self-reported toothlessness data were crosswalked to the standard oral examination in MR-BRT. However, no statistically significant difference was detected between self-reporting and oral examination, thus no adjustments were applied. Prevalence estimates for edentulism were calculated for each location, year, sex, and age using DisMod-MR 2.1. Remission was fixed at zero for all ages due to the irreversible nature of edentulism. Mortality and relative risk were held steady at zero before age 30. Incidence and prevalence were nullified during childhood, with incidence authorised to start at age 15 when permanent dentition is fully developed. A supplementary review for GBD 2010 uncovered six methodical studies on denture prevalence, all conducted in high- and middle-income countries. Linear regressions of denture presence and absence against health system access (HSA) were utilised to compute the prevalence of no dentures across all super-regions. A population-weighted average based on 2003 populations was computed for each super-region. Uncertainties were evaluated by determining the standard deviation and standard error of the prevalence values. The calculated prevalence of dentures in each location determined the proportion of individuals with asymptomatic edentulism and severe tooth loss (those with denture access) and those encountering eating difficulties due to edentulism and severe tooth loss (those lacking denture access), with the latter condition being recognized as a cause of YLDs.

The modelling approach for other oral disorders covers a range of dental, tongue, and jaw disorders and malformations, excluding those in the case definitions of permanent or deciduous dental caries, periodontal disease, or edentulism and severe tooth loss. Data on the prevalence of other oral disorders were sourced from the United States Medical Expenditure Panel Survey (MEPS), a nationally representative survey conducted annually from 1996 to 2011 by the US Agency for Healthcare Research and Quality. These data were analyzed in DisMod-MR 2.1 using a prevalence-only model, with disability weights and severity distribution for these conditions also derived from MEPS. The incidence data of other oral disorders was not available.

## Sociodemographic index

The sociodemographic index (SDI) is a composite indicator strongly correlated with the sociodemographic development status and health outcomes of a location, calculated as the geometric mean of three indices: total fertility rate among those under 25, mean education level for individuals aged 15 and older, and lag-distributed income per capita. The SDI ranges from 0 (lowest) to 1 (highest) [2]. It is calculated as the geometric mean of three indices: total fertility rate among those under 25, mean education level for individuals aged 15 and older, and lag-distributed income per capita. The SDI ranges from 0 to 1, which means a location with an SDI of 0 would have a theoretical minimum level of development relevant to health, while a location with an SDI of 1 would have a theoretical maximum level[3]. In this study, GBD SDI values for 1990 and 2021 were used. SDI quintiles of countries and territories were determined based on GBD SDI values of countries and territories with a population size greater than one million. The world's 204 countries and territories are categorized into five SDI quintiles, in descending order of SDI value: low-SDI quintiles, low-middle-SDI quintiles, middle-SDI quintiles, high-middle-SDI quintiles, and high-SDI quintiles. The corresponding range of SDI values for each SDI quintile is shown in GBD 2021 SDI reference quintile values (Supplementary Table 2).

## Decomposition analysis

The purpose of decomposition analysis is to quantitatively analyze the drivers of change in a health indicator. For the burden of disease, the result obtained from a decomposition analysis is the effect of a change in a driver on the change in total when the year is changed, and other drivers are held constant[3, 4]. Moreover, the sum of the effects of the drivers should be exactly equal to the total change in health indicators. In this study, we used the decomposition methodology of Das Gupta[5] to decompose the burden of oral disorders (incidence, prevalence, and YLDs) by shifts in population age structure, population growth, and epidemiological changes (referred to this study as age-standardized rates). The number of burdens at each SDI quintile was obtained from the following formula, using YLDs as an example:

$${YLD}_{ay, py, ey}=\sum_{i=1}^{8} (a_{i,y}*p_{y}*e_{i,y})$$

${YLD}_{ay,py,ey}$ respectively represented YLD based on shifts in population age structure, population growth, and epidemiological change for a specific year *y*, $a_{i,y}$ represents the proportion of population for the age category *i* of the 8 age categories for children, adolescents, and young adults (0-4y, 5-9y, 10-14y, 15-19y, 20-24y, 25-29y, 30-34y, and 35-39y) in the specific year *y*; $p_{y}$ represents the total population in given year *y*; and $e_{i,y}$ represents the epidemiological change (age-standardized YLD rate) of the given age category *i* in the specific year *y*. The contribution of each driver to the change in the number of YLDs from 1990 to 2021 was defined by the effect of one driver changing while the other drivers were held constant. For example, the effect of shifts in population age structure was calculated as:

$$\left( \frac{{YLD}_{a2021, p1990, e1990}+ {YLD}_{a2021, p2021, e2021}}{3}+ \frac{{YLD}_{a2021, p1990, e2021}+ {YLD}_{a2021, p2021, e1990}}{6} \right)-\left( \frac{{YLD}_{a1990, p2021, e2021}+{YLD}_{a1990, p1990, e1990}}{3}+ \frac{{YLD}_{a1990, p2021, e1990}+{YLD}_{a1990, p1990, e2021}}{6} \right)$$

The effects of drivers do not necessarily imply any causal relationships. They simply indicate the nature of the association of the drivers with the phenomenon being measured. There might be some hidden forces behind the drivers that are responsible for the numbers we allocate to different drivers as effects, but identifying those forces is beyond the scope of the decomposition analysis.

## Measurement of health inequality

### Slope index of inequality

The Slope Index of Inequality (SII) is utilised to demonstrate the gradient of health outcomes across various groups with a natural order, commonly based on education or wealth status[6, 7]. In this study, the SDI quintiles were employed for this purpose. The SII quantifies the absolute difference in health indicator values (incidence, prevalence, or YLDs) between countries and territories within the high-SDI and low-SDI quintiles, accounting for the entire SDI distribution through an appropriate regression model. The choice of the model in this research was guided by the outcomes of the Breusch-Pagan test, with an iterative weighted linear model selected when the test's p-value was below 0.05; otherwise, a linear regression model was utilised.

To compute the SII, a weighted sample representing the entire population is ordered from the low-SDI quintile to the high-SDI quintile. This ordering is weighted, taking into consideration the population proportion of countries and territories within each SDI quintile. The population of each country or territory is then analysed in terms of its position in the cumulative population distribution and its midpoint. Subsequently, a suitable model is applied to regress the relevant health indicator against the midpoint value for SDI quintiles, predicting the health indicator values at the extremes (SDI = 1 and 0). The disparity between these predicted values across the entire distribution yields the SII. Consequently, the SII illustrates the difference between the low-SDI and high-SDI quintiles, encompassing all other SDI quintiles in the regression analysis. In the ranking from the low-SDI quintile to the high-SDI quintile, positive SII values signify a higher prevalence of the health indicator in the high-SDI quintile, whereas negative values indicate a higher prevalence in the low-SDI quintile.

### Concentration index of inequality

The concentration index (CII) is a relative measure of inequality that shows the health gradient across multiple groups with a natural order, commonly based on education or wealth status[6, 7]. In this study, the SDI quintiles were employed for this purpose. The CII indicates the extent to which a health indicator of interest is concentrated among the lower SDI quintiles or the higher SDI quintiles. As the entire population is arranged in ascending order of SDI values, the CII assumes a negative value when the health indicator is concentrated among the lower SDI quintiles (poorer countries and territories) and a positive value when the indicator is prevalent in the higher SDI quintiles (richer countries and territories). In situations of complete equality, the CII equates to 0. In extreme scenarios where a country or territory monopolises 100% of a health indicator across all countries and territories (the maximal relative inequality conceivable), the CII tends towards its absolute extremities of either -1 or +1. While 1 is the theoretical maximum of the absolute value of a CII, in practice absolute values for the CII will rarely exceed 0.5, and a range between 0.2 and 0.3 is deemed to exemplify a notably high level of relative inequality.

To illustrate the CII, a concentration curve is employed. Like the SII, it starts with ranking a weighted sample of the whole population of all countries and territories from the low-SDI quintile to the high-SDI quintile. The *y* axis indicates the cumulative fraction of the health indicator (incidence, prevalence, or YLDs) corresponding to each SDI quintile. The concentration curve is drawn by connecting the dots. A 45° diagonal line from the bottom left corner to the top right is called the line of equality. Specifically, the concentration curve lies below the line of equality if the health indicator is concentrated among the higher SDI quintiles (richer countries and territories); the concentration curve lies above the line of equality if the health indicator is concentrated among the lower SDI quintiles (poorer countries and territories). In scenarios devoid of inequality, the concentration curve lies on the line of equality. The CII is calculated as twice the area enclosed between the line of equality and the concentration curve.

### Sociodemographic attribution analysis

Sociodemographic attribution analysis is to determine the burden attributable to inequality in sociodemographic development status. It was conducted to determine the number and proportion of incident cases of oral disorders in 2021 attributable to country-level sociodemographic inequality, under a scenario that equals disease burdens across all countries and territories akin to those with high-SDI quintiles[3]. The Monte Carlo method was employed to estimate a 95% CI, generating 10,000 simulations.

**References**

1. Ward, Z.J. and S.J. Goldie, *Global Burden of Disease Study 2021 estimates: implications for health policy and research.* Lancet, 2024. **403**(10440): p. 1958-1959.

2. GBD 2021 Diabetes Collaborators, *Global, regional, and national burden of diabetes from 1990 to 2021, with projections of prevalence to 2050: a systematic analysis for the Global Burden of Disease Study 2021.* Lancet, 2023. **402**(10397): p. 203-234.

3. Wen, P.Y.F., M.X. Chen, Y.J. Zhong, Q.Q. Dong, and H.M. Wong, *Global Burden and Inequality of Dental Caries, 1990 to 2019.* J. Dent. Res., 2022. **101**(4): p. 392-399.

4. Xie, Y., B. Bowe, A.H. Mokdad, H. Xian, Y. Yan, T. Li, et al., *Analysis of the Global Burden of Disease study highlights the global, regional, and national trends of chronic kidney disease epidemiology from 1990 to 2016.* Kidney Int., 2018. **94**(3): p. 567-581.

5. Das Gupta, P., *Standardization and decomposition of rates : a user's manual*. 1993, U.S. Dept. of Commerce, Economics and Statistics Administration, Bureau of the Census: Washington DC.

6. WHO, *Handbook on health inequality monitoring: with a special focus on low-and middle-income countries*. 2013, World Health Organization.

7. Ordunez, P., R. Martinez, P. Soliz, G. Giraldo, O.J. Mujica, and P. Nordet, *Rheumatic heart disease burden, trends, and inequalities in the Americas, 1990-2017: a population-based study.* Lancet Glob. Health, 2019. **7**(10): p. e1388-e1397.
