# Supplementary material for: Global and regional burden and inequalities of oral conditions in children, adolescents, and young adults (0–39 years), 1990 to 2021
Source: PLOS Glob Public Health. 2025 Oct 9;5(10):e0005274. doi: 10.1371/journal.pgph.0005274 (PMC12510561; doi:10.1371/journal.pgph.0005274)
Supplement: S2 Text — (DOCX) [file pgph.0005274.s002.docx]

# S2 Text: GATHER Checklist

This study complies with the Guidelines for Accurate and Transparent Health Estimates Reporting (GATHER) recommendations. It includes detailed tables and information on data to maximize transparency in our estimation processes and provide a comprehensive description of analytical steps.

| **Objectives and Funding** | Reported on |
| --- | --- |
| 1. Define the indicator(s), populations (including age, sex, and geographic entities), and time period(s) for which estimates were made. | Methods (Data source) and Supplementary Methods |
| 2. List the funding sources for the work. | Summary (Funding) |
| **Data Inputs** |  |
| **For all data inputs from multiple sources that are synthesised as part of the study:** |  |
| 3. Describe how the data were identified and how the data were accessed. | Main text (Methods) and Supplementary Methods |
| 4. Specify the inclusion and exclusion criteria. Identify all ad-hoc exclusions | Main text (Methods) and Supplementary Methods |
| 5. Provide information about all included data sources and their main characteristics. For each data source used, report reference information or contact name/institution, population represented, data collection method, year(s) of data collection, sex and age range, diagnostic criteria or measurement method, and sample size, as relevant. | Main text (Methods) and http://ghdx.healthdata.org |
| 6. Identify and describe any categories of input data that have potentially important biases (e.g., based on characteristics listed in item 5). | Main text (Methods) and Supplementary Methods |
| **For data inputs that contribute to the analysis but were not synthesised as part of the study:** |  |
| 7. Describe and give sources for any other data inputs. | Main text (Methods) and online data source tool http://ghdx.healthdata.org |
| **For all data inputs:** |  |
| 8. Provide all data inputs in a file format from which data can be efficiently extracted (e.g., a spreadsheet rather than a PDF), including all relevant meta-data listed in item 5. For any data inputs that cannot be shared because of ethical or legal reasons, such as third-party ownership, provide a contact name or the name of the institution that retains the right to the data. | http://ghdx.healthdata.org |
| **Data Analysis** |  |
| 9. Provide a conceptual overview of the data analysis method. A diagram may be helpful. | Main text (Methods) and Figure 1 |
| 10. Provide a detailed description of all steps of the analysis, including mathematical formulae. This description should cover, as relevant, data cleaning, data pre-processing, data adjustments and weighting of data sources, and mathematical or statistical model(s). | Main text (Methods) and Supplementary Methods |
| 11. Describe how candidate models were evaluated and how the final model(s) were selected. | Supplementary Methods |
| 12. Provide the results of an evaluation of model performance, if done, as well as the results of any relevant sensitivity analysis. | Main text (Methods) and Supplementary Methods |
| 13. Describe methods of calculating uncertainty of the estimates. State which sources of uncertainty were, and were not, accounted for in the uncertainty analysis. | Supplementary Methods |
| 14. State how analytic or statistical source code used to generate estimates can be accessed. | For GBD estimates, see [Global Burden of Disease Study 2021 (GBD 2021) Data Resources \| GHDx (healthdata.org)](https://ghdx.healthdata.org/gbd-2021); for Joinpoint Regression Analysis program, see <https://surveillance.cancer.gov/joinpoint/> |
| **Results and Discussion** |  |
| 15. Provide published estimates in a file format from which data can be efficiently extracted. | http://ghdx.healthdata.org |
| 16. Report a quantitative measure of the uncertainty of the estimates (e.g., uncertainty intervals). | Main text (Results) and Supplementary Results |
| 17. Interpret results in light of existing evidence. If updating a previous set of estimates, describe the reasons for changes in estimates. | Main text (Discussion) and Supplementary Discussion |
| 18. Discuss limitations of the estimates. Include a discussion of any modelling assumptions or data limitations that affect interpretation of the estimates. | Main text (Discussion) |
