# Supplementary material for: Global and regional burden and inequalities of oral conditions in children, adolescents, and young adults (0–39 years), 1990 to 2021
Source: PLOS Glob Public Health. 2025 Oct 9;5(10):e0005274. doi: 10.1371/journal.pgph.0005274 (PMC12510561; doi:10.1371/journal.pgph.0005274)
Supplement: S3 Text — (DOCX) [file pgph.0005274.s003.docx]

# S3 Text: Supplementary Figures and Tables

# Supplementary Figures

**List of Figure Legends**

**Fig A. Age distribution and the SDl quintiles distribution of global standard population in 1990 and 2021**

**(A)** Distribution of the global standard population per 100,000 population in 1990 and 2021, by SDI quintiles.

**(B)** Distribution of the global standard population per 100,000 population in 1990 and 2021, by age groups. SDI, sociodemographic index.

**Fig B. The distribution of age-standardized incidence rates per 100,000 populations of oral disorders for 204 countries and territories in 2021.**

1. The distribution of age-standardized incidence rates per 100,000 populations in young population (0-39 years) for oral disorders across 204 countries and territories in 2021.
2. The distribution of age-standardized incidence rates per 100,000 populations in children (0-9 years) for oral disorders across 204 countries and territories in 2021.
3. The distribution of age-standardized incidence rates per 100,000 populations in adolescents (10-19 years) for oral disorders across 204 countries and territories in 2021.
4. The distribution of age-standardized incidence rates per 100,000 populations in young adults (20-39 years) for oral disorders across 204 countries and territories in 2021.

ASIR, age-standardized incidence rate. The link to the base layer of the map: https://www.resdc.cn/data.aspx?DATAID=205.

**Fig C. The distribution of age-standardized prevalence rates per 100,000 populations of oral disorders for 204 countries and territories in 2021.**

1. The distribution of age-standardized prevalence rates per 100,000 populations in young population (0-39 years) for oral disorders across 204 countries and territories in 2021.
2. The distribution of age-standardized prevalence rates per 100,000 populations in children (0-9 years) for oral disorders across 204 countries and territories in 2021.
3. The distribution of age-standardized prevalence rates per 100,000 populations in adolescents (10-19 years) for oral disorders across 204 countries and territories in 2021.
4. The distribution of age-standardized prevalence rates per 100,000 populations in young adults (20-39 years) for oral disorders across 204 countries and territories in 2021.

ASPR, age-standardized prevalence rate. The link to the base layer of the map: https://www.resdc.cn/data.aspx?DATAID=205.

**Fig D. The distribution of age-standardized YLD rates per 100,000 populations of oral disorders for 204 countries and territories in 2021 (with unified the scale across all plots).**

1. The distribution of age-standardized YLD rates per 100,000 populations in young population (0-39 years) for oral disorders across 204 countries and territories in 2021.
2. The distribution of age-standardized YLD rates per 100,000 populations in children (0-9 years) for oral disorders across 204 countries and territories in 2021.
3. The distribution of age-standardized YLD rates per 100,000 populations in adolescents (10-19 years) for oral disorders across 204 countries and territories in 2021.
4. The distribution of age-standardized YLD rates per 100,000 populations in young adults (20-39 years) for oral disorders across 204 countries and territories in 2021.

ASYLDR, age-standardized years lived with disability rate. The link to the base layer of the map: https://www.resdc.cn/data.aspx?DATAID=205.

**Fig E. The age-standardized rates per 100,000 populations and number of the incidence of oral disorders across different sexes in the period 1990-2021**

The age-standardized incidence rate per 100,000 populations and number of incident cases for all sexes in the period 1990-2021, of untreated caries deciduous teeth, untreated caries of permanent teeth, periodontal diseases, and edentulism. ASIR, age-standardized incidence rate.

**Fig F. The age-standardized rates per 100,000 populations and number of the prevalence of oral disorders across different sexes in the period 1990-2021**

The age-standardized prevalence rate per 100,000 populations and number of prevalent cases for all sexes in the period 1990-2021, of untreated caries deciduous teeth, untreated caries of permanent teeth, periodontal diseases, edentulism, and other oral disorders. ASPR, age-standardized prevalence rate.

**Fig G. The age-standardized rates per 100,000 populations and number of the YLDs of oral disorders across different sexes in the period 1990-2021**

The age-standardized YLD rate per 100,000 populations and number of YLDs for all sexes in the period 1990-2021, of untreated caries deciduous teeth, untreated caries of permanent teeth, periodontal diseases, edentulism, and other oral disorders. ASYLDR, age-standardized years lived with disability rate.

**Fig H. The temporal trends in age-standardized incidence rates per 100,000 populations of oral disorders from 1990 to 2021, and joinpoints, annual percentage changes, and average annual percentage changes in the period 1990-2021.**

1. The temporal trends in age-standardized incidence rates per 100,000 populations in young population (0-39 years) for oral disorders from 1990 to 2021.
2. The temporal trends in age-standardized incidence rates per 100,000 populations in children (0-9 years) for oral disorders from 1990 to 2021.
3. The temporal trends in age-standardized incidence rates per 100,000 populations in adolescents (10-19 years) for oral disorders from 1990 to 2021.
4. The temporal trends in age-standardized incidence rates per 100,000 populations in young adults (20-39 years) for oral disorders from 1990 to 2021.

ASIR, age-standardized incidence rate; APC, annual percentage changes; AAPC, average annual percentage changes.

**Fig I. The temporal trends in age-standardized prevalence rates per 100,000 populations of oral disorders from 1990 to 2021, and joinpoints, annual percentage changes, and average annual percentage changes in the period 1990-2021.**

1. The temporal trends in age-standardized prevalence rates per 100,000 populations in young population (0-39 years) for oral disorders from 1990 to 2021.
2. The temporal trends in age-standardized prevalence rates per 100,000 populations in children (0-9 years) for oral disorders from 1990 to 2021.
3. The temporal trends in age-standardized prevalence rates per 100,000 populations in adolescents (10-19 years) for oral disorders from 1990 to 2021.
4. The temporal trends in age-standardized prevalence rates per 100,000 populations in young adults (20-39 years) for oral disorders from 1990 to 2021.

ASPR, age-standardized prevalence rate; APC, annual percentage changes; AAPC, average annual percentage changes.

**Fig J. Changes in the number of incidence cases of oral disorders attributed to different drivers from 1990 to 2021 at the global level and by SDI quintiles.**

1. Changes in the number of incidence cases for oral disorders among young population (0-39 years) attributed to different drivers from 1990 to 2021 at the global level and by SDI quintiles.
2. Changes in the number of incidence cases for oral disorders among children (0-9 years) attributed to different drivers from 1990 to 2021 at the global level and by SDI quintiles.
3. Changes in the number of incidence cases for oral disorders among adolescents (10-19 years) attributed to different drivers from 1990 to 2021 at the global level and by SDI quintiles.
4. Changes in the number of incidence cases for oral disorders among young adults (20-39 years) attributed to different drivers from 1990 to 2021 at the global level and by SDI quintiles.

SDI, sociodemographic index.

**Fig K. Changes in the number of prevalence cases of oral disorders attributed to different drivers from 1990 to 2021 at the global level and by SDI quintiles.**

1. Changes in the number of prevalence cases for oral disorders among young population (0-39 years) attributed to different drivers from 1990 to 2021 at the global level and by SDI quintiles.
2. Changes in the number of prevalence cases for oral disorders among children (0-9 years) attributed to different drivers from 1990 to 2021 at the global level and by SDI quintiles.
3. Changes in the number of prevalence cases for oral disorders among adolescents (10-19 years) attributed to different drivers from 1990 to 2021 at the global level and by SDI quintiles.
4. Changes in the number of prevalence cases for oral disorders among young adults (20-39 years) attributed to different drivers from 1990 to 2021 at the global level and by SDI quintiles.

SDI, sociodemographic index.

**Fig L. SDI-related health inequality regression lines indicating absolute inequality in the ASIR of oral disorders, 1990 and 2021.**

1. SDI-related health inequality regression lines indicating absolute inequality in the ASIR of oral disorders among young population (0-39 years).
2. SDI-related health inequality regression lines indicating absolute inequality in the ASIR of oral disorders among children (0-9 years).
3. SDI-related health inequality regression lines indicating absolute inequality in the ASIR of oral disorders among adolescents (10-19 years).
4. SDI-related health inequality regression lines indicating absolute inequality in the ASIR of oral disorders among young adults (20-39 years).

Dots represent countries and territories; dot size represents population. CI, confidence interval; SDI, sociodemographic index; ASIR, age-standardized incidence rate.

**Fig M. SDI-related health inequality regression lines indicating absolute inequality in the ASPR of oral disorders, 1990 and 2021.**

1. SDI-related health inequality regression lines indicating absolute inequality in the ASPR of oral disorders among young population (0-39 years).
2. SDI-related health inequality regression lines indicating absolute inequality in the ASPR of oral disorders among children (0-9 years).
3. SDI-related health inequality regression lines indicating absolute inequality in the ASPR of oral disorders among adolescents (10-19 years).
4. SDI-related health inequality regression lines indicating absolute inequality in the ASPR of oral disorders among young adults (20-39 years).

Dots represent countries and territories; dot size represents population. CI, confidence interval; SDI, sociodemographic index; ASPR, age-standardized prevalence rate.

**Fig N. SDI-related health inequality concentration curves indicating relative inequality in the ASIR of oral disorders, 1990 and 2021.**

1. SDI-related health inequality concentration curves indicating relative inequality in the ASIR of oral disorders among young population (0-39 years).
2. SDI-related health inequality concentration curves indicating relative inequality in the ASIR of oral disorders among children (0-9 years).
3. SDI-related health inequality concentration curves indicating relative inequality in the ASIR of oral disorders among adolescents (10-19 years).
4. SDI-related health inequality concentration curves indicating relative inequality in the ASIR of oral disorders among young adults (20-39 years).

Dots represent countries and territories; dot size represents population. CI, confidence interval; SDI, sociodemographic index; ASIR, age-standardized incidence rate.

**FigO. SDI-related health inequality concentration curves indicating relative inequality in the ASPR of oral disorders, 1990 and 2021.**

1. SDI-related health inequality concentration curves indicating relative inequality in the ASPR of oral disorders among young population (0-39 years).
2. SDI-related health inequality concentration curves indicating relative inequality in the ASPR of oral disorders among children (0-9 years).
3. SDI-related health inequality concentration curves indicating relative inequality in the ASPR of oral disorders among adolescents (10-19 years).
4. SDI-related health inequality concentration curves indicating relative inequality in the ASPR of oral disorders among young adults (20-39 years).

Dots represent countries and territories; dot size represents population. CI, confidence interval; SDI, sociodemographic index; ASPR, age-standardized prevalence rate.

**Fig P. The global distribution and temporal trends of ASPRs and ASYLDRs per 100,000 populations of other oral disorders in 2021.**

1. The distribution of age-standardized prevalence rates per 100,000 populations in young population (0-39 years) for other oral disorders across 204 countries and territories in 2021.
2. The distribution of age-standardized YLD rates per 100,000 populations in young population (0-39 years) for other oral disorders across 204 countries and territories in 2021.
3. The temporal trends in age-standardized prevalence rates per 100,000 populations in young population (0-39 years) for oral disorders from 1990 to 2021, and joinpoints, annual percentage changes, and average annual percentage changes in the period 1990-2021.
4. The temporal trends in age-standardized YLD rates per 100,000 populations in young population (0-39 years) for oral disorders from 1990 to 2021, and joinpoints, annual percentage changes, and average annual percentage changes in the period 1990-2021.

ASPR, age-standardized prevalence rate; ASYLDR, age-standardized years lived with disability rate; APC, annual percentage changes; AAPC, average annual percentage changes.

**Fig Q. Changes in the number of prevalence cases and YLDs of other oral disorders attributed to different drivers from 1990 to 2021 by SDI quintiles, as well as the SDI-related absolute inequality and relative inequality in the prevalence and YLDs of other oral disorders, 1990 and 2021.**

1. Changes in the number of prevalence cases and YLDs for other oral disorders among young population (0-39 years) attributed to different drivers from 1990 to 2021 at the global level and by SDI quintiles.
2. SDI-related health inequality regression lines indicating absolute inequality in the ASPR of other oral disorders among young population (0-39 years).
3. SDI-related health inequality regression lines indicating absolute inequality in the ASYLDR of other oral disorders among young population (0-39 years).
4. SDI-related health inequality concentration curves indicating relative inequality in the ASPR of other oral disorders among young population (0-39 years).
5. SDI-related health inequality concentration curves indicating relative inequality in the ASYLDR of other oral disorders among young population (0-39 years).

Dots represent countries and territories; dot size represents population. CI, confidence interval; SDI, sociodemographic index; ASPR, age-standardized prevalence rate; ASYLDR, age-standardized years lived with disability rate.

## Fig A. Age distribution and the SDl quintiles distribution of global standard population in 1990 and 2021

**
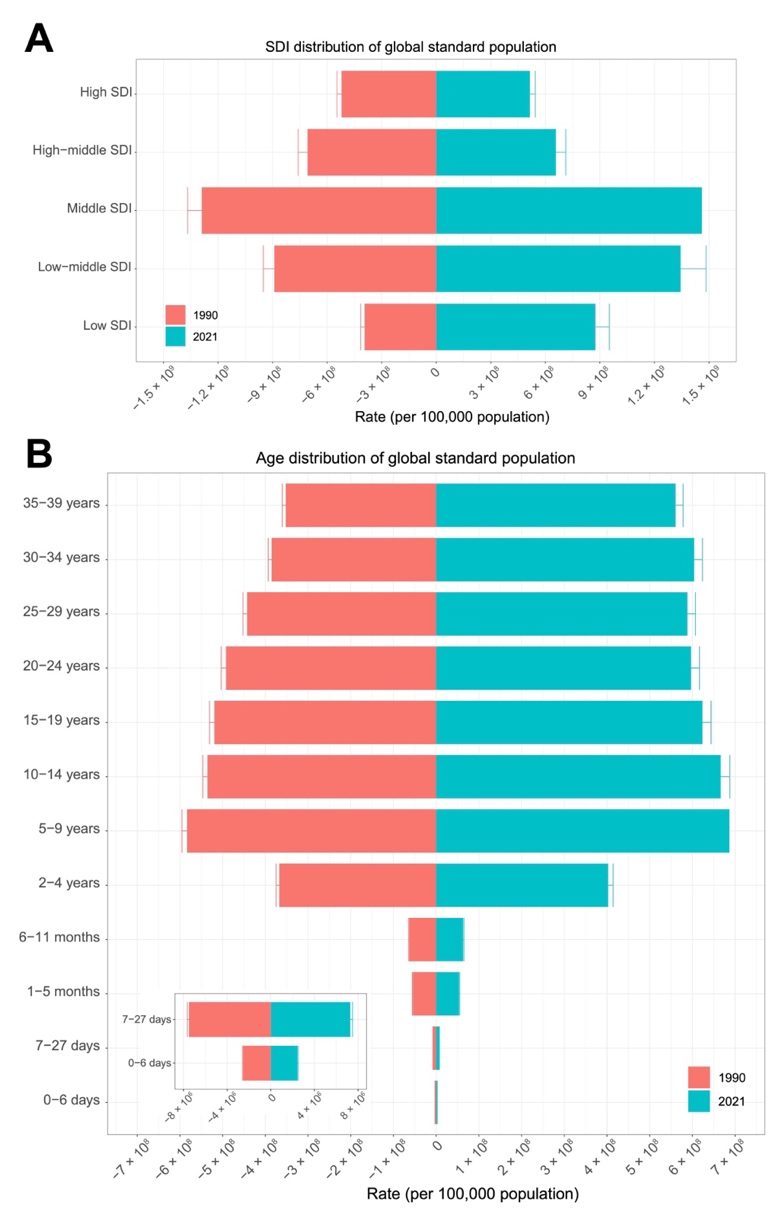
**

**(A)** Distribution of the global standard population per 100,000 population in 1990 and 2021, by SDI quintiles.

**(B)** Distribution of the global standard population per 100,000 population in 1990 and 2021, by age groups. SDI, sociodemographic index.

## Fig B. The distribution of age-standardized incidence rates per 100,000 populations of oral disorders for 204 countries and territories in 2021.


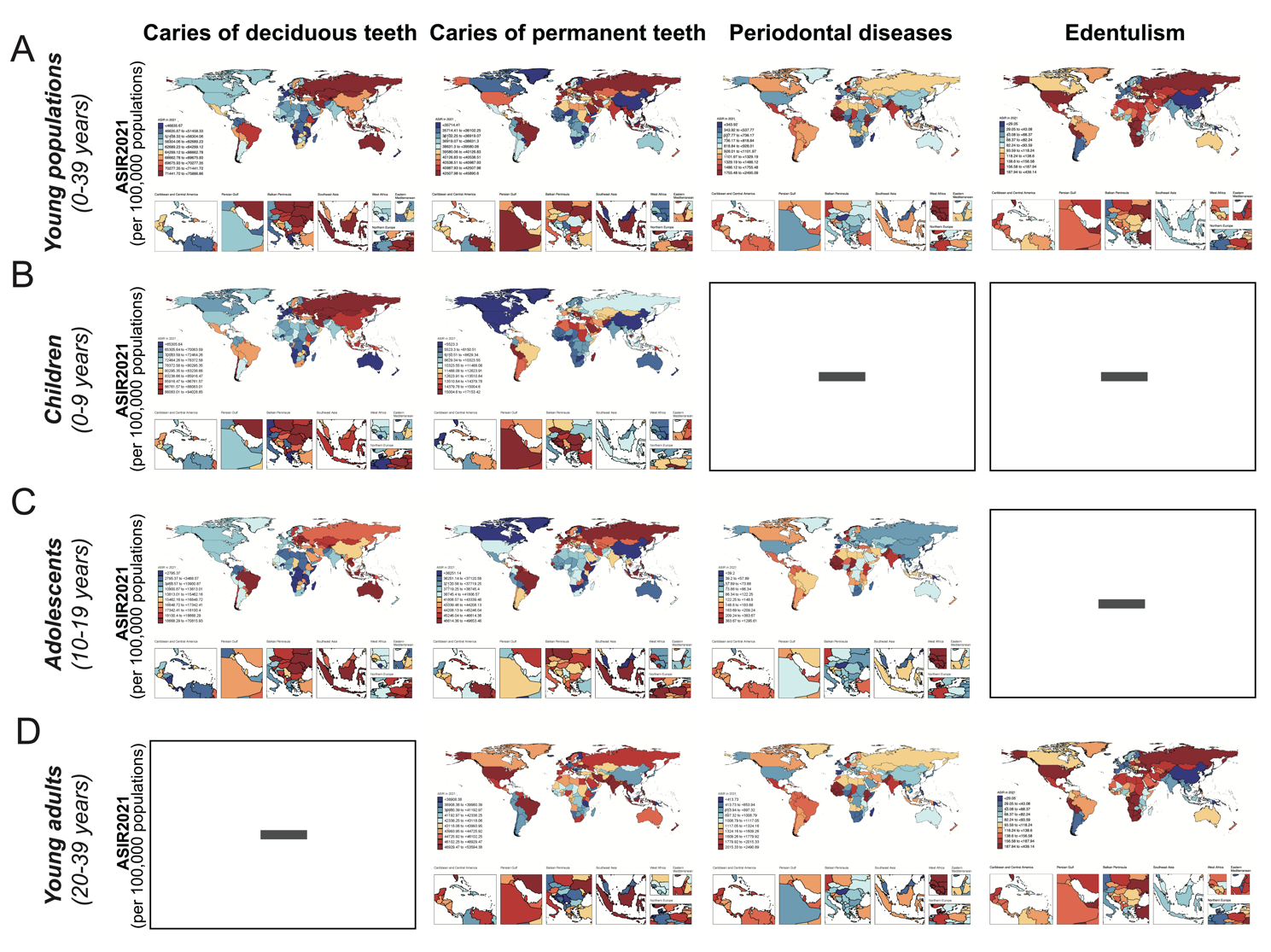


1. The distribution of age-standardized incidence rates per 100,000 populations in young population (0-39 years) for oral disorders across 204 countries and territories in 2021.
2. The distribution of age-standardized incidence rates per 100,000 populations in children (0-9 years) for oral disorders across 204 countries and territories in 2021.
3. The distribution of age-standardized incidence rates per 100,000 populations in adolescents (10-19 years) for oral disorders across 204 countries and territories in 2021.
4. The distribution of age-standardized incidence rates per 100,000 populations in young adults (20-39 years) for oral disorders across 204 countries and territories in 2021.

ASIR, age-standardized incidence rate. The link to the base layer of the map: https://www.resdc.cn/data.aspx?DATAID=205.

## Fig C. The distribution of age-standardized prevalence rates per 100,000 populations of oral disorders for 204 countries and territories in 2021.


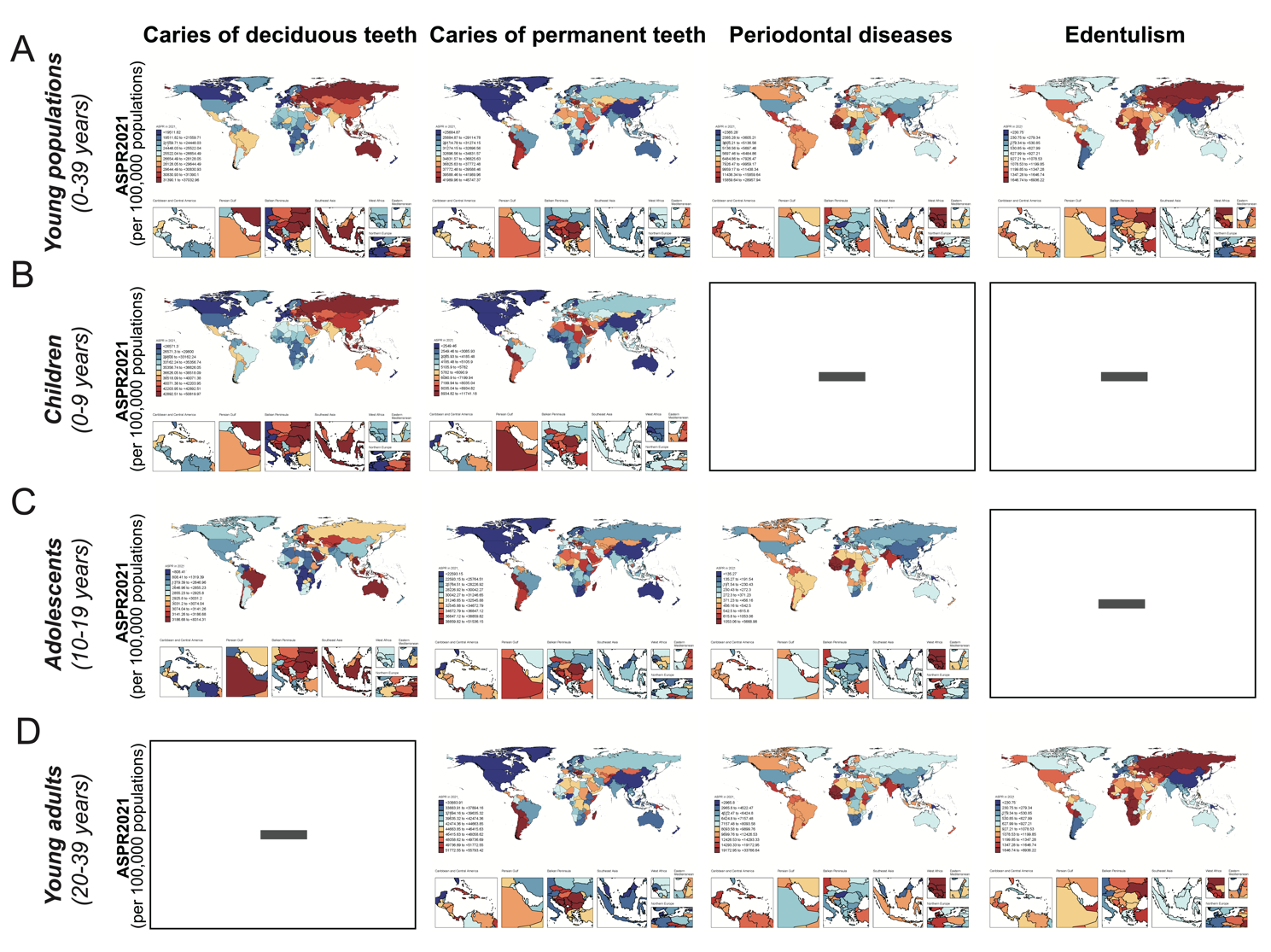


1. The distribution of age-standardized prevalence rates per 100,000 populations in young population (0-39 years) for oral disorders across 204 countries and territories in 2021.
2. The distribution of age-standardized prevalence rates per 100,000 populations in children (0-9 years) for oral disorders across 204 countries and territories in 2021.
3. The distribution of age-standardized prevalence rates per 100,000 populations in adolescents (10-19 years) for oral disorders across 204 countries and territories in 2021.
4. The distribution of age-standardized prevalence rates per 100,000 populations in young adults (20-39 years) for oral disorders across 204 countries and territories in 2021.

ASPR, age-standardized prevalence rate. The link to the base layer of the map: https://www.resdc.cn/data.aspx?DATAID=205.

## Fig D. The distribution of age-standardized YLD rates per 100,000 populations of oral disorders for 204 countries and territories in 2021 (with unified the scale across all plots).


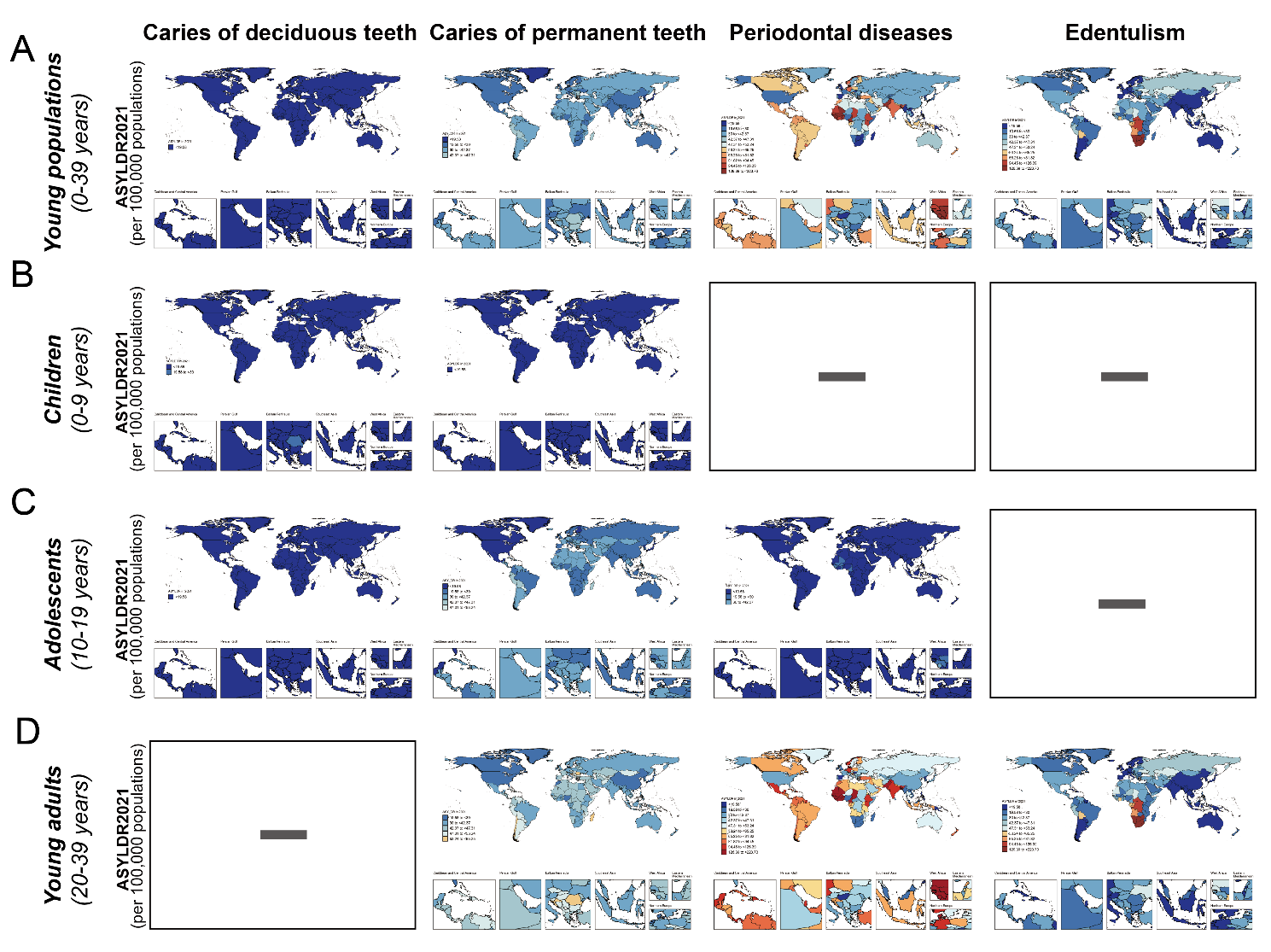


1. The distribution of age-standardized YLD rates per 100,000 populations in young population (0-39 years) for oral disorders across 204 countries and territories in 2021.
2. The distribution of age-standardized YLD rates per 100,000 populations in children (0-9 years) for oral disorders across 204 countries and territories in 2021.
3. The distribution of age-standardized YLD rates per 100,000 populations in adolescents (10-19 years) for oral disorders across 204 countries and territories in 2021.
4. The distribution of age-standardized YLD rates per 100,000 populations in young adults (20-39 years) for oral disorders across 204 countries and territories in 2021.

ASYLDR, age-standardized years lived with disability rate. The link to the base layer of the map: https://www.resdc.cn/data.aspx?DATAID=205.

## Fig E. The age-standardized rates per 100,000 populations and number of the incidence of oral disorders across different sexes in the period 1990-2021


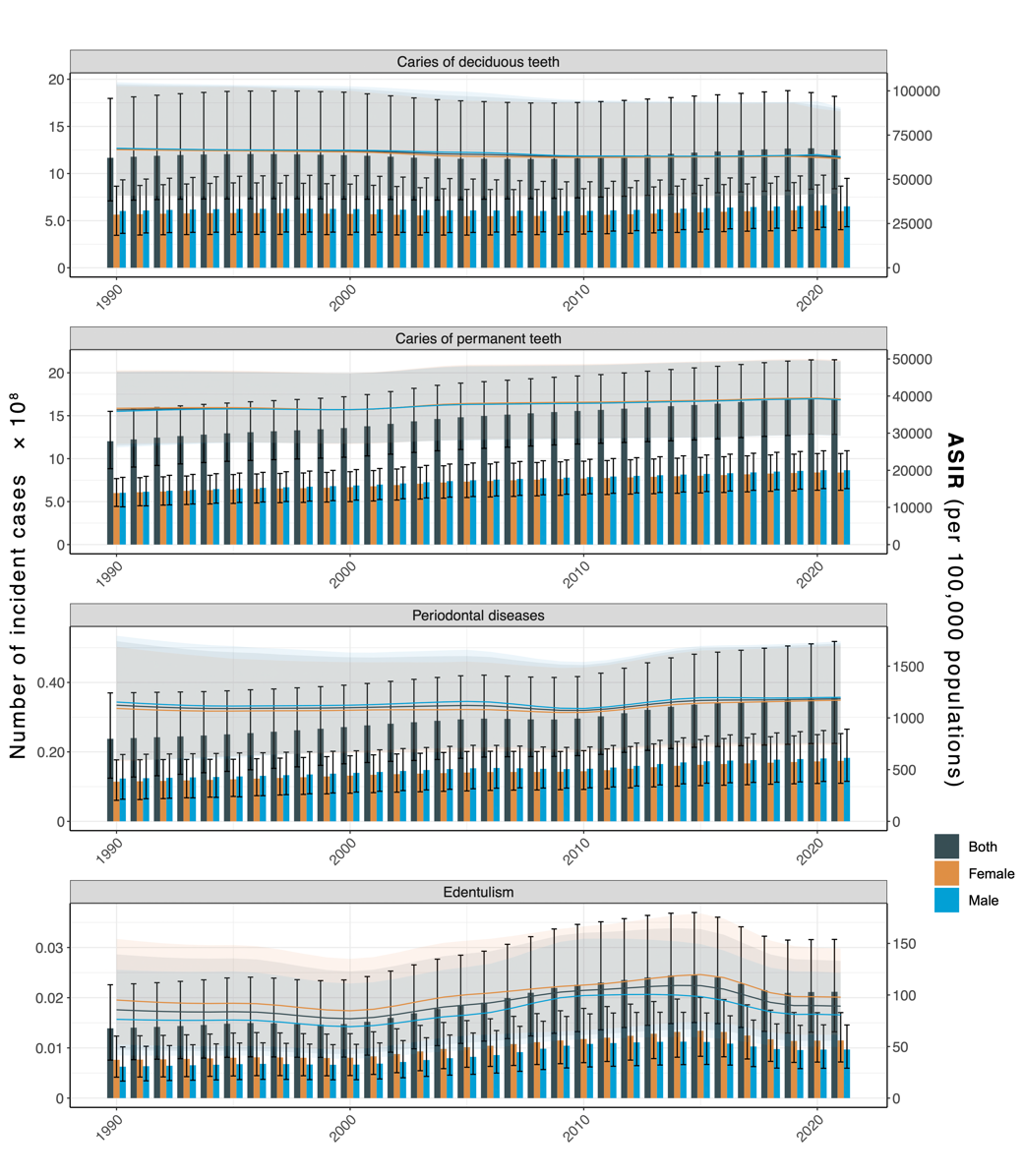


The age-standardized incidence rate per 100,000 populations and number of incident cases for all sexes in the period 1990-2021, of untreated caries deciduous teeth, untreated caries of permanent teeth, periodontal diseases, and edentulism. ASIR, age-standardized incidence rate.

## Fig F. The age-standardized rates per 100,000 populations and number of the prevalence of oral disorders across different sexes in the period 1990-2021


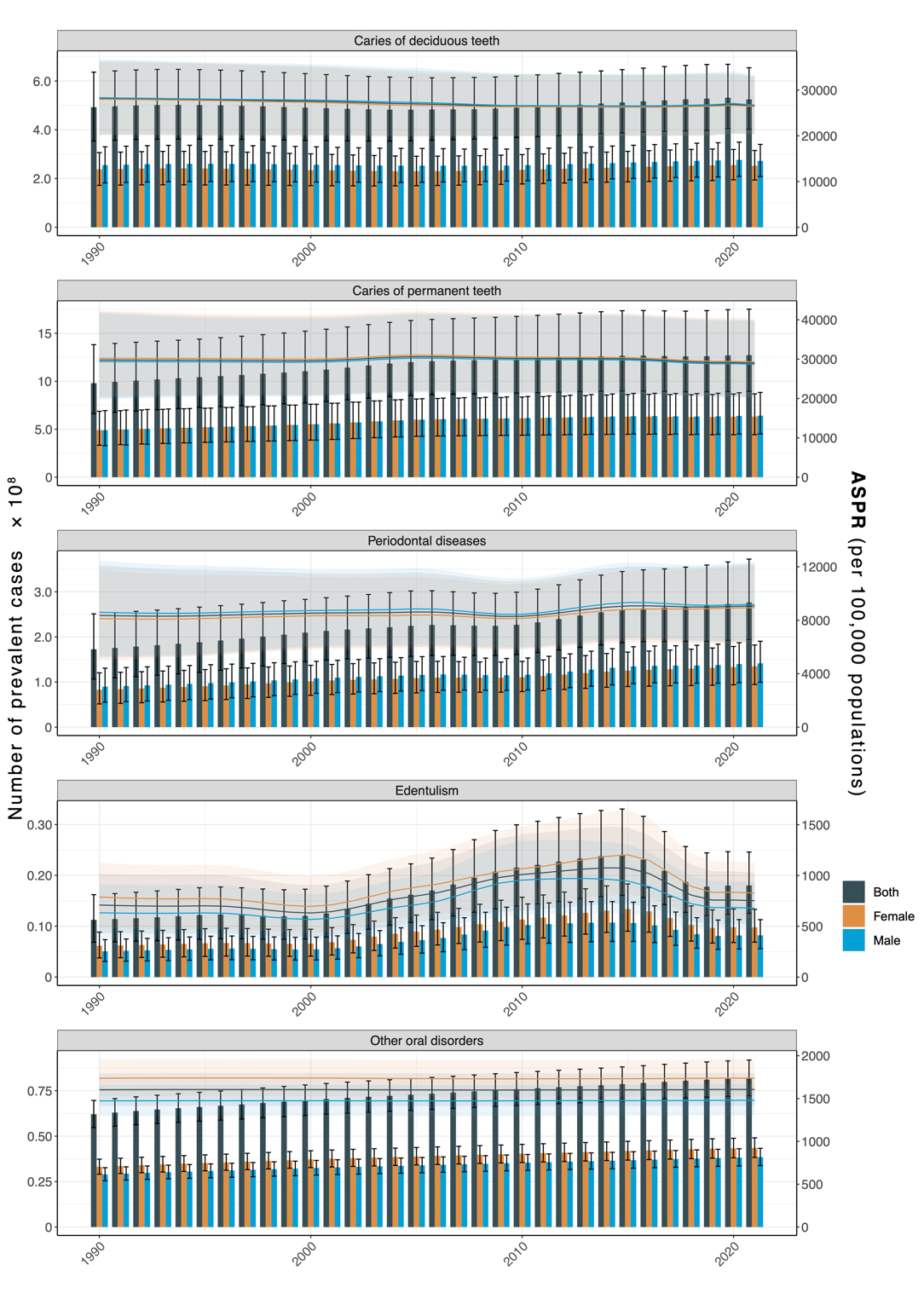


The age-standardized prevalence rate per 100,000 populations and number of prevalent cases for all sexes in the period 1990-2021, of untreated caries deciduous teeth, untreated caries of permanent teeth, periodontal diseases, edentulism, and other oral disorders. ASPR, age-standardized prevalence rate.

## Fig G. The age-standardized rates per 100,000 populations and number of the YLDs of oral disorders across different sexes in the period 1990-2021


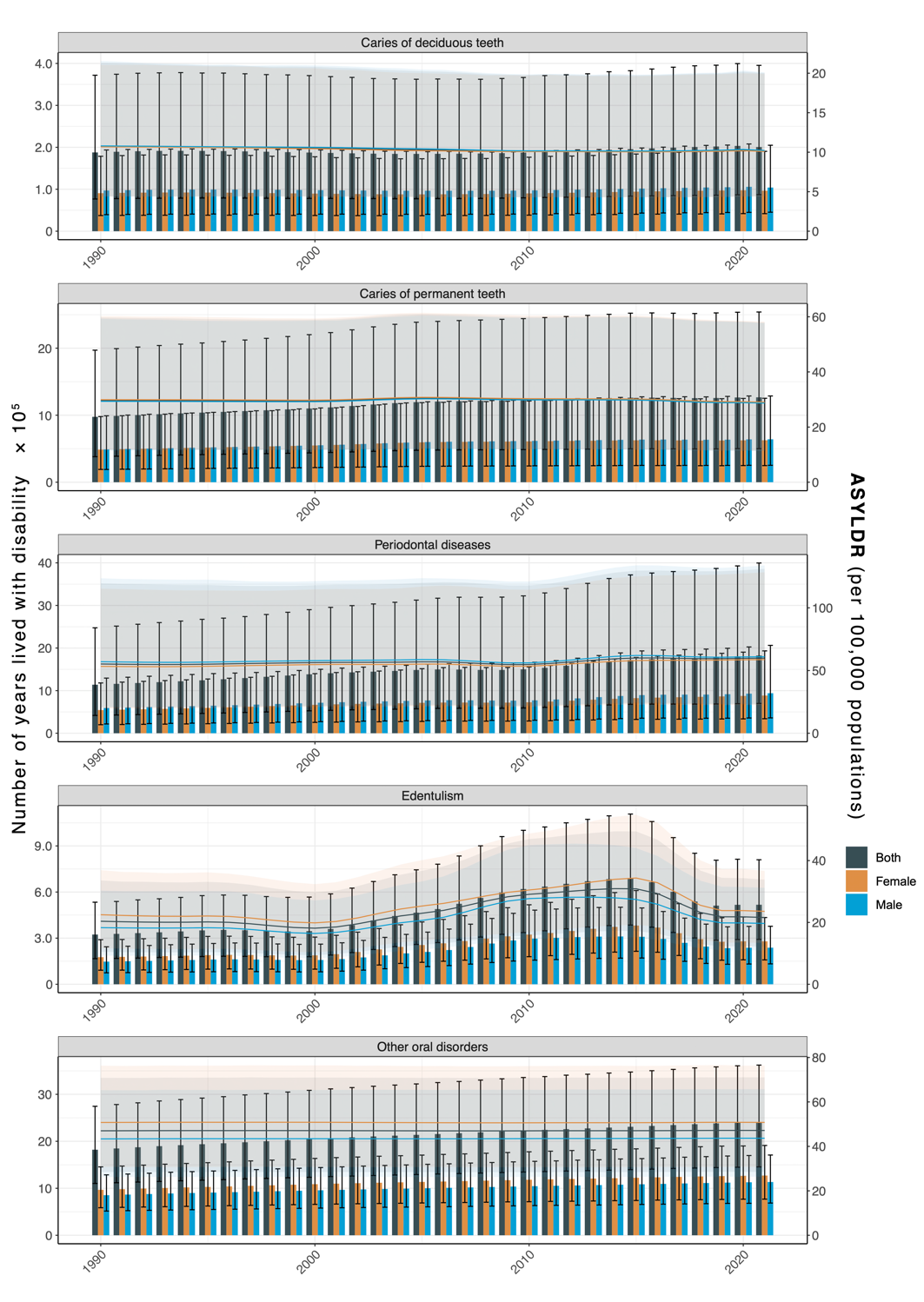


The age-standardized YLD rate per 100,000 populations and number of YLDs for all sexes in the period 1990-2021, of untreated caries deciduous teeth, untreated caries of permanent teeth, periodontal diseases, edentulism, and other oral disorders. ASYLDR, age-standardized years lived with disability rate.

## Fig H. The temporal trends in age-standardized incidence rates per 100,000 populations of oral disorders from 1990 to 2021, and joinpoints, annual percentage changes, and average annual percentage changes in the period 1990-2021.


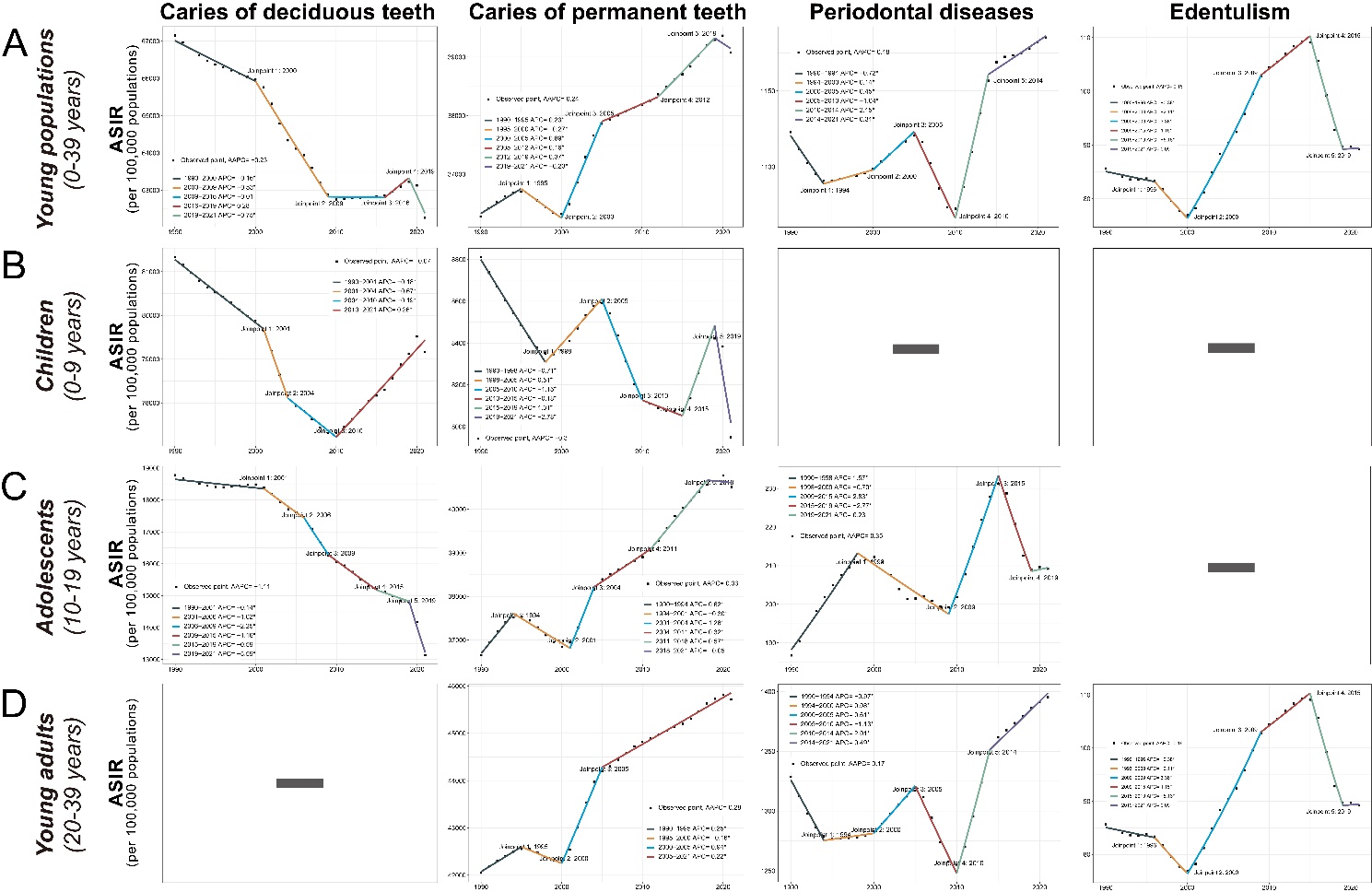


1. The temporal trends in age-standardized incidence rates per 100,000 populations in young population (0-39 years) for oral disorders from 1990 to 2021.
2. The temporal trends in age-standardized incidence rates per 100,000 populations in children (0-9 years) for oral disorders from 1990 to 2021.
3. The temporal trends in age-standardized incidence rates per 100,000 populations in adolescents (10-19 years) for oral disorders from 1990 to 2021.
4. The temporal trends in age-standardized incidence rates per 100,000 populations in young adults (20-39 years) for oral disorders from 1990 to 2021.

ASIR, age-standardized incidence rate; APC, annual percentage changes; AAPC, average annual percentage changes.

## Fig I. The temporal trends in age-standardized prevalence rates per 100,000 populations of oral disorders from 1990 to 2021, and joinpoints, annual percentage changes, and average annual percentage changes in the period 1990-2021.


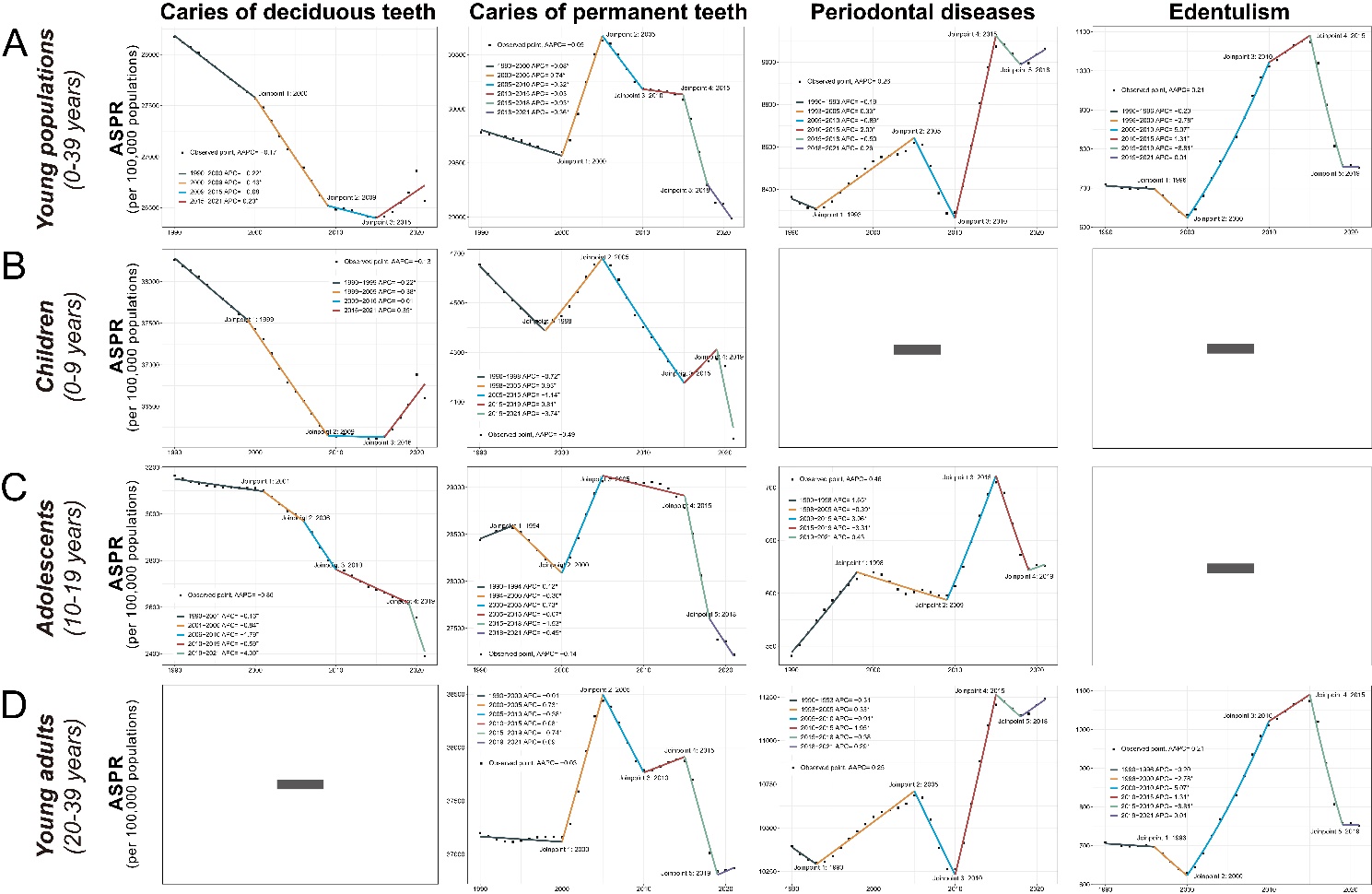


1. The temporal trends in age-standardized prevalence rates per 100,000 populations in young population (0-39 years) for oral disorders from 1990 to 2021.
2. The temporal trends in age-standardized prevalence rates per 100,000 populations in children (0-9 years) for oral disorders from 1990 to 2021.
3. The temporal trends in age-standardized prevalence rates per 100,000 populations in adolescents (10-19 years) for oral disorders from 1990 to 2021.
4. The temporal trends in age-standardized prevalence rates per 100,000 populations in young adults (20-39 years) for oral disorders from 1990 to 2021.

ASPR, age-standardized prevalence rate; APC, annual percentage changes; AAPC, average annual percentage changes.

## Fig J. Changes in the number of incidence cases of oral disorders attributed to different drivers from 1990 to 2021 at the global level and by SDI quintiles.

**
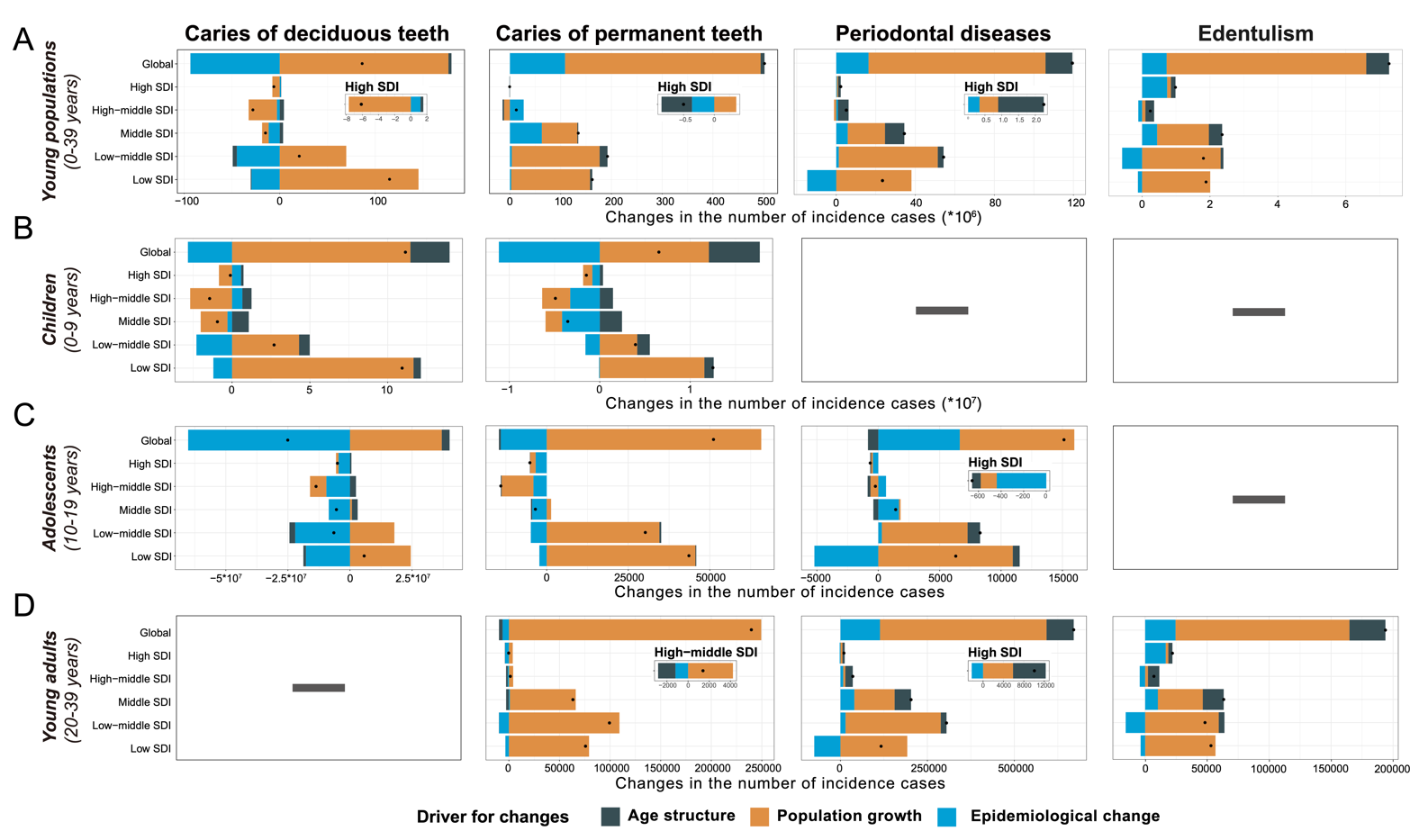
**

1. Changes in the number of incidence cases for oral disorders among young population (0-39 years) attributed to different drivers from 1990 to 2021 at the global level and by SDI quintiles.
2. Changes in the number of incidence cases for oral disorders among children (0-9 years) attributed to different drivers from 1990 to 2021 at the global level and by SDI quintiles.
3. Changes in the number of incidence cases for oral disorders among adolescents (10-19 years) attributed to different drivers from 1990 to 2021 at the global level and by SDI quintiles.
4. Changes in the number of incidence cases for oral disorders among young adults (20-39 years) attributed to different drivers from 1990 to 2021 at the global level and by SDI quintiles.

SDI, sociodemographic index.

## Fig K. Changes in the number of prevalence cases of oral disorders attributed to different drivers from 1990 to 2021 at the global level and by SDI quintiles.

**
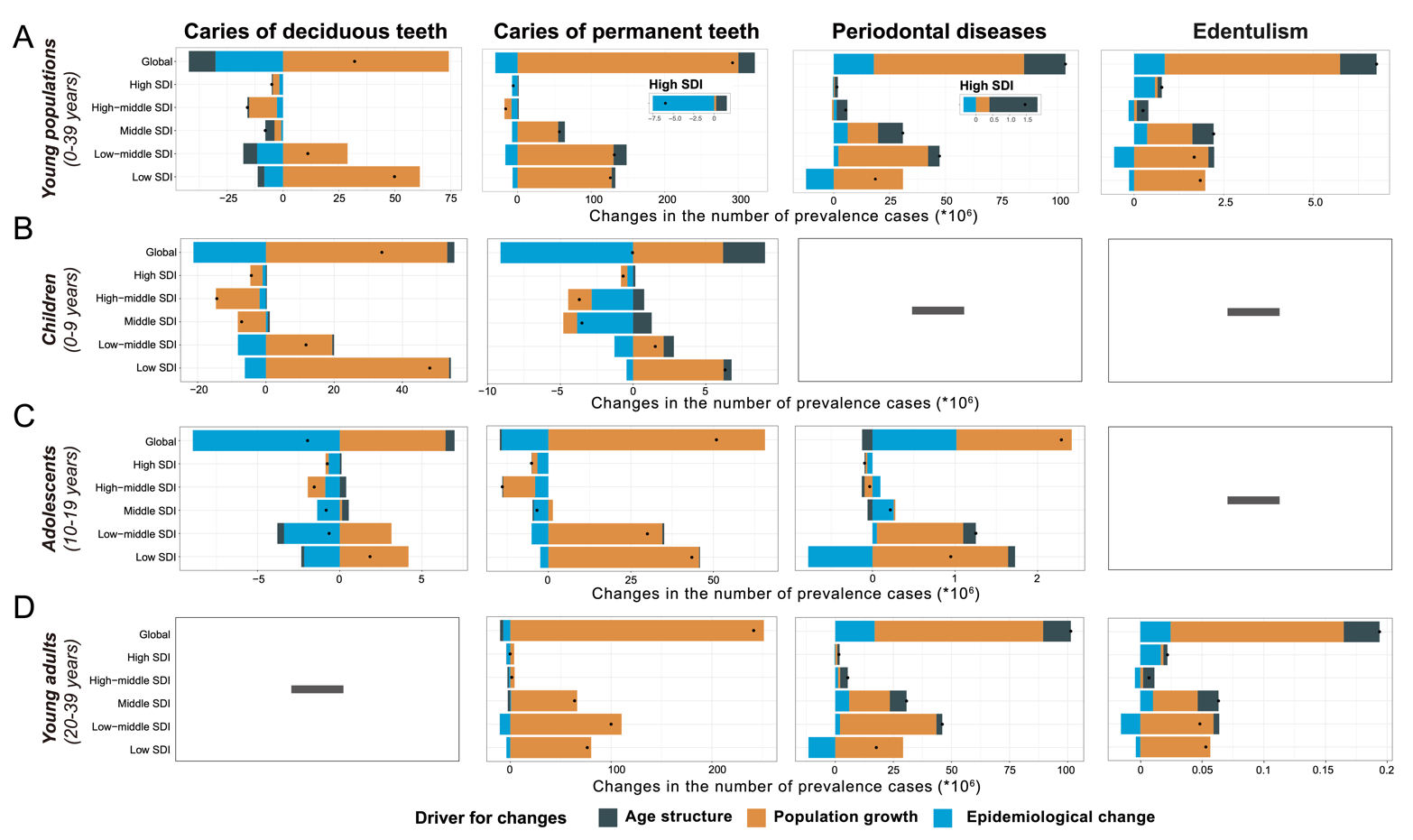
**

1. Changes in the number of prevalence cases for oral disorders among young population (0-39 years) attributed to different drivers from 1990 to 2021 at the global level and by SDI quintiles.
2. Changes in the number of prevalence cases for oral disorders among children (0-9 years) attributed to different drivers from 1990 to 2021 at the global level and by SDI quintiles.
3. Changes in the number of prevalence cases for oral disorders among adolescents (10-19 years) attributed to different drivers from 1990 to 2021 at the global level and by SDI quintiles.
4. Changes in the number of prevalence cases for oral disorders among young adults (20-39 years) attributed to different drivers from 1990 to 2021 at the global level and by SDI quintiles.

SDI, sociodemographic index.

## Fig L. SDI-related health inequality regression lines indicating absolute inequality in the ASIR of oral disorders, 1990 and 2021.

**
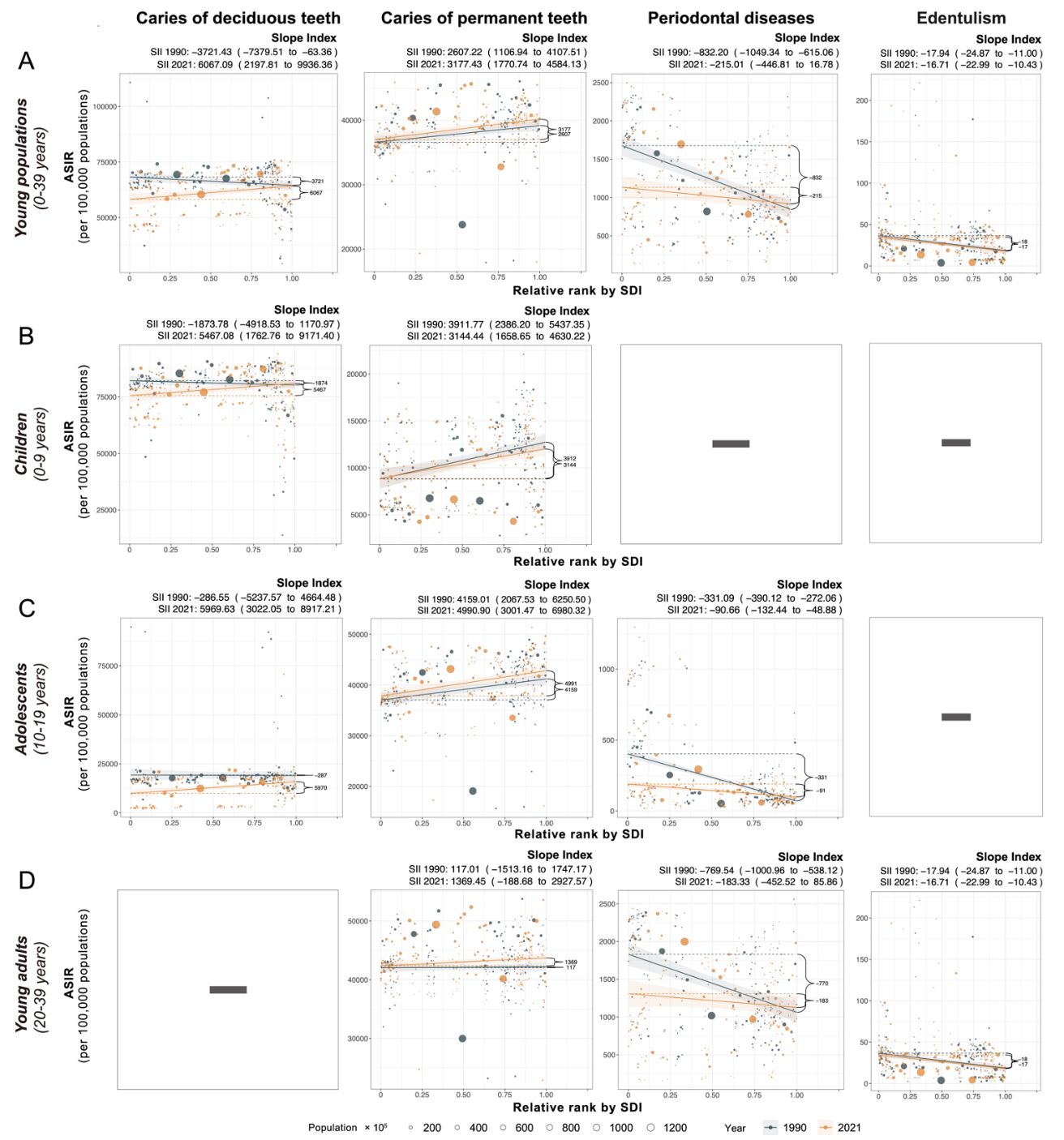
**

1. SDI-related health inequality regression lines indicating absolute inequality in the ASIR of oral disorders among young population (0-39 years).
2. SDI-related health inequality regression lines indicating absolute inequality in the ASIR of oral disorders among children (0-9 years).
3. SDI-related health inequality regression lines indicating absolute inequality in the ASIR of oral disorders among adolescents (10-19 years).
4. SDI-related health inequality regression lines indicating absolute inequality in the ASIR of oral disorders among young adults (20-39 years).

Dots represent countries and territories; dot size represents population. CI, confidence interval; SDI, sociodemographic index; ASIR, age-standardized incidence rate.

## Fig M. SDI-related health inequality regression lines indicating absolute inequality in the ASPR of oral disorders, 1990 and 2021.

**
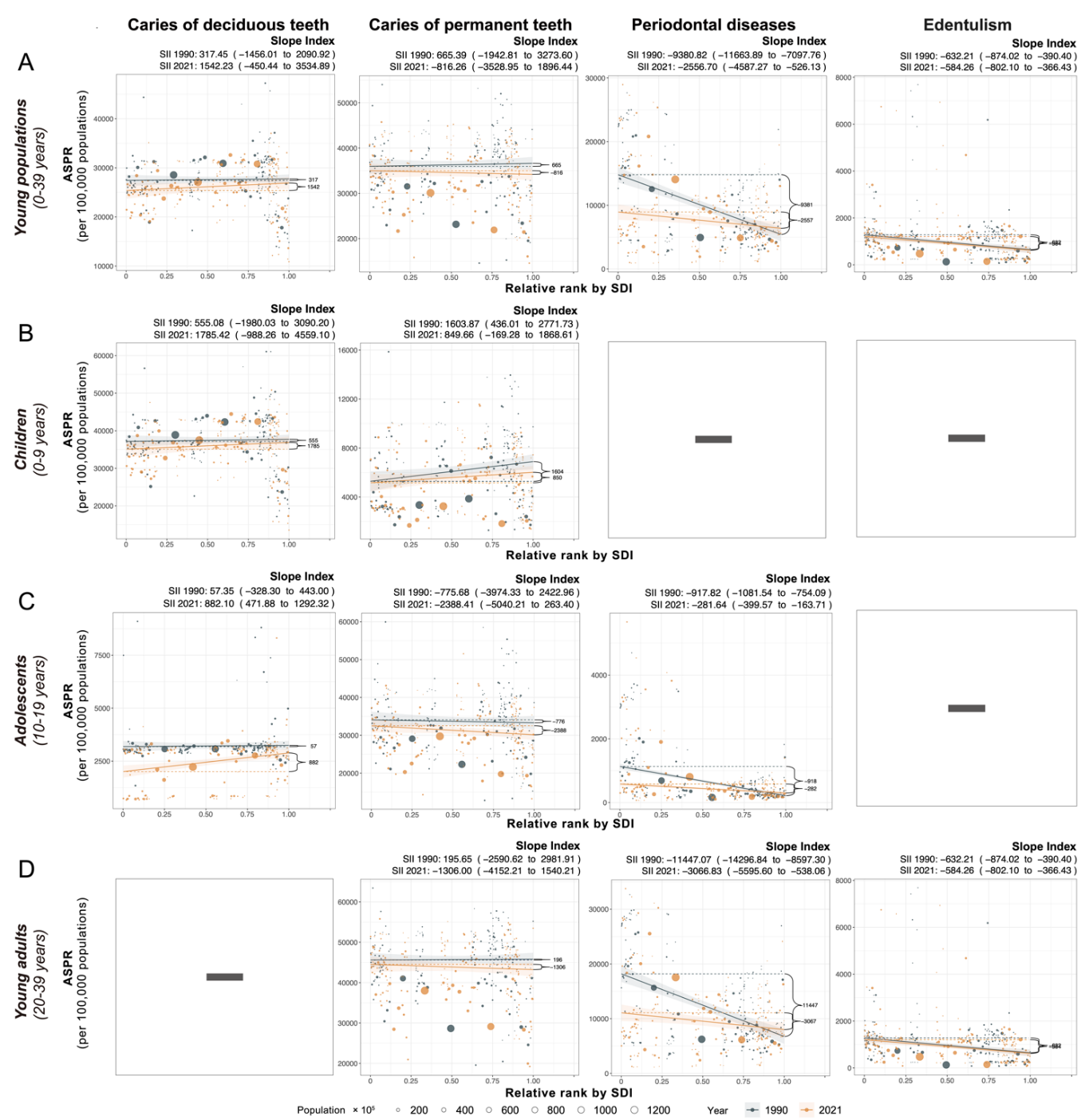
**

1. SDI-related health inequality regression lines indicating absolute inequality in the ASPR of oral disorders among young population (0-39 years).
2. SDI-related health inequality regression lines indicating absolute inequality in the ASPR of oral disorders among children (0-9 years).
3. SDI-related health inequality regression lines indicating absolute inequality in the ASPR of oral disorders among adolescents (10-19 years).
4. SDI-related health inequality regression lines indicating absolute inequality in the ASPR of oral disorders among young adults (20-39 years).

Dots represent countries and territories; dot size represents population. CI, confidence interval; SDI, sociodemographic index; ASPR, age-standardized prevalence rate.

## Fig N. SDI-related health inequality concentration curves indicating relative inequality in the ASIR of oral disorders, 1990 and 2021.

**
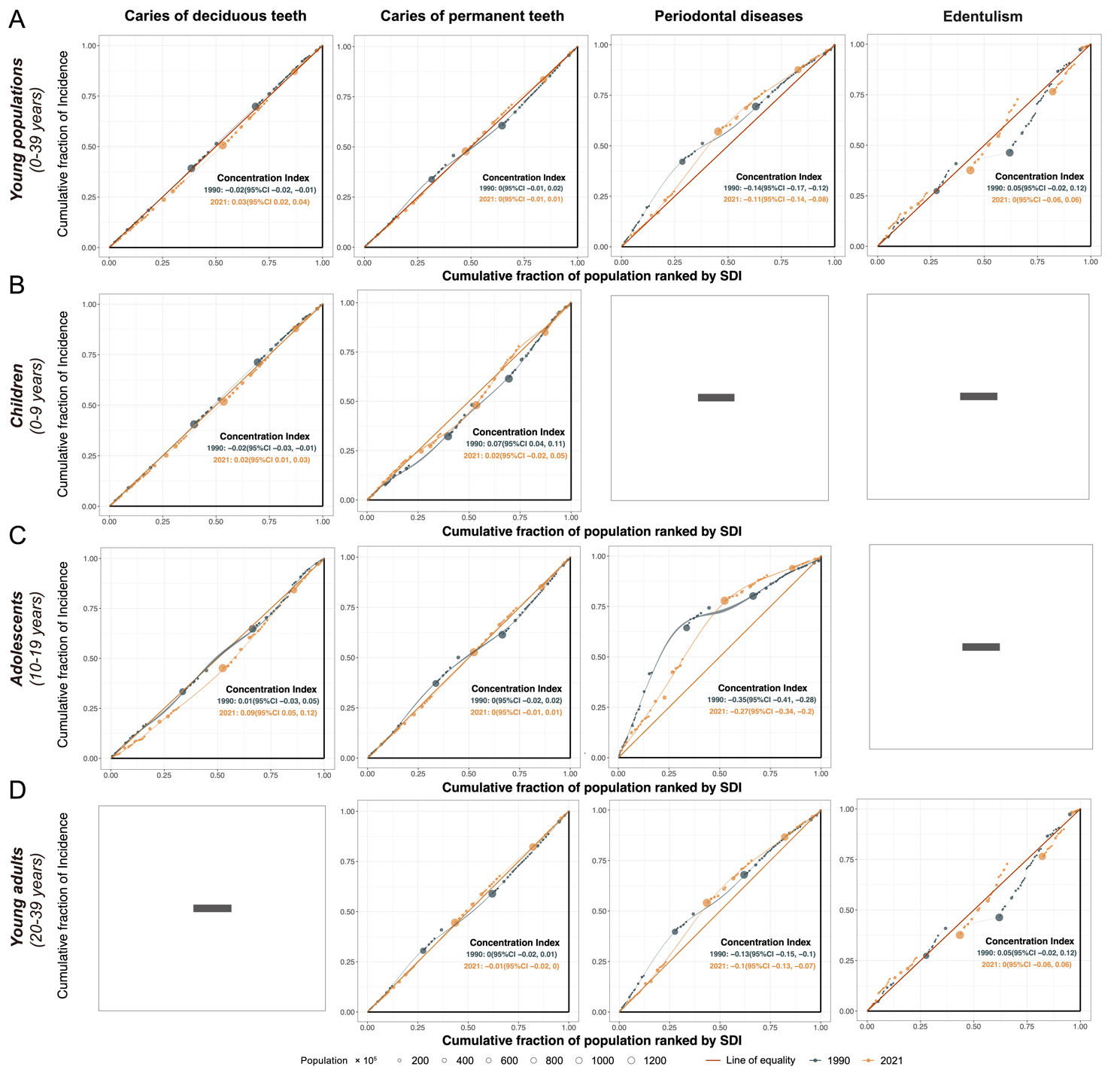
**

1. SDI-related health inequality concentration curves indicating relative inequality in the ASIR of oral disorders among young population (0-39 years).
2. SDI-related health inequality concentration curves indicating relative inequality in the ASIR of oral disorders among children (0-9 years).
3. SDI-related health inequality concentration curves indicating relative inequality in the ASIR of oral disorders among adolescents (10-19 years).
4. SDI-related health inequality concentration curves indicating relative inequality in the ASIR of oral disorders among young adults (20-39 years).

Dots represent countries and territories; dot size represents population. CI, confidence interval; SDI, sociodemographic index; ASIR, age-standardized incidence rate.

## Fig O. SDI-related health inequality concentration curves indicating relative inequality in the ASPR of oral disorders, 1990 and 2021.


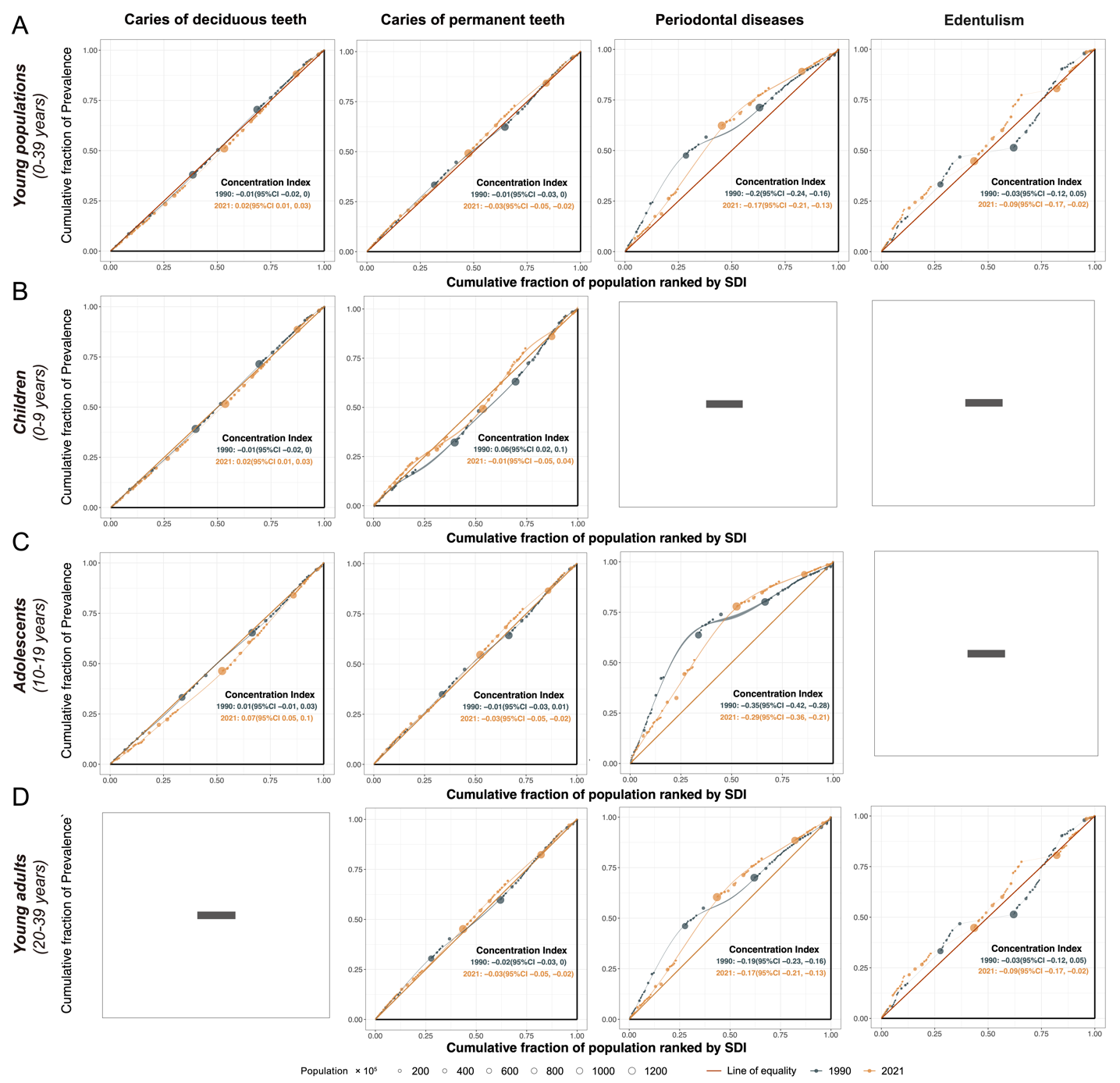


1. SDI-related health inequality concentration curves indicating relative inequality in the ASPR of oral disorders among young population (0-39 years).
2. SDI-related health inequality concentration curves indicating relative inequality in the ASPR of oral disorders among children (0-9 years).
3. SDI-related health inequality concentration curves indicating relative inequality in the ASPR of oral disorders among adolescents (10-19 years).
4. SDI-related health inequality concentration curves indicating relative inequality in the ASPR of oral disorders among young adults (20-39 years).

Dots represent countries and territories; dot size represents population. CI, confidence interval; SDI, sociodemographic index; ASPR, age-standardized prevalence rate.

## Fig P. The global distribution and temporal trends of ASPRs and ASYLDRs per 100,000 populations of other oral disorders in 2021.


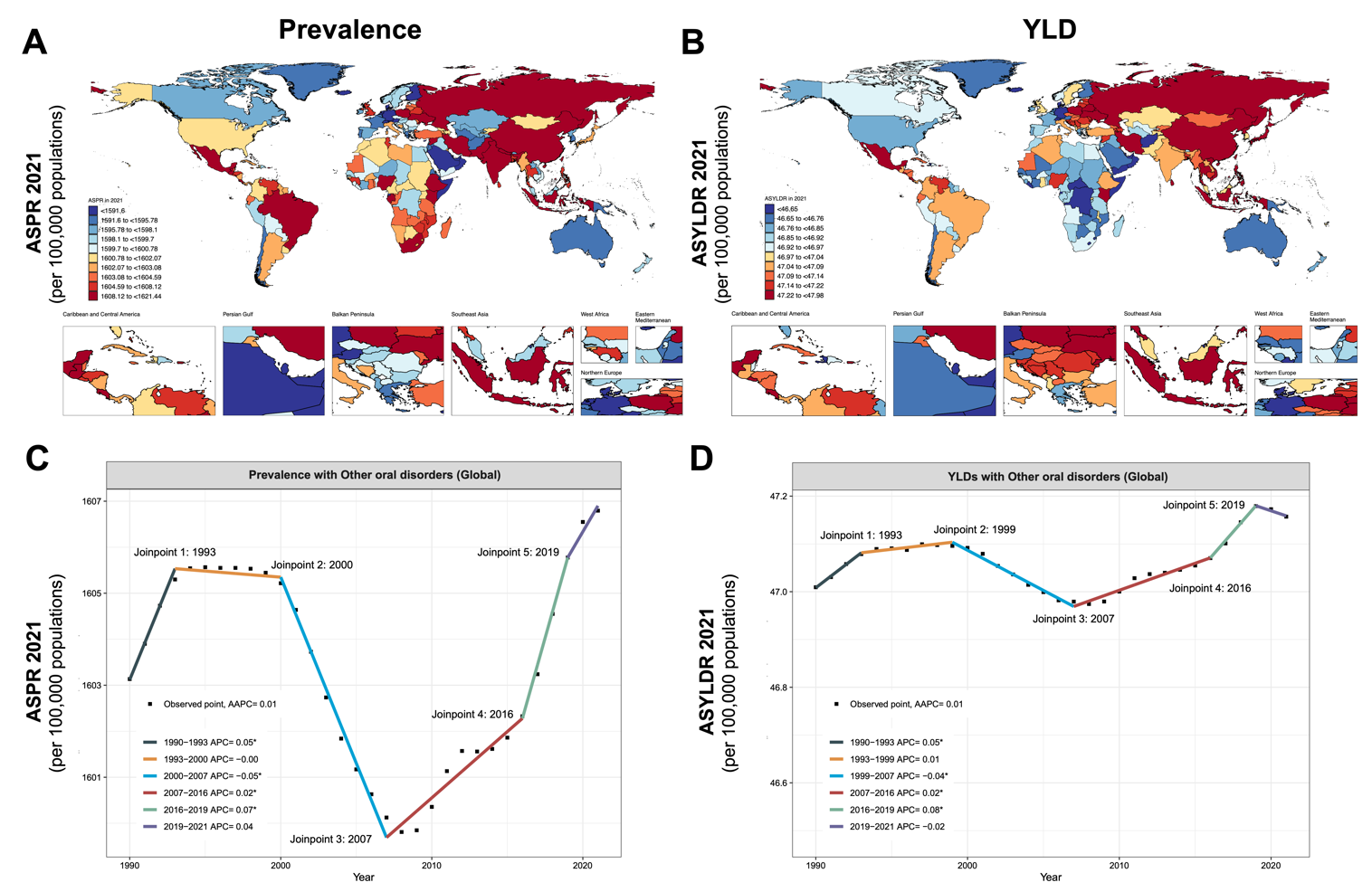


1. The distribution of age-standardized prevalence rates per 100,000 populations in young population (0-39 years) for other oral disorders across 204 countries and territories in 2021.
2. The distribution of age-standardized YLD rates per 100,000 populations in young population (0-39 years) for other oral disorders across 204 countries and territories in 2021.
3. The temporal trends in age-standardized prevalence rates per 100,000 populations in young population (0-39 years) for oral disorders from 1990 to 2021, and joinpoints, annual percentage changes, and average annual percentage changes in the period 1990-2021.
4. The temporal trends in age-standardized YLD rates per 100,000 populations in young population (0-39 years) for oral disorders from 1990 to 2021, and joinpoints, annual percentage changes, and average annual percentage changes in the period 1990-2021.

ASPR, age-standardized prevalence rate; ASYLDR, age-standardized years lived with disability rate; APC, annual percentage changes; AAPC, average annual percentage changes.

## Fig Q. Changes in the number of prevalence cases and YLDs of other oral disorders attributed to different drivers from 1990 to 2021 by SDI quintiles, as well as the SDI-related absolute inequality and relative inequality in the prevalence and YLDs of other oral disorders, 1990 and 2021.


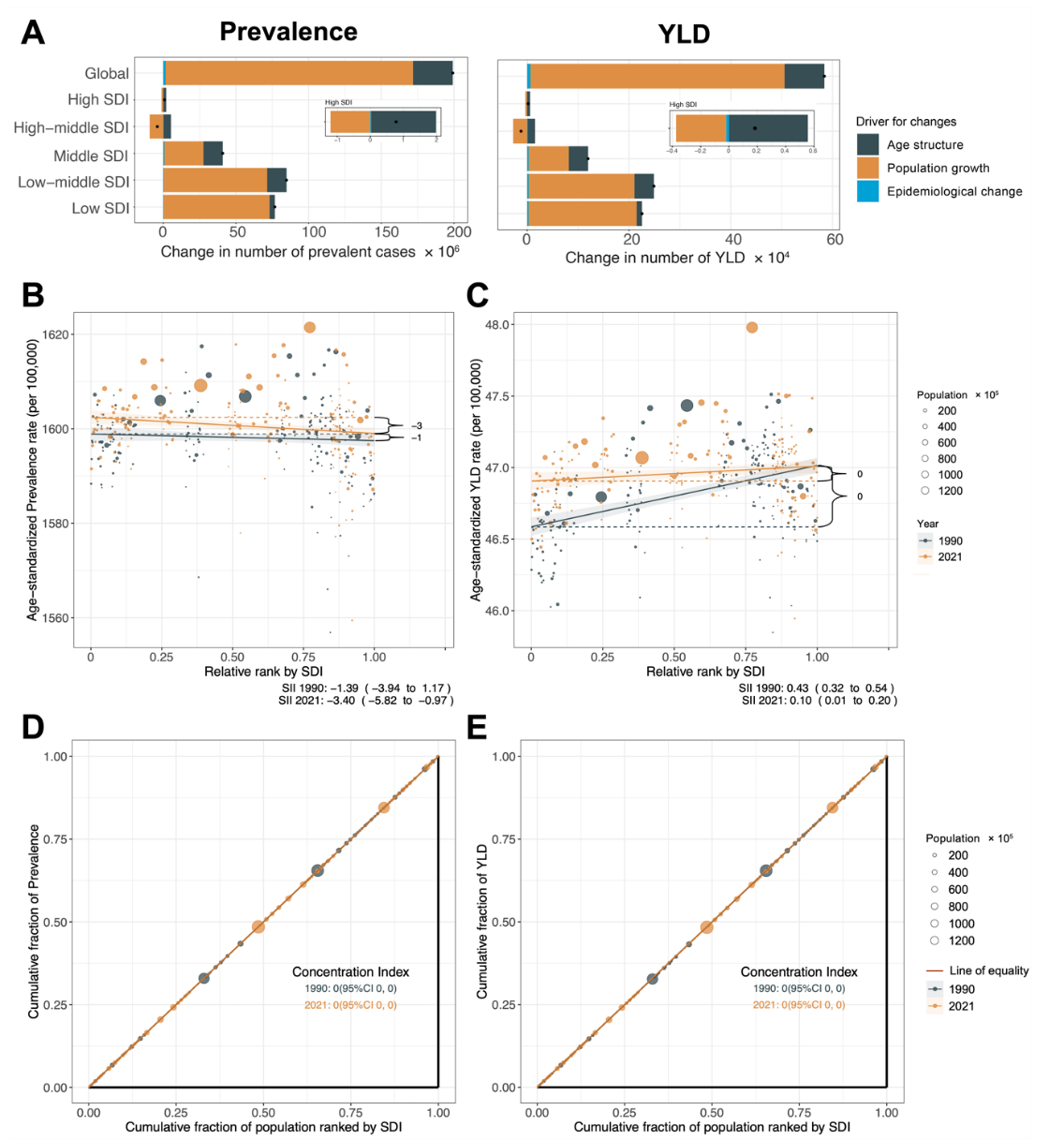


1. Changes in the number of prevalence cases and YLDs for other oral disorders among young population (0-39 years) attributed to different drivers from 1990 to 2021 at the global level and by SDI quintiles.
2. SDI-related health inequality regression lines indicating absolute inequality in the ASPR of other oral disorders among young population (0-39 years).
3. SDI-related health inequality regression lines indicating absolute inequality in the ASYLDR of other oral disorders among young population (0-39 years).
4. SDI-related health inequality concentration curves indicating relative inequality in the ASPR of other oral disorders among young population (0-39 years).
5. SDI-related health inequality concentration curves indicating relative inequality in the ASYLDR of other oral disorders among young population (0-39 years).

Dots represent countries and territories; dot size represents population. CI, confidence interval; SDI, sociodemographic index; ASPR, age-standardized prevalence rate; ASYLDR, age-standardized years lived with disability rate.

# Supplementary Tables

## Table A. SDI Quintile for 204 countries and territories and the reference quintiles in 2021

| **GBD 2021 SDI Reference Quintile Values** | | |  |
| --- | --- | --- | --- |
| Lower bound | Upper bound | SDI Quintile |  |
| 0.810295989 | 1 | High SDI |  |
| 0.711974622 | 0.810295989 | High-middle SDI |  |
| 0.618829445 | 0.711974622 | Middle SDI |  |
| 0.465815803 | 0.618829445 | Low-middle SDI |  |
| 0 | 0.465815803 | Low SDI |  |
|  |  |  |  |
| **GBD 2021 SDI Quintile Values** | | | Notes |
| Location Name | 2021 SDI Index Value | SDI Quintile |
| Switzerland | 0.933531726 | High SDI | The 10 richest countries or territories |
| Norway | 0.916631633 | High SDI |
| Monaco | 0.909519124 | High SDI |
| Germany | 0.903515704 | High SDI |
| Denmark | 0.897314038 | High SDI |
| Netherlands | 0.888375951 | High SDI |
| San Marino | 0.887883596 | High SDI |
| Sweden | 0.887384361 | High SDI |
| Republic of Korea | 0.887195638 | High SDI |
| Luxembourg | 0.884636327 | High SDI |
| Taiwan (Province of China) | 0.875139514 | High SDI |  |
| Iceland | 0.874628639 | High SDI |  |
| Ireland | 0.873989853 | High SDI |  |
| Canada | 0.873181934 | High SDI |  |
| Japan | 0.871459701 | High SDI |  |
| Andorra | 0.869895393 | High SDI |  |
| United States of America | 0.863243823 | High SDI |  |
| Finland | 0.860244219 | High SDI |  |
| United Kingdom | 0.858444983 | High SDI |  |
| Lithuania | 0.857613278 | High SDI |  |
| Singapore | 0.856235308 | High SDI |  |
| Austria | 0.854558286 | High SDI |  |
| Belgium | 0.853674059 | High SDI |  |
| New Zealand | 0.850145187 | High SDI |  |
| United Arab Emirates | 0.849740335 | High SDI |  |
| Kuwait | 0.846802486 | High SDI |  |
| Qatar | 0.846704498 | High SDI |  |
| Estonia | 0.845787294 | High SDI |  |
| Australia | 0.844269408 | High SDI |  |
| Slovenia | 0.842633141 | High SDI |  |
| France | 0.837816091 | High SDI |  |
| Cyprus | 0.835648571 | High SDI |  |
| Greenland | 0.835640003 | High SDI |  |
| Latvia | 0.830715451 | High SDI |  |
| Czechia | 0.828510085 | High SDI |  |
| Puerto Rico | 0.824543903 | High SDI |  |
| United States Virgin Islands | 0.822988043 | High SDI |  |
| Bermuda | 0.821319794 | High SDI |  |
| Saudi Arabia | 0.814515567 | High SDI |  |
| Poland | 0.812073312 | High SDI |  |
| Brunei Darussalam | 0.810288851 | High-middle SDI |  |
| Russian Federation | 0.809111108 | High-middle SDI |  |
| Israel | 0.809091066 | High-middle SDI |  |
| Slovakia | 0.808329132 | High-middle SDI |  |
| Italy | 0.805537426 | High-middle SDI |  |
| Bahamas | 0.805143711 | High-middle SDI |  |
| Guam | 0.80216771 | High-middle SDI |  |
| Malta | 0.801853922 | High-middle SDI |  |
| Croatia | 0.799069214 | High-middle SDI |  |
| Montenegro | 0.796532951 | High-middle SDI |  |
| Serbia | 0.79221264 | High-middle SDI |  |
| Greece | 0.791882294 | High-middle SDI |  |
| Hungary | 0.791024669 | High-middle SDI |  |
| Belarus | 0.784114127 | High-middle SDI |  |
| Cook Islands | 0.778251758 | High-middle SDI |  |
| Northern Mariana Islands | 0.777504838 | High-middle SDI |  |
| Oman | 0.773801229 | High-middle SDI |  |
| Chile | 0.770149297 | High-middle SDI |  |
| Spain | 0.76948336 | High-middle SDI |  |
| Trinidad and Tobago | 0.769401094 | High-middle SDI |  |
| Romania | 0.766321392 | High-middle SDI |  |
| Bulgaria | 0.764641037 | High-middle SDI |  |
| Ukraine | 0.761045561 | High-middle SDI |  |
| Saint Kitts and Nevis | 0.756332641 | High-middle SDI |  |
| Palau | 0.754590186 | High-middle SDI |  |
| Bahrain | 0.752218099 | High-middle SDI |  |
| North Macedonia | 0.750954677 | High-middle SDI |  |
| Antigua and Barbuda | 0.749849952 | High-middle SDI |  |
| Dominica | 0.747381853 | High-middle SDI |  |
| Barbados | 0.74706542 | High-middle SDI |  |
| Portugal | 0.745394909 | High-middle SDI |  |
| Malaysia | 0.742552841 | High-middle SDI |  |
| Lebanon | 0.741226017 | High-middle SDI |  |
| Libya | 0.73508433 | High-middle SDI |  |
| Argentina | 0.733528396 | High-middle SDI |  |
| Georgia | 0.733123642 | High-middle SDI |  |
| Republic of Moldova | 0.732393345 | High-middle SDI |  |
| Seychelles | 0.727579445 | High-middle SDI |  |
| American Samoa | 0.726267628 | High-middle SDI |  |
| Niue | 0.72621855 | High-middle SDI |  |
| Jordan | 0.725420238 | High-middle SDI |  |
| Bosnia and Herzegovina | 0.72296408 | High-middle SDI |  |
| Uruguay | 0.721713499 | High-middle SDI |  |
| China | 0.71867919 | High-middle SDI |  |
| Kazakhstan | 0.718331647 | High-middle SDI |  |
| Mauritius | 0.717977109 | High-middle SDI |  |
| Turkey | 0.713246106 | High-middle SDI |  |
| Albania | 0.706888685 | Middle SDI |  |
| Panama | 0.706659844 | Middle SDI |  |
| Costa Rica | 0.704369665 | Middle SDI |  |
| Armenia | 0.702496602 | Middle SDI |  |
| Sri Lanka | 0.701371778 | Middle SDI |  |
| Iran (Islamic Republic of) | 0.69729326 | Middle SDI |  |
| Azerbaijan | 0.695410598 | Middle SDI |  |
| Tokelau | 0.68701842 | Middle SDI |  |
| Jamaica | 0.68306364 | Middle SDI |  |
| Turkmenistan | 0.683039569 | Middle SDI |  |
| Thailand | 0.682657272 | Middle SDI |  |
| Tunisia | 0.681701488 | Middle SDI |  |
| South Africa | 0.681292244 | Middle SDI |  |
| Saint Lucia | 0.672601687 | Middle SDI |  |
| Grenada | 0.6693506 | Middle SDI |  |
| Cuba | 0.669331767 | Middle SDI |  |
| Fiji | 0.669068631 | Middle SDI |  |
| Ecuador | 0.665675436 | Middle SDI |  |
| Mexico | 0.66496867 | Middle SDI |  |
| Uzbekistan | 0.664964654 | Middle SDI |  |
| Equatorial Guinea | 0.663978286 | Middle SDI |  |
| Iraq | 0.662777495 | Middle SDI |  |
| Peru | 0.662036006 | Middle SDI |  |
| Algeria | 0.659720087 | Middle SDI |  |
| Indonesia | 0.657934796 | Middle SDI |  |
| Maldives | 0.657665453 | Middle SDI |  |
| Colombia | 0.65664043 | Middle SDI |  |
| Philippines | 0.651920253 | Middle SDI |  |
| Guyana | 0.650902479 | Middle SDI |  |
| Paraguay | 0.650487525 | Middle SDI |  |
| Brazil | 0.648846512 | Middle SDI |  |
| Botswana | 0.643077969 | Middle SDI |  |
| Suriname | 0.641162711 | Middle SDI |  |
| Saint Vincent and the Grenadines | 0.640886762 | Middle SDI |  |
| Gabon | 0.639080604 | Middle SDI |  |
| Palestine | 0.629201641 | Middle SDI |  |
| Tonga | 0.629100964 | Middle SDI |  |
| Nauru | 0.627549782 | Middle SDI |  |
| Syrian Arab Republic | 0.622855859 | Middle SDI |  |
| Viet Nam | 0.621620778 | Middle SDI |  |
| Dominican Republic | 0.619170694 | Middle SDI |  |
| Mongolia | 0.618744133 | Low-middle SDI |  |
| Namibia | 0.618073651 | Low-middle SDI |  |
| Belize | 0.61055234 | Low-middle SDI |  |
| Kyrgyzstan | 0.609180728 | Low-middle SDI |  |
| Bolivia (Plurinational State of) | 0.604496662 | Low-middle SDI |  |
| Egypt | 0.603962121 | Low-middle SDI |  |
| Venezuela (Bolivarian Republic of) | 0.596599587 | Low-middle SDI |  |
| Samoa | 0.592340278 | Low-middle SDI |  |
| Micronesia (Federated States of) | 0.588012508 | Low-middle SDI |  |
| Congo | 0.586908906 | Low-middle SDI |  |
| Eswatini | 0.586216849 | Low-middle SDI |  |
| Tuvalu | 0.578627145 | Low-middle SDI |  |
| India | 0.5777383 | Low-middle SDI |  |
| Marshall Islands | 0.573524783 | Low-middle SDI |  |
| Democratic People's Republic of Korea | 0.56945513 | Low-middle SDI |  |
| El Salvador | 0.565569678 | Low-middle SDI |  |
| Ghana | 0.563348184 | Low-middle SDI |  |
| Morocco | 0.561680434 | Low-middle SDI |  |
| Sudan | 0.542748299 | Low-middle SDI |  |
| Guatemala | 0.540099007 | Low-middle SDI |  |
| Tajikistan | 0.536613238 | Low-middle SDI |  |
| Cabo Verde | 0.533600978 | Low-middle SDI |  |
| Myanmar | 0.528492169 | Low-middle SDI |  |
| Kiribati | 0.525957502 | Low-middle SDI |  |
| Kenya | 0.524783146 | Low-middle SDI |  |
| Nicaragua | 0.52364671 | Low-middle SDI |  |
| Honduras | 0.513585699 | Low-middle SDI |  |
| Lesotho | 0.51157061 | Low-middle SDI |  |
| Zambia | 0.510230369 | Low-middle SDI |  |
| Pakistan | 0.504275856 | Low-middle SDI |  |
| Nigeria | 0.503698612 | Low-middle SDI |  |
| Sao Tome and Principe | 0.503305577 | Low-middle SDI |  |
| Mauritania | 0.495266784 | Low-middle SDI |  |
| Bangladesh | 0.493106236 | Low-middle SDI |  |
| Lao People's Democratic Republic | 0.489280726 | Low-middle SDI |  |
| Djibouti | 0.489200321 | Low-middle SDI |  |
| Angola | 0.482946052 | Low-middle SDI |  |
| Cameroon | 0.480364523 | Low-middle SDI |  |
| Comoros | 0.476955685 | Low-middle SDI |  |
| Bhutan | 0.476724988 | Low-middle SDI |  |
| Zimbabwe | 0.475577138 | Low-middle SDI |  |
| Cambodia | 0.473999694 | Low-middle SDI |  |
| Vanuatu | 0.472796337 | Low-middle SDI |  |
| Yemen | 0.453539967 | Low SDI |  |
| Timor-Leste | 0.450689053 | Low SDI |  |
| Haiti | 0.448751017 | Low SDI |  |
| United Republic of Tanzania | 0.448565569 | Low SDI |  |
| Rwanda | 0.436140248 | Low SDI |  |
| Nepal | 0.433952916 | Low SDI |  |
| Solomon Islands | 0.429541799 | Low SDI |  |
| Uganda | 0.426553554 | Low SDI |  |
| Cmete d'Ivoire | 0.424540566 | Low SDI |  |
| Papua New Guinea | 0.418098053 | Low SDI |  |
| Gambia | 0.410077462 | Low SDI |  |
| Togo | 0.410016394 | Low SDI |  |
| Senegal | 0.409005254 | Low SDI |  |
| Eritrea | 0.404572056 | Low SDI |  |
| Madagascar | 0.401385119 | Low SDI |  |
| Democratic Republic of the Congo | 0.390178166 | Low SDI |  |
| Malawi | 0.381985594 | Low SDI |  |
| Benin | 0.37452237 | Low SDI |  |
| Ethiopia | 0.360727644 | Low SDI |  |
| Sierra Leone | 0.35900867 | Low SDI |  |
| Guinea-Bissau | 0.353448423 | Low SDI |  |
| Liberia | 0.353229409 | Low SDI |  |
| Guinea | 0.336555329 | Low SDI |  |
| Afghanistan | 0.335068107 | Low SDI |  |
| Mozambique | 0.327475463 | Low SDI |  |
| Central African Republic | 0.311026626 | Low SDI |  |
| Burundi | 0.291288817 | Low SDI |  |
| Burkina Faso | 0.284470947 | Low SDI |  |
| South Sudan | 0.278377554 | Low SDI |  |
| Mali | 0.271175692 | Low SDI |  |
| Chad | 0.243516859 | Low SDI |  |
| Niger | 0.170310328 | Low SDI |  |
| Somalia | 0.077433678 | Low SDI |  |

## Table B. GBD world standard population for both sexes and all age groups in 1990-2021

|  | | **Population in 1990, No.(95% UI) * 10^5^** | | | | | | | | | | |
| --- | --- | --- | --- | --- | --- | --- | --- | --- | --- | --- | --- | --- |
|  |  | Both | | | | | | Female | | | Male | |
| Age Groups | | 53336.23 (52310.36-54446.5) | | | | | | 26478.8 (25978.5-27019.44) | | | 26857.43 (26332.35-27430.89) | |
| Children, adolescents, and young adults | |  | | | | | |  | | |  | |
| 0-6 days | | 25.39 (24.91-25.89) | | | | | | 12.24 (12.01-12.47) | | | 13.15 (12.9-13.42) | |
| 7-27 days | | 75.01 (73.59-76.48) | | | | | | 36.21 (35.54-36.92) | | | 38.79 (38.05-39.57) | |
| 1-5 months | | 545.96 (535.68-556.69) | | | | | | 263.86 (258.96-268.99) | | | 282.09 (276.72-287.78) | |
| 6-11 months | | 631.12 (619.21-643.57) | | | | | | 305.24 (299.56-311.2) | | | 325.88 (319.65-332.45) | |
| 2-4 years | | 3675.72 (3604.92-3750.37) | | | | | | 1782.73 (1748.9-1818.62) | | | 1893 (1855.96-1931.75) | |
| 5-9 years | | 5835.31 (5721.49-5954.61) | | | | | | 2838.28 (2783.96-2895.79) | | | 2997.02 (2937.53-3059.3) | |
| 10-14 years | | 5356.83 (5254.1-5465.24) | | | | | | 2614.3 (2564.98-2666.67) | | | 2742.52 (2688.84-2798.78) | |
| 15-19 years | | 5194.23 (5088.63-5307.25) | | | | | | 2555.39 (2504.3-2610.24) | | | 2638.85 (2584.31-2697.12) | |
| 20-24 years | | 4920.88 (4815.25-5034.81) | | | | | | 2441.35 (2389.98-2496.91) | | | 2479.53 (2425.59-2537.88) | |
| 25-29 years | | 4426.2 (4336.2-4524.4) | | | | | | 2201 (2157.22-2248.79) | | | 2225.2 (2179.25-2275.61) | |
| 30-34 years | | 3854.22 (3778.87-3935.51) | | | | | | 1901.09 (1864.64-1940.18) | | | 1953.13 (1914.47-1995.19) | |
| 35-39 years | | 3522.44 (3449.23-3603.67) | | | | | | 1734.53 (1699.18-1773.56) | | | 1787.91 (1750.02-1829.75) | |
|  | **Population in 2000, No.(95% UI) * 10^5^** | | | | | | | | | | | |
|  | Both | | | | Female | | | | | | | Male |
| Age Groups | 60987.78 (59764.44-62226.2) | | | | 30265.89 (29668.88-30870.55) | | | | | | | 30721.89 (30094.63-31356.54) |
| Children, adolescents, and young adults |  | | | |  | | | | | | |  |
| 0-6 days | 24.77 (24.3-25.26) | | | | 11.92 (11.69-12.15) | | | | | | | 12.85 (12.61-13.11) |
| 7-27 days | 73.28 (71.91-74.73) | | | | 35.32 (34.66-36.01) | | | | | | | 37.96 (37.25-38.71) |
| 1-5 months | 534.78 (524.78-545.37) | | | | 257.94 (253.11-262.99) | | | | | | | 276.85 (271.62-282.32) |
| 6-11 months | 619.63 (608.02-631.94) | | | | 298.98 (293.4-304.85) | | | | | | | 320.65 (314.57-327) |
| 2-4 years | 3612.3 (3542.4-3684.01) | | | | 1743.84 (1710.67-1777.87) | | | | | | | 1868.46 (1831.78-1906.25) |
| 5-9 years | 6016.98 (5896.44-6138.73) | | | | 2901.7 (2845.13-2959.31) | | | | | | | 3115.28 (3051.68-3178.66) |
| 10-14 years | 6235.89 (6106.48-6367.77) | | | | 3014.46 (2952.49-3077.09) | | | | | | | 3221.43 (3153.86-3291.32) |
| 15-19 years | 5687.97 (5571.7-5804.76) | | | | 2785.82 (2730.11-2841.57) | | | | | | | 2902.15 (2842.3-2962.83) |
| 20-24 years | 5110.25 (5007.66-5212.07) | | | | 2550.45 (2499.39-2600.86) | | | | | | | 2559.81 (2508.25-2611.34) |
| 25-29 years | 4987.47 (4881.64-5098.14) | | | | 2488.42 (2436.09-2542.91) | | | | | | | 2499.05 (2445.54-2555.11) |
| 30-34 years | 4791.01 (4682.5-4903.67) | | | | 2377.88 (2324.29-2433.18) | | | | | | | 2413.12 (2357.49-2470.85) |
| 35-39 years | 4343.57 (4249.39-4440.97) | | | | 2148.83 (2102.79-2196.83) | | | | | | | 2194.74 (2146.31-2244.75) |
|  | | | | **Population in 2010, No.(95% UI) * 10^5^** | | | | | | | | |
|  |  |  |  | Both | | Female | | | Male | | | |
| Age Groups | | | | 69507.56 (68148.25-70896.65) | | 34580.71 (33914.03-35254.42) | | | 34926.85 (34237.99-35632.43) | | | |
| Children, adolescents, and young adults | | | |  | |  | | |  | | | |
| 0-6 days | | | | 26.3 (25.81-26.79) | | 12.68 (12.45-12.92) | | | 13.62 (13.36-13.88) | | | |
| 7-27 days | | | | 78.03 (76.56-79.48) | | 37.68 (36.97-38.37) | | | 40.35 (39.58-41.11) | | | |
| 1-5 months | | | | 572.16 (561.33-582.77) | | 276.36 (271.17-281.43) | | | 295.8 (290.17-301.31) | | | |
| 6-11 months | | | | 667.33 (654.65-679.61) | | 322.34 (316.26-328.25) | | | 344.99 (338.37-351.45) | | | |
| 2-4 years | | | | 3882.23 (3806.84-3957.1) | | 1876.14 (1839.83-1911.59) | | | 2006.09 (1966.82-2045.18) | | | |
| 5-9 years | | | | 6141.53 (6018.55-6263.59) | | 2965.14 (2906.8-3023.19) | | | 3176.39 (3112.29-3239.98) | | | |
| 10-14 years | | | | 6039.12 (5917.95-6161.38) | | 2915.76 (2858.49-2974.11) | | | 3123.36 (3059.74-3187.77) | | | |
| 15-19 years | | | | 6099.5 (5977.43-6224.97) | | 2974.38 (2915.44-3034.13) | | | 3125.12 (3061.29-3190.6) | | | |
| 20-24 years | | | | 6146.21 (6022.13-6273.17) | | 3048.36 (2987.62-3111.2) | | | 3097.85 (3034.98-3162.67) | | | |
| 25-29 years | | | | 5544.62 (5436.51-5654.42) | | 2768.69 (2715.23-2822.92) | | | 2775.93 (2721.29-2831.66) | | | |
| 30-34 years | | | | 5082.61 (4984.71-5185) | | 2530.07 (2481.61-2580.48) | | | 2552.53 (2502.91-2604.54) | | | |
| 35-39 years | | | | 4947.52 (4847.49-5053.76) | | 2453.7 (2404.36-2505.33) | | | 2493.82 (2442.76-2548.06) | | | |
|  | | | **Population in 2021, No.(95% UI) * 10^5^** | | | | | | | | | |
|  |  |  | Both | | | | Female | | | Male | | |
| Age Groups | | | 78913.53 (76667.34-81312.25) | | | | 39319.62 (38225.04-40494.01) | | | 39593.91 (38438.46-40823.98) | | |
| Children, adolescents, and young adults | | |  | | | |  | | |  | | |
| 0-6 days | | | 24.51 (23.86-25.2) | | | | 11.83 (11.52-12.16) | | | 12.68 (12.34-13.03) | | |
| 7-27 days | | | 72.94 (71-74.97) | | | | 35.25 (34.32-36.22) | | | 37.69 (36.69-38.75) | | |
| 1-5 months | | | 537.57 (523.36-552.51) | | | | 259.84 (252.97-266.97) | | | 277.73 (270.35-285.54) | | |
| 6-11 months | | | 631.92 (615.32-649.45) | | | | 305.43 (297.41-313.79) | | | 326.5 (317.75-335.66) | | |
| 2-4 years | | | 4030.66 (3920.98-4145.16) | | | | 1948.6 (1896.38-2002.83) | | | 2082.07 (2024.3-2141.36) | | |
| 5-9 years | | | 6870.54 (6674.93-7071.33) | | | | 3325.77 (3232.24-3421.7) | | | 3544.76 (3443.12-3649.56) | | |
| 10-14 years | | | 6666.36 (6465.69-6875.06) | | | | 3229.26 (3132.64-3328.34) | | | 3437.1 (3333.94-3545.44) | | |
| 15-19 years | | | 6239.8 (6046.17-6442.52) | | | | 3036.5 (2942.75-3133.51) | | | 3203.3 (3101.79-3308.9) | | |
| 20-24 years | | | 5971.58 (5787.49-6169.01) | | | | 2937.52 (2847.48-3033.23) | | | 3034.06 (2938.08-3134.57) | | |
| 25-29 years | | | 5883.43 (5704.87-6074.22) | | | | 2909.87 (2822.71-3003.08) | | | 2973.56 (2882.16-3071.09) | | |
| 30-34 years | | | 6044.8 (5861.95-6236.04) | | | | 2989.31 (2899.84-3082.92) | | | 3055.49 (2962.52-3153.39) | | |
| 35-39 years | | | 5608.66 (5442.52-5782.98) | | | | 2778.03 (2697.19-2862.91) | | | 2830.63 (2745.63-2919.71) | | |

## Table C. The age-standardized incidence rates per 100,000 populations of oral disorders, across different sexes, age groups, and SDI quintiles, in 1990-2021

| **Characteristic** | 1995 | 1996 | 1997 | 1998 | 1999 |
| --- | --- | --- | --- | --- | --- |
| **Caries of deciduous teeth** |  | | | | |
| Global | 66374.89 (40304.51 to 102758.08) | 66301.62 (40404.2 to 102432.99) | 66215.17 (40516.23 to 102083.52) | 66124.17 (40608.67 to 101800.64) | 66039.56 (40679.29 to 101470.9) |
| Sex |  |  |  |  |  |
| Female | 66097.62 (40220.93 to 102173.81) | 66008.71 (40365.1 to 101739.65) | 65875.11 (40458.86 to 101225.47) | 65722.73 (40532 to 100816.5) | 65580.89 (40543.91 to 100386.08) |
| Male | 66635.82 (40380.73 to 103392.4) | 66576.85 (40450.76 to 103248.28) | 66534.29 (40534.25 to 103133.74) | 66500.38 (40610.45 to 103017.26) | 66468.84 (40672.48 to 102820.52) |
| Age* |  |  |  |  |  |
| 2-4 years | 58792.76 (37824.35 to 83013.71) | 58707.35 (37968.31 to 82703.23) | 58495.38 (38028.48 to 82155.06) | 58227.03 (38010.24 to 81538.1) | 57978.95 (38032.03 to 80986.22) |
| 5-9 years | 120963.65 (83431.31 to 155747.54) | 120890.29 (83560.97 to 155457.02) | 120783.76 (83703.39 to 155175.78) | 120658.34 (83840.21 to 155011.15) | 120536.49 (83963.03 to 154632.8) |
| 10-14 years | 35378 (7926.11 to 99332.49) | 35359.67 (7930.35 to 98844.77) | 35390.25 (8014.62 to 98439.69) | 35444.65 (8109.32 to 98165.81) | 35498.74 (8150.25 to 98005.21) |
| Sociodemographic index |  |  |  |  |  |
| Low SDI | 67038.25 (40305.63 to 103190.34) | 66976.77 (40770.48 to 102424.77) | 66830.55 (41205.78 to 101503.63) | 66649.63 (41520.81 to 100603.74) | 66491.7 (41766.31 to 100002.06) |
| Low-middle SDI | 68347.18 (40378.28 to 107471.58) | 68332.37 (40493.75 to 107206.17) | 68336.98 (40640.68 to 107122.31) | 68349.11 (40765.05 to 107166.86) | 68355.45 (41048.84 to 107149.73) |
| Middle SDI | 66426.23 (40469.85 to 102485.85) | 66344.01 (40513.51 to 102146.3) | 66217.35 (40504.36 to 101751.05) | 66063.65 (40476.35 to 101329.05) | 65907.78 (40455.67 to 100925.6) |
| High-middle SDI | 68843.62 (41941.95 to 106366.75) | 68688.92 (41883.67 to 106185.87) | 68626.43 (41917.26 to 106125.01) | 68611.06 (42024.73 to 106137.89) | 68603.82 (42152.7 to 106069.56) |
| High SDI | 56578.06 (35048.14 to 86234.87) | 56451.89 (35163.47 to 85959.75) | 56239.14 (35172.7 to 85323.36) | 55999.62 (35100.73 to 84910.63) | 55782.68 (35045.31 to 84512.85) |
| **Caries of permanents teeth** |  | | | | |
| Global | 36701.01 (27467.4 to 46623.25) | 36654.31 (27452.35 to 46538.82) | 36552.29 (27382.35 to 46400.54) | 36434.89 (27290.96 to 46251.34) | 36344.02 (27212.03 to 46135.91) |
| Sex |  |  |  |  |  |
| Female | 36882.1 (27541.78 to 46886.62) | 36812.38 (27503.67 to 46763.33) | 36663.62 (27391.24 to 46577.58) | 36490.41 (27249.97 to 46366.88) | 36349.01 (27129.59 to 46208.13) |
| Male | 36528.44 (27396.55 to 46389.17) | 36504.35 (27403.25 to 46334.28) | 36448.11 (27369.78 to 46242) | 36385.39 (27323.62 to 46154.87) | 36344.09 (27287.61 to 46096.46) |
| Age* |  |  |  |  |  |
| 5-9 years | 17305.01 (8942.99 to 29930.95) | 17189.82 (8891.2 to 29677.2) | 17091.52 (8844.23 to 29414.99) | 17032.26 (8815.34 to 29209.87) | 17024.55 (8813.8 to 29106.97) |
| 10-14 years | 33647.89 (23595.33 to 44885.47) | 33421.24 (23444.98 to 44558.76) | 33131.85 (23248.19 to 44151.33) | 32849.05 (23052.53 to 43744.57) | 32633.98 (22898.39 to 43416.51) |
| 15-19 years | 41749.67 (32764.27 to 51188.55) | 41818.26 (32827.34 to 51293.05) | 41806.56 (32819.7 to 51309.79) | 41735.16 (32757.17 to 51255.27) | 41624.25 (32658.37 to 51148.08) |
| 20-24 years | 50157.8 (40699.66 to 59384.75) | 50276.97 (40835.3 to 59472.94) | 50364.65 (40922.02 to 59566.32) | 50443.68 (40998.73 to 59650.31) | 50525.28 (41053.72 to 59704.52) |
| 25-29 years | 44803.46 (34135.67 to 55228.82) | 44836.6 (34135.1 to 55279.55) | 44804.14 (34064.87 to 55278.74) | 44752.94 (33974.48 to 55276.37) | 44739.43 (33909.67 to 55329.79) |
| 30-34 years | 39273.91 (30248.4 to 46964.56) | 39049.61 (30102.19 to 46699.87) | 38811.82 (29924.16 to 46449.78) | 38630.1 (29761.25 to 46292.57) | 38539.82 (29676.46 to 46248.99) |
| 35-39 years | 34887.04 (26825.84 to 42467.12) | 34946.42 (26898.5 to 42557.8) | 34827.83 (26824.27 to 42463.44) | 34561.18 (26630.28 to 42196.56) | 34256.83 (26402.72 to 41874.92) |
| Sociodemographic index |  |  |  |  |  |
| Low SDI | 38334.76 (29464.38 to 48101.95) | 38298.24 (29475.89 to 48005.58) | 38208.93 (29444.62 to 47831.54) | 38100.61 (29395.28 to 47658.48) | 38006.14 (29344.57 to 47536.53) |
| Low-middle SDI | 39741.57 (29287.12 to 50921.38) | 39730.49 (29292.14 to 50894.4) | 39710.46 (29287.81 to 50852.3) | 39683.05 (29287.67 to 50813.61) | 39650.68 (29272.68 to 50764.62) |
| Middle SDI | 35002.98 (25496.56 to 45301.49) | 35016.01 (25513.03 to 45234.95) | 34964.18 (25480.41 to 45107.68) | 34891.26 (25438.48 to 44958.02) | 34848.29 (25411.61 to 44856.62) |
| High-middle SDI | 33819.29 (24907.64 to 43613.65) | 33639.87 (24776.03 to 43360.12) | 33365.73 (24558.92 to 42976.01) | 33072.31 (24321.23 to 42576.15) | 32835.54 (24115.71 to 42274.84) |
| High SDI | 39107.53 (30894.02 to 47864.39) | 38920.42 (30769.44 to 47651.03) | 38680.97 (30618.8 to 47354.17) | 38443.19 (30490.46 to 47037.73) | 38258 (30378.97 to 46793.98) |
| **Periodontal diseases** |  | | | | |
| Global | 1090.58 (625.78 to 1634.47) | 1092.18 (637.56 to 1626.04) | 1093.31 (646.26 to 1612.89) | 1094.18 (654.61 to 1602.52) | 1095.65 (662.42 to 1592.11) |
| Sex |  |  |  |  |  |
| Female | 1066.14 (631.31 to 1571.44) | 1068.04 (640.58 to 1563.47) | 1068.99 (648.32 to 1554.83) | 1069.51 (652.57 to 1546.85) | 1070.74 (655.28 to 1541.84) |
| Male | 1114.44 (607.07 to 1694.46) | 1115.78 (627.57 to 1678.22) | 1117.12 (644.41 to 1669.84) | 1118.39 (657.16 to 1657.38) | 1120.12 (665.93 to 1643.67) |
| Age* |  |  |  |  |  |
| 15-19 years | 427.45 (217.46 to 740.17) | 432.93 (225.04 to 742.55) | 437.02 (232.95 to 746.3) | 439.72 (238.59 to 747.31) | 441.41 (242.44 to 745.38) |
| 20-24 years | 894.8 (518.54 to 1342.16) | 909.14 (538.4 to 1354.7) | 923.83 (551.63 to 1364.46) | 938.06 (566.69 to 1370.92) | 950.78 (582.41 to 1370.6) |
| 25-29 years | 1207.16 (701.07 to 1809.98) | 1213.3 (712.13 to 1799.72) | 1220.67 (723.49 to 1795.04) | 1228.49 (736.57 to 1799.12) | 1237.93 (748.51 to 1803.72) |
| 30-34 years | 1406.87 (856.35 to 2047.11) | 1393.39 (860.01 to 2011.19) | 1383.28 (862.47 to 1980.36) | 1378.51 (865.86 to 1963.56) | 1378.15 (872.71 to 1949.51) |
| 35-39 years | 1650.51 (913.03 to 2416.52) | 1643.97 (930.22 to 2402.51) | 1631.12 (938.43 to 2352.21) | 1612.84 (942.47 to 2299.63) | 1594.27 (942.5 to 2254.57) |
| Sociodemographic index |  |  |  |  |  |
| Low SDI | 1731.31 (971.73 to 2521.27) | 1722.24 (976.81 to 2499.53) | 1703.8 (973.21 to 2465.48) | 1682.04 (964.3 to 2431.12) | 1663.64 (955.32 to 2402.8) |
| Low-middle SDI | 1508.42 (879.45 to 2189.36) | 1513.61 (895.71 to 2185.24) | 1520.1 (909.38 to 2182.16) | 1526.69 (920.37 to 2179.08) | 1532.5 (929.54 to 2178.71) |
| Middle SDI | 968.49 (533.92 to 1487.98) | 968.86 (545.99 to 1474.92) | 970 (556.15 to 1468.46) | 971.84 (562.74 to 1462.18) | 975.03 (568.86 to 1458.28) |
| High-middle SDI | 730.81 (395.91 to 1162.37) | 720.64 (400.08 to 1132.38) | 709.86 (403.1 to 1101.88) | 699.69 (403.74 to 1075.5) | 691.92 (405.67 to 1052.35) |
| High SDI | 856.83 (537.44 to 1245.18) | 855.49 (544.87 to 1229.16) | 853.94 (551.31 to 1219.32) | 851.5 (553.96 to 1209.18) | 847.6 (551.58 to 1207.59) |
| **Edentulism** |  |  |  |  |  |
| Global | 83.78 (45.9 to 135.5) | 83.39 (45.8 to 134.49) | 81.76 (45.05 to 131.4) | 79.59 (43.98 to 127.46) | 77.72 (43.04 to 124.22) |
| Sex |  |  |  |  |  |
| Female | 92 (50.5 to 148.42) | 91.59 (50.45 to 147.31) | 89.87 (49.69 to 144.14) | 87.54 (48.58 to 140.01) | 85.53 (47.6 to 136.46) |
| Male | 75.75 (41.33 to 122.91) | 75.35 (41.22 to 121.72) | 73.81 (40.5 to 118.85) | 71.76 (39.45 to 115.29) | 70.02 (38.48 to 112.31) |
| Age* |  |  |  |  |  |
| 20-24 years | 46.4 (24.8 to 72.55) | 46.64 (24.96 to 72.76) | 46.11 (24.75 to 71.74) | 45.24 (24.39 to 70.08) | 44.47 (24.1 to 68.76) |
| 25-29 years | 68.78 (37.79 to 107.17) | 68.69 (37.87 to 106.74) | 67.67 (37.44 to 104.54) | 66.34 (36.79 to 102.13) | 65.43 (36.34 to 100.59) |
| 30-34 years | 92.51 (51.9 to 151.59) | 90.66 (51.09 to 147.92) | 88.17 (49.91 to 143.25) | 85.87 (48.74 to 139.09) | 84.22 (47.84 to 136.28) |
| 35-39 years | 133.8 (72.56 to 221.67) | 133.92 (72.77 to 221.49) | 131.29 (71.52 to 216.71) | 126.82 (69.26 to 208.73) | 122.39 (66.98 to 200.91) |
| Sociodemographic index |  |  |  |  |  |
| Low SDI | 111.39 (61.12 to 176.93) | 110.59 (60.83 to 174.96) | 108.24 (59.66 to 170.71) | 105.44 (58.19 to 166.1) | 103.22 (57.02 to 162.47) |
| Low-middle SDI | 89.49 (47.7 to 146.05) | 87 (46.61 to 141.63) | 81.44 (43.82 to 132.31) | 74.91 (40.45 to 121.49) | 69.51 (37.61 to 112.55) |
| Middle SDI | 77.53 (42.21 to 125.76) | 76.97 (41.99 to 124.6) | 74.95 (41.11 to 121.01) | 72.3 (39.79 to 116.53) | 70.1 (38.62 to 112.8) |
| High-middle SDI | 73.99 (40.8 to 118.51) | 73.77 (40.64 to 118.09) | 73.76 (40.71 to 117.83) | 73.66 (40.77 to 117.42) | 73.44 (40.73 to 116.85) |
| High SDI | 82.41 (45.04 to 134.6) | 84.53 (46.38 to 137.76) | 86.73 (47.71 to 141.14) | 88.78 (48.9 to 144.36) | 90.51 (49.91 to 146.86) |
| **Characteristic** | 2000 | 2001 | 2002 | 2003 | 2004 |
| **Caries of deciduous teeth** |  |  |  |  |  |
| Global | 65975.7 (40734.38 to 101245.09) | 65762.61 (40778.95 to 100466.11) | 65318.62 (40652.61 to 99498.69) | 64793.23 (40427.99 to 98461.77) | 64338.22 (40334.7 to 97574.65) |
| Sex |  |  |  |  |  |
| Female | 65481.01 (40559.71 to 100016) | 65192.53 (40647.45 to 98829.25) | 64591.64 (40558.13 to 97609.12) | 63878.89 (40404.65 to 96363.7) | 63260.49 (40339.12 to 95187.33) |
| Male | 66438.08 (40733.34 to 102582.25) | 66294.83 (40815.84 to 102045.79) | 65996.77 (40740.63 to 101347.02) | 65645.77 (40601.97 to 100613.39) | 65342.95 (40483.17 to 99981.83) |
| Age* |  |  |  |  |  |
| 2-4 years | 57834.19 (38222.4 to 80617.03) | 57720.31 (38458.67 to 80075.98) | 57543.7 (38587.3 to 79489.39) | 57356.37 (38630.57 to 78888.97) | 57205.87 (38847.54 to 78316.05) |
| 5-9 years | 120450.4 (84096.07 to 154247.22) | 120090.67 (84062.03 to 153178.17) | 119301.55 (83631.82 to 151939) | 118350.89 (83037.14 to 150495.78) | 117524.09 (82847.45 to 149149.61) |
| 10-14 years | 35524.59 (8184.8 to 97987.26) | 35357.36 (8299.98 to 97077.49) | 34948.58 (8304.53 to 95775.96) | 34454.33 (8064.9 to 94369.13) | 34030.3 (7764 to 93270.27) |
| Sociodemographic index |  |  |  |  |  |
| Low SDI | 66403.78 (41976.53 to 99711.81) | 65897.54 (42335.29 to 98008.84) | 64735.25 (41904.01 to 95622.5) | 63351.01 (41301.57 to 93213.5) | 62192.29 (40745.28 to 91214.97) |
| Low-middle SDI | 68340.11 (41311.11 to 107050.17) | 68072.99 (41506.03 to 106065.16) | 67475.69 (41381.87 to 104610) | 66764.65 (40957.04 to 103541.47) | 66164.04 (40532.86 to 102552.18) |
| Middle SDI | 65785.14 (40462.97 to 100587.93) | 65619.65 (40567.41 to 100011.24) | 65362.19 (40630.79 to 99463.11) | 65089 (40673.84 to 98886.52) | 64857.51 (40798.2 to 98469.44) |
| High-middle SDI | 68567.19 (42219.78 to 105868.21) | 68513.61 (42221.9 to 105837.11) | 68491.99 (42128.45 to 105762.17) | 68521.59 (42111.88 to 105777.43) | 68563.4 (42185.09 to 105916.18) |
| High SDI | 55649.31 (35116.55 to 84345.7) | 55565.94 (35096.48 to 83887.97) | 55483.53 (35069.35 to 83458.91) | 55399.83 (35021.36 to 83107.46) | 55320.03 (34997.89 to 82810.06) |
| **Caries of permanents teeth** |  |  |  |  |  |
| Global | 36319.51 (27187.95 to 46110.77) | 36488.01 (27291.85 to 46327.25) | 36857.13 (27531.22 to 46809.2) | 37292.2 (27798.59 to 47396.52) | 37665.11 (28034.16 to 47916.24) |
| Sex |  |  |  |  |  |
| Female | 36292.93 (27075.83 to 46161.03) | 36464.61 (27191.87 to 46423.46) | 36868.46 (27472.07 to 46957.07) | 37352.97 (27808.11 to 47567.06) | 37772.5 (28090.19 to 48136.79) |
| Male | 36350.62 (27280.34 to 46103.84) | 36515.86 (27367.9 to 46289.6) | 36850.48 (27558.22 to 46744.54) | 37236.49 (27773.8 to 47291.27) | 37563.23 (27984.52 to 47731.85) |
| Age* |  |  |  |  |  |
| 5-9 years | 17063.92 (8839.68 to 29121.57) | 17151.64 (8877.95 to 29238.31) | 17276.13 (8939.13 to 29430.96) | 17403.2 (9002.46 to 29640.39) | 17497.55 (9047.76 to 29805.12) |
| 10-14 years | 32540.42 (22824.96 to 43236.27) | 32654.89 (22838.02 to 43501.1) | 32929.22 (22898.56 to 44230.56) | 33232.47 (22963.1 to 44888.33) | 33463.89 (23007.98 to 45448.77) |
| 15-19 years | 41510.32 (32555.97 to 51027.48) | 41592.72 (32556.11 to 51230.94) | 41998.69 (32756.09 to 51754.51) | 42610.56 (33036.62 to 52536.07) | 43234.36 (33461.59 to 53366.21) |
| 20-24 years | 50630.13 (41145.55 to 59832.96) | 50903.61 (41298.89 to 60164.55) | 51341.49 (41622.27 to 60680.77) | 51798.19 (41953.21 to 61255.22) | 52174.3 (42191.26 to 61734.41) |
| 25-29 years | 44824.41 (33922.98 to 55492.79) | 45190.22 (34242.27 to 55945.7) | 45806.14 (34730.46 to 56565.47) | 46437.67 (35199.11 to 57222.73) | 46917.1 (35561.29 to 57720.72) |
| 30-34 years | 38569.36 (29693.28 to 46332.59) | 38856.47 (29905.37 to 46628.21) | 39381.94 (30318.02 to 47225.26) | 39965.26 (30786.48 to 48037.78) | 40453.59 (31157.43 to 48668.54) |
| 35-39 years | 34014.02 (26250.12 to 41612.51) | 34005.98 (26280.49 to 41442.96) | 34280.65 (26496.87 to 41641.76) | 34708.22 (26792.01 to 42130.47) | 35111.14 (27029.19 to 42673.3) |
| Sociodemographic index |  |  |  |  |  |
| Low SDI | 37958.59 (29293.71 to 47500.02) | 37888.65 (29207.05 to 47423.84) | 37739.67 (29018.88 to 47270.18) | 37565.87 (28770.29 to 47112.96) | 37421.06 (28589.02 to 46996.94) |
| Low-middle SDI | 39615.94 (29243.98 to 50705.29) | 39435.91 (29065.95 to 50563.31) | 39055.95 (28698.19 to 50189.66) | 38610.34 (28247.43 to 49726.58) | 38231.46 (27858.74 to 49316.25) |
| Middle SDI | 34880.61 (25435.31 to 44882.38) | 35269.22 (25741.7 to 45413.92) | 36073.66 (26321.7 to 46449.46) | 37011.05 (26983 to 47660.09) | 37811.65 (27570.75 to 48687.81) |
| High-middle SDI | 32727.69 (24001.58 to 42200.81) | 33129.3 (24280.29 to 42643.48) | 34126.77 (25003.68 to 43888.08) | 35351.96 (25889.08 to 45535.95) | 36433.56 (26666.31 to 46874.35) |
| High SDI | 38175.43 (30307.22 to 46690.74) | 38220.18 (30337.27 to 46715.18) | 38349.16 (30453.49 to 46822.45) | 38513.76 (30610.16 to 46991.58) | 38667.02 (30723.52 to 47162.33) |
| **Periodontal diseases** |  |  |  |  |  |
| Global | 1098.82 (670.87 to 1585.52) | 1103.32 (676.63 to 1583.38) | 1108.09 (680.92 to 1587.71) | 1112.69 (683.6 to 1592.5) | 1116.7 (686.9 to 1596.5) |
| Sex |  |  |  |  |  |
| Female | 1074.22 (659.16 to 1538.6) | 1077.66 (664.34 to 1537.54) | 1078.79 (666.22 to 1539.26) | 1078.79 (668.03 to 1536.96) | 1078.85 (671.05 to 1538.08) |
| Male | 1123.03 (677.52 to 1631.73) | 1128.59 (686.41 to 1631.25) | 1136.96 (694.05 to 1639.87) | 1146.11 (700.92 to 1645.2) | 1154.01 (707.16 to 1652.66) |
| Age* |  |  |  |  |  |
| 15-19 years | 442.51 (245.87 to 741.98) | 439.91 (247.74 to 731.13) | 432.91 (244.98 to 715.14) | 425.19 (241.25 to 699.71) | 419.95 (239.76 to 689.49) |
| 20-24 years | 962.12 (593.36 to 1376.78) | 965.86 (602.09 to 1371.04) | 958.9 (600.01 to 1352.83) | 946.69 (594.81 to 1336.45) | 935.41 (589.77 to 1315.93) |
| 25-29 years | 1250.09 (758.65 to 1813.69) | 1262.06 (766.77 to 1824.85) | 1269.99 (769.42 to 1832.11) | 1274.23 (768.31 to 1835.04) | 1276.48 (766.59 to 1836.84) |
| 30-34 years | 1381.49 (881.45 to 1936.53) | 1390.43 (887.77 to 1939.88) | 1405.15 (899.63 to 1969.77) | 1421.28 (909.49 to 2005.16) | 1435.79 (919.97 to 2018.85) |
| 35-39 years | 1580.45 (951.98 to 2218) | 1581.38 (955.81 to 2209.88) | 1599.65 (969.65 to 2233.95) | 1626.47 (985.62 to 2256.74) | 1649.88 (1002.18 to 2298.06) |
| Sociodemographic index |  |  |  |  |  |
| Low SDI | 1655.39 (953.73 to 2386.97) | 1642.47 (959.33 to 2357.74) | 1611.75 (950.47 to 2305.15) | 1574.18 (931.88 to 2244.9) | 1542.15 (913.29 to 2194.89) |
| Low-middle SDI | 1536.71 (939.32 to 2177.79) | 1533.93 (943.82 to 2167.45) | 1522.88 (943.19 to 2145.24) | 1509.13 (939.03 to 2119.78) | 1498.31 (932.93 to 2097.71) |
| Middle SDI | 980.23 (575.79 to 1457.52) | 990.91 (587.53 to 1467.43) | 1007.49 (600.58 to 1485.97) | 1025.48 (612.1 to 1507.35) | 1040.6 (622.61 to 1524.62) |
| High-middle SDI | 688.58 (408.38 to 1036.87) | 703.22 (419.72 to 1056.35) | 738.85 (444.13 to 1108.46) | 781.56 (469.47 to 1173.9) | 817.35 (493.95 to 1227.27) |
| High SDI | 841.86 (544.26 to 1207.78) | 821.43 (531.11 to 1186.26) | 783.29 (502.62 to 1141.6) | 739.71 (467.22 to 1087.58) | 702.63 (437.93 to 1048.24) |
| **Edentulism** |  |  |  |  |  |
| Global | 76.96 (42.67 to 122.92) | 78.23 (44.03 to 124.55) | 81.2 (46.46 to 128.52) | 84.92 (49.38 to 133.32) | 88.32 (52 to 137.63) |
| Sex |  |  |  |  |  |
| Female | 84.72 (47.24 to 135.02) | 86.19 (48.87 to 136.74) | 89.66 (51.72 to 141.07) | 93.98 (55.17 to 146.3) | 97.92 (58.65 to 150.59) |
| Male | 69.31 (38.05 to 111.16) | 70.35 (39.1 to 112.41) | 72.83 (41.05 to 115.85) | 75.96 (43.33 to 120.27) | 78.83 (45.33 to 124.44) |
| Age* |  |  |  |  |  |
| 20-24 years | 44.39 (24.13 to 68.54) | 46.24 (25.96 to 70.99) | 50.07 (29.21 to 75.77) | 54.71 (32.78 to 81.39) | 58.93 (36.1 to 86.6) |
| 25-29 years | 65.74 (36.57 to 100.97) | 68.03 (38.65 to 104.26) | 71.92 (41.55 to 109.87) | 76.47 (44.91 to 116.06) | 80.58 (47.97 to 121.81) |
| 30-34 years | 83.87 (47.63 to 135.74) | 85.28 (48.9 to 137.77) | 87.98 (51 to 141.69) | 91.07 (53.52 to 146.09) | 93.8 (55.71 to 149.55) |
| 35-39 years | 119.25 (65.33 to 195.7) | 118.54 (65.42 to 194.15) | 119.8 (66.75 to 195.39) | 122.23 (68.86 to 198.15) | 124.64 (70.68 to 200.73) |
| Sociodemographic index |  |  |  |  |  |
| Low SDI | 102.53 (56.71 to 161.27) | 103.1 (57.28 to 161.77) | 104.06 (58.25 to 162.7) | 105.35 (59.43 to 164.24) | 106.75 (60.66 to 166.15) |
| Low-middle SDI | 67.27 (36.45 to 108.83) | 68.74 (37.88 to 110.63) | 72.3 (40.62 to 115.34) | 76.76 (43.94 to 121.16) | 80.94 (47.05 to 126.57) |
| Middle SDI | 69.34 (38.21 to 111.54) | 71.59 (40.35 to 114.14) | 76.64 (44.03 to 120.94) | 82.86 (48.28 to 129.02) | 88.46 (52.31 to 136.09) |
| High-middle SDI | 73.14 (40.56 to 116.26) | 73.07 (41.04 to 115.59) | 73.54 (42.12 to 115.56) | 74.41 (43.41 to 115.94) | 75.41 (44.65 to 116.48) |
| High SDI | 91.46 (50.38 to 148.08) | 92.76 (51.31 to 149.66) | 94.99 (53.03 to 151.97) | 97.43 (54.87 to 154.26) | 99.16 (56.33 to 155.87) |
| **Characteristic** | 2005 | 2006 | 2007 | 2008 | 2009 |
| **Caries of deciduous teeth** |  |  |  |  |  |
| Global | 64114.1 (40456.93 to 96962.75) | 63940.84 (40572.25 to 96409.67) | 63596.86 (40580.5 to 95670.36) | 63198.93 (40448.18 to 94957.75) | 62873.75 (40277.13 to 94369.05) |
| Sex |  |  |  |  |  |
| Female | 62954.15 (40243.47 to 94444.62) | 62839.41 (40256.74 to 94156.56) | 62671.41 (40206.34 to 93843.15) | 62496.13 (40153.3 to 93588.45) | 62366.26 (40113.81 to 93387.79) |
| Male | 65195.49 (40520.6 to 99524.2) | 64967.76 (40817.2 to 98573.26) | 64459.88 (40807.24 to 97385.85) | 63854.53 (40601.3 to 96211.89) | 63347.36 (40369.53 to 95272.55) |
| Age* |  |  |  |  |  |
| 2-4 years | 57132.84 (39241.63 to 77929.08) | 57156.26 (39493.08 to 77666.42) | 57242.84 (39783.19 to 77421.92) | 57358.32 (40059.9 to 77209.41) | 57470.89 (40271.66 to 77043.45) |
| 5-9 years | 117128.16 (83104.26 to 148247.6) | 116956.15 (83262.34 to 147880.16) | 116650.06 (83294.79 to 147380.52) | 116302.09 (83090.28 to 146815.71) | 116017.77 (82924.71 to 146284.6) |
| 10-14 years | 33839.73 (7646.63 to 92503.13) | 33550.26 (7738.71 to 91343.27) | 32860.03 (7577.61 to 89736.35) | 32005.84 (7153.6 to 88166.67) | 31246.57 (6648.32 to 86952.77) |
| Sociodemographic index |  |  |  |  |  |
| Low SDI | 61692.56 (40604.73 to 90246.66) | 61391.4 (40589.72 to 89478.88) | 60714.41 (40073.82 to 88434.57) | 59911.34 (39479.65 to 87366.78) | 59234.83 (38958.75 to 86524.01) |
| Low-middle SDI | 65892.8 (40478.32 to 102021.19) | 65676.17 (40709.04 to 101159.68) | 65204.1 (40709.08 to 99964.52) | 64634.38 (40608.89 to 98772.31) | 64133.94 (40413.35 to 97832.25) |
| Middle SDI | 64726.7 (40935.02 to 98060.8) | 64667.72 (41042.84 to 97675.14) | 64612.97 (41155.33 to 97368.37) | 64582.96 (41236.79 to 97153.57) | 64600.07 (41344.99 to 97091.75) |
| High-middle SDI | 68578.65 (42312.86 to 105943.34) | 68524.89 (42294.27 to 105625.09) | 68407.68 (42240.7 to 105192.27) | 68297.04 (42196.05 to 104797.97) | 68267.03 (42222.52 to 104535.93) |
| High SDI | 55268.33 (34894.52 to 82647.58) | 55099.07 (34707.99 to 82338.04) | 54733.66 (34282.06 to 81927.97) | 54319 (33865.03 to 81552.35) | 54007.56 (33558.7 to 81329.35) |
| **Caries of permanents teeth** |  |  |  |  |  |
| Global | 37860.75 (28160.88 to 48187.61) | 37932.6 (28247.25 to 48232.68) | 38002.98 (28337.24 to 48274.24) | 38070.32 (28431.9 to 48326.23) | 38132.02 (28510.59 to 48374.78) |
| Sex |  |  |  |  |  |
| Female | 37995.21 (28237.48 to 48455.25) | 38079.02 (28336.52 to 48502.91) | 38159 (28425.07 to 48547.55) | 38231.33 (28510.95 to 48585.21) | 38291.9 (28582.25 to 48629.54) |
| Male | 37732.21 (28080.81 to 47964.4) | 37792.7 (28157 to 48013.87) | 37854.18 (28253 to 48051.87) | 37917.11 (28341.08 to 48103.41) | 37980.17 (28432.57 to 48157.49) |
| Age* |  |  |  |  |  |
| 5-9 years | 17522.89 (9053.4 to 29859.47) | 17422.06 (9022.71 to 29710.69) | 17208.59 (8904.55 to 29433.47) | 16955.15 (8754.08 to 29130.02) | 16731.23 (8624.07 to 28866.76) |
| 10-14 years | 33571.76 (23076.23 to 45636.89) | 33614.61 (23110.76 to 45637.04) | 33661.3 (23174.83 to 45655.65) | 33717.57 (23228.71 to 45698.01) | 33783.84 (23278.29 to 45759.51) |
| 15-19 years | 43623.52 (33716.17 to 53904.84) | 43814.19 (33902.94 to 54109.25) | 43998.09 (34088.67 to 54294.93) | 44159.63 (34253.26 to 54453.88) | 44288.11 (34366.58 to 54589.26) |
| 20-24 years | 52378.01 (42345.97 to 62002.61) | 52483.96 (42500.45 to 62027.71) | 52625.66 (42689.08 to 62048.59) | 52774.91 (42933.18 to 62134.97) | 52907.95 (43100.94 to 62208.97) |
| 25-29 years | 47142.72 (35737.04 to 58042.35) | 47245.9 (35854.03 to 58104.85) | 47385.46 (36019.38 to 58200.44) | 47532.41 (36194.08 to 58319.39) | 47651.8 (36350.02 to 58432.76) |
| 30-34 years | 40705.81 (31309.85 to 49007.09) | 40824.02 (31366.44 to 49140.75) | 40985.03 (31470.93 to 49316.9) | 41163.46 (31611.6 to 49511.66) | 41327.06 (31767 to 49646.64) |
| 35-39 years | 35325.07 (27134.91 to 42912.5) | 35411.7 (27253.35 to 43005.94) | 35521.31 (27343.61 to 43172.96) | 35641.4 (27444.81 to 43343.73) | 35765.19 (27545.04 to 43516.98) |
| Sociodemographic index |  |  |  |  |  |
| Low SDI | 37356.58 (28490.75 to 46971.4) | 37369.33 (28477.75 to 46969.44) | 37411.23 (28495.82 to 47029.97) | 37465.2 (28532.91 to 47128.41) | 37515.34 (28554.65 to 47251.67) |
| Low-middle SDI | 38050.38 (27661.55 to 49137.97) | 38023.46 (27693.99 to 49032.47) | 38017.97 (27737.99 to 48978.59) | 38030.59 (27797.2 to 48964.68) | 38057.64 (27874.67 to 48978.7) |
| Middle SDI | 38226.57 (27877.49 to 49208.71) | 38357.38 (27990.07 to 49301.6) | 38465.14 (28135.48 to 49365.83) | 38556.16 (28259.97 to 49400.35) | 38634.9 (28367.4 to 49454.31) |
| High-middle SDI | 37004.02 (27079.8 to 47587.33) | 37203.3 (27215.66 to 47797.21) | 37403.19 (27328.52 to 48038.05) | 37587.74 (27430.96 to 48208.34) | 37738.57 (27531.77 to 48352.65) |
| High SDI | 38756.1 (30794.37 to 47278.86) | 38787.45 (30797.18 to 47309.63) | 38811.02 (30820.35 to 47351.48) | 38834.47 (30812.24 to 47422.23) | 38867.65 (30802.3 to 47551.46) |
| **Periodontal diseases** |  |  |  |  |  |
| Global | 1120.46 (693.04 to 1598.03) | 1116.55 (698.97 to 1588.01) | 1102.5 (695.12 to 1560.38) | 1085.69 (689.12 to 1535.62) | 1073.22 (684 to 1515.86) |
| Sex |  |  |  |  |  |
| Female | 1080.98 (673.07 to 1544.38) | 1079.22 (676.71 to 1533.1) | 1070.3 (676.18 to 1515.17) | 1059.58 (673.21 to 1497.4) | 1052.2 (671.23 to 1482.44) |
| Male | 1159.39 (711.81 to 1658.72) | 1153.32 (716.32 to 1639.12) | 1134.19 (716.23 to 1607.48) | 1111.37 (704.02 to 1571.48) | 1093.89 (695.56 to 1547.12) |
| Age* |  |  |  |  |  |
| 15-19 years | 420.16 (241.63 to 687.81) | 421.36 (248.17 to 677.16) | 418.83 (253.54 to 652.75) | 415.73 (255.54 to 642.83) | 415.29 (257.22 to 635.87) |
| 20-24 years | 931.05 (589.09 to 1305.83) | 923.84 (586.45 to 1287.11) | 905.89 (580.13 to 1256.71) | 885.22 (570.57 to 1222.5) | 869.94 (561.84 to 1193.92) |
| 25-29 years | 1281.35 (767.37 to 1832.86) | 1282.38 (777.83 to 1832.1) | 1273.4 (770.32 to 1814.08) | 1259.86 (765.17 to 1790) | 1247.3 (757.54 to 1778.4) |
| 30-34 years | 1445.87 (929.92 to 2025.49) | 1444.87 (936.85 to 2024.3) | 1433.12 (930.26 to 1993.92) | 1419 (926.25 to 1980.28) | 1409.18 (925.77 to 1958.5) |
| 35-39 years | 1659.23 (1023.18 to 2317.43) | 1644.23 (1032.05 to 2298.35) | 1612.53 (1026.82 to 2262.2) | 1576.99 (1012.11 to 2217.54) | 1550.42 (1000.64 to 2185.41) |
| Sociodemographic index |  |  |  |  |  |
| Low SDI | 1526.83 (905.55 to 2169.22) | 1498.12 (921.63 to 2108.52) | 1435.25 (910.08 to 2001.2) | 1361.68 (870.11 to 1905.24) | 1300.54 (828.27 to 1819.62) |
| Low-middle SDI | 1495.98 (932.23 to 2088.25) | 1497.79 (946.58 to 2075.02) | 1496.84 (959.76 to 2063.27) | 1495.17 (968.84 to 2048.37) | 1494.83 (972.43 to 2035.36) |
| Middle SDI | 1049.75 (631.6 to 1530.34) | 1047.17 (634.46 to 1517.83) | 1034.2 (628.62 to 1491.78) | 1018.44 (622.51 to 1464.73) | 1006.86 (619.65 to 1442.46) |
| High-middle SDI | 833.07 (505.62 to 1263.7) | 820.8 (497.1 to 1249.08) | 790.31 (478.47 to 1209.03) | 754.72 (455.09 to 1167.65) | 726.1 (435.33 to 1135.08) |
| High SDI | 683.73 (423.09 to 1029.54) | 684.95 (422.9 to 1036.54) | 697.16 (433.73 to 1059.71) | 713.73 (443.91 to 1085.66) | 727.9 (446.19 to 1116.59) |
| **Edentulism** |  |  |  |  |  |
| Global | 90.39 (53.61 to 140.09) | 92.42 (55.17 to 142.37) | 95.78 (56.92 to 147.26) | 99.56 (58.72 to 152.63) | 102.77 (60.41 to 157.86) |
| Sex |  |  |  |  |  |
| Female | 100.3 (60.75 to 152.69) | 101.9 (61.71 to 154.45) | 104.04 (62.83 to 157.37) | 106.31 (63.85 to 160.73) | 108.29 (64.63 to 164.09) |
| Male | 80.59 (46.43 to 127.08) | 83.04 (48.33 to 130.3) | 87.62 (51.06 to 136.79) | 92.89 (53.79 to 144.62) | 97.33 (56.04 to 151.58) |
| Age* |  |  |  |  |  |
| 20-24 years | 61.52 (38.04 to 89.96) | 64.2 (39.56 to 94.13) | 68.66 (41.76 to 101.54) | 73.62 (44.08 to 109.4) | 77.86 (46.22 to 116.87) |
| 25-29 years | 83.22 (49.87 to 125.3) | 86.52 (52.05 to 129.04) | 92.18 (55.17 to 136.67) | 98.53 (58.32 to 144.92) | 103.7 (61.83 to 152.96) |
| 30-34 years | 95.44 (57.07 to 151.45) | 97.17 (58.67 to 153.26) | 100.15 (60.56 to 157.64) | 103.52 (62.52 to 162.39) | 106.36 (63.51 to 166.27) |
| 35-39 years | 125.94 (71.86 to 201.62) | 126.12 (72.7 to 200.62) | 126.1 (72.3 to 200.12) | 126.1 (71.81 to 200.13) | 126.35 (71.71 to 201.15) |
| Sociodemographic index |  |  |  |  |  |
| Low SDI | 108.13 (61.92 to 168.34) | 112.47 (64 to 175.23) | 120.77 (68.2 to 188.56) | 130.1 (73.09 to 203.55) | 137.59 (77.09 to 216.08) |
| Low-middle SDI | 83.69 (48.94 to 130.19) | 88.96 (51.74 to 138.17) | 99.11 (57.07 to 154.6) | 110.62 (62.58 to 173.98) | 120.06 (66.97 to 190.38) |
| Middle SDI | 91.66 (54.97 to 139.58) | 93.15 (56.72 to 140.68) | 94.8 (58.24 to 142.44) | 96.44 (59.46 to 144.91) | 97.89 (60.54 to 147.5) |
| High-middle SDI | 76.27 (45.45 to 117.18) | 77.01 (46.32 to 117.95) | 77.81 (46.99 to 119.15) | 78.63 (47.78 to 120.48) | 79.44 (48.38 to 121.69) |
| High SDI | 99.5 (56.98 to 155.85) | 97.07 (55.87 to 151.62) | 92.41 (53.42 to 144.19) | 87.25 (50.53 to 136.35) | 83.11 (48.23 to 129.88) |
| **Characteristic** | 2010 | 2011 | 2012 | 2013 | 2014 |
| **Caries of deciduous teeth** |  |  |  |  |  |
| Global | 62753.54 (40252.72 to 94093.04) | 62777.29 (40391.64 to 93965.02) | 62782.94 (40436.09 to 93817.02) | 62787.5 (40476.76 to 93664.28) | 62809.6 (40535.82 to 93545.28) |
| Sex |  |  |  |  |  |
| Female | 62334.49 (40168.37 to 93295.62) | 62379.65 (40208.61 to 93167.04) | 62428.53 (40231.48 to 93039.56) | 62484.34 (40284.97 to 92917.08) | 62551.7 (40326.62 to 92912.67) |
| Male | 63144.8 (40344.58 to 94738.23) | 63148.74 (40488.81 to 94603.53) | 63114.15 (40575.1 to 94442.42) | 63070.92 (40686.49 to 94287.33) | 63050.83 (40749.45 to 94166.73) |
| Age* |  |  |  |  |  |
| 2-4 years | 57539.54 (40337.85 to 76975.19) | 57707.61 (40523.4 to 77177.72) | 58073.62 (40836.76 to 77618.86) | 58513.34 (41148.92 to 78160.24) | 58901.98 (41505.72 to 78610.79) |
| 5-9 years | 115912.83 (82933.77 to 145915.43) | 116017.42 (83161.07 to 146005.98) | 116235.69 (83271.49 to 146211.22) | 116504.43 (83453.07 to 146436.12) | 116748.86 (83688.27 to 146593.27) |
| 10-14 years | 30864.14 (6412.68 to 86353.6) | 30644.79 (6473.24 to 85594.35) | 30256.01 (6368.3 to 84678.47) | 29812.36 (6151.73 to 83827.24) | 29429.35 (5919.91 to 83181.88) |
| Sociodemographic index |  |  |  |  |  |
| Low SDI | 58949.86 (38916.4 to 86172.21) | 58995.26 (39129.89 to 86018.11) | 59100.1 (39348.54 to 85972.4) | 59223.24 (39513.78 to 86097.57) | 59330.03 (39612.18 to 86124.28) |
| Low-middle SDI | 63881.57 (40327.79 to 97310.2) | 63831.29 (40427.5 to 97113.91) | 63804.25 (40547.38 to 96928.84) | 63781.74 (40655.84 to 96807.2) | 63744.4 (40736.1 to 96676.62) |
| Middle SDI | 64688.59 (41555.33 to 97116.88) | 64825.58 (41677.61 to 97117.77) | 64935.09 (41754.57 to 97043.71) | 65041.11 (41846.58 to 96940.25) | 65175.79 (41961.53 to 96940.93) |
| High-middle SDI | 68389.23 (42404.39 to 104429.58) | 68439.31 (42510.93 to 104447.78) | 68185.93 (42300.96 to 104138.4) | 67818.11 (41998.64 to 103755.44) | 67554.43 (41753.18 to 103380.25) |
| High SDI | 53912.97 (33480.09 to 81205.27) | 53947.04 (33487.52 to 81038.33) | 53963.97 (33577.68 to 80900.51) | 53993.86 (33660.35 to 80806.84) | 54048.88 (33702.53 to 80715.89) |
| **Caries of permanents teeth** |  |  |  |  |  |
| Global | 38182.81 (28574.35 to 48422.74) | 38253.57 (28617.08 to 48520.66) | 38363.99 (28683.66 to 48695.3) | 38491.18 (28758.14 to 48887.1) | 38612.8 (28838.29 to 49058.64) |
| Sex |  |  |  |  |  |
| Female | 38334.72 (28640.22 to 48652.72) | 38395.64 (28674.89 to 48729.3) | 38498.73 (28747.19 to 48863.48) | 38619.88 (28833.59 to 49036.98) | 38735.95 (28911.1 to 49213.88) |
| Male | 38038.64 (28504.61 to 48225.46) | 38118.6 (28568.61 to 48361.29) | 38235.66 (28629.72 to 48539.27) | 38368.19 (28700.13 to 48757.58) | 38494.74 (28787.73 to 48938.21) |
| Age* |  |  |  |  |  |
| 5-9 years | 16598.17 (8561.55 to 28716.2) | 16539.84 (8506.15 to 28699.37) | 16499.87 (8448.2 to 28733.5) | 16475.56 (8395.38 to 28799.45) | 16465.1 (8353.19 to 28875.87) |
| 10-14 years | 33855.78 (23329.95 to 45826.3) | 33995.49 (23448.1 to 46029.99) | 34231.67 (23595.08 to 46449.92) | 34507.61 (23741.07 to 46928.05) | 34767.81 (23878.3 to 47372.69) |
| 15-19 years | 44377.06 (34450.96 to 54663.76) | 44519.12 (34573.51 to 54798.11) | 44769.24 (34802.73 to 55123.24) | 45066.66 (35078.16 to 55459.86) | 45349.21 (35343.56 to 55688.87) |
| 20-24 years | 52994.95 (43207.61 to 62290.14) | 53050.74 (43172.33 to 62383.15) | 53111.85 (43127.66 to 62488.09) | 53178.77 (43082.8 to 62596.53) | 53254.73 (43090.34 to 62698.93) |
| 25-29 years | 47711.89 (36446.43 to 58502.98) | 47737.94 (36450.08 to 58548.81) | 47771.9 (36462.61 to 58611.37) | 47812.73 (36484.66 to 58704.19) | 47861.2 (36514.32 to 58827.83) |
| 30-34 years | 41443.16 (31882.95 to 49732.67) | 41533.88 (31919.35 to 49812.08) | 41628.18 (31967.04 to 49895.91) | 41711.63 (32021.16 to 49944.79) | 41766.86 (32074.1 to 49974.27) |
| 35-39 years | 35880.09 (27637.04 to 43685.22) | 36001.44 (27761.72 to 43845.31) | 36148 (27908.55 to 44014.56) | 36298.69 (28041.46 to 44189.98) | 36433.95 (28162.87 to 44351.17) |
| Sociodemographic index |  |  |  |  |  |
| Low SDI | 37545.46 (28571.76 to 47326.6) | 37565.01 (28600.77 to 47326.67) | 37594.81 (28631.49 to 47353.1) | 37634.88 (28656.37 to 47414.17) | 37686.56 (28680.23 to 47478.91) |
| Low-middle SDI | 38093.94 (27939.13 to 49015.76) | 38149.28 (28000.25 to 49050.12) | 38234.81 (28087.09 to 49145.26) | 38346.75 (28179.76 to 49266) | 38481.87 (28294.71 to 49447.04) |
| Middle SDI | 38700.05 (28456.33 to 49514.89) | 38786.11 (28526.81 to 49587.21) | 38912.85 (28629.29 to 49713.14) | 39054.8 (28711.23 to 49916.26) | 39185.35 (28808.02 to 50092.61) |
| High-middle SDI | 37835.8 (27633.86 to 48464.87) | 37937.38 (27742.99 to 48614.46) | 38083.86 (27886.09 to 48846.9) | 38231.75 (27999.13 to 49115.42) | 38340.05 (28047.95 to 49338.29) |
| High SDI | 38918.95 (30812.84 to 47693.87) | 39063.31 (30911.42 to 47851.01) | 39323.46 (31083.38 to 48131.95) | 39621.83 (31272.22 to 48492.73) | 39874.65 (31422.27 to 48814.02) |
| **Periodontal diseases** |  |  |  |  |  |
| Global | 1072.33 (685.76 to 1510.74) | 1086.58 (692.87 to 1533.19) | 1109.74 (708.24 to 1571.75) | 1135.13 (722.68 to 1614.4) | 1156.71 (735.64 to 1651.15) |
| Sex |  |  |  |  |  |
| Female | 1053.43 (670.92 to 1486.87) | 1066.85 (678.98 to 1510.07) | 1088.12 (693.89 to 1546.77) | 1111.32 (708.17 to 1588.39) | 1131.09 (716.69 to 1624.76) |
| Male | 1090.9 (695.64 to 1541.19) | 1105.96 (707.37 to 1559.33) | 1130.96 (720.51 to 1595.06) | 1158.48 (737.3 to 1637.17) | 1181.8 (752.33 to 1673.73) |
| Age* |  |  |  |  |  |
| 15-19 years | 420.97 (261.72 to 639.81) | 433.17 (265.92 to 657.07) | 448.03 (277.11 to 681.15) | 462.81 (284.69 to 703.11) | 475.03 (291.64 to 719.91) |
| 20-24 years | 868.73 (562.12 to 1189.48) | 882.95 (574.62 to 1210.27) | 904.17 (588.63 to 1246.62) | 927.86 (598.97 to 1283.61) | 949.84 (613.84 to 1322.65) |
| 25-29 years | 1241.75 (752.32 to 1758.02) | 1245.33 (754.03 to 1768.63) | 1255.2 (760.56 to 1794.25) | 1269.46 (765.27 to 1831.67) | 1284.31 (770.34 to 1868.32) |
| 30-34 years | 1410.43 (936.07 to 1962.22) | 1428.1 (939.19 to 1992.64) | 1456.52 (954.32 to 2041.62) | 1486.73 (970.35 to 2097.52) | 1511.47 (978.41 to 2141.72) |
| 35-39 years | 1544.78 (999.39 to 2176.04) | 1569.74 (1014.17 to 2211.31) | 1614.32 (1046.25 to 2273.16) | 1661.75 (1082.82 to 2339.01) | 1698.32 (1114.9 to 2389.48) |
| Sociodemographic index |  |  |  |  |  |
| Low SDI | 1275.8 (815.34 to 1784.02) | 1275 (818.19 to 1786.19) | 1270.8 (813.06 to 1786.06) | 1265.08 (806.75 to 1789.54) | 1260.42 (800.89 to 1795.15) |
| Low-middle SDI | 1497.86 (978.98 to 2028.31) | 1502.5 (988.42 to 2032.21) | 1505.69 (991.77 to 2039.13) | 1507.61 (988.2 to 2052.18) | 1508.51 (981.62 to 2065.5) |
| Middle SDI | 1006.23 (622.26 to 1439.25) | 1021.83 (638.23 to 1458.34) | 1048.6 (654.76 to 1496.34) | 1078.17 (672.7 to 1538.07) | 1102.93 (689.92 to 1570.9) |
| High-middle SDI | 716.15 (429.57 to 1120.03) | 735.41 (442.29 to 1150.36) | 776.57 (468.05 to 1210.77) | 824.94 (499.82 to 1283.69) | 866.23 (524.42 to 1346.85) |
| High SDI | 734.07 (444.52 to 1134.68) | 744.7 (449.65 to 1154.13) | 768.26 (462.06 to 1191.67) | 796.58 (478.07 to 1237.68) | 821 (491.32 to 1280.35) |
| **Edentulism** |  |  |  |  |  |
| Global | 104.5 (61.27 to 160.3) | 105.54 (62.38 to 161.13) | 106.97 (63.95 to 162.58) | 108.37 (65.4 to 164.14) | 109.23 (66.38 to 164.73) |
| Sex |  |  |  |  |  |
| Female | 109.6 (65.83 to 166.57) | 111.13 (67.31 to 168.38) | 113.57 (69.26 to 171.33) | 116.31 (71.43 to 174.56) | 118.64 (73.33 to 177.59) |
| Male | 99.47 (57.25 to 154.59) | 100.02 (57.89 to 154.7) | 100.45 (58.46 to 154.45) | 100.52 (59.12 to 153.4) | 99.95 (59.33 to 151.98) |
| Age* |  |  |  |  |  |
| 20-24 years | 80.21 (47.29 to 120.37) | 81.51 (48.25 to 121.68) | 83.04 (49.38 to 123.28) | 84.51 (50.29 to 124.67) | 85.54 (51.07 to 125.43) |
| 25-29 years | 105.82 (62.67 to 157.87) | 105.85 (63.13 to 157.37) | 105.87 (63.79 to 156.78) | 105.62 (63.79 to 155.81) | 104.94 (63.53 to 154.61) |
| 30-34 years | 107.96 (64.76 to 167.76) | 109.27 (66.12 to 168.88) | 111.2 (67.78 to 171) | 113.03 (69.61 to 173.53) | 113.94 (70.82 to 174.28) |
| 35-39 years | 127.07 (71.93 to 200.64) | 128.65 (73.67 to 202.07) | 131 (76.65 to 204.89) | 133.67 (79.93 to 208.43) | 136.01 (82.3 to 210.62) |
| Sociodemographic index |  |  |  |  |  |
| Low SDI | 140.44 (78.74 to 221.14) | 139.34 (78.13 to 219.09) | 137.11 (76.84 to 215.26) | 134.2 (75.21 to 210.54) | 131.11 (73.49 to 205.5) |
| Low-middle SDI | 124.08 (69.21 to 197.61) | 123.78 (69.45 to 196.52) | 122.56 (69.34 to 193.59) | 120.59 (68.82 to 189.2) | 118.05 (67.99 to 184.13) |
| Middle SDI | 99.05 (61.59 to 149.17) | 100.6 (62.71 to 150.79) | 102.87 (64.37 to 153.83) | 105.25 (66.11 to 156.46) | 107.02 (67.39 to 158.67) |
| High-middle SDI | 80.22 (49.28 to 122.54) | 81.04 (50.18 to 122.78) | 81.93 (51.04 to 122.67) | 82.83 (52.26 to 123.2) | 83.58 (53.1 to 124.16) |
| High SDI | 81.49 (47.56 to 127.18) | 84.28 (49.32 to 131.46) | 90.56 (52.99 to 141.33) | 98.16 (57.34 to 153.09) | 104.9 (60.99 to 163.36) |
| **Characteristic** | 2015 | 2016 | 2017 | 2018 | 2019 |
| **Caries of deciduous teeth** |  |  |  |  |  |
| Global | 62825.62 (40581.78 to 93453.96) | 62863.27 (40653.42 to 93282.34) | 62962.39 (40774.27 to 93246.76) | 63097.75 (40931.74 to 93310.45) | 63225.3 (41073.05 to 93373.56) |
| Sex |  |  |  |  |  |
| Female | 62593.79 (40360.9 to 92921.03) | 62635.05 (40484.78 to 92798.68) | 62723.99 (40661.21 to 92757.67) | 62841.54 (40839.14 to 92814.41) | 62950.65 (40970.46 to 92804) |
| Male | 63042.55 (40803.28 to 94084.98) | 63076.93 (40857.8 to 93860.29) | 63185.67 (40954.62 to 93717.33) | 63337.81 (41015.6 to 93703.82) | 63482.78 (41077.92 to 93794.31) |
| Age* |  |  |  |  |  |
| 2-4 years | 59121.4 (41684.7 to 78726.24) | 59343.32 (41848.26 to 78897.88) | 59725.9 (42060.63 to 79165) | 60126.93 (42232.95 to 79407.01) | 60410.77 (42330.58 to 79632.83) |
| 5-9 years | 116875.65 (83842.58 to 146592.82) | 116984.31 (83912.95 to 146476.79) | 117187.36 (84035.14 to 146586.06) | 117400.31 (84259.88 to 146762.9) | 117531.91 (84431.7 to 146696.53) |
| 10-14 years | 29219.26 (5803.43 to 82801.28) | 29050.86 (5841.06 to 82148.25) | 28785.71 (5784.83 to 81405.48) | 28532.66 (5681.21 to 80814.61) | 28395.53 (5637.04 to 80464.31) |
| Sociodemographic index |  |  |  |  |  |
| Low SDI | 59389.19 (39639.32 to 86120.62) | 59422.34 (39676.77 to 86118.64) | 59453.01 (39726.59 to 86148.95) | 59460.62 (39753.62 to 86071.06) | 59433.61 (39756.88 to 86085.05) |
| Low-middle SDI | 63675.76 (40793.46 to 96428.87) | 63667.42 (40900.59 to 95963.48) | 63762.97 (40962.51 to 95608.67) | 63872.29 (41194.99 to 95347.65) | 63885.88 (41439.48 to 95079.57) |
| Middle SDI | 65269.93 (42039.54 to 97007.35) | 65312.31 (42224.96 to 96836.35) | 65382.28 (42378.08 to 96731.8) | 65504.17 (42519.69 to 96811.06) | 65667.57 (42685.3 to 97057.92) |
| High-middle SDI | 67481.49 (41777.16 to 103180.83) | 67529.72 (41671.94 to 103261.18) | 67632.63 (41679.58 to 103379.23) | 67816.94 (41861.75 to 103559.41) | 68068.92 (42094.48 to 103888.3) |
| High SDI | 54127.41 (33780.48 to 80709.93) | 54321.03 (34024.6 to 80892.14) | 54705.98 (34437.7 to 81430.58) | 55250.66 (35005.17 to 82140.12) | 55921.43 (35635.25 to 83130.51) |
| **Caries of permanents teeth** |  |  |  |  |  |
| Global | 38708.13 (28908.09 to 49205.41) | 38836.84 (29020.74 to 49339.17) | 39024.39 (29186.64 to 49519.03) | 39198.83 (29345.38 to 49682.61) | 39292.47 (29433.71 to 49782.3) |
| Sex |  |  |  |  |  |
| Female | 38825.23 (28959.07 to 49351.07) | 38952.06 (29065.95 to 49470.83) | 39144.76 (29240.8 to 49655.8) | 39326.84 (29397.15 to 49860.72) | 39424.83 (29506.14 to 49980.87) |
| Male | 38595.63 (28857.82 to 49084.08) | 38726.04 (28979.77 to 49209.6) | 38908.65 (29143.62 to 49367.3) | 39075.8 (29292.01 to 49510.03) | 39165.32 (29384.14 to 49590.88) |
| Age* |  |  |  |  |  |
| 5-9 years | 16469.1 (8331.06 to 28939.27) | 16593.86 (8386.7 to 29153.65) | 16844.46 (8508.62 to 29527.06) | 17079.98 (8626.25 to 29880.08) | 17177.85 (8677.1 to 30049.07) |
| 10-14 years | 34954.71 (23972.63 to 47680.95) | 35107.78 (24036.5 to 47891.33) | 35267.61 (24143.53 to 48029.52) | 35385.71 (24245.69 to 48070.89) | 35443.97 (24318.78 to 48149.73) |
| 15-19 years | 45556.56 (35575.15 to 55909.59) | 45748.43 (35748.8 to 56195.09) | 45982.29 (35931.64 to 56519.4) | 46187.52 (36074.39 to 56810.28) | 46296.75 (36136.68 to 56960.07) |
| 20-24 years | 53344.56 (43133.74 to 62797.35) | 53547.18 (43354.77 to 62970.52) | 53883 (43707.02 to 63234.36) | 54219.61 (44063.61 to 63491.39) | 54408.06 (44284.06 to 63641.92) |
| 25-29 years | 47911.9 (36529.92 to 58938.06) | 48021.37 (36669.41 to 58937.18) | 48212.3 (36914.61 to 59009.89) | 48425.33 (37177.49 to 59138.99) | 48586.32 (37348.03 to 59254.37) |
| 30-34 years | 41783.71 (32110.65 to 50030.19) | 41788.45 (32153.59 to 49954.37) | 41804.2 (32228.18 to 49902.8) | 41819.27 (32305.59 to 49863.08) | 41829.21 (32344 to 49847.04) |
| 35-39 years | 36541.87 (28260.06 to 44506.65) | 36637.28 (28364.6 to 44570.63) | 36727.04 (28446.51 to 44627.58) | 36794.59 (28502.76 to 44679.18) | 36816.49 (28506.91 to 44696.09) |
| Sociodemographic index |  |  |  |  |  |
| Low SDI | 37750.57 (28715.99 to 47556.81) | 37918.12 (28868.67 to 47756.61) | 38197.76 (29106.65 to 48074.4) | 38465.69 (29334.11 to 48395.73) | 38601.29 (29416.96 to 48564.99) |
| Low-middle SDI | 38637.58 (28430.59 to 49639.03) | 39002.83 (28743.74 to 50030.05) | 39596.62 (29261.18 to 50653.4) | 40165.21 (29807.92 to 51263.18) | 40463.89 (30092.09 to 51571.49) |
| Middle SDI | 39281.97 (28883.39 to 50225.69) | 39348.1 (28956.12 to 50197.9) | 39401.49 (29047.08 to 50170.58) | 39440.34 (29134.26 to 50151.71) | 39470.66 (29185.01 to 50133.76) |
| High-middle SDI | 38374.11 (28031.91 to 49458.5) | 38244.5 (27966.27 to 49175.75) | 37966.39 (27780.92 to 48768.59) | 37668.67 (27585.16 to 48392.14) | 37491.41 (27439.05 to 48179.15) |
| High SDI | 39987.51 (31476.95 to 49010.93) | 39962.57 (31455.72 to 49029.87) | 39889.32 (31356.46 to 49043.79) | 39811.9 (31259.46 to 49077.31) | 39774.65 (31194.47 to 49104.29) |
| **Periodontal diseases** |  |  |  |  |  |
| Global | 1168.89 (742.03 to 1673.73) | 1172.48 (743.64 to 1679.59) | 1173.54 (744.1 to 1684.25) | 1174.53 (743.99 to 1690.35) | 1177.94 (745.53 to 1699.58) |
| Sex |  |  |  |  |  |
| Female | 1142.59 (721.37 to 1651.22) | 1147.77 (723.86 to 1659.05) | 1152.24 (727.94 to 1664.36) | 1156.85 (731.67 to 1669.76) | 1162.41 (735.53 to 1676.05) |
| Male | 1194.61 (758.94 to 1695.56) | 1196.63 (760.57 to 1701.96) | 1194.35 (759.5 to 1704.23) | 1191.8 (756.64 to 1709.42) | 1193.1 (755.47 to 1719.14) |
| Age* |  |  |  |  |  |
| 15-19 years | 482.14 (294.14 to 734.06) | 476.9 (290.69 to 726.6) | 460.45 (280.4 to 707.47) | 443.34 (270.24 to 684.96) | 435.95 (263.24 to 676.99) |
| 20-24 years | 966.33 (623.26 to 1358.26) | 971.95 (624.46 to 1377.71) | 968.37 (619.02 to 1387.25) | 962.73 (613.31 to 1396.65) | 962.96 (610.17 to 1410.78) |
| 25-29 years | 1295.1 (775.38 to 1892.92) | 1305.03 (781.81 to 1909.24) | 1318.75 (793.02 to 1931.67) | 1334.47 (805.7 to 1955.1) | 1350 (817.75 to 1977.54) |
| 30-34 years | 1523.31 (988.82 to 2163.18) | 1528.76 (993.04 to 2173.32) | 1537.62 (998.18 to 2187.35) | 1546.48 (997.97 to 2198.71) | 1552.01 (1000.87 to 2209.74) |
| 35-39 years | 1713.67 (1119.75 to 2406.36) | 1716.62 (1119.82 to 2396.63) | 1721.79 (1123.1 to 2394.82) | 1727.51 (1127.46 to 2406.95) | 1731.89 (1131.58 to 2414.85) |
| Sociodemographic index |  |  |  |  |  |
| Low SDI | 1259.47 (798.51 to 1803.39) | 1262.12 (801.61 to 1806.41) | 1265.81 (806.08 to 1808.75) | 1269.19 (809.49 to 1809.16) | 1270.9 (811.34 to 1808.42) |
| Low-middle SDI | 1508.79 (980.45 to 2071.92) | 1505.67 (979.2 to 2074.79) | 1498.67 (969.59 to 2078.8) | 1491.86 (958.12 to 2085.37) | 1489.5 (951.41 to 2096.67) |
| Middle SDI | 1115.76 (700.69 to 1589.35) | 1118.21 (704.18 to 1592.86) | 1118.09 (705.82 to 1591.16) | 1118.24 (706.6 to 1589.71) | 1121.27 (710.55 to 1594.04) |
| High-middle SDI | 886.34 (533.3 to 1380.62) | 889 (532.94 to 1389.74) | 889.2 (530.02 to 1397.43) | 889.24 (527.43 to 1408.09) | 891.17 (525.87 to 1423.64) |
| High SDI | 832.83 (495.3 to 1303.56) | 834.82 (495.42 to 1308.72) | 836.06 (494.29 to 1315.21) | 836.62 (493.1 to 1321.13) | 836.94 (491.62 to 1327.03) |
| **Edentulism** |  |  |  |  |  |
| Global | 109.07 (66.72 to 163.91) | 105.7 (65.2 to 158.45) | 99.24 (61.34 to 148.76) | 92.82 (57.43 to 139.31) | 89.73 (55.48 to 134.72) |
| Sex |  |  |  |  |  |
| Female | 119.81 (74.37 to 179.04) | 116.84 (72.97 to 174.01) | 109.68 (68.55 to 163.45) | 102.19 (63.79 to 152.77) | 98.43 (61.45 to 147.42) |
| Male | 98.5 (59.07 to 149.46) | 94.74 (57.46 to 143.19) | 88.98 (54.31 to 134.43) | 83.62 (51.2 to 126.54) | 81.19 (49.79 to 122.91) |
| Age* |  |  |  |  |  |
| 20-24 years | 85.73 (51.23 to 125.09) | 80.42 (48.64 to 117.33) | 68.98 (41.78 to 101.09) | 57.37 (34.35 to 84.15) | 51.88 (30.74 to 76.23) |
| 25-29 years | 103.61 (62.97 to 152.9) | 98.7 (60.32 to 145.07) | 89.94 (55.25 to 131.37) | 81.41 (50.06 to 119.01) | 77.55 (47.69 to 113.53) |
| 30-34 years | 113.14 (71.25 to 172.27) | 109.81 (70.13 to 166.8) | 104.85 (67.01 to 159.26) | 100.22 (64.3 to 152.4) | 97.81 (62.85 to 148.69) |
| 35-39 years | 137.45 (83.74 to 211.55) | 137.98 (84.26 to 211.37) | 138.14 (84.39 to 211.31) | 138.07 (84.61 to 210.85) | 137.84 (84.49 to 210.12) |
| Sociodemographic index |  |  |  |  |  |
| Low SDI | 128.32 (71.94 to 200.97) | 123.52 (68.99 to 193.54) | 116.08 (64.87 to 182.08) | 108.97 (61.06 to 171.19) | 105.17 (59.07 to 165.37) |
| Low-middle SDI | 115.1 (66.79 to 178.8) | 107.88 (64.23 to 165.48) | 95.94 (58.06 to 146.23) | 84.54 (51.22 to 128.83) | 78.94 (47.89 to 119.92) |
| Middle SDI | 107.42 (67.9 to 158.95) | 104.42 (65.85 to 154.11) | 98.42 (61.98 to 145.27) | 92.42 (58.1 to 137) | 89.65 (56.26 to 133.02) |
| High-middle SDI | 84.04 (53.57 to 124.59) | 83.33 (52.94 to 123.46) | 81.4 (51.46 to 120.57) | 79.26 (50.07 to 117.13) | 78.05 (49.32 to 115.23) |
| High SDI | 108.46 (62.95 to 168.65) | 108.08 (62.37 to 168.41) | 105.56 (60.67 to 165.23) | 102.82 (59.06 to 161.5) | 101.92 (58.44 to 160.43) |
| **Characteristic** | 2020 | 2021 |  |  |  |
| **Caries of deciduous teeth** |  |  |  |  |  |
| Global | 63127.13 (41744.63 to 91926.01) | 62261.07 (41905.15 to 89708.76) |  |  |  |
| Sex |  |  |  |  |  |
| Female | 62285.08 (41869.23 to 89931.41) | 61743.74 (41709.07 to 88345.38) |  |  |  |
| Male | 63917.53 (41666.07 to 94103.46) | 62746.73 (42172.97 to 90639.04) |  |  |  |
| Age* |  |  |  |  |  |
| 2-4 years | 61826.93 (43966.86 to 79937.34) | 62840.87 (44962.95 to 80481.03) |  |  |  |
| 5-9 years | 117398.13 (85219.62 to 145127.72) | 116346.23 (85293.92 to 144635.08) |  |  |  |
| 10-14 years | 27227.14 (5694.34 to 76502.56) | 25238.35 (5536.23 to 69794.53) |  |  |  |
| Sociodemographic index |  |  |  |  |  |
| Low SDI | 59174.09 (39648.07 to 85587.42) | 58395.89 (39749.16 to 83223.58) |  |  |  |
| Low-middle SDI | 62490.47 (41890.88 to 90671.66) | 60391.4 (41125.01 to 85720.76) |  |  |  |
| Middle SDI | 66144.38 (42926.54 to 97329.11) | 65823.34 (43727.82 to 96019.36) |  |  |  |
| High-middle SDI | 68523.4 (42187.05 to 104632.63) | 68399.3 (42326.88 to 104303.25) |  |  |  |
| High SDI | 58006.72 (38009.85 to 85471.87) | 58202.28 (38570.71 to 84969.78) |  |  |  |
| **Caries of permanents teeth** |  |  |  |  |  |
| Global | 39365.27 (29571.07 to 49778.5) | 39079.88 (29317.45 to 49476.57) |  |  |  |
| Sex |  |  |  |  |  |
| Female | 39470.08 (29632.21 to 49995.81) | 39175.06 (29405.19 to 49687.56) |  |  |  |
| Male | 39265.07 (29461.1 to 49603.49) | 38989.49 (29245.29 to 49284.51) |  |  |  |
| Age* |  |  |  |  |  |
| 5-9 years | 17100.03 (8591.4 to 29688.21) | 16214.09 (8100.48 to 28720.91) |  |  |  |
| 10-14 years | 35589.82 (24556.57 to 48118.56) | 35249.44 (24001.19 to 47938.37) |  |  |  |
| 15-19 years | 46412.16 (36439.77 to 57108.9) | 46221.21 (36336.8 to 56734.71) |  |  |  |
| 20-24 years | 54513.71 (44494.99 to 63686.54) | 54325.99 (44192.91 to 63552.01) |  |  |  |
| 25-29 years | 48737.13 (37466.65 to 59326.38) | 48664.43 (37423.82 to 59306.85) |  |  |  |
| 30-34 years | 41887.83 (32502.37 to 49884.97) | 41834.01 (32452.85 to 49912.94) |  |  |  |
| 35-39 years | 36845.32 (28540.64 to 44863.7) | 36770.85 (28439.36 to 44571.32) |  |  |  |
| Sociodemographic index |  |  |  |  |  |
| Low SDI | 38482.21 (29360.86 to 48367.64) | 38399.8 (29275.48 to 48414) |  |  |  |
| Low-middle SDI | 40445.25 (30007.83 to 51522.67) | 40086.55 (29697.07 to 51047.91) |  |  |  |
| Middle SDI | 39695.07 (29446.88 to 50378.69) | 39231.58 (29071.52 to 49933.21) |  |  |  |
| High-middle SDI | 37573.94 (27418.9 to 48378.45) | 37256.14 (27200.41 to 48101.37) |  |  |  |
| High SDI | 39857.97 (31170.34 to 49111.24) | 39968.46 (31279.53 to 49242.42) |  |  |  |
| **Periodontal diseases** |  |  |  |  |  |
| Global | 1181.98 (748.13 to 1708.6) | 1185.12 (744.5 to 1720.65) |  |  |  |
| Sex |  |  |  |  |  |
| Female | 1167.24 (736.45 to 1690.25) | 1170.99 (735.59 to 1700.9) |  |  |  |
| Male | 1196.35 (758.1 to 1727.89) | 1198.86 (754.47 to 1740.66) |  |  |  |
| Age* |  |  |  |  |  |
| 15-19 years | 437.11 (263.9 to 684.08) | 436.16 (260.9 to 684.6) |  |  |  |
| 20-24 years | 967.84 (614.48 to 1423.78) | 970.24 (606.35 to 1435.73) |  |  |  |
| 25-29 years | 1362.2 (829.3 to 1997.86) | 1373.12 (833.21 to 2015.69) |  |  |  |
| 30-34 years | 1553.38 (1000.34 to 2209.5) | 1556.68 (1003.05 to 2231.4) |  |  |  |
| 35-39 years | 1732.29 (1127.99 to 2419.03) | 1732.39 (1113.58 to 2428.19) |  |  |  |
| Sociodemographic index |  |  |  |  |  |
| Low SDI | 1271.81 (816.29 to 1808.53) | 1270.69 (814.41 to 1805.89) |  |  |  |
| Low-middle SDI | 1490.49 (949.5 to 2094.43) | 1488.37 (944.04 to 2104.3) |  |  |  |
| Middle SDI | 1123.7 (714.52 to 1608.31) | 1125.54 (712.69 to 1621.53) |  |  |  |
| High-middle SDI | 893.29 (523.3 to 1439.26) | 897.87 (527.75 to 1447.66) |  |  |  |
| High SDI | 833.92 (479.59 to 1345.63) | 831.52 (474.61 to 1343.53) |  |  |  |
| **Edentulism** |  |  |  |  |  |
| Global | 89.69 (55.68 to 133.99) | 89.21 (55.6 to 133.08) |  |  |  |
| Sex |  |  |  |  |  |
| Female | 98.28 (61.58 to 145.93) | 97.9 (61.35 to 145.06) |  |  |  |
| Male | 81.28 (49.89 to 122.63) | 80.71 (49.66 to 121.33) |  |  |  |
| Age* |  |  |  |  |  |
| 20-24 years | 51.9 (31.11 to 75.55) | 51.62 (31.39 to 74.39) |  |  |  |
| 25-29 years | 77.8 (48.32 to 114) | 77.59 (48.46 to 113.22) |  |  |  |
| 30-34 years | 97.53 (62.53 to 147.74) | 97.16 (62.47 to 147.53) |  |  |  |
| 35-39 years | 137.69 (84.59 to 208.25) | 136.56 (83.82 to 206.7) |  |  |  |
| Sociodemographic index |  |  |  |  |  |
| Low SDI | 104.34 (59.59 to 162.63) | 103.43 (59.83 to 159.97) |  |  |  |
| Low-middle SDI | 78.33 (48.05 to 118.03) | 77.39 (47.55 to 116.5) |  |  |  |
| Middle SDI | 89.75 (56.4 to 132.38) | 88.98 (56.04 to 131.62) |  |  |  |
| High-middle SDI | 77.95 (49.35 to 114.92) | 78.02 (49.56 to 115.12) |  |  |  |
| High SDI | 102.92 (59.05 to 162.26) | 103.36 (59.44 to 162.94) |  |  |  |

^*^Rate for age groups.

## Table D. The age-standardized prevalence rates per 100,000 populations of oral disorders, across different sexes, age groups, and SDI quintiles, in 1990-2021

| **Characteristic** | 1990 | 1991 | 1992 | 1993 | 1994 |
| --- | --- | --- | --- | --- | --- |
| **Caries of deciduous teeth** |  |  |  |  |  |
| Global | 28174.18 (20233.66 to 36437.02) | 28117.56 (20216.73 to 36353) | 28071.74 (20204.74 to 36270.23) | 28014.66 (20175.69 to 36166.29) | 27945.97 (20137.84 to 36041.54) |
| Sex |  |  |  |  |  |
| Female | 27994.75 (20237.75 to 36080.43) | 27949.05 (20192.91 to 36020.66) | 27909.79 (20142.41 to 35959.11) | 27856.68 (20083.99 to 35876.89) | 27789.47 (20018.93 to 35777.31) |
| Male | 28343.26 (20219.31 to 36751.31) | 28276.02 (20233.53 to 36638.24) | 28223.74 (20245.92 to 36533.41) | 28162.7 (20243.34 to 36410.08) | 28092.46 (20228.88 to 36272.4) |
| Age* |  |  |  |  |  |
| 2-4 years | 54502.01 (40167.2 to 66150.95) | 54260.75 (39981.37 to 65856.33) | 54017.5 (39803.83 to 65598.49) | 53779.15 (39637.63 to 65329.3) | 53556.11 (39490.16 to 65029.39) |
| 5-9 years | 41641.47 (31536.23 to 53697.29) | 41604.18 (31541.71 to 53654.48) | 41576.12 (31553.64 to 53594.13) | 41555.43 (31573.77 to 53519.21) | 41541.17 (31603.04 to 53420.35) |
| 10-14 years | 6080.48 (2008.62 to 10679.48) | 6055.06 (2011.46 to 10643.58) | 6032.91 (2005.91 to 10616.62) | 6014.47 (1995.08 to 10597.56) | 6000.33 (1982.95 to 10584.97) |
| Sociodemographic index |  |  |  |  |  |
| Low SDI | 27089.6 (19308.69 to 35211.62) | 27047.51 (19269.6 to 35124.43) | 27009.24 (19227.83 to 35045.42) | 26974.61 (19201.53 to 35009.85) | 26954.63 (19190.28 to 35011.77) |
| Low-middle SDI | 27893.38 (19431.3 to 36578.7) | 27906.96 (19434.93 to 36638.52) | 27925.26 (19445.08 to 36701.22) | 27942.31 (19443.09 to 36753.74) | 27956.22 (19441.41 to 36775.85) |
| Middle SDI | 29537.1 (21472.71 to 38022.31) | 29488.6 (21509.54 to 37915.03) | 29459.03 (21552.56 to 37818.64) | 29409.58 (21589.06 to 37694.85) | 29337.92 (21607.84 to 37547.1) |
| High-middle SDI | 30274.75 (21981.55 to 38705.29) | 30150.82 (21863.16 to 38661.8) | 30040.08 (21727.43 to 38629.39) | 29898.11 (21562.17 to 38549.22) | 29718.75 (21392 to 38401.05) |
| High SDI | 22912.67 (16911.31 to 29427.72) | 22765.47 (16802.71 to 29202.22) | 22652.93 (16721.91 to 29024.97) | 22555.41 (16672.74 to 28876.87) | 22458.92 (16599.93 to 28716.6) |
| **Caries of permanent teeth** |  |  |  |  |  |
| Global | 29782.7 (20127.5 to 41986.59) | 29766.45 (20263.16 to 41761.9) | 29753.15 (20392.14 to 41563.27) | 29741.92 (20509.63 to 41355.58) | 29727.4 (20626.31 to 41157.98) |
| Sex |  |  |  |  |  |
| Female | 30195.33 (20529.23 to 42341.27) | 30187.89 (20654 to 42156.28) | 30180.67 (20753.15 to 42009.89) | 30172.93 (20841.66 to 41862.45) | 30159.82 (20925.89 to 41723.44) |
| Male | 29383.3 (19726.57 to 41616.1) | 29358.87 (19879.32 to 41345.47) | 29340.07 (20036.91 to 41113.53) | 29325.79 (20185.2 to 40878.47) | 29310.09 (20337.08 to 40654.67) |
| Age* |  |  |  |  |  |
| 5-9 years | 9496.68 (4802.12 to 16003.35) | 9416.25 (4817.19 to 15816.43) | 9341.34 (4835.03 to 15635.78) | 9271.35 (4826.06 to 15464.97) | 9203.14 (4795.9 to 15253.03) |
| 10-14 years | 26313.73 (16793.25 to 37762.77) | 26321.32 (17010.12 to 37472.66) | 26317.95 (17206.25 to 37259.55) | 26289.51 (17353.63 to 37034.02) | 26220.17 (17435.41 to 36762.61) |
| 15-19 years | 30747.84 (20757.71 to 43162.3) | 30837.84 (20926.63 to 43021.17) | 30941.97 (21099.1 to 42980.84) | 31041.78 (21262.4 to 42905.85) | 31112.68 (21461.72 to 42742.92) |
| 20-24 years | 39397.01 (28281.66 to 51505.12) | 39318.27 (28409.42 to 51266.49) | 39259.81 (28513.09 to 51092.71) | 39219.49 (28596.75 to 50897.51) | 39205.04 (28771.34 to 50755.73) |
| 25-29 years | 38100.13 (26503.79 to 55100.7) | 37921.32 (26430.79 to 54638.64) | 37783.17 (26489.9 to 54238.43) | 37714.55 (26613.3 to 53945.85) | 37690.6 (26692.81 to 53730.59) |
| 30-34 years | 35509.88 (25745.66 to 50155.49) | 35608.65 (25973.12 to 50045.36) | 35588.19 (26039.2 to 49744.62) | 35446.53 (25984.76 to 49296.37) | 35252.96 (25925.9 to 48785.69) |
| 35-39 years | 35501.45 (23002.02 to 48660.94) | 35544.42 (23303.42 to 48524.56) | 35660.76 (23622.46 to 48432.57) | 35857.44 (24040.3 to 48373.21) | 36089.72 (24473.74 to 48542.61) |
| Sociodemographic index |  |  |  |  |  |
| Low SDI | 33348.78 (24091.48 to 44284.66) | 33452.99 (24221.1 to 44292.53) | 33540.97 (24316.74 to 44318.94) | 33621.06 (24397.85 to 44368.82) | 33670.8 (24480.48 to 44371.87) |
| Low-middle SDI | 31410.1 (21334.31 to 44040.56) | 31279.71 (21342.93 to 43758.23) | 31161.68 (21347.18 to 43505.52) | 31061.79 (21350.86 to 43292.81) | 30987.49 (21371.39 to 43115.51) |
| Middle SDI | 29255.74 (19163.31 to 42162.73) | 29280.1 (19288.06 to 42001.31) | 29306.66 (19401.78 to 41830.63) | 29333.7 (19502.6 to 41695) | 29359.03 (19593.83 to 41576.41) |
| High-middle SDI | 29839.14 (19268.6 to 42977.92) | 29844.82 (19508.57 to 42609.9) | 29834.25 (19704.87 to 42307.32) | 29799.9 (19888.67 to 41975.74) | 29733.44 (20022.82 to 41670.3) |
| High SDI | 26946.54 (19297.5 to 36935.62) | 26785.17 (19444.66 to 36357.9) | 26630.61 (19514.12 to 35835.19) | 26484.93 (19584.32 to 35412.45) | 26352.28 (19607.05 to 35058.04) |
| **Periodontal diseases** |  |  |  |  |  |
| Global | 8364.63 (5197.6 to 12112.46) | 8334.51 (5216.23 to 12004.59) | 8313.62 (5243.58 to 11911.35) | 8305.61 (5284.39 to 11824.63) | 8314.45 (5335.01 to 11777.37) |
| Sex |  |  |  |  |  |
| Female | 8125.29 (5061.44 to 11749.18) | 8100.37 (5087.62 to 11638.79) | 8084.96 (5130.37 to 11542.4) | 8082.7 (5179.83 to 11472.69) | 8097.5 (5234.42 to 11416.39) |
| Male | 8597.84 (5322.25 to 12471.71) | 8562.55 (5337.64 to 12352.63) | 8536.23 (5355.74 to 12255.87) | 8522.62 (5385.2 to 12172.05) | 8525.79 (5426.86 to 12115.49) |
| Age* |  |  |  |  |  |
| 15-19 years | 1127.65 (551.88 to 2026.67) | 1149.35 (567.87 to 2060.62) | 1173.18 (585.09 to 2097.3) | 1197.25 (600.99 to 2133.58) | 1218.61 (614.43 to 2164.43) |
| 20-24 years | 3725.74 (2073.11 to 6028.84) | 3765.39 (2105.87 to 6078.6) | 3812.6 (2141.81 to 6138.61) | 3862.86 (2179.16 to 6206.3) | 3917.14 (2219.02 to 6277.69) |
| 25-29 years | 7923.55 (4734.28 to 11743.27) | 7905.58 (4754.1 to 11605.58) | 7903.25 (4782.14 to 11497.98) | 7931.59 (4842.65 to 11432.61) | 7981.26 (4915.97 to 11401.29) |
| 30-34 years | 12864.32 (8252.97 to 18445.48) | 12904.81 (8360.47 to 18376.74) | 12910.51 (8448.86 to 18260.53) | 12851.18 (8494.61 to 18056.85) | 12751.55 (8511 to 17828.71) |
| 35-39 years | 18115.61 (11646.08 to 24926.68) | 17853.66 (11555.13 to 24457.74) | 17651.52 (11518.78 to 24072.29) | 17552.59 (11566.6 to 23761.53) | 17565.23 (11686.07 to 23660.12) |
| Sociodemographic index |  |  |  |  |  |
| Low SDI | 15323.73 (10032.1 to 21050.95) | 15367.08 (10084.2 to 21095.66) | 15401.73 (10130.88 to 21118.67) | 15416.63 (10165.45 to 21098.74) | 15410.81 (10186.46 to 21051.08) |
| Low-middle SDI | 12138.74 (7774.18 to 17075.71) | 12290.8 (7908.7 to 17206.23) | 12430.07 (8032.23 to 17334.4) | 12552.41 (8143.26 to 17444.03) | 12652.01 (8236.1 to 17530.89) |
| Middle SDI | 7520.65 (4553.68 to 11111.52) | 7392.52 (4503.5 to 10854.73) | 7282.35 (4465.43 to 10655.33) | 7200.24 (4447.88 to 10492.26) | 7159.5 (4460.82 to 10375.93) |
| High-middle SDI | 5678.96 (3269.99 to 8786.85) | 5455.92 (3157.73 to 8445.55) | 5245.91 (3050.78 to 8104.06) | 5064.13 (2956.63 to 7801.03) | 4926.32 (2898.17 to 7560.11) |
| High SDI | 5912.38 (3760.7 to 8636.05) | 5989.78 (3899.02 to 8633.81) | 6065.93 (4021.28 to 8639.69) | 6129.86 (4133.64 to 8643.31) | 6173.7 (4229.78 to 8626.74) |
| **Edentulism** |  |  |  |  |  |
| Global | 709.56 (433.15 to 1018.48) | 704.23 (429.96 to 1010.66) | 700.31 (427.66 to 1005.8) | 698.49 (426.79 to 1003.59) | 699.08 (427.56 to 1004.63) |
| Sex |  |  |  |  |  |
| Female | 788.02 (482.81 to 1124.68) | 780.12 (478.12 to 1114.13) | 774.03 (474.57 to 1106.49) | 770.64 (472.84 to 1102.55) | 770.43 (473.17 to 1102.45) |
| Male | 633.03 (383.91 to 914.84) | 630.26 (382.52 to 909.69) | 628.48 (381.9 to 906.36) | 628.2 (382.33 to 905.68) | 629.53 (384 to 907.89) |
| Age* |  |  |  |  |  |
| 20-24 years | 161.75 (80.09 to 269.11) | 162.06 (80.43 to 269.35) | 163.03 (81.1 to 270.66) | 164.44 (81.97 to 272.93) | 166.29 (83.04 to 275.85) |
| 25-29 years | 464.01 (258.74 to 682.1) | 454.19 (253.87 to 668.7) | 446.74 (250.2 to 659.39) | 443.16 (248.62 to 655.67) | 442.63 (248.69 to 656.29) |
| 30-34 years | 892.52 (540.2 to 1276.51) | 894.07 (541.55 to 1278.63) | 889.23 (538.8 to 1272.78) | 876.3 (531.14 to 1253.59) | 860.16 (521.75 to 1229.42) |
| 35-39 years | 1412.25 (915.63 to 1971.95) | 1398.13 (905.42 to 1950.48) | 1393.48 (901.73 to 1944.58) | 1401.86 (906.94 to 1957.13) | 1420.21 (919.07 to 1983.54) |
| Sociodemographic index |  |  |  |  |  |
| Low SDI | 1155.37 (723.22 to 1631.6) | 1164.48 (729.4 to 1643.29) | 1172.42 (734.69 to 1654.02) | 1179.38 (739.49 to 1663.83) | 1185.54 (743.54 to 1672.29) |
| Low-middle SDI | 832.25 (497.8 to 1204.45) | 832.83 (498.06 to 1204.65) | 832.54 (497.73 to 1204.08) | 831.44 (496.89 to 1202.4) | 829.76 (496.01 to 1199.79) |
| Middle SDI | 685.07 (420.68 to 983.8) | 671.58 (411.99 to 964.55) | 660.6 (404.93 to 949.02) | 653.4 (400.55 to 938.78) | 650.85 (399.27 to 935.19) |
| High-middle SDI | 646.48 (394.84 to 931.01) | 633.78 (388.22 to 911.12) | 622.18 (381.51 to 892.86) | 612.85 (376.2 to 878.68) | 605.9 (372.41 to 868.89) |
| High SDI | 495.79 (299.72 to 735.28) | 498.55 (300.81 to 739.98) | 502.95 (302.3 to 747.38) | 508.68 (304.81 to 756.7) | 517.09 (310.02 to 769.99) |
| **Other oral disorders** |  |  |  |  |  |
| Global | 1603.13 (1413.17 to 1797.73) | 1603.9 (1413.82 to 1798.78) | 1604.73 (1414.37 to 1799.99) | 1605.3 (1414.83 to 1800.72) | 1605.54 (1415.13 to 1801.03) |
| Sex |  |  |  |  |  |
| Female | 1737.52 (1525.21 to 1959.53) | 1738.36 (1526.15 to 1960.67) | 1739.26 (1527.32 to 1961.68) | 1739.87 (1528.23 to 1962.03) | 1740.11 (1528.84 to 1961.99) |
| Male | 1472.76 (1294.32 to 1658.72) | 1473.55 (1295 to 1659.43) | 1474.4 (1295.68 to 1660.33) | 1474.97 (1296.05 to 1660.91) | 1475.2 (1296.08 to 1661.23) |
| Age* |  |  |  |  |  |
| 2-4 years | 1405.2 (1237.58 to 1594.86) | 1405.61 (1238.12 to 1595.37) | 1405.98 (1238.68 to 1595.55) | 1406.3 (1239.19 to 1595.65) | 1406.51 (1239.37 to 1595.62) |
| 5-9 years | 1030.26 (883.06 to 1181.56) | 1030.4 (883.26 to 1181.76) | 1030.5 (883.42 to 1181.89) | 1030.54 (883.55 to 1181.93) | 1030.52 (883.63 to 1181.84) |
| 10-14 years | 1087.75 (931.73 to 1250.87) | 1087.72 (931.64 to 1250.81) | 1087.73 (931.59 to 1250.8) | 1087.77 (931.56 to 1250.83) | 1087.84 (931.57 to 1250.92) |
| 15-19 years | 1626.03 (1430.07 to 1818.45) | 1626.49 (1430.15 to 1819.16) | 1626.88 (1430.19 to 1820.85) | 1627.19 (1430.19 to 1822.05) | 1627.43 (1430.17 to 1822.88) |
| 20-24 years | 1989.56 (1783.16 to 2193.85) | 1989.84 (1783.68 to 2194.06) | 1990.22 (1784.25 to 2194.37) | 1990.73 (1784.87 to 2194.75) | 1991.3 (1785.51 to 2195.2) |
| 25-29 years | 2176.8 (1931.32 to 2410.88) | 2177.46 (1930.26 to 2411.58) | 2177.91 (1929.2 to 2412.08) | 2178.16 (1928.18 to 2412.36) | 2178.33 (1927.25 to 2412.6) |
| 30-34 years | 2301.81 (2037.77 to 2565.53) | 2303.05 (2040.16 to 2567.55) | 2304.13 (2040.89 to 2569.51) | 2305.06 (2041.92 to 2571.38) | 2305.8 (2042.86 to 2573.02) |
| 35-39 years | 2368.38 (2107.21 to 2660.01) | 2370.04 (2108.74 to 2662.93) | 2371.49 (2110.06 to 2665.2) | 2372.69 (2111.66 to 2666.02) | 2373.68 (2114.33 to 2666.39) |
| Sociodemographic index |  |  |  |  |  |
| Low SDI | 1599.4 (1410.54 to 1793.13) | 1599.27 (1410.42 to 1792.97) | 1599.08 (1410.25 to 1792.75) | 1598.84 (1410.06 to 1792.45) | 1598.75 (1409.99 to 1792.32) |
| Low-middle SDI | 1603.37 (1412.44 to 1800.86) | 1603.32 (1412.41 to 1800.77) | 1603.3 (1412.41 to 1800.72) | 1603.29 (1412.4 to 1800.7) | 1603.3 (1412.42 to 1800.71) |
| Middle SDI | 1606.39 (1411.01 to 1811.88) | 1606.71 (1411.33 to 1812.26) | 1607.25 (1411.91 to 1812.84) | 1607.56 (1412.35 to 1813.16) | 1607.5 (1412.43 to 1812.92) |
| High-middle SDI | 1605.97 (1414.62 to 1801.98) | 1606.91 (1415.42 to 1803.11) | 1608.03 (1416.35 to 1804.42) | 1608.67 (1416.84 to 1805.1) | 1608.55 (1416.66 to 1804.83) |
| High SDI | 1597.55 (1416.4 to 1784.71) | 1601.31 (1420.39 to 1788.89) | 1605.22 (1423.72 to 1791.45) | 1608.57 (1426.94 to 1793.77) | 1611.08 (1428.65 to 1796.25) |
| **Characteristic** | 1995 | 1996 | 1997 | 1998 | 1999 |
| **Caries of deciduous teeth** |  |  |  |  |  |
| Global | 27882.06 (20106.01 to 35917.74) | 27822.39 (20112.03 to 35801) | 27759.4 (20120.54 to 35674.8) | 27696.22 (20120.21 to 35545.56) | 27633.61 (20115.77 to 35413.89) |
| Sex |  |  |  |  |  |
| Female | 27723.23 (19961.44 to 35702.11) | 27651.94 (19984.82 to 35585.24) | 27566.81 (19988.86 to 35432.6) | 27476.82 (19979.76 to 35246.44) | 27389.18 (19966.91 to 35063.75) |
| Male | 28030.62 (20219.71 to 36140.63) | 27981.72 (20229.85 to 36045.97) | 27939.35 (20238.3 to 35955.72) | 27901.14 (20245.42 to 35868.44) | 27861.83 (20246.66 to 35782.54) |
| Age* |  |  |  |  |  |
| 2-4 years | 53370.05 (39375.83 to 64884.19) | 53183.21 (39315.39 to 64607.15) | 52969.13 (39286.47 to 64251.08) | 52745.8 (39247.33 to 63872.74) | 52533.03 (39206.81 to 63537.22) |
| 5-9 years | 41536.7 (31644.67 to 53309.5) | 41526.21 (31711.37 to 53221.38) | 41496.87 (31745.97 to 53112.18) | 41455.6 (31742.05 to 52996.91) | 41409.28 (31737.64 to 52862.97) |
| 10-14 years | 5992.07 (1974.85 to 10578.32) | 5987.22 (1998.43 to 10541.02) | 5982.99 (2025.12 to 10500.54) | 5980.11 (2046.51 to 10463.58) | 5978.6 (2060.58 to 10432.89) |
| Sociodemographic index |  |  |  |  |  |
| Low SDI | 26948.21 (19170.48 to 35042.95) | 26942.75 (19211.22 to 34987.54) | 26931.19 (19251.31 to 34913.77) | 26915.84 (19280.92 to 34841.02) | 26899.61 (19303.91 to 34744.89) |
| Low-middle SDI | 27966.09 (19438.19 to 36741.74) | 27948.66 (19464.57 to 36680.03) | 27896.08 (19483.23 to 36590.82) | 27827.02 (19515.93 to 36475.52) | 27758.91 (19502.3 to 36358.31) |
| Middle SDI | 29271.07 (21613.2 to 37409.72) | 29239.19 (21620.82 to 37325.82) | 29242.57 (21683.14 to 37266.39) | 29262.44 (21765.02 to 37217.99) | 29278.74 (21839.48 to 37163.89) |
| High-middle SDI | 29546.83 (21271.14 to 38246.08) | 29437.62 (21217.95 to 38057.99) | 29390 (21241.26 to 37911.86) | 29372.43 (21303.47 to 37793.14) | 29344.9 (21363.18 to 37653.5) |
| High SDI | 22361.34 (16529.38 to 28547.03) | 22167.26 (16464.18 to 28218.83) | 21842.39 (16282.18 to 27766.84) | 21477.4 (16098.25 to 27280.59) | 21149.49 (15874.82 to 26808.39) |
| **Caries of permanent teeth** |  |  |  |  |  |
| Global | 29706.84 (20727.89 to 40982.7) | 29679.72 (20732.53 to 40925.61) | 29647.3 (20734.83 to 40852.9) | 29616.22 (20733.08 to 40799.13) | 29597.19 (20733.41 to 40774.02) |
| Sex |  |  |  |  |  |
| Female | 30138.05 (20990.18 to 41563.64) | 30106.38 (20990.29 to 41493.43) | 30067.55 (20987.24 to 41422.24) | 30029.34 (20974.08 to 41370.64) | 30003.4 (20967.37 to 41337.93) |
| Male | 29290.88 (20469.56 to 40452.37) | 29268.35 (20475.46 to 40394.82) | 29242.35 (20481.45 to 40323.1) | 29218.45 (20482.96 to 40257.42) | 29206.38 (20484.73 to 40231.85) |
| Age* |  |  |  |  |  |
| 5-9 years | 9135.24 (4787.27 to 15054.47) | 9072.63 (4756.55 to 14941.83) | 9027.74 (4735.76 to 14853.7) | 9011.18 (4727.34 to 14809.99) | 9027.04 (4732.5 to 14825.04) |
| 10-14 years | 26099.07 (17482.62 to 36527.27) | 25924.26 (17354.83 to 36312.69) | 25726.49 (17216.64 to 36055.69) | 25549.68 (17107.81 to 35860.64) | 25431.72 (17036.02 to 35748.97) |
| 15-19 years | 31149.49 (21608.42 to 42579.3) | 31160.15 (21668.89 to 42587.09) | 31154.59 (21714.44 to 42588.6) | 31139.04 (21743.02 to 42601.9) | 31124.38 (21758.65 to 42645.61) |
| 20-24 years | 39224.53 (28967.94 to 50667.11) | 39270.13 (29073.99 to 50706.3) | 39318.11 (29183.06 to 50723.55) | 39359.83 (29282.72 to 50747.27) | 39386.42 (29364.74 to 50763.42) |
| 25-29 years | 37705.02 (26758.21 to 53582.93) | 37736.63 (26764.64 to 53613.49) | 37789.34 (26780.01 to 53632.22) | 37847.02 (26788.39 to 53653.05) | 37923.93 (26809.55 to 53693.81) |
| 30-34 years | 35062.71 (25935.56 to 48368.71) | 34924.23 (25859.96 to 48151.75) | 34837.07 (25832.03 to 47975.39) | 34831.92 (25835.89 to 47888.2) | 34874.18 (25863.64 to 47858.76) |
| 35-39 years | 36296.2 (24778.24 to 48594.66) | 36438.97 (24907.73 to 48708.79) | 36481.56 (24971.13 to 48717.23) | 36393.43 (24949.9 to 48618.26) | 36225.53 (24873.85 to 48451.08) |
| Sociodemographic index |  |  |  |  |  |
| Low SDI | 33678.92 (24531.94 to 44286.8) | 33634.24 (24529.33 to 44208.56) | 33540.49 (24478.3 to 44063.56) | 33433.28 (24411.76 to 43900.62) | 33349.02 (24349.49 to 43772.15) |
| Low-middle SDI | 30945.43 (21397.51 to 43005.5) | 30915.87 (21379.69 to 42967.82) | 30883.44 (21350.93 to 42907.28) | 30860.56 (21327.48 to 42888.67) | 30857.81 (21329.89 to 42896.26) |
| Middle SDI | 29373.45 (19674.71 to 41489.71) | 29400.28 (19715.75 to 41498.59) | 29452.44 (19775.23 to 41545.16) | 29508.89 (19829.38 to 41571.9) | 29562.36 (19882.56 to 41605.83) |
| High-middle SDI | 29633.66 (20104.02 to 41388.96) | 29498.1 (20006.55 to 41173.47) | 29336.73 (19911.06 to 40912.71) | 29172.18 (19826.96 to 40652.92) | 29031.63 (19756.83 to 40437.22) |
| High SDI | 26241.39 (19654.58 to 34686.27) | 26124.85 (19591.08 to 34494.22) | 25986.47 (19506.63 to 34254.85) | 25851.74 (19434.65 to 34018.33) | 25734.76 (19377.17 to 33810.99) |
| **Periodontal diseases** |  |  |  |  |  |
| Global | 8342.94 (5397.03 to 11763.84) | 8384.65 (5514.79 to 11724.54) | 8427.24 (5636.7 to 11680.56) | 8464.05 (5732.17 to 11629.84) | 8497.52 (5855 to 11589.95) |
| Sex |  |  |  |  |  |
| Female | 8131.37 (5294.78 to 11401.8) | 8177.1 (5425.85 to 11378.55) | 8222.33 (5517.68 to 11362.8) | 8260.96 (5636.55 to 11328.84) | 8296.52 (5716.84 to 11296.66) |
| Male | 8549.26 (5479.75 to 12101.97) | 8587.4 (5624.73 to 12045.46) | 8627.81 (5737.85 to 11997.48) | 8663.25 (5851.62 to 11919.24) | 8695.06 (5973.12 to 11883.61) |
| Age* |  |  |  |  |  |
| 15-19 years | 1237.06 (627.93 to 2189.35) | 1253.57 (656.2 to 2190.98) | 1267.91 (676.84 to 2190.82) | 1279.13 (690.2 to 2189.14) | 1287.26 (702.87 to 2185.39) |
| 20-24 years | 3980.64 (2264.87 to 6359.16) | 4053.15 (2347.38 to 6418.45) | 4128.43 (2439.44 to 6487.64) | 4202.09 (2507.84 to 6526.28) | 4268.88 (2580.2 to 6570.33) |
| 25-29 years | 8046.33 (4998.51 to 11427.63) | 8119.12 (5126.21 to 11445.05) | 8201.06 (5244.13 to 11468.73) | 8285.98 (5353.4 to 11472.29) | 8381.81 (5514.67 to 11550.16) |
| 30-34 years | 12653.7 (8524.49 to 17639.83) | 12602.07 (8613.09 to 17447.28) | 12603.43 (8683.63 to 17314.43) | 12664.14 (8852.78 to 17265.08) | 12755.77 (9061.36 to 17192.31) |
| 35-39 years | 17660.71 (11854.74 to 23635.05) | 17763.4 (12141.57 to 23534.69) | 17803.39 (12480.1 to 23328.27) | 17750.02 (12611.77 to 23054.13) | 17644.52 (12791.32 to 22778.77) |
| Sociodemographic index |  |  |  |  |  |
| Low SDI | 15396.93 (10201.39 to 20990.16) | 15333.56 (10216.55 to 20825.83) | 15197.06 (10172.59 to 20600.46) | 15033.66 (10097.5 to 20358.12) | 14895.07 (10036.65 to 20140.68) |
| Low-middle SDI | 12723.81 (8316.44 to 17577.82) | 12789.37 (8472.44 to 17539.45) | 12866.64 (8651.45 to 17533.79) | 12944.63 (8803.27 to 17530.99) | 13014.44 (8937.69 to 17486.08) |
| Middle SDI | 7164.58 (4504.51 to 10310.07) | 7210.77 (4603.26 to 10288) | 7276.76 (4720.19 to 10298.01) | 7339.81 (4834.92 to 10313.26) | 7393.9 (4910.81 to 10337.65) |
| High-middle SDI | 4846.11 (2878.63 to 7413.73) | 4799.68 (2957.49 to 7243.81) | 4755.71 (3036.99 to 7070.18) | 4714.48 (3092.88 to 6867.77) | 4684.56 (3131.6 to 6699.87) |
| High SDI | 6190.03 (4273.9 to 8579.77) | 6180.57 (4448.14 to 8418.6) | 6157.64 (4488.57 to 8262.73) | 6125.18 (4464.46 to 8130.59) | 6082.67 (4417.34 to 8040.7) |
| **Edentulism** |  |  |  |  |  |
| Global | 701.8 (429.85 to 1008.67) | 697.46 (428.95 to 1001.38) | 680.9 (420.94 to 975.56) | 658.7 (408.35 to 943.88) | 639.46 (397.28 to 916.49) |
| Sex |  |  |  |  |  |
| Female | 773.2 (475.5 to 1106.43) | 768.57 (474.25 to 1099.01) | 750.48 (465.6 to 1072.4) | 726.03 (452.56 to 1036.63) | 704.7 (440.28 to 1006.22) |
| Male | 632.13 (386.42 to 912.03) | 627.97 (384.3 to 903.62) | 612.76 (376.53 to 880.81) | 592.62 (364.64 to 851.7) | 575.31 (354.47 to 826.85) |
| Age* |  |  |  |  |  |
| 20-24 years | 168.66 (84.36 to 279.44) | 169.52 (84.95 to 281.06) | 167.63 (84.31 to 278.54) | 164.44 (83.03 to 273.39) | 161.63 (81.87 to 268.5) |
| 25-29 years | 444.7 (250.22 to 660.6) | 442.89 (249.54 to 654.36) | 434.66 (245.46 to 638.34) | 424.32 (240.23 to 622.74) | 417.29 (236.79 to 611.69) |
| 30-34 years | 845.07 (513.44 to 1206.98) | 825.02 (503.44 to 1180.57) | 796.8 (488.94 to 1136.7) | 770.17 (474.77 to 1095.44) | 751.39 (464.44 to 1066.02) |
| 35-39 years | 1443.16 (934.66 to 2016.17) | 1446.92 (941.57 to 2018.02) | 1416.83 (927.73 to 1974.06) | 1364.44 (895.71 to 1904.47) | 1312.36 (864.06 to 1835.38) |
| Sociodemographic index |  |  |  |  |  |
| Low SDI | 1190.59 (746.76 to 1679.06) | 1182.14 (743.53 to 1665.48) | 1156.37 (728.87 to 1625.2) | 1125.16 (710.09 to 1578.18) | 1099.73 (695.26 to 1542.16) |
| Low-middle SDI | 827.53 (494.81 to 1196.38) | 803.29 (483.19 to 1159.31) | 749.16 (452.53 to 1078.64) | 685.63 (415.2 to 988.51) | 632.95 (383.99 to 913.27) |
| Middle SDI | 652.08 (400.43 to 936.74) | 645.48 (397.12 to 927.6) | 624.44 (384.96 to 897.75) | 596.76 (368.32 to 857.76) | 573.22 (353.25 to 824.02) |
| High-middle SDI | 600.93 (370.08 to 862.55) | 599.4 (370.71 to 858.06) | 599.63 (373.31 to 855.27) | 597.65 (373.78 to 850.06) | 592.97 (371.27 to 842.28) |
| High SDI | 529.35 (317.64 to 787.43) | 543.53 (326.7 to 807.66) | 556.8 (334.27 to 828.56) | 568.92 (340.55 to 849.24) | 580.5 (347.14 to 868.48) |
| **Other oral disorders** |  |  |  |  |  |
| Global | 1605.57 (1415.33 to 1800.98) | 1605.55 (1414.94 to 1800.72) | 1605.55 (1414.68 to 1800.51) | 1605.53 (1414.38 to 1800.39) | 1605.44 (1414.12 to 1800.28) |
| Sex |  |  |  |  |  |
| Female | 1740.08 (1529.09 to 1961.67) | 1740.02 (1529.07 to 1961.13) | 1739.98 (1529.1 to 1961.3) | 1739.92 (1529.1 to 1961.6) | 1739.77 (1528.98 to 1961.88) |
| Male | 1475.23 (1295.86 to 1661.36) | 1475.2 (1296.24 to 1661.52) | 1475.14 (1296.66 to 1661.67) | 1475.07 (1296.9 to 1661.84) | 1474.96 (1296.92 to 1661.84) |
| Age* |  |  |  |  |  |
| 2-4 years | 1406.55 (1239.39 to 1595.55) | 1406.45 (1239.14 to 1595.67) | 1406.27 (1238.85 to 1595.47) | 1406.07 (1238.57 to 1595.24) | 1405.89 (1238.34 to 1594.99) |
| 5-9 years | 1030.42 (883.66 to 1181.63) | 1030.29 (883.27 to 1181.16) | 1030.14 (882.87 to 1180.64) | 1030 (882.45 to 1180.33) | 1029.85 (882.03 to 1180.27) |
| 10-14 years | 1087.95 (931.61 to 1251.06) | 1088.05 (931.6 to 1250.93) | 1088.14 (931.6 to 1250.77) | 1088.15 (931.56 to 1250.53) | 1088.07 (931.47 to 1250.17) |
| 15-19 years | 1627.54 (1430.09 to 1822.64) | 1627.63 (1429.97 to 1822) | 1627.75 (1429.92 to 1821.52) | 1627.85 (1429.9 to 1821.15) | 1627.88 (1429.89 to 1820.94) |
| 20-24 years | 1991.83 (1786.09 to 2195.64) | 1992.29 (1786.12 to 2195.4) | 1992.77 (1786.17 to 2195.16) | 1993.17 (1786.16 to 2194.85) | 1993.44 (1786.05 to 2194.42) |
| 25-29 years | 2178.5 (1926.41 to 2412.84) | 2178.63 (1926.45 to 2412.97) | 2178.74 (1926.47 to 2413.25) | 2178.87 (1926.47 to 2413.86) | 2178.99 (1926.44 to 2414.51) |
| 30-34 years | 2306.31 (2043.47 to 2574.34) | 2306.6 (2041.73 to 2574.58) | 2306.62 (2041.19 to 2574.51) | 2306.4 (2040.7 to 2574.14) | 2306.05 (2040.09 to 2573.63) |
| 35-39 years | 2374.38 (2117.65 to 2666.45) | 2374.8 (2117.95 to 2667) | 2374.99 (2117.48 to 2667.58) | 2374.98 (2116.21 to 2668.23) | 2374.84 (2115.74 to 2668.87) |
| Sociodemographic index |  |  |  |  |  |
| Low SDI | 1598.72 (1410 to 1792.26) | 1598.72 (1410.03 to 1792.22) | 1598.78 (1410.13 to 1792.27) | 1598.9 (1410.3 to 1792.42) | 1599.1 (1410.52 to 1792.67) |
| Low-middle SDI | 1603.37 (1412.49 to 1800.8) | 1603.48 (1412.6 to 1800.92) | 1603.59 (1412.71 to 1801.05) | 1603.76 (1412.86 to 1801.23) | 1603.99 (1413.06 to 1801.47) |
| Middle SDI | 1607.31 (1412.29 to 1812.53) | 1607.27 (1412.23 to 1812.37) | 1607.4 (1412.32 to 1812.46) | 1607.54 (1412.44 to 1812.63) | 1607.55 (1412.45 to 1812.68) |
| High-middle SDI | 1607.97 (1416.1 to 1804.04) | 1607.46 (1415.65 to 1803.36) | 1607.15 (1415.39 to 1802.97) | 1606.88 (1415.19 to 1802.64) | 1606.41 (1414.83 to 1802.11) |
| High SDI | 1612.44 (1428.58 to 1797.76) | 1612.96 (1429.85 to 1797.71) | 1612.93 (1429.27 to 1796.98) | 1612.56 (1426.7 to 1797.33) | 1611.91 (1424.83 to 1797.93) |
| **Characteristic** | 2000 | 2001 | 2002 | 2003 | 2004 |
| **Caries of deciduous teeth** |  |  |  |  |  |
| Global | 27573.03 (20103.72 to 35292.75) | 27482.18 (20109.54 to 35110.79) | 27348.19 (20042.19 to 34901.21) | 27202.35 (19961.59 to 34680.11) | 27073.05 (19892.69 to 34473.54) |
| Sex |  |  |  |  |  |
| Female | 27311.61 (19946.48 to 34938.74) | 27206.31 (19952.28 to 34739.88) | 27053.25 (19900.98 to 34491.32) | 26888.21 (19839.1 to 34216.56) | 26744.76 (19825.06 to 33930.51) |
| Male | 27817.05 (20234.01 to 35700.03) | 27739.62 (20231.83 to 35526.2) | 27623.36 (20175.34 to 35332.1) | 27495.42 (20079.8 to 35138.7) | 27379.33 (20004.69 to 34964.88) |
| Age* |  |  |  |  |  |
| 2-4 years | 52358.04 (39159.32 to 63273.97) | 52189.18 (39156.72 to 62998.12) | 51993.49 (39113.8 to 62748.6) | 51799.56 (39103.35 to 62552.71) | 51632.99 (39009.24 to 62418.49) |
| 5-9 years | 41363.29 (31732.12 to 52725.24) | 41257.02 (31759.39 to 52420.25) | 41058.92 (31696.63 to 52046.48) | 40824.34 (31599.9 to 51644.58) | 40611.74 (31527.49 to 51269.54) |
| 10-14 years | 5978.63 (2073.73 to 10412.12) | 5958.11 (2112.89 to 10377.44) | 5907.38 (2099.73 to 10300.38) | 5844.45 (2081.56 to 10198.12) | 5788.66 (2056.77 to 10089.55) |
| Sociodemographic index |  |  |  |  |  |
| Low SDI | 26880.89 (19314.53 to 34654.22) | 26762.06 (19559.79 to 34312.1) | 26502.95 (19627.42 to 33895.66) | 26194.04 (19551.86 to 33413.42) | 25933.16 (19444.18 to 32964.69) |
| Low-middle SDI | 27703.64 (19495.65 to 36257.43) | 27618.96 (19505.66 to 36059.33) | 27474.7 (19435.99 to 35799.79) | 27304.3 (19332.95 to 35565.83) | 27148.09 (19224.24 to 35356.7) |
| Middle SDI | 29273.32 (21879.39 to 37080.17) | 29250.86 (21911.13 to 37035.71) | 29239.7 (21948.7 to 37001.16) | 29248.01 (21997.4 to 36988.12) | 29257.6 (22047.27 to 36946.52) |
| High-middle SDI | 29276.43 (21378.29 to 37458.44) | 29186.33 (21354.95 to 37336.91) | 29125.09 (21355.24 to 37266.13) | 29104.67 (21390.64 to 37251.31) | 29102.94 (21441.93 to 37267.02) |
| High SDI | 20930.7 (15749.17 to 26478.6) | 20730.65 (15601.27 to 26288.81) | 20442.97 (15380.99 to 25988.25) | 20113.27 (15118.39 to 25651.45) | 19808.42 (14853.91 to 25334.15) |
| **Caries of permanent teeth** |  |  |  |  |  |
| Global | 29599.7 (20747.06 to 40789.8) | 29708.75 (20878.54 to 40883.95) | 29952.97 (21128.86 to 41088.2) | 30249.19 (21388.12 to 41359.75) | 30504.78 (21615.82 to 41619.1) |
| Sex |  |  |  |  |  |
| Female | 30000.49 (20977 to 41336.31) | 30107.44 (21109.63 to 41420.05) | 30352.33 (21371.26 to 41634.62) | 30649.46 (21658.4 to 41902.78) | 30903.77 (21886.55 to 42136.36) |
| Male | 29214.3 (20489.62 to 40259.53) | 29325.34 (20628.33 to 40357.34) | 29568.62 (20869.49 to 40561.61) | 29863.6 (21118.89 to 40854.14) | 30120.17 (21341.04 to 41139.73) |
| Age* |  |  |  |  |  |
| 5-9 years | 9069.71 (4748.12 to 14897.57) | 9149.38 (4794.72 to 14991.84) | 9266.77 (4857.35 to 15144.6) | 9393.37 (4922.76 to 15314.37) | 9496.27 (4985.31 to 15451.79) |
| 10-14 years | 25412.01 (17029.91 to 35738.13) | 25509.24 (17113.22 to 35851.09) | 25686.84 (17237.16 to 36070.25) | 25903.15 (17354.37 to 36367.67) | 26114.34 (17473.64 to 36677.43) |
| 15-19 years | 31122 (21770.34 to 42728.19) | 31226.53 (21869.82 to 42819.72) | 31469.53 (22127.57 to 43029.52) | 31756.4 (22294.26 to 43262.32) | 31993.16 (22477.63 to 43461.2) |
| 20-24 years | 39391.09 (29430.42 to 50777.12) | 39451.81 (29549.19 to 50863.11) | 39614.65 (29846.24 to 50878.53) | 39807.6 (30142.64 to 50909.68) | 39939 (30342.9 to 50950.82) |
| 25-29 years | 38023.48 (26865.58 to 53760.35) | 38250.82 (27184.24 to 53987.78) | 38638.68 (27656.87 to 54252.6) | 39071.23 (28166.91 to 54535.07) | 39426.36 (28572.56 to 54756.76) |
| 30-34 years | 34941.6 (25910 to 47895.01) | 35154.69 (26145.2 to 48028.84) | 35588.72 (26514.16 to 48426.52) | 36083.41 (26956.22 to 48975.51) | 36521.72 (27379.71 to 49511.95) |
| 35-39 years | 36026.46 (24771.54 to 48264.48) | 36010.27 (24820.53 to 48155.47) | 36252.87 (25066.04 to 48347.17) | 36659.22 (25381.76 to 48740.72) | 37047.43 (25663.24 to 49173.58) |
| Sociodemographic index |  |  |  |  |  |
| Low SDI | 33322.21 (24323.74 to 43724.78) | 33430.21 (24397.04 to 43858.91) | 33674.49 (24570.08 to 44146.49) | 33963.49 (24776.35 to 44476.75) | 34196.41 (24944.11 to 44764.68) |
| Low-middle SDI | 30886.74 (21346.2 to 42968.56) | 31142.61 (21561.79 to 43282.85) | 31679.94 (21999.07 to 43905.35) | 32301.62 (22472.14 to 44563.78) | 32812.99 (22873.89 to 45117.18) |
| Middle SDI | 29598.52 (19953.65 to 41661.73) | 29705.33 (20124.14 to 41743.91) | 29939.36 (20432.11 to 41954.5) | 30225.78 (20731.67 to 42221.54) | 30478.91 (20966.28 to 42438.54) |
| High-middle SDI | 28940.3 (19714.7 to 40309.54) | 28923.91 (19856.45 to 40175.39) | 28970.96 (20025.87 to 40083.64) | 29047.91 (20170.92 to 40053.52) | 29121.03 (20324.75 to 40077.85) |
| High SDI | 25655.89 (19319.21 to 33670.38) | 25565.35 (19264.44 to 33561.1) | 25427.45 (19183.41 to 33396.97) | 25273.96 (19074.72 to 33218.72) | 25132.95 (18938.33 to 33057.41) |
| **Periodontal diseases** |  |  |  |  |  |
| Global | 8531.49 (5926.6 to 11516.2) | 8552.65 (5972.85 to 11492.81) | 8558.72 (5986.18 to 11483.97) | 8564.9 (5994.2 to 11502.24) | 8580.5 (6000.64 to 11545.26) |
| Sex |  |  |  |  |  |
| Female | 8334.7 (5794.69 to 11229.95) | 8355.91 (5835.25 to 11210.22) | 8352.96 (5844.08 to 11206.62) | 8346.85 (5829.6 to 11207.82) | 8352.1 (5831.72 to 11214.51) |
| Male | 8725.2 (6063.01 to 11787.55) | 8746.55 (6101.57 to 11782.9) | 8761.68 (6124.66 to 11783.44) | 8780.1 (6134.63 to 11803.37) | 8805.98 (6155.88 to 11859.03) |
| Age* |  |  |  |  |  |
| 15-19 years | 1292.38 (714.15 to 2176.73) | 1287.96 (719.63 to 2153.28) | 1273.94 (722.82 to 2116.74) | 1258.87 (722.04 to 2078.96) | 1249.89 (718.9 to 2052.11) |
| 20-24 years | 4327.81 (2649.2 to 6597.98) | 4354.73 (2693.98 to 6592.38) | 4337.98 (2708.74 to 6542.11) | 4298.83 (2701.36 to 6445.27) | 4261.32 (2689.39 to 6374.66) |
| 25-29 years | 8495.39 (5670.86 to 11523.09) | 8589.37 (5780.75 to 11575.12) | 8632.82 (5835.15 to 11644.34) | 8644.56 (5857.87 to 11698.58) | 8649.9 (5885.02 to 11806.51) |
| 30-34 years | 12853.64 (9217.74 to 17114.98) | 12914.46 (9261.51 to 17104.64) | 12942.45 (9245.84 to 17079.46) | 12958.4 (9231.3 to 17143.31) | 12995.52 (9230.25 to 17220.53) |
| 35-39 years | 17527.97 (12754.2 to 22462.12) | 17450.08 (12783.52 to 22320.66) | 17441.54 (12792.93 to 22323.52) | 17508.66 (12837.12 to 22452.43) | 17603.11 (12861.21 to 22601.25) |
| Sociodemographic index |  |  |  |  |  |
| Low SDI | 14833.45 (10029.88 to 20018.33) | 14763.73 (10041.88 to 19862.15) | 14594.11 (9970.18 to 19580.85) | 14381.25 (9847.7 to 19261.29) | 14195.67 (9746.14 to 18975.68) |
| Low-middle SDI | 13067.57 (9077.97 to 17409.79) | 13060.26 (9142.15 to 17346.55) | 12984.07 (9151.27 to 17197.81) | 12885.18 (9135.71 to 17025.17) | 12809.89 (9117.91 to 16889.34) |
| Middle SDI | 7431.67 (4957.08 to 10339.84) | 7461.45 (5024.83 to 10346.37) | 7500.55 (5076.65 to 10378.75) | 7547.85 (5114.43 to 10434.88) | 7598.13 (5144.53 to 10479.6) |
| High-middle SDI | 4674.72 (3147.36 to 6588.52) | 4760.7 (3203.58 to 6670.98) | 4964.95 (3333.03 to 6960.81) | 5212.11 (3484.24 to 7308.36) | 5422.88 (3615.76 to 7612.43) |
| High SDI | 6033.81 (4380.56 to 7984.63) | 5870.58 (4233.75 to 7802.52) | 5558.52 (3981.3 to 7459.87) | 5203.09 (3688.5 to 7073.21) | 4905.55 (3441.38 to 6766.68) |
| **Edentulism** |  |  |  |  |  |
| Global | 631.12 (392.17 to 904.87) | 645.19 (405.68 to 920.07) | 680.31 (435.75 to 959.68) | 725.59 (472.65 to 1012.06) | 769.08 (506.84 to 1061.31) |
| Sex |  |  |  |  |  |
| Female | 695.47 (434.48 to 992.9) | 711.55 (450.69 to 1009.24) | 751.5 (486.05 to 1054.22) | 802.88 (529.23 to 1112.7) | 852.13 (570.76 to 1168.54) |
| Male | 567.74 (349.9 to 816.25) | 579.73 (360.94 to 829.23) | 609.99 (385.34 to 865.6) | 649.18 (416.61 to 913.37) | 686.93 (444.49 to 959.62) |
| Age* |  |  |  |  |  |
| 20-24 years | 161.29 (81.84 to 267.82) | 169.95 (89.31 to 277.4) | 188.74 (104.17 to 300.42) | 211.82 (121.79 to 328.75) | 233.07 (138.69 to 354.33) |
| 25-29 years | 419.09 (238.31 to 613.68) | 439.95 (255.92 to 641.21) | 479.68 (286.23 to 691.71) | 527.87 (323.14 to 751.21) | 573.04 (357.52 to 804.89) |
| 30-34 years | 748.49 (462.99 to 1060.67) | 772 (482.07 to 1088.88) | 819.4 (519.63 to 1143.85) | 877.07 (564.58 to 1212.37) | 932.43 (606.54 to 1275.68) |
| 35-39 years | 1277.8 (841.85 to 1789.4) | 1280.37 (851.71 to 1783.36) | 1315.85 (890.86 to 1813.34) | 1369.97 (941.19 to 1867.99) | 1424.25 (986.55 to 1924.29) |
| Sociodemographic index |  |  |  |  |  |
| Low SDI | 1091.01 (689.7 to 1529.45) | 1101.99 (702.02 to 1539.19) | 1125.08 (725.32 to 1561.85) | 1154.99 (752.74 to 1594.9) | 1186.06 (778.79 to 1631.66) |
| Low-middle SDI | 611.2 (371.21 to 882.27) | 629.92 (388.65 to 899.35) | 675.91 (428.51 to 949.32) | 734.81 (477.53 to 1015.27) | 792.6 (523.62 to 1083.27) |
| Middle SDI | 563.04 (346.64 to 810) | 583.33 (365.29 to 833.53) | 634.27 (404.88 to 896.43) | 699.11 (454.32 to 974.89) | 760.11 (502 to 1049.48) |
| High-middle SDI | 585.75 (366.19 to 832.31) | 582.34 (366.14 to 825.45) | 587.65 (375.31 to 824.56) | 599.11 (389.95 to 831.89) | 612.32 (404.49 to 841.99) |
| High SDI | 589.23 (351.87 to 881.07) | 605.37 (365.82 to 894.4) | 631.8 (386.55 to 920.37) | 660.83 (410.66 to 952.03) | 683.57 (428.36 to 975.81) |
| **Other oral disorders** |  |  |  |  |  |
| Global | 1605.22 (1413.83 to 1800.08) | 1604.64 (1413.48 to 1799.7) | 1603.73 (1412.8 to 1798.94) | 1602.73 (1411.95 to 1798.07) | 1601.84 (1411.21 to 1797.25) |
| Sex |  |  |  |  |  |
| Female | 1739.45 (1528.62 to 1961.88) | 1738.62 (1527.78 to 1960.5) | 1737.27 (1526.51 to 1958.44) | 1735.74 (1525.11 to 1956.22) | 1734.3 (1523.97 to 1954.19) |
| Male | 1474.76 (1296.88 to 1661.79) | 1474.38 (1296.42 to 1661.25) | 1473.87 (1295.86 to 1660.6) | 1473.4 (1295.36 to 1660.14) | 1473.04 (1295 to 1659.97) |
| Age* |  |  |  |  |  |
| 2-4 years | 1405.75 (1238.1 to 1594.74) | 1405.6 (1237.98 to 1594.65) | 1405.38 (1237.73 to 1594.54) | 1405.14 (1237.45 to 1594.45) | 1404.93 (1237.21 to 1594.36) |
| 5-9 years | 1029.72 (881.63 to 1180.21) | 1029.49 (881.33 to 1179.98) | 1029.17 (880.95 to 1179.62) | 1028.8 (880.56 to 1179.22) | 1028.48 (880.22 to 1178.86) |
| 10-14 years | 1087.9 (931.31 to 1249.76) | 1087.54 (930.93 to 1249.56) | 1086.97 (930.35 to 1249.19) | 1086.31 (929.7 to 1248.68) | 1085.66 (929.08 to 1248.16) |
| 15-19 years | 1627.81 (1429.87 to 1820.77) | 1627.5 (1429.6 to 1821.48) | 1626.95 (1429.19 to 1821.86) | 1626.31 (1428.64 to 1822.01) | 1625.72 (1428.05 to 1821.29) |
| 20-24 years | 1993.52 (1785.79 to 2194.11) | 1993.2 (1785.85 to 2194.07) | 1992.4 (1785.53 to 2193.61) | 1991.38 (1785.04 to 2193.07) | 1990.39 (1784.6 to 2193.08) |
| 25-29 years | 2179.06 (1926.35 to 2415.34) | 2178.74 (1926.8 to 2414.76) | 2177.95 (1926.91 to 2413.66) | 2176.87 (1926.79 to 2412.21) | 2175.73 (1926.64 to 2410.65) |
| 30-34 years | 2305.66 (2039.19 to 2573.08) | 2304.84 (2038.56 to 2573.23) | 2303.51 (2037.41 to 2572.69) | 2302.07 (2036.08 to 2571.88) | 2300.83 (2034.95 to 2571.13) |
| 35-39 years | 2374.55 (2116.24 to 2669.27) | 2373.62 (2115.89 to 2667.96) | 2371.82 (2114.3 to 2665.45) | 2369.7 (2111.63 to 2662.71) | 2367.79 (2109.23 to 2660.33) |
| Sociodemographic index |  |  |  |  |  |
| Low SDI | 1599.32 (1410.75 to 1792.95) | 1599.51 (1410.94 to 1793.19) | 1599.62 (1411.04 to 1793.34) | 1599.68 (1411.09 to 1793.43) | 1599.84 (1411.22 to 1793.62) |
| Low-middle SDI | 1604.19 (1413.24 to 1801.66) | 1604.29 (1413.32 to 1801.74) | 1604.26 (1413.29 to 1801.71) | 1604.13 (1413.16 to 1801.56) | 1603.96 (1413 to 1801.4) |
| Middle SDI | 1607.37 (1412.24 to 1812.5) | 1607.01 (1411.94 to 1812.07) | 1606.64 (1411.63 to 1811.61) | 1606.47 (1411.51 to 1811.38) | 1606.43 (1411.51 to 1811.32) |
| High-middle SDI | 1605.67 (1414.17 to 1801.26) | 1604.71 (1413.3 to 1800.08) | 1603.85 (1412.57 to 1799.06) | 1603.32 (1412.18 to 1798.47) | 1603 (1412.04 to 1798.07) |
| High SDI | 1610.88 (1423.99 to 1798.21) | 1608.14 (1422.13 to 1794.63) | 1603.16 (1418.97 to 1788.17) | 1596.91 (1414.3 to 1781.92) | 1590.62 (1409.28 to 1775.39) |
| **Characteristic** | 2005 | 2006 | 2007 | 2008 | 2009 |
| **Caries of deciduous teeth** |  |  |  |  |  |
| Global | 26985.47 (19896.15 to 34291.65) | 26894.83 (19953.35 to 34090.83) | 26761.04 (19957.46 to 33907.58) | 26623.73 (19935.32 to 33723.09) | 26520.28 (19925.21 to 33554.47) |
| Sex |  |  |  |  |  |
| Female | 26653.99 (19832.24 to 33720.85) | 26585.5 (19834.78 to 33616.69) | 26499.13 (19874.82 to 33520.56) | 26418.01 (19869.51 to 33408.69) | 26362.57 (19882.1 to 33338.9) |
| Male | 27294.78 (19987.06 to 34859.57) | 27183.54 (20048.83 to 34613.38) | 27005.59 (20072.24 to 34299.37) | 26815.91 (20011.6 to 34041.23) | 26667.71 (19967.29 to 33758.47) |
| Age* |  |  |  |  |  |
| 2-4 years | 51509.57 (38868.11 to 62311.76) | 51379.04 (38889.26 to 62195.13) | 51203.2 (38910.89 to 61909.88) | 51019.4 (38815.09 to 61605.02) | 50863.56 (38834.8 to 61397.71) |
| 5-9 years | 40482.1 (31540.1 to 50991.92) | 40390.43 (31621.53 to 50861.55) | 40266.24 (31690.43 to 50696.76) | 40131.55 (31711.79 to 50520.31) | 40007 (31744.32 to 50305.62) |
| 10-14 years | 5760.88 (2052 to 10022.24) | 5715.56 (2070.6 to 9905.61) | 5613.52 (2050.62 to 9741.41) | 5489.82 (1996.9 to 9559.99) | 5381.16 (1932.99 to 9408.41) |
| Sociodemographic index |  |  |  |  |  |
| Low SDI | 25813.74 (19446.66 to 32647.23) | 25733.1 (19498.27 to 32469.33) | 25564.84 (19460.96 to 32201.35) | 25362.01 (19327.22 to 31900.61) | 25180.35 (19209.37 to 31650.69) |
| Low-middle SDI | 27047.08 (19186.55 to 35199.53) | 26945.14 (19303.51 to 34886.95) | 26784.66 (19329.63 to 34608.19) | 26599.85 (19306.02 to 34324.84) | 26429.74 (19239.65 to 34039.51) |
| Middle SDI | 29237.07 (22068.61 to 36869.89) | 29176.58 (22074.31 to 36724.26) | 29099.07 (22052.81 to 36575.39) | 29035.33 (22048.47 to 36414.62) | 29008.25 (22095.92 to 36291.04) |
| High-middle SDI | 29082.29 (21476.95 to 37243.06) | 29008.32 (21453.64 to 37138.51) | 28894.19 (21385.49 to 36984.87) | 28802.52 (21325.21 to 36860.5) | 28791.59 (21321.22 to 36845) |
| High SDI | 19602.2 (14649.32 to 25095.98) | 19469.17 (14508.01 to 24955.43) | 19340.95 (14368.61 to 24841.58) | 19247.92 (14256.43 to 24778.17) | 19203.49 (14181.7 to 24747.33) |
| **Caries of permanent teeth** |  |  |  |  |  |
| Global | 30628.37 (21722.53 to 41761.11) | 30601.07 (21725.7 to 41718.49) | 30499.6 (21663.03 to 41588.63) | 30366.31 (21569.31 to 41418.96) | 30247.44 (21484.79 to 41285.93) |
| Sex |  |  |  |  |  |
| Female | 31022.09 (21994.24 to 42263.03) | 30985.84 (21990.5 to 42202.32) | 30873.19 (21912.81 to 42046.91) | 30728.06 (21813.61 to 41872.08) | 30598.32 (21724.65 to 41724.88) |
| Male | 30248.84 (21437.22 to 41304.33) | 30230.44 (21447.47 to 41278.22) | 30139.99 (21400.51 to 41162.85) | 30018.38 (21323.65 to 41003.33) | 29910.18 (21249.53 to 40874.73) |
| Age* |  |  |  |  |  |
| 5-9 years | 9538.67 (5025.28 to 15501.45) | 9489.92 (4983.95 to 15445.26) | 9369.99 (4892.06 to 15305.79) | 9219.55 (4791.77 to 15131.99) | 9077.9 (4696.95 to 14968.76) |
| 10-14 years | 26270.16 (17570.42 to 36934.7) | 26337.06 (17650.68 to 37070.78) | 26337.05 (17683.57 to 37088.3) | 26296.82 (17681.57 to 37069.82) | 26244.9 (17661.81 to 37055.15) |
| 15-19 years | 32094.51 (22519.84 to 43545.82) | 32090.68 (22536.24 to 43512.24) | 32073.57 (22499.47 to 43517.93) | 32064.29 (22461.41 to 43494.79) | 32078.68 (22491.99 to 43516.06) |
| 20-24 years | 39912.75 (30368.69 to 50868.12) | 39721.48 (30272.72 to 50567.07) | 39462.99 (30102.21 to 50181.29) | 39194.83 (29919.73 to 49789.78) | 38970.5 (29754.25 to 49462.54) |
| 25-29 years | 39591.89 (28773.5 to 54848.7) | 39547.99 (28793.2 to 54763.74) | 39385.74 (28715.89 to 54544.28) | 39164.02 (28579.03 to 54255.72) | 38939.13 (28443.92 to 53963.93) |
| 30-34 years | 36779.96 (27624.3 to 49883.71) | 36838.02 (27664.65 to 49901.65) | 36792.17 (27635.62 to 49800.61) | 36696.86 (27546.18 to 49687.4) | 36602.34 (27436.42 to 49605.91) |
| 35-39 years | 37250.53 (25795.58 to 49430.18) | 37215.34 (25799.13 to 49437.75) | 37091.22 (25723.87 to 49323.92) | 36920.76 (25595.82 to 49108.16) | 36796.43 (25476.66 to 49017.78) |
| Sociodemographic index |  |  |  |  |  |
| Low SDI | 34282.72 (24988.85 to 44878.51) | 34214.6 (24917.99 to 44851.6) | 34065.86 (24783.56 to 44750.93) | 33880.28 (24597.25 to 44612.6) | 33705.07 (24417.22 to 44484.89) |
| Low-middle SDI | 33022.47 (23017.55 to 45361.92) | 32956.22 (22990.77 to 45335.21) | 32799.93 (22871.66 to 45199.18) | 32601.44 (22724.75 to 44981.42) | 32407.18 (22576.26 to 44790.04) |
| Middle SDI | 30611.53 (21079.68 to 42539.76) | 30582.19 (21124.33 to 42412.83) | 30447.65 (21121.05 to 42147.39) | 30271.12 (21086.72 to 41836.63) | 30116.35 (21029.29 to 41569.68) |
| High-middle SDI | 29154.57 (20418.26 to 40099.88) | 29119.77 (20396.28 to 40049.5) | 29035.87 (20331.82 to 39986.09) | 28933.91 (20217.29 to 39959.48) | 28849.05 (20107.92 to 39943.28) |
| High SDI | 25039.52 (18846.18 to 32941.92) | 24978.06 (18788.5 to 32906.96) | 24938.12 (18744.85 to 32900.56) | 24914.23 (18714.75 to 32908.72) | 24904.46 (18687.91 to 32926.39) |
| **Periodontal diseases** |  |  |  |  |  |
| Global | 8620.07 (6023.05 to 11576.06) | 8609.79 (6039.39 to 11479.73) | 8510.56 (6000.13 to 11285.44) | 8382.21 (5924.42 to 11076.55) | 8287.1 (5864.93 to 10938.05) |
| Sex |  |  |  |  |  |
| Female | 8388.48 (5858.19 to 11231.98) | 8387.78 (5886.02 to 11153.29) | 8308.23 (5858.69 to 11002.08) | 8202.15 (5797.81 to 10837.4) | 8124.76 (5760.6 to 10712.17) |
| Male | 8848.74 (6199.87 to 11914.54) | 8828.92 (6193.22 to 11817.75) | 8710.2 (6133.73 to 11578.02) | 8559.83 (6046.75 to 11328.53) | 8447.21 (5976.35 to 11152.48) |
| Age* |  |  |  |  |  |
| 15-19 years | 1253.5 (722.97 to 2043.72) | 1258.31 (739.98 to 2012.43) | 1253.15 (748.43 to 1992.3) | 1246.52 (749.4 to 1967.25) | 1247.22 (757.64 to 1983.91) |
| 20-24 years | 4248.79 (2693.23 to 6337.34) | 4215.04 (2695.54 to 6184.23) | 4128.02 (2672.25 to 5963.41) | 4027.83 (2643.62 to 5756.88) | 3954.81 (2618.91 to 5604.51) |
| 25-29 years | 8692.48 (5917.74 to 11842.65) | 8701.14 (5993.25 to 11735.48) | 8609.34 (5946.1 to 11617.71) | 8472.79 (5870.81 to 11369.31) | 8350.97 (5783.16 to 11127.7) |
| 30-34 years | 13086.06 (9278.22 to 17324.94) | 13126.75 (9301.16 to 17295.77) | 13044.02 (9274.57 to 17053.9) | 12920.85 (9209.5 to 16831.68) | 12832.77 (9167.84 to 16802.23) |
| 35-39 years | 17687.88 (12888.24 to 22672.59) | 17610.37 (12846.89 to 22502.21) | 17357.05 (12726.66 to 22092.77) | 17052.34 (12492.22 to 21716.23) | 16837.82 (12322.94 to 21402.91) |
| Sociodemographic index |  |  |  |  |  |
| Low SDI | 14097.21 (9697.17 to 18805.95) | 13767.56 (9750.03 to 18067.31) | 13041.68 (9469.06 to 16997.31) | 12191.45 (8890.22 to 15883.35) | 11484.99 (8357.12 to 14940.82) |
| Low-middle SDI | 12803.72 (9147.74 to 16819.3) | 12815.73 (9229.37 to 16746.23) | 12782.89 (9265.34 to 16644.3) | 12735.9 (9256.44 to 16529.52) | 12706.52 (9258.23 to 16456.49) |
| Middle SDI | 7655.49 (5191.76 to 10522.68) | 7663.2 (5228.7 to 10497.65) | 7595.64 (5208.87 to 10358.64) | 7505.98 (5157.23 to 10207.1) | 7445.86 (5134.12 to 10076.27) |
| High-middle SDI | 5523.62 (3674.33 to 7765.76) | 5454.87 (3633.4 to 7654.54) | 5265.1 (3533.38 to 7474.38) | 5040.81 (3379.35 to 7177.16) | 4861.96 (3236.95 to 7048.1) |
| High SDI | 4761.73 (3321.32 to 6620.56) | 4782.85 (3314.74 to 6664.33) | 4893.3 (3369.46 to 6845.43) | 5034.44 (3447.51 to 7104.81) | 5153.03 (3499.98 to 7341.28) |
| **Edentulism** |  |  |  |  |  |
| Global | 799.09 (530.38 to 1097.58) | 830.98 (550.3 to 1142.03) | 880.76 (580.24 to 1212.37) | 935.88 (610.42 to 1293.2) | 983.46 (637.79 to 1365.8) |
| Sex |  |  |  |  |  |
| Female | 886.09 (599.12 to 1207.81) | 915.79 (619.7 to 1250.32) | 956.79 (641.98 to 1313.04) | 1001.02 (663.68 to 1378.83) | 1040.32 (683.78 to 1440.41) |
| Male | 713.02 (462.24 to 990.94) | 747.11 (484.06 to 1038.26) | 805.6 (520.13 to 1121.05) | 871.53 (559.64 to 1214.37) | 927.3 (593.41 to 1295.73) |
| Age* |  |  |  |  |  |
| 20-24 years | 246.44 (148.03 to 370.65) | 256.18 (152.92 to 385.64) | 269.14 (157.88 to 408.46) | 282.94 (162.58 to 433.76) | 295.2 (167.61 to 455.22) |
| 25-29 years | 603.78 (379.75 to 844.26) | 634.85 (398.37 to 889.22) | 681.45 (423.09 to 959.31) | 732.03 (449.07 to 1035.83) | 774.15 (471.59 to 1101.57) |
| 30-34 years | 972.85 (635.26 to 1325.28) | 1020.68 (664.58 to 1391.62) | 1095.1 (707.23 to 1499.93) | 1177.53 (753.29 to 1621.48) | 1247.75 (793.99 to 1732.89) |
| 35-39 years | 1461.31 (1022.22 to 1965.62) | 1502.33 (1050.71 to 2019.67) | 1571.29 (1101.36 to 2103.88) | 1649.41 (1148.29 to 2209.28) | 1719.13 (1192.28 to 2306.28) |
| Sociodemographic index |  |  |  |  |  |
| Low SDI | 1212.88 (797.51 to 1666.89) | 1274.46 (835.08 to 1757.66) | 1387.33 (901.25 to 1920.39) | 1513.25 (974.55 to 2104.92) | 1614.83 (1033.5 to 2258.78) |
| Low-middle SDI | 835.61 (556.21 to 1138.97) | 912.81 (600.36 to 1249.27) | 1051.62 (675.06 to 1460.36) | 1206.48 (759.83 to 1701.35) | 1332.96 (830.03 to 1898.46) |
| Middle SDI | 799.2 (532.3 to 1098.45) | 824.97 (554.12 to 1130.9) | 856.54 (577.37 to 1172.29) | 889.73 (599.42 to 1218) | 919.94 (619.43 to 1260.18) |
| High-middle SDI | 622.86 (414.81 to 853.19) | 628.52 (419.77 to 859.59) | 631.72 (421.19 to 865.48) | 633.95 (421.16 to 872.02) | 637.48 (422.58 to 880.03) |
| High SDI | 692.88 (434.57 to 985.29) | 677.84 (427.7 to 961.09) | 644.6 (408.05 to 915.16) | 606.24 (384.16 to 863.48) | 574.79 (364.56 to 821.02) |
| **Other oral disorders** |  |  |  |  |  |
| Global | 1601.17 (1410.66 to 1796.54) | 1600.63 (1410.2 to 1795.85) | 1600.12 (1409.73 to 1795.21) | 1599.81 (1409.41 to 1794.77) | 1599.85 (1409.39 to 1794.73) |
| Sex |  |  |  |  |  |
| Female | 1733.14 (1523.11 to 1952.68) | 1732.16 (1522.13 to 1951.45) | 1731.16 (1521.19 to 1950.28) | 1730.37 (1520.46 to 1949.46) | 1730.02 (1520.23 to 1949.12) |
| Male | 1472.86 (1294.76 to 1659.77) | 1472.79 (1294.74 to 1659.68) | 1472.82 (1294.88 to 1659.71) | 1473.03 (1294.99 to 1659.91) | 1473.5 (1295.28 to 1660.45) |
| Age* |  |  |  |  |  |
| 2-4 years | 1404.81 (1237.05 to 1594.18) | 1404.77 (1237.18 to 1593.94) | 1404.76 (1237.33 to 1593.44) | 1404.74 (1237.48 to 1593.31) | 1404.75 (1237.65 to 1593.44) |
| 5-9 years | 1028.28 (879.99 to 1178.65) | 1028.15 (880.04 to 1178.32) | 1027.98 (880.04 to 1177.97) | 1027.83 (880.03 to 1177.64) | 1027.72 (880.08 to 1177.36) |
| 10-14 years | 1085.16 (928.6 to 1247.74) | 1084.76 (928.29 to 1247.09) | 1084.38 (928 to 1246.49) | 1084.05 (927.82 to 1245.96) | 1083.79 (927.82 to 1245.52) |
| 15-19 years | 1625.31 (1427.51 to 1819.63) | 1624.98 (1427.24 to 1819.32) | 1624.58 (1426.8 to 1818.75) | 1624.16 (1426.28 to 1817.66) | 1623.78 (1425.77 to 1816.61) |
| 20-24 years | 1989.68 (1784.4 to 2193.84) | 1989.19 (1783.79 to 2193.4) | 1988.71 (1783.18 to 2193.32) | 1988.25 (1782.55 to 2193.33) | 1987.87 (1781.97 to 2193.18) |
| 25-29 years | 2174.8 (1926.68 to 2409.26) | 2174.13 (1925.56 to 2408.69) | 2173.62 (1924.47 to 2408.32) | 2173.3 (1923.45 to 2408.21) | 2173.24 (1922.53 to 2408.49) |
| 30-34 years | 2300.07 (2034.29 to 2570.82) | 2299.6 (2034.23 to 2569.53) | 2299.14 (2034.12 to 2568.3) | 2298.69 (2034 to 2567.13) | 2298.42 (2033.99 to 2566.15) |
| 35-39 years | 2366.5 (2107.47 to 2658.65) | 2365.63 (2106.79 to 2658) | 2364.95 (2106.26 to 2657.56) | 2364.53 (2105.94 to 2657.4) | 2364.41 (2105.93 to 2657.53) |
| Sociodemographic index |  |  |  |  |  |
| Low SDI | 1600.13 (1411.47 to 1793.97) | 1600.47 (1411.76 to 1794.37) | 1600.81 (1412.05 to 1794.77) | 1601.15 (1412.35 to 1795.17) | 1601.46 (1412.63 to 1795.51) |
| Low-middle SDI | 1603.89 (1412.92 to 1801.33) | 1603.93 (1412.94 to 1801.39) | 1604.04 (1413.02 to 1801.5) | 1604.12 (1413.07 to 1801.58) | 1604.15 (1413.09 to 1801.6) |
| Middle SDI | 1606.25 (1411.41 to 1811.11) | 1605.86 (1411.12 to 1810.69) | 1605.46 (1410.77 to 1810.27) | 1605.28 (1410.63 to 1810.12) | 1605.49 (1410.84 to 1810.35) |
| High-middle SDI | 1602.6 (1411.85 to 1797.53) | 1601.94 (1411.45 to 1796.8) | 1601.17 (1410.92 to 1795.88) | 1600.73 (1410.61 to 1795.29) | 1601.1 (1410.9 to 1795.64) |
| High SDI | 1585.81 (1405.63 to 1769.76) | 1582.23 (1402.22 to 1766.1) | 1578.78 (1399.04 to 1762.05) | 1576.2 (1395.93 to 1759.26) | 1575.11 (1394.72 to 1758.29) |
| **Characteristic** | 2010 | 2011 | 2012 | 2013 | 2014 |
| **Caries of deciduous teeth** |  |  |  |  |  |
| Global | 26483.33 (19935.65 to 33468.06) | 26491.41 (19953.76 to 33472.89) | 26477.52 (19954.09 to 33473.84) | 26439.9 (19957.28 to 33424.11) | 26415.91 (19967.39 to 33366.73) |
| Sex |  |  |  |  |  |
| Female | 26347.91 (19899.57 to 33306.47) | 26358.16 (19920.86 to 33343.42) | 26345.07 (19928.25 to 33336.66) | 26306.89 (19924.97 to 33230.76) | 26280.66 (19897 to 33191.03) |
| Male | 26609.99 (20012.38 to 33631.52) | 26616.13 (20026.84 to 33623.17) | 26601.53 (20023.13 to 33583.56) | 26564.45 (20010.45 to 33562.12) | 26542.55 (20016.14 to 33573.6) |
| Age* |  |  |  |  |  |
| 2-4 years | 50761.65 (38921.39 to 61188) | 50777.09 (38982.7 to 61206.31) | 50895.54 (39131.24 to 61304.46) | 51034.56 (39296.08 to 61401.11) | 51153.24 (39374.01 to 61505.01) |
| 5-9 years | 39913.18 (31705.85 to 50122.66) | 39834.8 (31627.08 to 50010.13) | 39751.38 (31536.56 to 49963.01) | 39675.71 (31518.79 to 49860.35) | 39616.92 (31508.56 to 49705.19) |
| 10-14 years | 5326.66 (1903.57 to 9329.65) | 5299.79 (1905.33 to 9281.19) | 5255.92 (1892.17 to 9212.35) | 5207.03 (1867.44 to 9139.65) | 5164.56 (1843.13 to 9077.58) |
| Sociodemographic index |  |  |  |  |  |
| Low SDI | 25078.84 (19122.3 to 31496.36) | 25063.45 (19100.62 to 31473.17) | 25086.14 (19102.3 to 31500.22) | 25123.35 (19110.48 to 31542.17) | 25152.12 (19098.21 to 31576.03) |
| Low-middle SDI | 26319.57 (19274.79 to 33906.41) | 26269.82 (19292.05 to 33814.64) | 26244.25 (19325.06 to 33726.25) | 26231.27 (19343.56 to 33667.57) | 26217.47 (19364.57 to 33650.37) |
| Middle SDI | 29038.13 (22172.34 to 36297.36) | 29086.97 (22232.05 to 36320.46) | 29055.5 (22225.21 to 36237.6) | 28953.71 (22166.89 to 36083.72) | 28881.52 (22128.07 to 35982.25) |
| High-middle SDI | 28898.47 (21410.5 to 36966.32) | 29030.88 (21545.36 to 37090.19) | 29005.58 (21569.57 to 36994.3) | 28851.52 (21510.35 to 36776.82) | 28746.09 (21467.97 to 36604.43) |
| High SDI | 19197.27 (14142.42 to 24765.48) | 19212.59 (14150.02 to 24820.64) | 19230.06 (14120.32 to 24905.78) | 19250.78 (14078.76 to 24974.18) | 19286.26 (14068.28 to 25061.35) |
| **Caries of permanent teeth** |  |  |  |  |  |
| Global | 30186.95 (21431.54 to 41225.73) | 30176.47 (21434.89 to 41233.1) | 30170.89 (21424.75 to 41259.38) | 30160.59 (21395.51 to 41294.75) | 30134.08 (21348.8 to 41316.45) |
| Sex |  |  |  |  |  |
| Female | 30529.97 (21675.94 to 41656.76) | 30510.3 (21660.32 to 41643.59) | 30492.18 (21633.1 to 41648.54) | 30470.73 (21602.83 to 41662.05) | 30438.89 (21548.39 to 41657.87) |
| Male | 29857.29 (21208.31 to 40828.84) | 29855.68 (21219.37 to 40857.82) | 29862.3 (21208.76 to 40913.75) | 29862.86 (21176.6 to 40960.23) | 29841.58 (21123.73 to 40973.34) |
| Age* |  |  |  |  |  |
| 5-9 years | 8979.39 (4630.89 to 14851.81) | 8896.75 (4579.33 to 14729.04) | 8795.33 (4512.6 to 14579.54) | 8695.32 (4444.85 to 14431.15) | 8616.69 (4390.58 to 14313.9) |
| 10-14 years | 26204.77 (17640.9 to 37060.05) | 26144.56 (17606.77 to 37043.36) | 26029.51 (17514.78 to 36984.53) | 25883.19 (17393.16 to 36890.43) | 25727.26 (17258.05 to 36762.83) |
| 15-19 years | 32127.29 (22550.45 to 43591.36) | 32211.16 (22564.1 to 43725.99) | 32299.61 (22634.55 to 43867.96) | 32354.04 (22667.06 to 43974.28) | 32345.85 (22648.55 to 43996.31) |
| 20-24 years | 38840.28 (29624.24 to 49257.74) | 38831.61 (29640.12 to 49315.49) | 38900.72 (29649.85 to 49496.23) | 39002.47 (29676.78 to 49737.94) | 39087.79 (29667.23 to 49979.06) |
| 25-29 years | 38770.52 (28314.71 to 53745.69) | 38667.32 (28247.16 to 53635.55) | 38603.35 (28198.86 to 53575.65) | 38570.74 (28161.67 to 53565.99) | 38552.19 (28140.55 to 53598.22) |
| 30-34 years | 36569.95 (27378.99 to 49591.51) | 36593.05 (27472.99 to 49630.02) | 36606.42 (27478.35 to 49676.8) | 36594.86 (27398.1 to 49718.56) | 36541.6 (27282.92 to 49724.05) |
| 35-39 years | 36800.22 (25439.97 to 49079.4) | 36907.74 (25525.12 to 49187.78) | 37027.08 (25608.16 to 49331.69) | 37142.3 (25679.46 to 49518.87) | 37229.83 (25728.18 to 49688.33) |
| Sociodemographic index |  |  |  |  |  |
| Low SDI | 33583.1 (24299.79 to 44380.02) | 33478.7 (24232.37 to 44270.89) | 33343.22 (24121.72 to 44118.45) | 33198 (23999.46 to 43956.85) | 33060.87 (23881.75 to 43812.57) |
| Low-middle SDI | 32262.08 (22470.83 to 44650.5) | 32126.77 (22343.77 to 44492.36) | 31946.09 (22190.36 to 44281.97) | 31740.6 (22037.5 to 44042.53) | 31530.51 (21897.51 to 43812.36) |
| Middle SDI | 30043.55 (21000.91 to 41433.39) | 30053.48 (20996.26 to 41459.23) | 30091.98 (20976.91 to 41551.81) | 30132.66 (20961.56 to 41666.37) | 30147.35 (20950.45 to 41758.81) |
| High-middle SDI | 28818.65 (20049.99 to 39958.84) | 28860.01 (20081.76 to 40027.65) | 28943.18 (20127.41 to 40150.6) | 29041.28 (20184.59 to 40322.6) | 29121.66 (20191.68 to 40448.08) |
| High SDI | 24905.42 (18674.02 to 32946.56) | 24975.81 (18697.08 to 33135.96) | 25125.59 (18758.34 to 33392.26) | 25304.82 (18828.07 to 33674.48) | 25457.9 (18879.49 to 33940.95) |
| **Periodontal diseases** |  |  |  |  |  |
| Global | 8291.37 (5888.02 to 10922.97) | 8417.21 (5999.34 to 11078.77) | 8605.25 (6149.15 to 11329.4) | 8805.71 (6301.72 to 11619.71) | 8974.43 (6430.24 to 11872.99) |
| Sex |  |  |  |  |  |
| Female | 8135.61 (5799.88 to 10713.7) | 8249.82 (5908.46 to 10858.39) | 8411.88 (6041.75 to 11104.18) | 8581.7 (6163.2 to 11367.4) | 8724.54 (6284.71 to 11582.33) |
| Male | 8445.04 (5980.19 to 11130.36) | 8582.41 (6101.62 to 11299.72) | 8796.08 (6257.11 to 11587.71) | 9026.69 (6428.27 to 11904.36) | 9220.72 (6573.52 to 12179.06) |
| Age* |  |  |  |  |  |
| 15-19 years | 1265 (773.46 to 2012.77) | 1303.59 (803.64 to 2060.13) | 1353.24 (837.46 to 2111.29) | 1403.93 (878.68 to 2164.56) | 1446.13 (917.89 to 2225.61) |
| 20-24 years | 3951.72 (2634 to 5567.73) | 4031.21 (2672.38 to 5653.4) | 4154.88 (2758.89 to 5765.34) | 4295.73 (2865.32 to 5977.95) | 4427.44 (2972.09 to 6138.89) |
| 25-29 years | 8307.15 (5752.79 to 11023.66) | 8348.87 (5735.27 to 11068.93) | 8433.27 (5825.46 to 11227.54) | 8543.62 (5904.9 to 11411.32) | 8653.23 (5965.8 to 11599.32) |
| 30-34 years | 12866.54 (9219.05 to 16889.35) | 13047.4 (9387.74 to 17076.7) | 13292.5 (9572.09 to 17381.94) | 13537.33 (9722.98 to 17714.78) | 13731.59 (9868.15 to 18014.58) |
| 35-39 years | 16855.9 (12393.3 to 21348.6) | 17175.16 (12768.19 to 21805.25) | 17658.77 (13160.36 to 22500.21) | 18161.77 (13583.95 to 23239.89) | 18564.72 (13903.04 to 23854.71) |
| Sociodemographic index |  |  |  |  |  |
| Low SDI | 11198.44 (8142.64 to 14546.16) | 11198.4 (8195.67 to 14511.32) | 11174.33 (8157.22 to 14480.97) | 11139.61 (8089.12 to 14481.26) | 11114.93 (8053.64 to 14501.24) |
| Low-middle SDI | 12726.71 (9298.89 to 16420.81) | 12789.83 (9415.88 to 16386.59) | 12858.19 (9462.22 to 16442.04) | 12919.53 (9504.62 to 16497.7) | 12962.86 (9582.43 to 16566.19) |
| Middle SDI | 7467.04 (5161.17 to 10055.87) | 7609.8 (5264.28 to 10204.09) | 7835.4 (5440.35 to 10505.54) | 8078.64 (5612.34 to 10837.15) | 8280.26 (5756.91 to 11084.93) |
| High-middle SDI | 4807.21 (3194.4 to 7047.54) | 4942.39 (3283.51 to 7227.1) | 5214.31 (3470.88 to 7599.88) | 5527.47 (3693.53 to 8032.09) | 5791.21 (3878.17 to 8399.52) |
| High SDI | 5205.33 (3515.46 to 7477.13) | 5266.15 (3536.34 to 7593.53) | 5397.26 (3599.28 to 7811.82) | 5554.81 (3681.05 to 8088.36) | 5691.63 (3750.09 to 8336.68) |
| **Edentulism** |  |  |  |  |  |
| Global | 1010.88 (656.25 to 1404.09) | 1027.29 (670.38 to 1423.68) | 1046.92 (686.81 to 1445) | 1064.86 (705.5 to 1466.84) | 1075.6 (717.64 to 1478.75) |
| Sex |  |  |  |  |  |
| Female | 1066.9 (700.38 to 1480.26) | 1091.26 (721.28 to 1508.58) | 1124.45 (748.67 to 1547.65) | 1159.51 (777.42 to 1591.74) | 1188.49 (800.18 to 1628.31) |
| Male | 955.52 (611.14 to 1342.68) | 964.02 (618.77 to 1348.87) | 970.24 (626.07 to 1350.21) | 971.29 (631.71 to 1346.12) | 964.07 (630.76 to 1332.57) |
| Age* |  |  |  |  |  |
| 20-24 years | 303.75 (172.66 to 467.16) | 311.12 (178.8 to 477.59) | 320.11 (183.74 to 490.31) | 329.2 (190.4 to 504.81) | 336.68 (194.61 to 515.89) |
| 25-29 years | 794.94 (485.46 to 1132.19) | 800.67 (491.65 to 1139.56) | 805.61 (497.92 to 1144.69) | 808.08 (502.12 to 1150.42) | 806.77 (504.83 to 1146.8) |
| 30-34 years | 1286.82 (818.23 to 1794.09) | 1307.8 (831.7 to 1819.74) | 1331.44 (851.95 to 1845.17) | 1350.61 (875.28 to 1866.9) | 1357.63 (889.49 to 1870.41) |
| 35-39 years | 1763 (1224.96 to 2358.63) | 1796.7 (1257.73 to 2395.8) | 1840.5 (1294.49 to 2440.53) | 1884.46 (1338.1 to 2488.8) | 1916.29 (1367.53 to 2527.93) |
| Sociodemographic index |  |  |  |  |  |
| Low SDI | 1656.09 (1056 to 2322.7) | 1648.34 (1050.46 to 2314.95) | 1628.72 (1037.26 to 2287.04) | 1601.03 (1019.03 to 2247.39) | 1569.04 (997.81 to 2199.31) |
| Low-middle SDI | 1388.3 (861.31 to 1987.62) | 1389.17 (869.58 to 1981.43) | 1380.66 (872.45 to 1966.68) | 1363.68 (867.58 to 1937.07) | 1339.05 (856.02 to 1897.07) |
| Middle SDI | 943.04 (637.98 to 1291.14) | 966.57 (656.75 to 1318.99) | 996.83 (682.9 to 1357.31) | 1027.11 (708.16 to 1395.5) | 1049.46 (728.92 to 1424.64) |
| High-middle SDI | 645.11 (428.63 to 891.42) | 657.59 (445.07 to 905.85) | 672.06 (461.25 to 919.26) | 686.63 (474.63 to 936.61) | 698.07 (487.19 to 949.8) |
| High SDI | 562.66 (358.45 to 802.85) | 586.09 (373.82 to 834.93) | 638.86 (406.54 to 907.45) | 702.1 (446.45 to 994.76) | 756.27 (481.67 to 1071.67) |
| **Other oral disorders** |  |  |  |  |  |
| Global | 1600.36 (1409.81 to 1795.21) | 1601.13 (1410.38 to 1795.88) | 1601.57 (1410.74 to 1796.19) | 1601.56 (1410.71 to 1796) | 1601.62 (1410.76 to 1795.9) |
| Sex |  |  |  |  |  |
| Female | 1730.28 (1520.47 to 1949.5) | 1730.94 (1521.33 to 1950.5) | 1731.32 (1522.03 to 1951.17) | 1731.31 (1522.44 to 1951.37) | 1731.42 (1522.87 to 1951.59) |
| Male | 1474.24 (1295.79 to 1661.38) | 1475.12 (1296.67 to 1662.39) | 1475.63 (1297.19 to 1662.91) | 1475.68 (1297.3 to 1662.9) | 1475.76 (1297.41 to 1663.04) |
| Age* |  |  |  |  |  |
| 2-4 years | 1404.77 (1237.84 to 1593.6) | 1404.86 (1238.03 to 1593.43) | 1405.04 (1238.26 to 1593.3) | 1405.25 (1238.5 to 1593.23) | 1405.42 (1238.69 to 1593.26) |
| 5-9 years | 1027.72 (880.21 to 1177.2) | 1027.8 (880.17 to 1177.46) | 1027.92 (880.15 to 1177.77) | 1028.07 (880.15 to 1178.13) | 1028.24 (880.16 to 1178.51) |
| 10-14 years | 1083.64 (927.88 to 1245.23) | 1083.69 (927.82 to 1245.27) | 1083.91 (927.9 to 1245.53) | 1084.23 (928.06 to 1245.96) | 1084.59 (928.28 to 1246.26) |
| 15-19 years | 1623.53 (1425.42 to 1815.72) | 1623.43 (1424.92 to 1815.82) | 1623.44 (1425.38 to 1816.04) | 1623.52 (1425.87 to 1816.28) | 1623.61 (1426.28 to 1816.52) |
| 20-24 years | 1987.68 (1781.57 to 2193.02) | 1987.65 (1781.48 to 2192.23) | 1987.7 (1781.39 to 2191.28) | 1987.79 (1781.31 to 2190.22) | 1987.88 (1781.22 to 2189.41) |
| 25-29 years | 2173.51 (1921.81 to 2409.19) | 2174.03 (1921.93 to 2410.01) | 2174.66 (1922.2 to 2410.83) | 2175.24 (1922.5 to 2411.51) | 2175.69 (1922.81 to 2411.9) |
| 30-34 years | 2298.46 (2034.21 to 2565.52) | 2298.81 (2034.7 to 2564.83) | 2299.34 (2035.04 to 2564.64) | 2299.96 (2035.58 to 2565.01) | 2300.56 (2036.26 to 2565.33) |
| 35-39 years | 2364.54 (2106.44 to 2657.9) | 2364.8 (2106.54 to 2657.75) | 2365.03 (2106.76 to 2657.75) | 2365.18 (2106.95 to 2657.67) | 2365.28 (2107.09 to 2657.52) |
| Sociodemographic index |  |  |  |  |  |
| Low SDI | 1601.75 (1412.9 to 1795.83) | 1602.07 (1413.19 to 1796.17) | 1602.39 (1413.49 to 1796.5) | 1602.7 (1413.78 to 1796.81) | 1602.96 (1414.02 to 1797.06) |
| Low-middle SDI | 1604.17 (1413.12 to 1801.6) | 1604.27 (1413.22 to 1801.69) | 1604.49 (1413.43 to 1801.93) | 1604.8 (1413.72 to 1802.23) | 1605.09 (1414.01 to 1802.52) |
| Middle SDI | 1606.28 (1411.52 to 1811.24) | 1607.34 (1412.41 to 1812.46) | 1607.52 (1412.55 to 1812.64) | 1606.67 (1411.8 to 1811.64) | 1606.01 (1411.2 to 1810.88) |
| High-middle SDI | 1602.58 (1412.09 to 1797.23) | 1604.46 (1413.55 to 1799.22) | 1604.69 (1413.53 to 1799.32) | 1603.13 (1411.99 to 1797.37) | 1601.83 (1410.79 to 1795.74) |
| High SDI | 1575.59 (1394.14 to 1759.24) | 1577.03 (1395.39 to 1760.73) | 1578.79 (1397.23 to 1762) | 1580.61 (1399.14 to 1763.88) | 1582.49 (1400.18 to 1766.87) |
| **Characteristic** | 2015 | 2016 | 2017 | 2018 | 2019 |
| **Caries of deciduous teeth** |  |  |  |  |  |
| Global | 26407.34 (19975.81 to 33338.65) | 26414.82 (19982.3 to 33371.02) | 26460.87 (19996.31 to 33465.43) | 26548.12 (20034.73 to 33541.8) | 26650.74 (20092.21 to 33667.05) |
| Sex |  |  |  |  |  |
| Female | 26270.04 (19888.66 to 33184.79) | 26273.92 (19865.29 to 33200.78) | 26312.29 (19863.6 to 33274.09) | 26389.47 (19925.34 to 33366.27) | 26482.34 (20012.35 to 33455.9) |
| Male | 26535.84 (20028.07 to 33587.38) | 26546.59 (20058.33 to 33593.27) | 26599.78 (20102.94 to 33653.68) | 26696.42 (20163.91 to 33748.9) | 26808.19 (20240.32 to 33876.13) |
| Age* |  |  |  |  |  |
| 2-4 years | 51205.06 (39399.77 to 61554.58) | 51216.81 (39400.76 to 61584.72) | 51251.27 (39463.45 to 61674.86) | 51305.76 (39543.22 to 61804.88) | 51374.65 (39639.61 to 61939.28) |
| 5-9 years | 39579.87 (31473.9 to 49616.33) | 39587.94 (31451.48 to 49701.53) | 39642.88 (31430.74 to 49871.37) | 39714.2 (31399.37 to 49913.54) | 39769.4 (31382.5 to 49971.17) |
| 10-14 years | 5139.13 (1834.19 to 9038.48) | 5115.66 (1844.57 to 8989.36) | 5080.27 (1835.45 to 8928.24) | 5046.78 (1820.18 to 8873.05) | 5028.7 (1813.44 to 8851.03) |
| Sociodemographic index |  |  |  |  |  |
| Low SDI | 25148.44 (19072.16 to 31579.03) | 25067.57 (18983.41 to 31487.57) | 24921.23 (18835.22 to 31324.69) | 24771.13 (18683.72 to 31156.73) | 24685.36 (18594.15 to 31101.63) |
| Low-middle SDI | 26192.66 (19382.06 to 33620.16) | 26209.51 (19402.25 to 33573.22) | 26291.78 (19483.87 to 33589.46) | 26387.35 (19535.44 to 33684.36) | 26433.78 (19615.1 to 33735.24) |
| Middle SDI | 28858.43 (22110.75 to 35953.16) | 28869.9 (22146.16 to 35968.3) | 28946.37 (22235.93 to 36027.4) | 29101.19 (22372.99 to 36194.25) | 29284.76 (22536.53 to 36408.51) |
| High-middle SDI | 28732.92 (21458.27 to 36550.93) | 28757.48 (21469.01 to 36576.96) | 28842.87 (21509.2 to 36698.54) | 29034.96 (21625.17 to 36924.62) | 29291.73 (21812.54 to 37220.83) |
| High SDI | 19330.04 (14078.74 to 25114.56) | 19436.99 (14151.29 to 25245.05) | 19661.5 (14295.14 to 25524.78) | 19988.42 (14569.29 to 25943.94) | 20389.74 (14868.12 to 26449.59) |
| **Caries of permanent teeth** |  |  |  |  |  |
| Global | 30083.37 (21286.22 to 41283.59) | 29908.88 (21143.41 to 41046.3) | 29602.78 (20903.41 to 40671.21) | 29298.81 (20656.35 to 40314.23) | 29135.32 (20507.35 to 40147.6) |
| Sex |  |  |  |  |  |
| Female | 30392.84 (21483.66 to 41625.47) | 30226.35 (21368.39 to 41414.41) | 29923.36 (21119.06 to 41063.35) | 29620.16 (20864.82 to 40727.52) | 29458.5 (20720.3 to 40567.45) |
| Male | 29786.42 (21055.2 to 40947.23) | 29604.26 (20922.8 to 40692.92) | 29295.26 (20702.68 to 40311.82) | 28990.66 (20457.34 to 39955.11) | 28825.49 (20306.1 to 39787.67) |
| Age* |  |  |  |  |  |
| 5-9 years | 8580.38 (4365.39 to 14259.22) | 8600.32 (4364.33 to 14333.21) | 8650.87 (4376.8 to 14452.45) | 8698.4 (4386.4 to 14564.9) | 8716.9 (4383.3 to 14629.67) |
| 10-14 years | 25582.56 (17125.45 to 36600.44) | 25349.84 (16916.99 to 36123.2) | 24997.1 (16614.24 to 35474.55) | 24643.3 (16260.97 to 34893.73) | 24418.87 (15991.09 to 34624.46) |
| 15-19 years | 32253.42 (22572.77 to 43854.01) | 31926.39 (22348.58 to 43479.48) | 31383.69 (21932.25 to 42831.07) | 30861.65 (21538.85 to 42208.92) | 30589.57 (21277.36 to 41860.94) |
| 20-24 years | 39111.96 (29617.47 to 50118.51) | 38918.62 (29424.08 to 49930.94) | 38511.94 (29100.94 to 49445.45) | 38100.92 (28803.1 to 48919.01) | 37905.53 (28656.64 to 48699.69) |
| 25-29 years | 38522.66 (28115.88 to 53620.13) | 38364.6 (28087.48 to 53411.18) | 38067.6 (27966.2 to 53153.46) | 37783.59 (27801.74 to 52908.21) | 37674.92 (27752.5 to 52808.88) |
| 30-34 years | 36437.19 (27131.89 to 49660.62) | 36170.8 (26853.7 to 49348.12) | 35760.25 (26497.09 to 48884.8) | 35371.22 (26199.67 to 48465.82) | 35161.73 (26084.34 to 48262.57) |
| 35-39 years | 37281.68 (25759 to 49764.79) | 37195.88 (25676.71 to 49562.22) | 36950.74 (25464.34 to 49270.27) | 36671.28 (25191.96 to 49001.92) | 36487.27 (24982.42 to 48888.01) |
| Sociodemographic index |  |  |  |  |  |
| Low SDI | 32946.82 (23774.09 to 43707.11) | 32767.25 (23651.15 to 43495.9) | 32487.21 (23441.16 to 43167.96) | 32203.22 (23198.76 to 42841.11) | 32010.1 (23001.64 to 42653.71) |
| Low-middle SDI | 31335.93 (21766.81 to 43583.41) | 31001.66 (21571.27 to 43232.81) | 30495.09 (21140.18 to 42618.95) | 30015.91 (20721.09 to 42059.4) | 29765.26 (20474.29 to 41789.1) |
| Middle SDI | 30116.87 (20917.8 to 41778.3) | 29924.45 (20780.05 to 41531.07) | 29566.71 (20522.95 to 41099.91) | 29202.9 (20280.16 to 40635.96) | 29000.74 (20162.65 to 40399.42) |
| High-middle SDI | 29150.3 (20149.46 to 40499.53) | 29058.36 (20063.7 to 40386.62) | 28862.18 (19927.29 to 40123.03) | 28660.12 (19803.8 to 39858.37) | 28554.49 (19719.61 to 39694.77) |
| High SDI | 25527.42 (18885.77 to 34101.47) | 25487.91 (18844.47 to 34054.33) | 25389.76 (18774.67 to 33939.68) | 25287.63 (18695.04 to 33814.96) | 25235.91 (18632.52 to 33734.82) |
| **Periodontal diseases** |  |  |  |  |  |
| Global | 9072.77 (6526.69 to 12030.68) | 9081.38 (6538.55 to 12024.53) | 9038.24 (6470.89 to 11970.64) | 8992.44 (6402.42 to 11970.83) | 8995.31 (6350.29 to 12007.81) |
| Sex |  |  |  |  |  |
| Female | 8811.8 (6332.84 to 11683.47) | 8840.03 (6319.57 to 11726.47) | 8841.55 (6308.76 to 11756.98) | 8842.81 (6279.68 to 11775.73) | 8871.3 (6263.61 to 11840.5) |
| Male | 9329.68 (6682.81 to 12339.67) | 9318.68 (6732.42 to 12318.1) | 9231.39 (6636.9 to 12206.55) | 9139.21 (6518.74 to 12136.83) | 9116.78 (6434.48 to 12176.16) |
| Age* |  |  |  |  |  |
| 15-19 years | 1469.87 (934.32 to 2270.08) | 1449.26 (920.45 to 2220.48) | 1389.02 (871.34 to 2091.91) | 1326.83 (830.47 to 1987.19) | 1300.2 (812.65 to 1945.91) |
| 20-24 years | 4523.56 (3061.96 to 6296.7) | 4518.06 (3076.07 to 6244.01) | 4416.28 (3009.1 to 6108.77) | 4301.67 (2924.04 to 6021.61) | 4264.41 (2839.01 to 6051.26) |
| 25-29 years | 8730.93 (6074.54 to 11684.23) | 8739.52 (6106.82 to 11659.68) | 8697.4 (6049.52 to 11657.15) | 8667.24 (5987.54 to 11840.9) | 8715.81 (5918.66 to 11923.69) |
| 30-34 years | 13823.5 (9944.55 to 18186.04) | 13800.32 (9912.07 to 18156.73) | 13728.54 (9819.37 to 18044.65) | 13662.06 (9743.52 to 18007.69) | 13650.44 (9676.97 to 18107.32) |
| 35-39 years | 18786 (14112.01 to 24215.91) | 18880.33 (14177.39 to 24360.92) | 18954.5 (14101.74 to 24495.98) | 19011.99 (14019.42 to 24559.05) | 19061.26 (13997.31 to 24581.31) |
| Sociodemographic index |  |  |  |  |  |
| Low SDI | 11120.82 (8088.21 to 14520.53) | 11115.6 (8094.11 to 14493.69) | 11069.98 (8071.13 to 14417.84) | 11019.37 (8031.1 to 14346.68) | 11001.2 (8016.6 to 14332.01) |
| Low-middle SDI | 12979.08 (9635.8 to 16586.23) | 12901.05 (9558.97 to 16460.2) | 12729.25 (9341.52 to 16298.23) | 12559.05 (9120.55 to 16208.2) | 12485.81 (8942.44 to 16296.96) |
| Middle SDI | 8386.77 (5856.8 to 11221.28) | 8384.81 (5862.74 to 11196.53) | 8327.65 (5857.86 to 11084.76) | 8267.15 (5837.91 to 10994.94) | 8256.25 (5842.57 to 10976.69) |
| High-middle SDI | 5919.93 (3967.76 to 8595.8) | 5936.67 (3978.73 to 8680.19) | 5936.18 (3931.22 to 8785.59) | 5934.28 (3901.87 to 8861.57) | 5945.07 (3887.97 to 8946.68) |
| High SDI | 5763.49 (3784.62 to 8474.57) | 5782.62 (3787.25 to 8507.04) | 5796.53 (3782.86 to 8541.62) | 5806.6 (3775.41 to 8580.81) | 5815.6 (3773.96 to 8623.02) |
| **Edentulism** |  |  |  |  |  |
| Global | 1073.79 (719.72 to 1475.02) | 1019.91 (691.45 to 1395.27) | 913.11 (623.05 to 1249.28) | 807.38 (549.54 to 1107.68) | 758.23 (515.71 to 1042.25) |
| Sex |  |  |  |  |  |
| Female | 1203.44 (812.43 to 1646.1) | 1149.59 (786.06 to 1564.9) | 1023.07 (704.51 to 1390.29) | 893.73 (616.99 to 1217.65) | 833.52 (575.39 to 1138.82) |
| Male | 945.85 (623.97 to 1306.08) | 892.1 (596.71 to 1229.61) | 804.87 (542 to 1108.59) | 722.49 (485.98 to 999.33) | 684.31 (460.28 to 949.11) |
| Age* |  |  |  |  |  |
| 20-24 years | 340.55 (196.28 to 522.85) | 318.68 (189.43 to 484.05) | 267.83 (159.15 to 405.03) | 215.64 (125.77 to 328.85) | 191.56 (110.81 to 294.11) |
| 25-29 years | 800.1 (502.4 to 1136.21) | 750.15 (476.98 to 1055.26) | 651.19 (416.36 to 910.48) | 552.81 (351.6 to 778.05) | 508.78 (322.04 to 717.15) |
| 30-34 years | 1344.95 (886.5 to 1846.39) | 1263.69 (840.1 to 1728.08) | 1118.04 (751.3 to 1523.37) | 978.58 (655.04 to 1331.92) | 913.4 (611.74 to 1239.49) |
| 35-39 years | 1925.06 (1380.55 to 2541.36) | 1859.61 (1344.07 to 2457.53) | 1721.82 (1245.35 to 2296.65) | 1582.92 (1140.63 to 2123.55) | 1516.61 (1090.65 to 2046.97) |
| Sociodemographic index |  |  |  |  |  |
| Low SDI | 1536.7 (976.59 to 2152.13) | 1453.06 (927.97 to 2035.55) | 1310.27 (838.95 to 1832.3) | 1175.75 (751.76 to 1644.07) | 1115.85 (712.26 to 1560.89) |
| Low-middle SDI | 1307.6 (838.29 to 1848.6) | 1199.15 (787.72 to 1669.42) | 1005.04 (671.57 to 1386.36) | 817.35 (546.63 to 1129.33) | 727.46 (484.56 to 1006.92) |
| Middle SDI | 1055.61 (735.86 to 1425.93) | 1005.6 (703.31 to 1356.84) | 900.53 (632.77 to 1214.04) | 795.21 (561.07 to 1075.56) | 746.44 (526.54 to 1011.44) |
| High-middle SDI | 704.13 (493.94 to 959.25) | 691.84 (488.49 to 942.41) | 661.29 (469.03 to 901.27) | 628.58 (448.27 to 853.34) | 611 (435.62 to 827.12) |
| High SDI | 781.04 (497.38 to 1108.89) | 767.41 (491.76 to 1091.21) | 731.57 (470.05 to 1048.26) | 694.9 (443.52 to 1005.36) | 679.38 (432.76 to 983.28) |
| **Other oral disorders** |  |  |  |  |  |
| Global | 1601.86 (1411.03 to 1796.04) | 1602.33 (1411.61 to 1796.39) | 1603.24 (1412.46 to 1797.31) | 1604.55 (1413.73 to 1798.7) | 1605.78 (1414.91 to 1800.04) |
| Sex |  |  |  |  |  |
| Female | 1731.78 (1523.4 to 1952.05) | 1732.56 (1523.83 to 1952.8) | 1733.98 (1524.75 to 1954.22) | 1735.79 (1525.88 to 1956.36) | 1737.27 (1526.67 to 1958.15) |
| Male | 1476 (1297.51 to 1663.41) | 1476.28 (1297.83 to 1663.59) | 1476.81 (1298.35 to 1664.19) | 1477.76 (1298.9 to 1665.25) | 1478.87 (1299.62 to 1666.48) |
| Age* |  |  |  |  |  |
| 2-4 years | 1405.47 (1238.79 to 1593.3) | 1405.44 (1238.57 to 1593.38) | 1405.39 (1238.34 to 1593.49) | 1405.34 (1238.11 to 1593.63) | 1405.29 (1237.88 to 1593.77) |
| 5-9 years | 1028.38 (880.16 to 1178.86) | 1028.61 (880.5 to 1179.24) | 1028.96 (880.95 to 1179.71) | 1029.28 (881.36 to 1180.14) | 1029.41 (881.6 to 1180.37) |
| 10-14 years | 1084.92 (928.57 to 1246.52) | 1085.31 (929.07 to 1247.05) | 1085.78 (929.67 to 1247.67) | 1086.21 (930.24 to 1248.11) | 1086.42 (930.6 to 1248.33) |
| 15-19 years | 1623.67 (1426.62 to 1816.75) | 1623.83 (1426.93 to 1817.03) | 1624.15 (1427.38 to 1817.41) | 1624.47 (1427.75 to 1817.55) | 1624.6 (1427.89 to 1817.51) |
| 20-24 years | 1987.93 (1781.14 to 2188.87) | 1988.03 (1781.19 to 2188.18) | 1988.22 (1781.33 to 2187.75) | 1988.39 (1781.46 to 2187.43) | 1988.42 (1781.45 to 2186.96) |
| 25-29 years | 2175.91 (1923.08 to 2411.89) | 2176.01 (1924.05 to 2410.78) | 2176.07 (1925.14 to 2410.14) | 2176.03 (1926.23 to 2409.71) | 2175.85 (1927.14 to 2409.24) |
| 30-34 years | 2301.06 (2037.01 to 2565.54) | 2301.7 (2038.64 to 2566.35) | 2302.52 (2039.3 to 2567.27) | 2303.16 (2039.3 to 2568.07) | 2303.29 (2038.81 to 2568.46) |
| 35-39 years | 2365.31 (2107.16 to 2657.27) | 2366.05 (2106.9 to 2658.14) | 2367.76 (2107.35 to 2660.1) | 2369.47 (2108.96 to 2662.08) | 2370.23 (2109.85 to 2663.04) |
| Sociodemographic index |  |  |  |  |  |
| Low SDI | 1603.19 (1414.23 to 1797.27) | 1603.37 (1414.39 to 1797.44) | 1603.49 (1414.49 to 1797.54) | 1603.52 (1414.53 to 1797.55) | 1603.52 (1414.51 to 1797.52) |
| Low-middle SDI | 1605.35 (1414.26 to 1802.76) | 1605.55 (1414.45 to 1802.94) | 1605.73 (1414.61 to 1803.09) | 1605.95 (1414.81 to 1803.33) | 1606.23 (1415.05 to 1803.65) |
| Middle SDI | 1605.88 (1411.09 to 1810.75) | 1605.97 (1411.19 to 1810.88) | 1606.6 (1411.78 to 1811.55) | 1608.13 (1413.17 to 1813.27) | 1610.07 (1414.92 to 1815.44) |
| High-middle SDI | 1601.48 (1410.49 to 1795.22) | 1601.55 (1410.58 to 1795.21) | 1602.54 (1411.41 to 1796.29) | 1604.99 (1413.53 to 1798.97) | 1608.06 (1416.21 to 1802.41) |
| High SDI | 1584.04 (1400.56 to 1769.47) | 1586.8 (1404.9 to 1771.73) | 1591.45 (1410.19 to 1776.4) | 1596.12 (1415.48 to 1782.83) | 1598.68 (1418.34 to 1786.1) |
| **Characteristic** | 2020 | 2021 |  |  |  |
| **Caries of deciduous teeth** |  |  |  |  |  |
| Global | 26861.68 (20506.69 to 33707.69) | 26566.05 (20396.01 to 33066.03) |  |  |  |
| Sex |  |  |  |  |  |
| Female | 26631.65 (20387.65 to 33256.66) | 26410.87 (20324.99 to 32874.05) |  |  |  |
| Male | 27076.99 (20493.01 to 34118.34) | 26711.32 (20443.09 to 33288.97) |  |  |  |
| Age* |  |  |  |  |  |
| 2-4 years | 51990.99 (40256.44 to 62424.79) | 51568.54 (40017.45 to 61973.34) |  |  |  |
| 5-9 years | 39965.7 (31731.97 to 49853.98) | 39735.11 (31639.6 to 48980.08) |  |  |  |
| 10-14 years | 4910.57 (1827.64 to 8611.31) | 4592.45 (1862.11 to 7990.86) |  |  |  |
| Sociodemographic index |  |  |  |  |  |
| Low SDI | 24777.06 (18692.95 to 31175.84) | 24661.72 (18732.19 to 30991.19) |  |  |  |
| Low-middle SDI | 26433.98 (19846.96 to 33342.63) | 25687.6 (19450.81 to 32236.46) |  |  |  |
| Middle SDI | 29482.82 (22769.68 to 36626.98) | 29422.9 (22821.37 to 36493.35) |  |  |  |
| High-middle SDI | 29413.98 (21832.91 to 37397.6) | 29252.41 (21741.01 to 37275.82) |  |  |  |
| High SDI | 22001.1 (16268.05 to 28230.91) | 22040.88 (16369.35 to 28331.23) |  |  |  |
| **Caries of permanent teeth** |  |  |  |  |  |
| Global | 29125.31 (20458.32 to 40094.99) | 28989.1 (20361.45 to 39942.86) |  |  |  |
| Sex |  |  |  |  |  |
| Female | 29417.95 (20630.07 to 40523.33) | 29287.66 (20536.18 to 40356.46) |  |  |  |
| Male | 28845.47 (20275.53 to 39758.9) | 28703.45 (20171.14 to 39607.33) |  |  |  |
| Age* |  |  |  |  |  |
| 5-9 years | 8657.57 (4328.71 to 14443.33) | 8060.5 (4034.26 to 13424.05) |  |  |  |
| 10-14 years | 24402.89 (15939.75 to 34514.14) | 23989.37 (15620.64 to 34167.91) |  |  |  |
| 15-19 years | 30571.41 (21324.37 to 41723.14) | 30718.27 (21337.49 to 41982.12) |  |  |  |
| 20-24 years | 37912.8 (28650.91 to 48786.89) | 38039.95 (28656.54 to 48896.86) |  |  |  |
| 25-29 years | 37778.44 (27692.99 to 53062.86) | 37842.44 (27751.75 to 53114.74) |  |  |  |
| 30-34 years | 35199.11 (25992.13 to 48233.14) | 35193.22 (26021.33 to 48401.82) |  |  |  |
| 35-39 years | 36368.02 (24836.17 to 48678.49) | 36242.12 (24752.67 to 48639.22) |  |  |  |
| Sociodemographic index |  |  |  |  |  |
| Low SDI | 31840.63 (22779.65 to 42571.76) | 31978.8 (22992.58 to 42807.2) |  |  |  |
| Low-middle SDI | 29813.82 (20519.56 to 41796.12) | 29642.63 (20375.11 to 41497.03) |  |  |  |
| Middle SDI | 28920.79 (19924.02 to 40470.64) | 28617.76 (19748.8 to 39931.58) |  |  |  |
| High-middle SDI | 28664.36 (19778.28 to 39817.71) | 28300.95 (19507.51 to 39447.72) |  |  |  |
| High SDI | 25208.86 (18572.65 to 33710.68) | 25307.91 (18681.66 to 33819.14) |  |  |  |
| **Periodontal diseases** |  |  |  |  |  |
| Global | 9035.57 (6363.88 to 12124.61) | 9063.06 (6368.42 to 12231.02) |  |  |  |
| Sex |  |  |  |  |  |
| Female | 8911.3 (6274.96 to 11972.11) | 8941.8 (6281.44 to 12097.97) |  |  |  |
| Male | 9157.12 (6449.18 to 12263.84) | 9181.54 (6450.99 to 12365.04) |  |  |  |
| Age* |  |  |  |  |  |
| 15-19 years | 1306.75 (818.42 to 1965.58) | 1305.39 (807.67 to 1967.64) |  |  |  |
| 20-24 years | 4299.72 (2862.75 to 6141.8) | 4309.53 (2879.48 to 6191.41) |  |  |  |
| 25-29 years | 8829.1 (5985.93 to 12190.85) | 8912.1 (6035.08 to 12376.57) |  |  |  |
| 30-34 years | 13687.77 (9658.53 to 18202.14) | 13751.46 (9691.44 to 18498.31) |  |  |  |
| 35-39 years | 19070.03 (13983.44 to 24702.46) | 19052.24 (13912.6 to 24708.77) |  |  |  |
| Sociodemographic index |  |  |  |  |  |
| Low SDI | 11025.39 (8067.17 to 14348.57) | 11025.85 (8066.72 to 14344.74) |  |  |  |
| Low-middle SDI | 12518.23 (8979 to 16344.32) | 12497.28 (8910.99 to 16380.95) |  |  |  |
| Middle SDI | 8265.48 (5862.96 to 10990.66) | 8267.89 (5825.4 to 11042.38) |  |  |  |
| High-middle SDI | 5939.23 (3843.29 to 9103.65) | 5971.04 (3876.57 to 9118.35) |  |  |  |
| High SDI | 5805.24 (3710.69 to 8690.97) | 5787.48 (3656.09 to 8799.97) |  |  |  |
| **Edentulism** |  |  |  |  |  |
| Global | 758.89 (518.98 to 1041.27) | 752.26 (522.08 to 1027.41) |  |  |  |
| Sex |  |  |  |  |  |
| Female | 834.79 (578.37 to 1140.46) | 827.03 (579.4 to 1124.99) |  |  |  |
| Male | 684.49 (462.61 to 945.55) | 679.06 (462.67 to 933.93) |  |  |  |
| Age* |  |  |  |  |  |
| 20-24 years | 192.63 (112.75 to 293.93) | 191.05 (114.83 to 288.26) |  |  |  |
| 25-29 years | 513.01 (325.97 to 720.38) | 510.61 (329.65 to 711.06) |  |  |  |
| 30-34 years | 912.51 (617.19 to 1238.76) | 907 (628.37 to 1226.27) |  |  |  |
| 35-39 years | 1514.52 (1092.4 to 2040.1) | 1496.2 (1087.35 to 2010.4) |  |  |  |
| Sociodemographic index |  |  |  |  |  |
| Low SDI | 1117.09 (720.83 to 1561.27) | 1090.35 (710.91 to 1512.41) |  |  |  |
| Low-middle SDI | 721.17 (486.36 to 992.25) | 709.41 (485.44 to 971.87) |  |  |  |
| Middle SDI | 747.99 (529.13 to 1011.07) | 741.41 (524.56 to 998.97) |  |  |  |
| High-middle SDI | 608.48 (433.47 to 825.71) | 607.5 (433.44 to 824.14) |  |  |  |
| High SDI | 683.11 (436.17 to 989.13) | 686.84 (440.25 to 992.86) |  |  |  |
| **Other oral disorders** |  |  |  |  |  |
| Global | 1606.55 (1415.62 to 1800.9) | 1606.79 (1415.88 to 1801.16) |  |  |  |
| Sex |  |  |  |  |  |
| Female | 1738.08 (1527.34 to 1959.07) | 1738.33 (1527.55 to 1959.37) |  |  |  |
| Male | 1479.73 (1300.36 to 1667.51) | 1480.07 (1300.59 to 1667.93) |  |  |  |
| Age* |  |  |  |  |  |
| 2-4 years | 1405.3 (1237.86 to 1593.68) | 1405.38 (1237.97 to 1593.52) |  |  |  |
| 5-9 years | 1029.43 (881.59 to 1180.37) | 1029.44 (881.59 to 1180.36) |  |  |  |
| 10-14 years | 1086.44 (930.61 to 1248.31) | 1086.42 (930.6 to 1248.23) |  |  |  |
| 15-19 years | 1624.59 (1427.87 to 1817.6) | 1624.59 (1427.86 to 1817.72) |  |  |  |
| 20-24 years | 1988.34 (1781.32 to 2186.61) | 1988.26 (1781.19 to 2186.28) |  |  |  |
| 25-29 years | 2175.6 (1927.1 to 2408.92) | 2175.39 (1927.01 to 2408.64) |  |  |  |
| 30-34 years | 2303.05 (2038.54 to 2568.26) | 2302.8 (2038.31 to 2568.17) |  |  |  |
| 35-39 years | 2370.23 (2110.15 to 2663.18) | 2370.25 (2110.51 to 2663.34) |  |  |  |
| Sociodemographic index |  |  |  |  |  |
| Low SDI | 1603.54 (1414.52 to 1797.53) | 1603.58 (1414.53 to 1797.57) |  |  |  |
| Low-middle SDI | 1606.46 (1415.24 to 1803.91) | 1606.57 (1415.31 to 1803.98) |  |  |  |
| Middle SDI | 1611.61 (1416.31 to 1817.18) | 1612.28 (1416.89 to 1817.95) |  |  |  |
| High-middle SDI | 1610.45 (1418.33 to 1805.2) | 1611.41 (1419.25 to 1806.42) |  |  |  |
| High SDI | 1599.23 (1418.72 to 1786.72) | 1599.12 (1418.51 to 1786.55) |  |  |  |

^*^Rate for age groups.

## Table E. The age-standardized YLD rates per 100,000 populations of oral disorders across different sexes, age groups, and SDI quintiles, 1990-2021

| **Characteristic** | 1990 | 1991 | 1992 | 1993 | 1994 |
| --- | --- | --- | --- | --- | --- |
| **Caries of deciduous teeth** |  |  |  |  |  |
| Global | 10.74 (4.39 to 21.27) | 10.73 (4.39 to 21.21) | 10.71 (4.39 to 21.17) | 10.69 (4.39 to 21.09) | 10.67 (4.39 to 21.05) |
| Sex |  |  |  |  |  |
| Female | 10.68 (4.39 to 21.04) | 10.67 (4.4 to 21.06) | 10.65 (4.39 to 21.06) | 10.64 (4.38 to 21) | 10.61 (4.37 to 20.93) |
| Male | 10.8 (4.39 to 21.55) | 10.78 (4.4 to 21.45) | 10.77 (4.4 to 21.29) | 10.75 (4.4 to 21.22) | 10.72 (4.41 to 21.17) |
| Age* |  |  |  |  |  |
| 2-4 years | 20.76 (8.75 to 41.28) | 20.68 (8.75 to 41.21) | 20.6 (8.74 to 41.17) | 20.52 (8.74 to 41.04) | 20.43 (8.73 to 40.92) |
| 5-9 years | 15.89 (6.69 to 30.49) | 15.89 (6.68 to 30.34) | 15.88 (6.67 to 30.36) | 15.88 (6.65 to 30.19) | 15.87 (6.67 to 30.19) |
| 10-14 years | 2.32 (0.58 to 5.52) | 2.31 (0.59 to 5.47) | 2.31 (0.59 to 5.44) | 2.3 (0.59 to 5.39) | 2.29 (0.57 to 5.38) |
| Sociodemographic index |  |  |  |  |  |
| Low SDI | 10.28 (4.23 to 20.2) | 10.27 (4.24 to 20.27) | 10.26 (4.27 to 20.23) | 10.25 (4.26 to 20.17) | 10.25 (4.26 to 20.24) |
| Low-middle SDI | 10.61 (4.31 to 21.06) | 10.62 (4.34 to 21.1) | 10.63 (4.34 to 21.05) | 10.64 (4.35 to 21.01) | 10.65 (4.36 to 21.12) |
| Middle SDI | 11.28 (4.61 to 22.43) | 11.27 (4.63 to 22.31) | 11.26 (4.66 to 22.39) | 11.24 (4.65 to 22.27) | 11.22 (4.65 to 22.2) |
| High-middle SDI | 11.59 (4.8 to 23.07) | 11.54 (4.76 to 22.97) | 11.5 (4.73 to 22.83) | 11.45 (4.72 to 22.63) | 11.38 (4.69 to 22.41) |
| High SDI | 8.77 (3.66 to 17.41) | 8.72 (3.64 to 17.38) | 8.68 (3.6 to 17.3) | 8.65 (3.61 to 17.21) | 8.61 (3.61 to 17.11) |
| **Caries of permanent teeth** |  |  |  |  |  |
| Global | 29.64 (11.61 to 59.77) | 29.62 (11.64 to 59.64) | 29.61 (11.68 to 59.53) | 29.6 (11.7 to 59.45) | 29.59 (11.72 to 59.34) |
| Sex |  |  |  |  |  |
| Female | 29.95 (11.79 to 60.26) | 29.94 (11.82 to 60.24) | 29.94 (11.85 to 60.06) | 29.93 (11.86 to 60.08) | 29.92 (11.86 to 60.04) |
| Male | 29.34 (11.43 to 59.31) | 29.31 (11.46 to 59.09) | 29.3 (11.52 to 59) | 29.29 (11.53 to 58.88) | 29.27 (11.59 to 58.65) |
| Age* |  |  |  |  |  |
| 5-9 years | 9.57 (3.14 to 22.51) | 9.49 (3.13 to 22.17) | 9.41 (3.13 to 21.96) | 9.34 (3.11 to 21.79) | 9.28 (3.11 to 21.47) |
| 10-14 years | 26.35 (10.32 to 56.21) | 26.36 (10.35 to 55.78) | 26.36 (10.34 to 55.26) | 26.33 (10.33 to 54.83) | 26.26 (10.37 to 54.44) |
| 15-19 years | 30.8 (11.58 to 64.65) | 30.88 (11.65 to 64.48) | 30.98 (11.74 to 64.68) | 31.08 (11.83 to 64.56) | 31.16 (11.89 to 64.61) |
| 20-24 years | 39.41 (14.98 to 75.82) | 39.34 (15.03 to 76.08) | 39.28 (15.11 to 76.49) | 39.24 (15.11 to 76.43) | 39.22 (15.11 to 76.63) |
| 25-29 years | 37.67 (15.23 to 72.53) | 37.5 (15.24 to 72.01) | 37.37 (15.29 to 71.6) | 37.31 (15.33 to 71.27) | 37.29 (15.39 to 71.33) |
| 30-34 years | 35.03 (14.53 to 67.89) | 35.13 (14.58 to 68.16) | 35.11 (14.61 to 67.94) | 34.98 (14.55 to 67.83) | 34.79 (14.46 to 67.52) |
| 35-39 years | 35.05 (14.34 to 69.98) | 35.1 (14.32 to 70.15) | 35.22 (14.42 to 70.25) | 35.42 (14.47 to 71.11) | 35.65 (14.58 to 71.1) |
| Sociodemographic index |  |  |  |  |  |
| Low SDI | 32.97 (13.36 to 65.7) | 33.08 (13.42 to 65.92) | 33.17 (13.55 to 65.9) | 33.25 (13.56 to 65.68) | 33.3 (13.63 to 65.72) |
| Low-middle SDI | 31.14 (12.14 to 62.87) | 31.02 (12.09 to 62.71) | 30.91 (12.07 to 62.47) | 30.81 (12.04 to 62.39) | 30.74 (12.08 to 62.19) |
| Middle SDI | 29.16 (11.26 to 59.68) | 29.19 (11.25 to 59.65) | 29.22 (11.31 to 59.55) | 29.25 (11.34 to 59.49) | 29.27 (11.36 to 59.42) |
| High-middle SDI | 29.79 (11.45 to 60.8) | 29.79 (11.52 to 60.46) | 29.78 (11.57 to 60.3) | 29.75 (11.64 to 60.16) | 29.69 (11.67 to 59.68) |
| High SDI | 26.84 (10.72 to 53.76) | 26.69 (10.74 to 53.26) | 26.54 (10.76 to 52.79) | 26.39 (10.75 to 52.62) | 26.27 (10.73 to 52.34) |
| **Periodontal diseases** |  |  |  |  |  |
| Global | 55.21 (20.29 to 119.47) | 55.01 (20.31 to 119.03) | 54.88 (20.35 to 118.79) | 54.82 (20.43 to 118.47) | 54.88 (20.51 to 118.19) |
| Sex |  |  |  |  |  |
| Female | 53.33 (19.66 to 115.28) | 53.17 (19.72 to 114.93) | 53.07 (19.77 to 114.5) | 53.06 (19.81 to 114.13) | 53.16 (19.92 to 113.87) |
| Male | 57.04 (20.91 to 123.58) | 56.81 (20.89 to 123.02) | 56.63 (20.9 to 122.76) | 56.55 (20.99 to 122.55) | 56.56 (21.08 to 122.4) |
| Age* |  |  |  |  |  |
| 15-19 years | 7.54 (2.13 to 18.97) | 7.69 (2.18 to 19.32) | 7.84 (2.24 to 19.64) | 8 (2.28 to 19.96) | 8.15 (2.34 to 20.23) |
| 20-24 years | 24.81 (8.74 to 58.86) | 25.07 (8.89 to 59.56) | 25.38 (9.04 to 60.27) | 25.72 (9.17 to 61.02) | 26.08 (9.29 to 61.91) |
| 25-29 years | 52.4 (19.22 to 115.29) | 52.28 (19.22 to 114.27) | 52.28 (19.39 to 113.92) | 52.47 (19.72 to 113.93) | 52.79 (19.9 to 113.82) |
| 30-34 years | 84.77 (29.66 to 182.4) | 85.05 (29.87 to 183.1) | 85.08 (29.92 to 182.98) | 84.69 (29.84 to 181.92) | 84.04 (29.63 to 179.96) |
| 35-39 years | 119.22 (46.7 to 247.91) | 117.51 (46.32 to 244.64) | 116.17 (46.06 to 242.58) | 115.51 (46.02 to 240.68) | 115.58 (46.31 to 240.05) |
| Sociodemographic index |  |  |  |  |  |
| Low SDI | 100.76 (37.19 to 215.53) | 101.05 (37.34 to 215.93) | 101.28 (37.45 to 216.15) | 101.37 (37.54 to 216.24) | 101.33 (37.59 to 215.76) |
| Low-middle SDI | 79.95 (29.39 to 171.63) | 80.96 (29.89 to 173.3) | 81.88 (30.27 to 175.04) | 82.7 (30.69 to 176.57) | 83.36 (31.05 to 177.49) |
| Middle SDI | 49.7 (18.02 to 106.98) | 48.86 (17.78 to 105.42) | 48.13 (17.58 to 103.94) | 47.59 (17.46 to 102.56) | 47.32 (17.48 to 101.73) |
| High-middle SDI | 37.56 (13.3 to 84.26) | 36.1 (12.79 to 80.68) | 34.7 (12.3 to 77.27) | 33.5 (11.89 to 74.43) | 32.59 (11.6 to 72.18) |
| High SDI | 39.11 (14.35 to 84.61) | 39.63 (14.62 to 85.42) | 40.14 (14.87 to 85.74) | 40.57 (15.1 to 86.07) | 40.86 (15.25 to 86.35) |
| **Edentulism** |  |  |  |  |  |
| Global | 20.38 (10.51 to 33.58) | 20.22 (10.38 to 33.19) | 20.11 (10.33 to 33.15) | 20.06 (10.34 to 32.96) | 20.07 (10.32 to 32.94) |
| Sex |  |  |  |  |  |
| Female | 22.49 (11.71 to 36.91) | 22.27 (11.52 to 36.34) | 22.09 (11.45 to 36.22) | 21.99 (11.43 to 35.94) | 21.98 (11.39 to 36.03) |
| Male | 18.31 (9.28 to 30.3) | 18.23 (9.23 to 30.11) | 18.18 (9.23 to 30.09) | 18.17 (9.26 to 30.13) | 18.21 (9.27 to 30.08) |
| Age* |  |  |  |  |  |
| 20-24 years | 4.7 (2.09 to 8.4) | 4.7 (2.1 to 8.33) | 4.73 (2.14 to 8.4) | 4.78 (2.14 to 8.51) | 4.83 (2.17 to 8.56) |
| 25-29 years | 13.39 (6.56 to 22.16) | 13.11 (6.38 to 21.63) | 12.89 (6.28 to 21.41) | 12.79 (6.27 to 21.28) | 12.77 (6.26 to 21.24) |
| 30-34 years | 25.66 (13.23 to 42.74) | 25.71 (13.14 to 42.57) | 25.57 (13.11 to 42.56) | 25.2 (12.95 to 41.74) | 24.72 (12.69 to 41.12) |
| 35-39 years | 40.4 (21.6 to 65.23) | 39.99 (21.35 to 64.37) | 39.86 (21.21 to 64.36) | 40.09 (21.44 to 64.48) | 40.62 (21.59 to 65.05) |
| Sociodemographic index |  |  |  |  |  |
| Low SDI | 32.96 (17.27 to 53.98) | 33.2 (17.36 to 54.37) | 33.45 (17.45 to 54.91) | 33.64 (17.64 to 54.88) | 33.8 (17.75 to 55.29) |
| Low-middle SDI | 23.84 (12.04 to 39.68) | 23.86 (12.05 to 39.81) | 23.86 (12.1 to 39.56) | 23.83 (12.08 to 39.44) | 23.77 (12.03 to 39.36) |
| Middle SDI | 19.71 (10.09 to 32.39) | 19.33 (9.83 to 31.7) | 19 (9.69 to 31.26) | 18.8 (9.6 to 30.9) | 18.72 (9.52 to 30.74) |
| High-middle SDI | 18.62 (9.34 to 30.8) | 18.26 (9.23 to 29.85) | 17.92 (9.07 to 29.47) | 17.65 (8.98 to 29.12) | 17.45 (8.9 to 28.62) |
| High SDI | 14.26 (7.11 to 23.81) | 14.33 (7.17 to 23.98) | 14.46 (7.25 to 24.24) | 14.62 (7.24 to 24.26) | 14.87 (7.36 to 24.86) |
| **Other oral disorders** |  |  |  |  |  |
| Global | 47.01 (28.47 to 70.86) | 47.03 (28.47 to 70.83) | 47.06 (28.45 to 70.81) | 47.08 (28.44 to 70.94) | 47.09 (28.45 to 70.91) |
| Sex |  |  |  |  |  |
| Female | 50.76 (30.76 to 76.33) | 50.78 (30.73 to 76.2) | 50.81 (30.71 to 76.26) | 50.84 (30.68 to 76.38) | 50.84 (30.69 to 76.31) |
| Male | 43.37 (26.19 to 65.31) | 43.4 (26.25 to 65.26) | 43.42 (26.24 to 65.27) | 43.44 (26.26 to 65.47) | 43.46 (26.24 to 65.4) |
| Age* |  |  |  |  |  |
| 2-4 years | 41.57 (24.93 to 61.63) | 41.59 (24.92 to 61.69) | 41.61 (24.84 to 61.61) | 41.64 (24.89 to 61.87) | 41.64 (24.88 to 61.93) |
| 5-9 years | 30.54 (18.06 to 46.79) | 30.57 (18.05 to 46.9) | 30.57 (18.06 to 46.79) | 30.57 (18.03 to 46.76) | 30.57 (17.98 to 46.85) |
| 10-14 years | 32.19 (18.98 to 49.93) | 32.18 (18.98 to 49.98) | 32.17 (18.92 to 49.74) | 32.19 (18.89 to 49.81) | 32.19 (19.01 to 49.46) |
| 15-19 years | 47.91 (28.9 to 72.88) | 47.92 (28.9 to 73.09) | 47.94 (28.77 to 73.12) | 47.94 (28.76 to 73.1) | 47.96 (28.76 to 73.17) |
| 20-24 years | 58.4 (35.52 to 87.2) | 58.41 (35.49 to 86.73) | 58.4 (35.47 to 86.88) | 58.42 (35.43 to 86.69) | 58.44 (35.53 to 86.93) |
| 25-29 years | 63.63 (39.21 to 94.55) | 63.65 (39.02 to 94.63) | 63.67 (39.13 to 94.87) | 63.69 (39.26 to 95.25) | 63.69 (39.08 to 95.05) |
| 30-34 years | 67 (41.01 to 100.44) | 67.03 (41.04 to 100.42) | 67.08 (41.11 to 100.37) | 67.11 (40.94 to 100.52) | 67.15 (41.06 to 100.46) |
| 35-39 years | 68.68 (41.85 to 103.75) | 68.71 (42.03 to 103.32) | 68.78 (41.96 to 103.05) | 68.8 (41.97 to 103.66) | 68.83 (41.96 to 103.57) |
| Sociodemographic index |  |  |  |  |  |
| Low SDI | 46.5 (28.33 to 69.74) | 46.49 (28.23 to 69.8) | 46.49 (28.16 to 69.44) | 46.5 (28.32 to 69.61) | 46.51 (28.22 to 69.68) |
| Low-middle SDI | 46.81 (28.43 to 70.4) | 46.8 (28.4 to 70.36) | 46.8 (28.35 to 70.46) | 46.8 (28.31 to 70.39) | 46.81 (28.35 to 70.36) |
| Middle SDI | 47.21 (28.58 to 71.26) | 47.22 (28.52 to 71.22) | 47.24 (28.53 to 71.23) | 47.26 (28.56 to 71.39) | 47.27 (28.49 to 71.35) |
| High-middle SDI | 47.28 (28.49 to 71.41) | 47.32 (28.56 to 71.45) | 47.36 (28.58 to 71.25) | 47.38 (28.55 to 71.49) | 47.36 (28.48 to 71.24) |
| High SDI | 46.93 (28.25 to 70.76) | 47.05 (28.45 to 70.87) | 47.16 (28.42 to 70.94) | 47.27 (28.55 to 71.06) | 47.34 (28.61 to 71.37) |
| **Characteristic** | 1995 | 1996 | 1997 | 1998 | 1999 |
| **Caries of deciduous teeth** |  |  |  |  |  |
| Global | 10.65 (4.4 to 20.98) | 10.63 (4.41 to 20.97) | 10.6 (4.4 to 20.9) | 10.58 (4.4 to 20.83) | 10.56 (4.39 to 20.85) |
| Sex |  |  |  |  |  |
| Female | 10.59 (4.39 to 20.86) | 10.57 (4.4 to 20.83) | 10.53 (4.38 to 20.83) | 10.5 (4.37 to 20.65) | 10.46 (4.36 to 20.65) |
| Male | 10.7 (4.42 to 21.06) | 10.68 (4.42 to 21.1) | 10.67 (4.42 to 20.96) | 10.66 (4.42 to 21.02) | 10.64 (4.42 to 21.03) |
| Age* |  |  |  |  |  |
| 2-4 years | 20.37 (8.73 to 40.66) | 20.3 (8.74 to 40.69) | 20.22 (8.69 to 40.68) | 20.13 (8.65 to 40.38) | 20.06 (8.64 to 40.27) |
| 5-9 years | 15.87 (6.72 to 30.12) | 15.87 (6.72 to 30.22) | 15.86 (6.74 to 30.01) | 15.85 (6.75 to 29.9) | 15.82 (6.73 to 29.98) |
| 10-14 years | 2.29 (0.58 to 5.37) | 2.29 (0.59 to 5.35) | 2.29 (0.6 to 5.42) | 2.29 (0.6 to 5.4) | 2.29 (0.6 to 5.43) |
| Sociodemographic index |  |  |  |  |  |
| Low SDI | 10.25 (4.22 to 20.24) | 10.24 (4.28 to 20.21) | 10.24 (4.27 to 20.17) | 10.24 (4.27 to 20.18) | 10.23 (4.26 to 20.18) |
| Low-middle SDI | 10.65 (4.39 to 21.17) | 10.65 (4.4 to 21.24) | 10.63 (4.4 to 21.17) | 10.61 (4.38 to 21.01) | 10.58 (4.38 to 20.92) |
| Middle SDI | 11.19 (4.66 to 22.05) | 11.18 (4.64 to 22.14) | 11.19 (4.65 to 22.19) | 11.2 (4.66 to 22.16) | 11.2 (4.66 to 22.18) |
| High-middle SDI | 11.32 (4.66 to 22.27) | 11.28 (4.66 to 22.16) | 11.26 (4.66 to 22.28) | 11.26 (4.64 to 22.28) | 11.25 (4.66 to 22.18) |
| High SDI | 8.57 (3.59 to 16.98) | 8.5 (3.54 to 16.74) | 8.37 (3.49 to 16.53) | 8.23 (3.43 to 16.21) | 8.11 (3.41 to 15.96) |
| **Caries of permanent teeth** |  |  |  |  |  |
| Global | 29.57 (11.74 to 59.17) | 29.54 (11.74 to 59.09) | 29.51 (11.73 to 59) | 29.49 (11.72 to 58.95) | 29.47 (11.7 to 58.95) |
| Sex |  |  |  |  |  |
| Female | 29.9 (11.87 to 59.88) | 29.87 (11.87 to 59.78) | 29.83 (11.85 to 59.62) | 29.79 (11.84 to 59.45) | 29.77 (11.82 to 59.45) |
| Male | 29.26 (11.63 to 58.54) | 29.23 (11.62 to 58.49) | 29.21 (11.61 to 58.48) | 29.19 (11.59 to 58.49) | 29.18 (11.58 to 58.5) |
| Age* |  |  |  |  |  |
| 5-9 years | 9.21 (3.1 to 21.26) | 9.14 (3.08 to 21.08) | 9.1 (3.05 to 20.87) | 9.08 (3.08 to 20.82) | 9.1 (3.06 to 20.92) |
| 10-14 years | 26.14 (10.35 to 53.77) | 25.97 (10.25 to 53.45) | 25.76 (10.15 to 52.99) | 25.6 (10.08 to 52.69) | 25.49 (10.03 to 52.51) |
| 15-19 years | 31.19 (11.96 to 64.42) | 31.2 (11.99 to 64.56) | 31.2 (11.97 to 64.56) | 31.19 (11.97 to 64.46) | 31.18 (11.96 to 64.46) |
| 20-24 years | 39.24 (15.12 to 76.78) | 39.29 (15.14 to 76.7) | 39.34 (15.16 to 76.97) | 39.38 (15.17 to 77.01) | 39.42 (15.18 to 77.12) |
| 25-29 years | 37.31 (15.47 to 71.18) | 37.34 (15.51 to 71.15) | 37.4 (15.55 to 71.35) | 37.46 (15.59 to 71.44) | 37.54 (15.61 to 71.62) |
| 30-34 years | 34.61 (14.45 to 67.25) | 34.48 (14.42 to 67.05) | 34.4 (14.37 to 66.71) | 34.4 (14.34 to 66.78) | 34.44 (14.36 to 66.77) |
| 35-39 years | 35.86 (14.64 to 71.36) | 36 (14.7 to 71.59) | 36.04 (14.77 to 71.59) | 35.96 (14.7 to 71.54) | 35.8 (14.62 to 71.28) |
| Sociodemographic index |  |  |  |  |  |
| Low SDI | 33.31 (13.62 to 65.69) | 33.28 (13.58 to 65.81) | 33.18 (13.55 to 65.66) | 33.08 (13.51 to 65.54) | 33.01 (13.45 to 65.48) |
| Low-middle SDI | 30.7 (12.04 to 62.03) | 30.68 (12.06 to 61.96) | 30.65 (12.04 to 61.84) | 30.63 (12.04 to 61.74) | 30.64 (12.01 to 61.9) |
| Middle SDI | 29.29 (11.4 to 59.32) | 29.32 (11.41 to 59.29) | 29.37 (11.46 to 59.4) | 29.43 (11.51 to 59.55) | 29.49 (11.54 to 59.54) |
| High-middle SDI | 29.59 (11.66 to 59.42) | 29.46 (11.61 to 59.14) | 29.29 (11.51 to 58.72) | 29.13 (11.42 to 58.39) | 28.99 (11.33 to 58.06) |
| High SDI | 26.16 (10.71 to 52.33) | 26.04 (10.67 to 52.03) | 25.91 (10.62 to 51.74) | 25.77 (10.57 to 51.38) | 25.66 (10.53 to 51.27) |
| **Periodontal diseases** |  |  |  |  |  |
| Global | 55.07 (20.64 to 118.24) | 55.35 (20.81 to 117.74) | 55.64 (20.99 to 117.42) | 55.88 (21.15 to 117.41) | 56.11 (21.29 to 117.51) |
| Sex |  |  |  |  |  |
| Female | 53.38 (20.08 to 113.88) | 53.68 (20.25 to 113.74) | 53.99 (20.44 to 113.66) | 54.24 (20.55 to 113.61) | 54.49 (20.69 to 114.16) |
| Male | 56.72 (21.2 to 122.49) | 56.98 (21.36 to 121.89) | 57.25 (21.52 to 121.13) | 57.49 (21.72 to 120.96) | 57.7 (21.88 to 120.9) |
| Age* |  |  |  |  |  |
| 15-19 years | 8.27 (2.39 to 20.43) | 8.38 (2.48 to 20.37) | 8.48 (2.55 to 20.37) | 8.55 (2.57 to 20.32) | 8.61 (2.58 to 20.29) |
| 20-24 years | 26.5 (9.45 to 62.8) | 26.98 (9.61 to 63.41) | 27.49 (9.82 to 64.07) | 27.98 (9.98 to 64.72) | 28.42 (10.14 to 65.35) |
| 25-29 years | 53.23 (20.1 to 114.4) | 53.71 (20.23 to 114.06) | 54.27 (20.37 to 113.66) | 54.83 (20.59 to 113.99) | 55.48 (20.92 to 114.88) |
| 30-34 years | 83.4 (29.44 to 178) | 83.07 (29.37 to 175.82) | 83.09 (29.58 to 176.05) | 83.49 (29.96 to 177.67) | 84.1 (30.39 to 177.39) |
| 35-39 years | 116.19 (46.77 to 240.53) | 116.87 (47.34 to 239.84) | 117.14 (47.63 to 237.48) | 116.79 (47.63 to 234.66) | 116.11 (47.39 to 233.84) |
| Sociodemographic index |  |  |  |  |  |
| Low SDI | 101.24 (37.6 to 215.17) | 100.83 (37.53 to 214.2) | 99.94 (37.3 to 211.9) | 98.88 (36.94 to 209.62) | 97.99 (36.7 to 207.36) |
| Low-middle SDI | 83.84 (31.31 to 178.37) | 84.28 (31.54 to 178.6) | 84.81 (31.78 to 178.92) | 85.34 (32.02 to 179.34) | 85.81 (32.35 to 179.89) |
| Middle SDI | 47.35 (17.57 to 101.59) | 47.66 (17.78 to 101.92) | 48.1 (18 to 102.22) | 48.52 (18.2 to 102.61) | 48.88 (18.37 to 103.21) |
| High-middle SDI | 32.06 (11.47 to 71.02) | 31.75 (11.49 to 69.64) | 31.46 (11.52 to 68.42) | 31.19 (11.63 to 67.05) | 30.99 (11.76 to 65.8) |
| High SDI | 40.97 (15.34 to 86.43) | 40.91 (15.58 to 85.87) | 40.76 (15.71 to 85.04) | 40.54 (15.86 to 85.09) | 40.26 (15.74 to 85.58) |
| **Edentulism** |  |  |  |  |  |
| Global | 20.16 (10.38 to 33.12) | 20.03 (10.35 to 32.83) | 19.55 (10.13 to 32.17) | 18.92 (9.8 to 31.08) | 18.37 (9.53 to 30.17) |
| Sex |  |  |  |  |  |
| Female | 22.08 (11.47 to 36.21) | 21.93 (11.45 to 35.76) | 21.42 (11.15 to 34.96) | 20.72 (10.83 to 33.9) | 20.12 (10.51 to 32.94) |
| Male | 18.29 (9.29 to 30.13) | 18.17 (9.25 to 30.08) | 17.72 (9.06 to 29.48) | 17.15 (8.79 to 28.31) | 16.64 (8.51 to 27.49) |
| Age* |  |  |  |  |  |
| 20-24 years | 4.9 (2.21 to 8.75) | 4.92 (2.22 to 8.83) | 4.87 (2.22 to 8.75) | 4.77 (2.18 to 8.56) | 4.7 (2.17 to 8.36) |
| 25-29 years | 12.84 (6.36 to 21.36) | 12.78 (6.21 to 21.01) | 12.54 (6.11 to 20.79) | 12.25 (6.02 to 20.17) | 12.04 (5.9 to 19.94) |
| 30-34 years | 24.3 (12.51 to 40.35) | 23.73 (12.27 to 39.29) | 22.9 (11.81 to 38.19) | 22.15 (11.44 to 36.87) | 21.61 (11.15 to 36.02) |
| 35-39 years | 41.29 (21.93 to 66.29) | 41.39 (22.17 to 66.45) | 40.53 (21.82 to 65.1) | 39.03 (20.94 to 62.71) | 37.53 (20.24 to 60.21) |
| Sociodemographic index |  |  |  |  |  |
| Low SDI | 33.97 (17.89 to 55.67) | 33.72 (17.7 to 55.34) | 32.99 (17.33 to 54.07) | 32.1 (17.03 to 52.5) | 31.36 (16.57 to 51.15) |
| Low-middle SDI | 23.73 (12.02 to 39.32) | 23.04 (11.69 to 38) | 21.48 (10.98 to 35.42) | 19.66 (10.11 to 32.44) | 18.15 (9.36 to 29.81) |
| Middle SDI | 18.76 (9.57 to 30.95) | 18.56 (9.49 to 30.44) | 17.96 (9.17 to 29.65) | 17.16 (8.79 to 28.25) | 16.49 (8.43 to 27.17) |
| High-middle SDI | 17.31 (8.83 to 28.45) | 17.27 (8.82 to 28.33) | 17.28 (8.87 to 28.55) | 17.23 (8.94 to 28.22) | 17.08 (8.88 to 28.02) |
| High SDI | 15.23 (7.55 to 25.41) | 15.64 (7.76 to 26.21) | 16 (7.99 to 26.89) | 16.35 (8.11 to 27.38) | 16.7 (8.28 to 28.02) |
| **Other oral disorders** |  |  |  |  |  |
| Global | 47.09 (28.47 to 70.94) | 47.09 (28.46 to 70.92) | 47.1 (28.5 to 71.06) | 47.1 (28.44 to 70.94) | 47.1 (28.48 to 70.93) |
| Sex |  |  |  |  |  |
| Female | 50.84 (30.75 to 76.32) | 50.84 (30.79 to 76.37) | 50.85 (30.75 to 76.49) | 50.85 (30.79 to 76.43) | 50.85 (30.76 to 76.4) |
| Male | 43.46 (26.25 to 65.39) | 43.45 (26.24 to 65.37) | 43.46 (26.23 to 65.45) | 43.46 (26.26 to 65.53) | 43.45 (26.27 to 65.38) |
| Age* |  |  |  |  |  |
| 2-4 years | 41.63 (24.93 to 61.72) | 41.63 (24.83 to 61.83) | 41.63 (24.92 to 62.13) | 41.62 (24.86 to 61.75) | 41.63 (24.76 to 61.91) |
| 5-9 years | 30.57 (18.01 to 46.93) | 30.57 (18.17 to 46.78) | 30.58 (17.99 to 46.98) | 30.56 (17.97 to 46.73) | 30.56 (17.97 to 46.75) |
| 10-14 years | 32.18 (18.84 to 49.67) | 32.2 (18.85 to 49.86) | 32.22 (18.87 to 50.06) | 32.21 (18.88 to 49.85) | 32.21 (18.98 to 49.73) |
| 15-19 years | 47.96 (28.87 to 73.26) | 47.96 (28.7 to 73) | 47.96 (28.88 to 72.83) | 47.96 (28.8 to 73.14) | 47.96 (29.03 to 73.08) |
| 20-24 years | 58.45 (35.62 to 87.04) | 58.47 (35.46 to 87.14) | 58.48 (35.61 to 87.02) | 58.49 (35.46 to 87.2) | 58.5 (35.35 to 86.77) |
| 25-29 years | 63.72 (39.15 to 95.13) | 63.7 (39.07 to 95.04) | 63.72 (39.17 to 95.3) | 63.71 (39.17 to 95.44) | 63.72 (39.15 to 94.98) |
| 30-34 years | 67.17 (41.2 to 100.72) | 67.17 (41.31 to 100.72) | 67.21 (41.2 to 100.84) | 67.21 (41.1 to 100.41) | 67.19 (41.1 to 101.03) |
| 35-39 years | 68.86 (41.8 to 103.17) | 68.85 (41.9 to 103.18) | 68.87 (42.13 to 103.69) | 68.89 (41.94 to 103.29) | 68.88 (42.18 to 103.6) |
| Sociodemographic index |  |  |  |  |  |
| Low SDI | 46.5 (28.13 to 69.45) | 46.5 (28.23 to 69.64) | 46.52 (28.21 to 69.62) | 46.52 (28.27 to 69.57) | 46.54 (28.28 to 69.76) |
| Low-middle SDI | 46.82 (28.35 to 70.3) | 46.83 (28.41 to 70.26) | 46.84 (28.42 to 70.54) | 46.86 (28.35 to 70.52) | 46.88 (28.38 to 70.61) |
| Middle SDI | 47.26 (28.49 to 71.16) | 47.25 (28.55 to 71.43) | 47.27 (28.57 to 71.51) | 47.27 (28.55 to 71.44) | 47.27 (28.58 to 71.33) |
| High-middle SDI | 47.36 (28.62 to 71.4) | 47.35 (28.59 to 71.31) | 47.35 (28.61 to 71.57) | 47.34 (28.57 to 71.41) | 47.33 (28.63 to 71.29) |
| High SDI | 47.38 (28.68 to 71.38) | 47.4 (28.64 to 71.33) | 47.39 (28.59 to 71.31) | 47.39 (28.61 to 71.08) | 47.36 (28.54 to 71.19) |
| **Characteristic** | 2000 | 2001 | 2002 | 2003 | 2004 |
| **Caries of deciduous teeth** |  |  |  |  |  |
| Global | 10.53 (4.4 to 20.81) | 10.5 (4.41 to 20.7) | 10.45 (4.38 to 20.61) | 10.39 (4.38 to 20.44) | 10.35 (4.35 to 20.35) |
| Sex |  |  |  |  |  |
| Female | 10.44 (4.37 to 20.63) | 10.39 (4.36 to 20.42) | 10.34 (4.34 to 20.38) | 10.27 (4.33 to 20.18) | 10.22 (4.3 to 20.06) |
| Male | 10.62 (4.43 to 20.99) | 10.6 (4.44 to 20.93) | 10.55 (4.41 to 20.82) | 10.5 (4.42 to 20.68) | 10.46 (4.39 to 20.62) |
| Age* |  |  |  |  |  |
| 2-4 years | 19.99 (8.64 to 40.07) | 19.92 (8.62 to 39.83) | 19.85 (8.56 to 39.54) | 19.78 (8.59 to 39.26) | 19.72 (8.54 to 38.98) |
| 5-9 years | 15.81 (6.77 to 30.04) | 15.77 (6.77 to 29.9) | 15.7 (6.75 to 29.77) | 15.6 (6.72 to 29.46) | 15.53 (6.67 to 29.39) |
| 10-14 years | 2.29 (0.6 to 5.41) | 2.28 (0.61 to 5.37) | 2.26 (0.62 to 5.26) | 2.24 (0.61 to 5.23) | 2.21 (0.6 to 5.19) |
| Sociodemographic index |  |  |  |  |  |
| Low SDI | 10.23 (4.25 to 20.19) | 10.18 (4.29 to 19.95) | 10.08 (4.24 to 19.78) | 9.97 (4.21 to 19.41) | 9.87 (4.16 to 19.17) |
| Low-middle SDI | 10.57 (4.39 to 20.98) | 10.53 (4.39 to 20.87) | 10.48 (4.35 to 20.71) | 10.42 (4.35 to 20.72) | 10.36 (4.29 to 20.51) |
| Middle SDI | 11.2 (4.68 to 22.27) | 11.19 (4.7 to 22.21) | 11.19 (4.7 to 22.22) | 11.19 (4.71 to 22.13) | 11.2 (4.72 to 22.18) |
| High-middle SDI | 11.22 (4.66 to 22.25) | 11.18 (4.63 to 22.11) | 11.16 (4.64 to 22.18) | 11.15 (4.67 to 22.13) | 11.15 (4.65 to 22.12) |
| High SDI | 8.02 (3.37 to 15.88) | 7.95 (3.35 to 15.65) | 7.84 (3.27 to 15.37) | 7.71 (3.23 to 15.12) | 7.59 (3.19 to 14.89) |
| **Caries of permanent teeth** |  |  |  |  |  |
| Global | 29.47 (11.72 to 58.99) | 29.58 (11.75 to 59.13) | 29.83 (11.84 to 59.6) | 30.12 (11.97 to 60.21) | 30.38 (12.09 to 60.67) |
| Sex |  |  |  |  |  |
| Female | 29.77 (11.85 to 59.39) | 29.88 (11.88 to 59.66) | 30.12 (11.97 to 60.09) | 30.42 (12.1 to 60.76) | 30.67 (12.21 to 61.28) |
| Male | 29.19 (11.59 to 58.61) | 29.3 (11.63 to 58.63) | 29.55 (11.72 to 59.14) | 29.84 (11.84 to 59.73) | 30.1 (11.96 to 60.12) |
| Age* |  |  |  |  |  |
| 5-9 years | 9.14 (3.08 to 20.85) | 9.22 (3.12 to 21.04) | 9.34 (3.15 to 21.43) | 9.46 (3.19 to 21.66) | 9.57 (3.23 to 21.98) |
| 10-14 years | 25.47 (10.03 to 52.55) | 25.56 (10.05 to 52.64) | 25.74 (10.09 to 53.03) | 25.95 (10.19 to 53.51) | 26.16 (10.29 to 53.92) |
| 15-19 years | 31.18 (11.96 to 64.45) | 31.28 (12.02 to 64.82) | 31.53 (12.15 to 65.25) | 31.82 (12.27 to 66.02) | 32.06 (12.41 to 66.39) |
| 20-24 years | 39.42 (15.19 to 77.16) | 39.48 (15.12 to 76.96) | 39.65 (15.09 to 77.26) | 39.85 (15.18 to 77.74) | 39.97 (15.24 to 77.85) |
| 25-29 years | 37.64 (15.69 to 71.89) | 37.87 (15.76 to 72.03) | 38.26 (16 to 72.41) | 38.69 (16.2 to 73.13) | 39.05 (16.31 to 73.6) |
| 30-34 years | 34.51 (14.42 to 67.1) | 34.72 (14.57 to 67.7) | 35.15 (14.72 to 68.66) | 35.64 (14.94 to 69.52) | 36.08 (15.17 to 70.54) |
| 35-39 years | 35.6 (14.57 to 70.98) | 35.59 (14.55 to 70.72) | 35.83 (14.65 to 71.24) | 36.23 (14.83 to 72.13) | 36.62 (14.98 to 72.76) |
| Sociodemographic index |  |  |  |  |  |
| Low SDI | 32.98 (13.48 to 65.32) | 33.09 (13.5 to 65.54) | 33.34 (13.63 to 66.15) | 33.64 (13.71 to 66.46) | 33.88 (13.82 to 66.98) |
| Low-middle SDI | 30.67 (12.02 to 61.89) | 30.92 (12.13 to 62.19) | 31.46 (12.38 to 63.35) | 32.08 (12.63 to 64.55) | 32.59 (12.89 to 65.5) |
| Middle SDI | 29.53 (11.58 to 59.74) | 29.63 (11.63 to 59.8) | 29.87 (11.73 to 60.23) | 30.16 (11.86 to 60.82) | 30.41 (11.99 to 61.3) |
| High-middle SDI | 28.9 (11.34 to 57.96) | 28.89 (11.37 to 58.24) | 28.93 (11.4 to 58.21) | 29.01 (11.47 to 58.51) | 29.09 (11.53 to 58.62) |
| High SDI | 25.57 (10.54 to 50.99) | 25.49 (10.5 to 50.91) | 25.35 (10.45 to 50.63) | 25.19 (10.38 to 50.28) | 25.05 (10.33 to 50.03) |
| **Periodontal diseases** |  |  |  |  |  |
| Global | 56.34 (21.45 to 118.03) | 56.49 (21.6 to 118.44) | 56.53 (21.74 to 118.49) | 56.58 (21.78 to 118.73) | 56.69 (21.87 to 119.08) |
| Sex |  |  |  |  |  |
| Female | 54.74 (20.92 to 114.69) | 54.89 (21.07 to 114.99) | 54.87 (21.14 to 114.93) | 54.84 (21.16 to 114.95) | 54.88 (21.24 to 115.6) |
| Male | 57.91 (22.02 to 121.27) | 58.06 (22.14 to 121.74) | 58.17 (22.29 to 121.94) | 58.3 (22.38 to 122.34) | 58.47 (22.49 to 122.85) |
| Age* |  |  |  |  |  |
| 15-19 years | 8.64 (2.61 to 20.33) | 8.61 (2.62 to 20.1) | 8.51 (2.59 to 19.75) | 8.41 (2.55 to 19.46) | 8.35 (2.54 to 19.26) |
| 20-24 years | 28.81 (10.27 to 65.64) | 28.99 (10.41 to 65.56) | 28.88 (10.47 to 64.74) | 28.62 (10.37 to 63.95) | 28.37 (10.31 to 63.02) |
| 25-29 years | 56.23 (21.41 to 116.94) | 56.86 (21.76 to 118.66) | 57.15 (22.06 to 119.74) | 57.23 (22.16 to 120.41) | 57.28 (22.32 to 121.19) |
| 30-34 years | 84.75 (30.89 to 177.06) | 85.17 (31.23 to 178.38) | 85.36 (31.47 to 179.07) | 85.48 (31.61 to 180) | 85.74 (31.81 to 180.33) |
| 35-39 years | 115.34 (47.01 to 234.43) | 114.85 (46.93 to 233.71) | 114.81 (47.07 to 233.44) | 115.27 (47.2 to 234.24) | 115.91 (47.39 to 236.32) |
| Sociodemographic index |  |  |  |  |  |
| Low SDI | 97.59 (36.63 to 206.51) | 97.15 (36.52 to 205.12) | 96.05 (36.22 to 202.4) | 94.68 (35.79 to 199.28) | 93.49 (35.28 to 196.73) |
| Low-middle SDI | 86.17 (32.75 to 180.6) | 86.13 (32.79 to 180.45) | 85.63 (32.72 to 178.58) | 84.99 (32.52 to 177.1) | 84.5 (32.41 to 175.83) |
| Middle SDI | 49.13 (18.45 to 103.43) | 49.34 (18.56 to 103.59) | 49.6 (18.69 to 103.93) | 49.92 (18.84 to 104.64) | 50.26 (18.97 to 105.26) |
| High-middle SDI | 30.92 (11.87 to 65.27) | 31.51 (12.12 to 66.84) | 32.87 (12.62 to 70.28) | 34.51 (13.21 to 74.04) | 35.91 (13.67 to 77.56) |
| High SDI | 39.94 (15.7 to 86.08) | 38.85 (15.32 to 84.39) | 36.79 (14.44 to 80.7) | 34.44 (13.45 to 75.53) | 32.47 (12.63 to 71.62) |
| **Edentulism** |  |  |  |  |  |
| Global | 18.13 (9.38 to 29.7) | 18.54 (9.78 to 30.28) | 19.55 (10.45 to 31.76) | 20.85 (11.36 to 33.57) | 22.1 (12.19 to 35.43) |
| Sex |  |  |  |  |  |
| Female | 19.86 (10.37 to 32.39) | 20.32 (10.77 to 33.02) | 21.46 (11.59 to 34.64) | 22.93 (12.65 to 36.63) | 24.33 (13.55 to 38.72) |
| Male | 16.43 (8.42 to 27.15) | 16.78 (8.72 to 27.61) | 17.65 (9.28 to 28.82) | 18.79 (10.07 to 30.49) | 19.88 (10.72 to 32.12) |
| Age* |  |  |  |  |  |
| 20-24 years | 4.68 (2.14 to 8.31) | 4.93 (2.32 to 8.77) | 5.48 (2.69 to 9.61) | 6.15 (3.11 to 10.66) | 6.77 (3.46 to 11.69) |
| 25-29 years | 12.1 (5.94 to 20.14) | 12.7 (6.34 to 20.94) | 13.84 (7.07 to 22.98) | 15.23 (7.99 to 24.88) | 16.54 (8.89 to 26.96) |
| 30-34 years | 21.53 (11.08 to 35.52) | 22.21 (11.69 to 36.57) | 23.57 (12.49 to 38.18) | 25.22 (13.65 to 40.55) | 26.81 (14.75 to 42.8) |
| 35-39 years | 36.56 (19.64 to 58.53) | 36.64 (20.07 to 58.47) | 37.64 (20.88 to 59.93) | 39.2 (22.06 to 61.94) | 40.73 (23.07 to 64.05) |
| Sociodemographic index |  |  |  |  |  |
| Low SDI | 31.13 (16.39 to 50.6) | 31.45 (16.7 to 51.18) | 32.12 (17.24 to 52.09) | 32.98 (17.92 to 52.94) | 33.87 (18.58 to 54.74) |
| Low-middle SDI | 17.54 (9.06 to 28.8) | 18.08 (9.5 to 29.55) | 19.39 (10.39 to 31.32) | 21.08 (11.58 to 33.79) | 22.75 (12.63 to 36.21) |
| Middle SDI | 16.2 (8.27 to 26.61) | 16.78 (8.72 to 27.44) | 18.24 (9.69 to 29.57) | 20.11 (10.91 to 32.44) | 21.86 (11.98 to 35.02) |
| High-middle SDI | 16.88 (8.81 to 27.67) | 16.79 (8.83 to 27.3) | 16.94 (8.96 to 27.34) | 17.28 (9.39 to 27.63) | 17.65 (9.72 to 28.1) |
| High SDI | 16.94 (8.43 to 28.53) | 17.41 (8.76 to 29.19) | 18.18 (9.2 to 30) | 19.01 (9.79 to 31.47) | 19.68 (10.35 to 32.16) |
| **Other oral disorders** |  |  |  |  |  |
| Global | 47.09 (28.43 to 70.99) | 47.08 (28.47 to 70.96) | 47.05 (28.43 to 70.91) | 47.04 (28.42 to 70.92) | 47.01 (28.43 to 70.89) |
| Sex |  |  |  |  |  |
| Female | 50.84 (30.72 to 76.44) | 50.82 (30.72 to 76.44) | 50.77 (30.7 to 76.28) | 50.75 (30.64 to 76.37) | 50.71 (30.64 to 76.21) |
| Male | 43.46 (26.25 to 65.37) | 43.44 (26.29 to 65.4) | 43.44 (26.27 to 65.46) | 43.43 (26.28 to 65.49) | 43.42 (26.21 to 65.45) |
| Age* |  |  |  |  |  |
| 2-4 years | 41.6 (24.83 to 61.98) | 41.61 (24.96 to 61.78) | 41.61 (24.81 to 61.84) | 41.62 (24.77 to 62.03) | 41.62 (24.92 to 61.96) |
| 5-9 years | 30.55 (17.99 to 46.93) | 30.54 (18.05 to 46.62) | 30.53 (18.03 to 46.65) | 30.53 (18.04 to 46.71) | 30.52 (18.04 to 46.85) |
| 10-14 years | 32.22 (18.87 to 49.99) | 32.2 (18.95 to 49.99) | 32.18 (18.93 to 49.74) | 32.17 (18.91 to 49.99) | 32.15 (18.75 to 49.83) |
| 15-19 years | 47.96 (28.89 to 72.99) | 47.98 (29.02 to 73.09) | 47.96 (28.84 to 73.06) | 47.94 (28.78 to 72.97) | 47.95 (28.85 to 73.2) |
| 20-24 years | 58.51 (35.34 to 86.98) | 58.52 (35.48 to 87.35) | 58.49 (35.39 to 87.08) | 58.47 (35.36 to 87.01) | 58.44 (35.42 to 87.04) |
| 25-29 years | 63.73 (39.12 to 95.26) | 63.72 (39.1 to 95.06) | 63.69 (39.08 to 94.89) | 63.66 (39.13 to 94.91) | 63.64 (39.27 to 94.67) |
| 30-34 years | 67.19 (41.21 to 100.84) | 67.16 (41.14 to 100.94) | 67.12 (41.18 to 100.95) | 67.11 (41.08 to 100.76) | 67.06 (41.1 to 100.67) |
| 35-39 years | 68.89 (41.9 to 103.35) | 68.87 (41.84 to 103.32) | 68.83 (41.87 to 103.63) | 68.8 (42 to 103.57) | 68.75 (41.91 to 103.42) |
| Sociodemographic index |  |  |  |  |  |
| Low SDI | 46.54 (28.12 to 69.64) | 46.57 (28.21 to 69.65) | 46.58 (28.3 to 69.67) | 46.6 (28.28 to 69.89) | 46.62 (28.32 to 69.85) |
| Low-middle SDI | 46.88 (28.43 to 70.65) | 46.89 (28.46 to 70.46) | 46.88 (28.36 to 70.69) | 46.9 (28.39 to 70.66) | 46.91 (28.43 to 70.74) |
| Middle SDI | 47.27 (28.54 to 71.47) | 47.27 (28.53 to 71.31) | 47.27 (28.57 to 71.46) | 47.27 (28.48 to 71.46) | 47.26 (28.5 to 71.44) |
| High-middle SDI | 47.32 (28.48 to 71.36) | 47.28 (28.55 to 71.47) | 47.27 (28.57 to 71.29) | 47.26 (28.54 to 71.29) | 47.25 (28.53 to 71.33) |
| High SDI | 47.33 (28.54 to 70.97) | 47.25 (28.53 to 70.8) | 47.11 (28.51 to 70.61) | 46.93 (28.33 to 70.49) | 46.76 (28.25 to 70.23) |
| **Characteristic** | 2005 | 2006 | 2007 | 2008 | 2009 |
| **Caries of deciduous teeth** |  |  |  |  |  |
| Global | 10.31 (4.33 to 20.24) | 10.28 (4.33 to 20.18) | 10.23 (4.31 to 20.05) | 10.18 (4.3 to 19.88) | 10.14 (4.3 to 19.82) |
| Sex |  |  |  |  |  |
| Female | 10.19 (4.29 to 20.01) | 10.16 (4.3 to 19.93) | 10.13 (4.29 to 19.87) | 10.1 (4.27 to 19.72) | 10.08 (4.29 to 19.75) |
| Male | 10.43 (4.37 to 20.46) | 10.38 (4.36 to 20.42) | 10.32 (4.34 to 20.21) | 10.25 (4.31 to 20.02) | 10.19 (4.31 to 19.9) |
| Age* |  |  |  |  |  |
| 2-4 years | 19.67 (8.55 to 38.7) | 19.62 (8.53 to 38.66) | 19.56 (8.51 to 38.54) | 19.49 (8.44 to 38.35) | 19.44 (8.48 to 38.33) |
| 5-9 years | 15.48 (6.64 to 29.34) | 15.44 (6.62 to 29.38) | 15.4 (6.58 to 29.24) | 15.35 (6.6 to 29.01) | 15.3 (6.6 to 28.97) |
| 10-14 years | 2.2 (0.6 to 5.18) | 2.19 (0.62 to 5.07) | 2.15 (0.61 to 4.98) | 2.1 (0.59 to 4.85) | 2.06 (0.58 to 4.76) |
| Sociodemographic index |  |  |  |  |  |
| Low SDI | 9.83 (4.17 to 19.13) | 9.79 (4.19 to 19.1) | 9.73 (4.18 to 19.06) | 9.66 (4.17 to 18.95) | 9.59 (4.15 to 18.8) |
| Low-middle SDI | 10.32 (4.28 to 20.37) | 10.28 (4.29 to 20.39) | 10.23 (4.28 to 20.05) | 10.16 (4.23 to 19.79) | 10.09 (4.24 to 19.76) |
| Middle SDI | 11.19 (4.72 to 22.23) | 11.17 (4.72 to 22.11) | 11.14 (4.73 to 22.02) | 11.11 (4.72 to 21.86) | 11.11 (4.73 to 21.86) |
| High-middle SDI | 11.15 (4.64 to 22.01) | 11.12 (4.64 to 22.12) | 11.08 (4.61 to 21.89) | 11.04 (4.61 to 21.94) | 11.03 (4.61 to 21.78) |
| High SDI | 7.51 (3.15 to 14.68) | 7.46 (3.13 to 14.6) | 7.42 (3.11 to 14.5) | 7.38 (3.09 to 14.35) | 7.36 (3.08 to 14.25) |
| **Caries of permanent teeth** |  |  |  |  |  |
| Global | 30.5 (12.15 to 60.95) | 30.48 (12.13 to 60.78) | 30.38 (12.1 to 60.61) | 30.25 (12.02 to 60.34) | 30.13 (11.98 to 60.12) |
| Sex |  |  |  |  |  |
| Female | 30.79 (12.27 to 61.47) | 30.75 (12.25 to 61.25) | 30.64 (12.21 to 61.14) | 30.5 (12.12 to 60.85) | 30.38 (12.08 to 60.65) |
| Male | 30.23 (12.03 to 60.47) | 30.21 (12.01 to 60.33) | 30.13 (11.99 to 60.12) | 30.01 (11.93 to 59.9) | 29.9 (11.88 to 59.65) |
| Age* |  |  |  |  |  |
| 5-9 years | 9.61 (3.25 to 22.15) | 9.56 (3.23 to 22.06) | 9.44 (3.17 to 21.82) | 9.29 (3.11 to 21.56) | 9.15 (3.05 to 21.24) |
| 10-14 years | 26.31 (10.32 to 54.22) | 26.39 (10.34 to 54.45) | 26.39 (10.36 to 54.34) | 26.35 (10.29 to 54.21) | 26.3 (10.27 to 54.1) |
| 15-19 years | 32.16 (12.48 to 66.88) | 32.15 (12.47 to 66.5) | 32.14 (12.48 to 66.46) | 32.13 (12.47 to 66.52) | 32.14 (12.48 to 66.36) |
| 20-24 years | 39.95 (15.23 to 77.78) | 39.76 (15.19 to 77.24) | 39.51 (15.1 to 76.74) | 39.25 (15.02 to 76.16) | 39.03 (14.9 to 75.87) |
| 25-29 years | 39.21 (16.39 to 73.95) | 39.17 (16.32 to 73.52) | 39.01 (16.26 to 73.28) | 38.8 (16.16 to 72.69) | 38.58 (16.09 to 72.47) |
| 30-34 years | 36.34 (15.35 to 71.12) | 36.4 (15.37 to 71.2) | 36.36 (15.33 to 71.12) | 36.27 (15.25 to 70.96) | 36.18 (15.24 to 70.86) |
| 35-39 years | 36.82 (15.06 to 72.95) | 36.79 (15.06 to 72.76) | 36.67 (15.06 to 72.82) | 36.51 (14.88 to 72.56) | 36.39 (14.83 to 72.23) |
| Sociodemographic index |  |  |  |  |  |
| Low SDI | 33.95 (13.89 to 67.05) | 33.9 (13.87 to 66.86) | 33.76 (13.74 to 66.75) | 33.59 (13.65 to 66.39) | 33.43 (13.58 to 66.11) |
| Low-middle SDI | 32.81 (12.99 to 65.94) | 32.74 (12.94 to 65.64) | 32.59 (12.86 to 65.34) | 32.41 (12.77 to 64.94) | 32.22 (12.63 to 64.75) |
| Middle SDI | 30.54 (12.04 to 61.66) | 30.51 (12.04 to 61.47) | 30.38 (12 to 61.23) | 30.21 (11.93 to 60.77) | 30.05 (11.88 to 60.42) |
| High-middle SDI | 29.12 (11.51 to 58.84) | 29.09 (11.54 to 58.72) | 29.01 (11.5 to 58.5) | 28.91 (11.43 to 58.27) | 28.82 (11.38 to 58.05) |
| High SDI | 24.96 (10.28 to 49.99) | 24.9 (10.23 to 49.85) | 24.86 (10.2 to 49.83) | 24.84 (10.2 to 49.71) | 24.83 (10.2 to 49.77) |
| **Periodontal diseases** |  |  |  |  |  |
| Global | 56.95 (22.03 to 119.91) | 56.89 (22.17 to 120.44) | 56.24 (22.06 to 120.16) | 55.4 (21.85 to 118.98) | 54.78 (21.64 to 117.91) |
| Sex |  |  |  |  |  |
| Female | 55.12 (21.39 to 116.98) | 55.13 (21.55 to 117.75) | 54.61 (21.53 to 117.08) | 53.92 (21.28 to 115.65) | 53.42 (21.1 to 114.6) |
| Male | 58.76 (22.66 to 123.69) | 58.64 (22.78 to 123.4) | 57.86 (22.6 to 122.89) | 56.86 (22.3 to 122.12) | 56.12 (22.12 to 121.01) |
| Age* |  |  |  |  |  |
| 15-19 years | 8.38 (2.55 to 19.22) | 8.41 (2.6 to 19.22) | 8.38 (2.61 to 18.92) | 8.33 (2.62 to 18.56) | 8.33 (2.63 to 18.5) |
| 20-24 years | 28.29 (10.27 to 62.66) | 28.07 (10.32 to 61.57) | 27.5 (10.09 to 59.99) | 26.83 (9.82 to 58.69) | 26.35 (9.66 to 57.73) |
| 25-29 years | 57.57 (22.53 to 122.4) | 57.64 (22.86 to 123.39) | 57.04 (22.88 to 122.29) | 56.15 (22.6 to 119.83) | 55.36 (22.36 to 117.24) |
| 30-34 years | 86.34 (32.27 to 181.74) | 86.62 (32.76 to 182.45) | 86.09 (32.97 to 183.64) | 85.29 (33.12 to 183.94) | 84.72 (33.19 to 183.83) |
| 35-39 years | 116.47 (47.55 to 238.49) | 115.97 (47.34 to 240.8) | 114.31 (46.71 to 241.35) | 112.32 (46.02 to 239.15) | 110.9 (45.2 to 237.4) |
| Sociodemographic index |  |  |  |  |  |
| Low SDI | 92.86 (35.03 to 195.45) | 90.72 (34.6 to 189.87) | 85.96 (33.25 to 180.79) | 80.38 (31.37 to 169.3) | 75.74 (29.77 to 160.98) |
| Low-middle SDI | 84.47 (32.45 to 175.58) | 84.58 (32.74 to 176.04) | 84.37 (32.89 to 175.74) | 84.1 (32.96 to 175.03) | 83.92 (33.13 to 174.56) |
| Middle SDI | 50.64 (19.18 to 106.1) | 50.69 (19.3 to 106.31) | 50.25 (19.26 to 105.84) | 49.65 (19.15 to 104.92) | 49.25 (19.04 to 104.57) |
| High-middle SDI | 36.57 (14.01 to 79.58) | 36.12 (13.96 to 79.63) | 34.86 (13.45 to 78.32) | 33.38 (12.85 to 75.11) | 32.18 (12.23 to 72.74) |
| High SDI | 31.52 (12.18 to 70.38) | 31.66 (12.14 to 71.05) | 32.39 (12.34 to 72.67) | 33.33 (12.65 to 74.98) | 34.12 (12.86 to 76.82) |
| **Edentulism** |  |  |  |  |  |
| Global | 22.97 (12.77 to 36.69) | 23.88 (13.24 to 38.13) | 25.32 (13.96 to 40.34) | 26.92 (14.69 to 43.11) | 28.3 (15.41 to 45.51) |
| Sex |  |  |  |  |  |
| Female | 25.31 (14.15 to 40.17) | 26.15 (14.69 to 41.38) | 27.33 (15.22 to 43.36) | 28.59 (15.89 to 45.51) | 29.72 (16.46 to 47.45) |
| Male | 20.65 (11.28 to 33.26) | 21.64 (11.8 to 34.86) | 23.34 (12.63 to 37.6) | 25.27 (13.49 to 40.78) | 26.89 (14.22 to 43.49) |
| Age* |  |  |  |  |  |
| 20-24 years | 7.16 (3.69 to 12.39) | 7.44 (3.85 to 12.89) | 7.82 (3.95 to 13.48) | 8.22 (4.12 to 14.34) | 8.58 (4.22 to 15.18) |
| 25-29 years | 17.43 (9.44 to 28.36) | 18.33 (9.95 to 29.78) | 19.68 (10.58 to 31.92) | 21.15 (11.19 to 34.55) | 22.38 (11.78 to 36.48) |
| 30-34 years | 27.99 (15.55 to 44.2) | 29.36 (16.25 to 46.32) | 31.5 (17.38 to 49.78) | 33.88 (18.45 to 53.83) | 35.92 (19.59 to 57.51) |
| 35-39 years | 41.79 (23.86 to 65.65) | 42.97 (24.38 to 67.45) | 44.97 (25.49 to 70.28) | 47.22 (26.64 to 74.02) | 49.22 (27.76 to 77.32) |
| Sociodemographic index |  |  |  |  |  |
| Low SDI | 34.66 (19.18 to 55.61) | 36.45 (20.17 to 58.55) | 39.71 (21.66 to 64.1) | 43.36 (23.43 to 70.34) | 46.3 (25.04 to 75.03) |
| Low-middle SDI | 23.98 (13.5 to 38.23) | 26.19 (14.59 to 41.79) | 30.18 (16.34 to 48.83) | 34.65 (18.39 to 56.48) | 38.3 (20.06 to 62.92) |
| Middle SDI | 22.99 (12.68 to 36.48) | 23.73 (13.14 to 37.72) | 24.66 (13.76 to 39.03) | 25.63 (14.27 to 40.79) | 26.51 (14.82 to 42.22) |
| High-middle SDI | 17.96 (9.93 to 28.68) | 18.13 (10.04 to 28.97) | 18.22 (10.08 to 29.02) | 18.28 (9.99 to 29.28) | 18.38 (10.01 to 29.55) |
| High SDI | 19.95 (10.46 to 32.52) | 19.52 (10.31 to 31.95) | 18.56 (9.82 to 30.18) | 17.47 (9.18 to 28.54) | 16.55 (8.77 to 27.02) |
| **Other oral disorders** |  |  |  |  |  |
| Global | 47 (28.41 to 70.84) | 46.98 (28.42 to 70.88) | 46.98 (28.39 to 70.8) | 46.97 (28.36 to 70.83) | 46.98 (28.37 to 70.83) |
| Sex |  |  |  |  |  |
| Female | 50.68 (30.67 to 76.15) | 50.65 (30.67 to 76.2) | 50.63 (30.58 to 76.06) | 50.61 (30.59 to 76.04) | 50.6 (30.66 to 76.07) |
| Male | 43.43 (26.23 to 65.47) | 43.42 (26.18 to 65.39) | 43.43 (26.22 to 65.56) | 43.45 (26.23 to 65.47) | 43.47 (26.23 to 65.47) |
| Age* |  |  |  |  |  |
| 2-4 years | 41.62 (24.82 to 61.83) | 41.62 (24.94 to 61.83) | 41.63 (24.81 to 61.83) | 41.65 (24.82 to 61.96) | 41.66 (24.75 to 62.02) |
| 5-9 years | 30.52 (18.02 to 46.67) | 30.52 (18.11 to 46.89) | 30.52 (18 to 46.54) | 30.52 (18.05 to 46.73) | 30.52 (18 to 46.42) |
| 10-14 years | 32.14 (18.81 to 49.88) | 32.12 (18.83 to 49.79) | 32.1 (18.79 to 49.75) | 32.1 (18.83 to 49.83) | 32.1 (18.84 to 49.92) |
| 15-19 years | 47.94 (28.94 to 73.02) | 47.91 (28.94 to 73.05) | 47.93 (28.85 to 72.95) | 47.89 (28.82 to 73.02) | 47.9 (28.88 to 73.08) |
| 20-24 years | 58.44 (35.54 to 87.07) | 58.43 (35.38 to 87.01) | 58.45 (35.43 to 86.96) | 58.44 (35.27 to 87.25) | 58.43 (35.33 to 87.08) |
| 25-29 years | 63.6 (39.06 to 94.86) | 63.59 (39.11 to 94.97) | 63.59 (39.11 to 94.7) | 63.58 (39.06 to 94.49) | 63.6 (39.14 to 94.9) |
| 30-34 years | 67.04 (40.99 to 100.58) | 67.02 (41.03 to 101.01) | 67.04 (40.98 to 100.88) | 67.04 (40.97 to 100.62) | 67 (40.97 to 100.62) |
| 35-39 years | 68.7 (41.83 to 103.4) | 68.7 (41.8 to 103.09) | 68.68 (41.97 to 103.54) | 68.67 (41.84 to 103.41) | 68.67 (41.83 to 103.29) |
| Sociodemographic index |  |  |  |  |  |
| Low SDI | 46.64 (28.3 to 69.91) | 46.66 (28.3 to 70.22) | 46.69 (28.23 to 69.96) | 46.71 (28.31 to 70.04) | 46.74 (28.23 to 70.04) |
| Low-middle SDI | 46.91 (28.38 to 70.54) | 46.92 (28.42 to 70.63) | 46.94 (28.4 to 70.63) | 46.96 (28.43 to 70.68) | 46.97 (28.47 to 70.84) |
| Middle SDI | 47.27 (28.6 to 71.46) | 47.25 (28.53 to 71.33) | 47.25 (28.52 to 71.38) | 47.25 (28.5 to 71.39) | 47.25 (28.48 to 71.36) |
| High-middle SDI | 47.25 (28.45 to 71.22) | 47.22 (28.56 to 71.18) | 47.22 (28.55 to 71.1) | 47.2 (28.51 to 71.19) | 47.23 (28.46 to 71.38) |
| High SDI | 46.62 (28.17 to 70.09) | 46.52 (28.12 to 70.11) | 46.41 (28 to 69.66) | 46.34 (27.92 to 69.61) | 46.31 (27.91 to 69.51) |
| **Characteristic** | 2010 | 2011 | 2012 | 2013 | 2014 |
| **Caries of deciduous teeth** |  |  |  |  |  |
| Global | 10.13 (4.31 to 19.78) | 10.13 (4.32 to 19.86) | 10.12 (4.31 to 19.78) | 10.11 (4.29 to 19.72) | 10.1 (4.3 to 19.79) |
| Sex |  |  |  |  |  |
| Female | 10.08 (4.28 to 19.71) | 10.08 (4.29 to 19.78) | 10.07 (4.29 to 19.68) | 10.06 (4.27 to 19.63) | 10.05 (4.28 to 19.63) |
| Male | 10.17 (4.32 to 19.85) | 10.18 (4.34 to 19.91) | 10.17 (4.34 to 19.9) | 10.16 (4.3 to 19.81) | 10.15 (4.32 to 19.91) |
| Age* |  |  |  |  |  |
| 2-4 years | 19.4 (8.47 to 38.34) | 19.41 (8.5 to 38.3) | 19.46 (8.52 to 38.24) | 19.51 (8.47 to 38.53) | 19.57 (8.53 to 38.71) |
| 5-9 years | 15.26 (6.59 to 28.9) | 15.24 (6.61 to 29.01) | 15.2 (6.59 to 28.86) | 15.18 (6.55 to 28.64) | 15.16 (6.57 to 28.77) |
| 10-14 years | 2.04 (0.58 to 4.72) | 2.03 (0.58 to 4.71) | 2.01 (0.57 to 4.67) | 1.99 (0.57 to 4.57) | 1.98 (0.56 to 4.6) |
| Sociodemographic index |  |  |  |  |  |
| Low SDI | 9.55 (4.12 to 18.66) | 9.55 (4.14 to 18.73) | 9.56 (4.15 to 18.76) | 9.57 (4.13 to 18.81) | 9.59 (4.15 to 18.93) |
| Low-middle SDI | 10.05 (4.25 to 19.6) | 10.04 (4.22 to 19.85) | 10.03 (4.23 to 19.66) | 10.02 (4.2 to 19.56) | 10.03 (4.25 to 19.69) |
| Middle SDI | 11.12 (4.75 to 21.93) | 11.14 (4.77 to 21.96) | 11.13 (4.74 to 21.88) | 11.09 (4.74 to 21.73) | 11.06 (4.73 to 21.69) |
| High-middle SDI | 11.08 (4.64 to 21.84) | 11.14 (4.64 to 22.15) | 11.13 (4.63 to 21.98) | 11.07 (4.59 to 21.76) | 11.02 (4.56 to 21.64) |
| High SDI | 7.36 (3.06 to 14.27) | 7.37 (3.08 to 14.28) | 7.37 (3.08 to 14.33) | 7.38 (3.08 to 14.33) | 7.4 (3.06 to 14.37) |
| **Caries of permanent teeth** |  |  |  |  |  |
| Global | 30.07 (11.96 to 59.97) | 30.06 (11.95 to 60) | 30.06 (11.91 to 60.01) | 30.05 (11.9 to 60.06) | 30.02 (11.87 to 60.05) |
| Sex |  |  |  |  |  |
| Female | 30.31 (12.05 to 60.43) | 30.29 (12.04 to 60.48) | 30.27 (12.01 to 60.36) | 30.25 (11.99 to 60.38) | 30.22 (11.96 to 60.4) |
| Male | 29.85 (11.87 to 59.57) | 29.85 (11.86 to 59.58) | 29.86 (11.81 to 59.69) | 29.86 (11.81 to 59.78) | 29.84 (11.78 to 59.77) |
| Age* |  |  |  |  |  |
| 5-9 years | 9.05 (2.99 to 21.03) | 8.97 (2.96 to 21.07) | 8.87 (2.92 to 20.9) | 8.77 (2.86 to 20.65) | 8.69 (2.84 to 20.58) |
| 10-14 years | 26.26 (10.24 to 53.96) | 26.2 (10.2 to 53.96) | 26.09 (10.11 to 53.84) | 25.93 (10.02 to 53.64) | 25.78 (9.98 to 53.29) |
| 15-19 years | 32.19 (12.53 to 66.46) | 32.28 (12.56 to 66.66) | 32.37 (12.55 to 66.94) | 32.43 (12.56 to 67.29) | 32.42 (12.51 to 67.18) |
| 20-24 years | 38.91 (14.93 to 75.82) | 38.91 (14.89 to 75.57) | 38.97 (14.84 to 75.43) | 39.07 (14.92 to 75.83) | 39.16 (14.84 to 76.14) |
| 25-29 years | 38.41 (15.99 to 71.98) | 38.32 (15.96 to 71.89) | 38.26 (15.92 to 72.09) | 38.23 (15.88 to 72.1) | 38.21 (15.87 to 72.08) |
| 30-34 years | 36.15 (15.22 to 70.75) | 36.16 (15.23 to 70.9) | 36.18 (15.24 to 70.76) | 36.17 (15.24 to 70.63) | 36.12 (15.19 to 70.67) |
| 35-39 years | 36.4 (14.82 to 72.08) | 36.5 (14.89 to 72.24) | 36.61 (14.86 to 72.51) | 36.73 (14.93 to 72.78) | 36.81 (14.97 to 73) |
| Sociodemographic index |  |  |  |  |  |
| Low SDI | 33.31 (13.5 to 65.97) | 33.21 (13.5 to 65.78) | 33.09 (13.41 to 65.56) | 32.95 (13.32 to 65.35) | 32.82 (13.25 to 64.98) |
| Low-middle SDI | 32.08 (12.65 to 64.25) | 31.94 (12.58 to 64.21) | 31.76 (12.5 to 63.86) | 31.56 (12.4 to 63.57) | 31.36 (12.32 to 63.29) |
| Middle SDI | 29.98 (11.87 to 60.15) | 29.99 (11.84 to 60.25) | 30.03 (11.84 to 60.37) | 30.07 (11.83 to 60.49) | 30.08 (11.82 to 60.58) |
| High-middle SDI | 28.8 (11.41 to 58.02) | 28.84 (11.42 to 58.07) | 28.93 (11.4 to 58.25) | 29.03 (11.47 to 58.58) | 29.1 (11.44 to 58.96) |
| High SDI | 24.83 (10.19 to 49.86) | 24.9 (10.2 to 49.94) | 25.05 (10.26 to 50.21) | 25.23 (10.35 to 50.57) | 25.38 (10.38 to 50.89) |
| **Periodontal diseases** |  |  |  |  |  |
| Global | 54.81 (21.63 to 117.84) | 55.65 (21.97 to 119.35) | 56.9 (22.45 to 122.05) | 58.23 (22.99 to 125.26) | 59.34 (23.44 to 128.07) |
| Sex |  |  |  |  |  |
| Female | 53.5 (21.12 to 114.46) | 54.25 (21.46 to 116.06) | 55.32 (21.89 to 118.68) | 56.44 (22.33 to 121.36) | 57.38 (22.67 to 123.77) |
| Male | 56.11 (22.15 to 121.06) | 57.03 (22.48 to 122.85) | 58.46 (23.01 to 125.79) | 59.99 (23.61 to 129.09) | 61.28 (24.09 to 132.11) |
| Age* |  |  |  |  |  |
| 15-19 years | 8.45 (2.69 to 18.71) | 8.71 (2.81 to 19.04) | 9.04 (2.96 to 19.54) | 9.38 (3.11 to 20.31) | 9.66 (3.23 to 20.86) |
| 20-24 years | 26.33 (9.62 to 57.58) | 26.86 (9.79 to 58.69) | 27.68 (10.12 to 60.39) | 28.62 (10.41 to 62.47) | 29.49 (10.67 to 64.24) |
| 25-29 years | 55.08 (22.15 to 116.19) | 55.36 (22.21 to 116.16) | 55.93 (22.35 to 117.73) | 56.67 (22.54 to 119.97) | 57.4 (22.67 to 122.62) |
| 30-34 years | 84.96 (33.38 to 185.01) | 86.16 (33.88 to 187.34) | 87.79 (34.59 to 190.69) | 89.42 (35.25 to 195.04) | 90.71 (36.05 to 198.9) |
| 35-39 years | 111.02 (45.19 to 236.84) | 113.14 (46.1 to 241.08) | 116.33 (47.3 to 248.16) | 119.64 (48.84 to 255.47) | 122.29 (49.92 to 261.27) |
| Sociodemographic index |  |  |  |  |  |
| Low SDI | 73.86 (29.22 to 156.84) | 73.87 (29.28 to 156.87) | 73.71 (29.2 to 156.75) | 73.49 (29.04 to 156.6) | 73.33 (28.92 to 156.85) |
| Low-middle SDI | 84.08 (33.35 to 175.14) | 84.51 (33.73 to 176.52) | 84.96 (33.79 to 178.02) | 85.37 (33.78 to 179.61) | 85.65 (33.92 to 180.96) |
| Middle SDI | 49.39 (19.15 to 105.21) | 50.34 (19.63 to 107.43) | 51.84 (20.31 to 110.66) | 53.46 (20.99 to 114.16) | 54.79 (21.54 to 117.3) |
| High-middle SDI | 31.82 (11.97 to 72.25) | 32.72 (12.3 to 74.46) | 34.53 (12.98 to 78.62) | 36.61 (13.82 to 83.03) | 38.36 (14.47 to 87.3) |
| High SDI | 34.46 (12.87 to 77.82) | 34.86 (13.02 to 78.99) | 35.72 (13.32 to 81.12) | 36.75 (13.68 to 83.99) | 37.65 (13.98 to 86.28) |
| **Edentulism** |  |  |  |  |  |
| Global | 29.09 (15.8 to 46.96) | 29.56 (16.19 to 47.55) | 30.12 (16.64 to 48.36) | 30.62 (17.03 to 48.94) | 30.93 (17.27 to 49.47) |
| Sex |  |  |  |  |  |
| Female | 30.49 (16.95 to 48.75) | 31.18 (17.42 to 49.76) | 32.12 (18.04 to 51.18) | 33.12 (18.63 to 52.83) | 33.94 (19.01 to 54.16) |
| Male | 27.71 (14.64 to 45.01) | 27.96 (14.87 to 45.27) | 28.15 (15.09 to 45.5) | 28.15 (15.28 to 45.36) | 27.95 (15.29 to 45) |
| Age* |  |  |  |  |  |
| 20-24 years | 8.83 (4.32 to 15.76) | 9.05 (4.45 to 15.89) | 9.31 (4.64 to 16.27) | 9.57 (4.82 to 16.66) | 9.78 (4.84 to 17.12) |
| 25-29 years | 22.98 (12 to 37.43) | 23.15 (12.26 to 37.87) | 23.29 (12.51 to 37.78) | 23.34 (12.49 to 37.83) | 23.32 (12.64 to 38.04) |
| 30-34 years | 37.05 (20.27 to 59.41) | 37.64 (20.76 to 60.32) | 38.34 (21.25 to 61.33) | 38.87 (21.59 to 61.76) | 39.06 (21.85 to 61.82) |
| 35-39 years | 50.49 (28.36 to 79.87) | 51.46 (29.09 to 80.78) | 52.7 (30.01 to 82.89) | 53.92 (31.15 to 84.44) | 54.82 (31.73 to 85.88) |
| Sociodemographic index |  |  |  |  |  |
| Low SDI | 47.49 (25.57 to 77.26) | 47.29 (25.48 to 76.48) | 46.72 (25.3 to 75.91) | 45.94 (24.8 to 74.62) | 45 (24.42 to 72.73) |
| Low-middle SDI | 39.89 (20.76 to 65.67) | 39.93 (20.77 to 65.41) | 39.7 (20.83 to 65.17) | 39.17 (20.75 to 64.14) | 38.46 (20.44 to 62.94) |
| Middle SDI | 27.19 (15.16 to 43.15) | 27.85 (15.6 to 44.13) | 28.71 (16.06 to 45.71) | 29.57 (16.55 to 46.64) | 30.2 (16.94 to 47.62) |
| High-middle SDI | 18.61 (10.07 to 29.92) | 18.96 (10.38 to 30.22) | 19.38 (10.76 to 30.85) | 19.8 (11.04 to 31.22) | 20.14 (11.45 to 31.49) |
| High SDI | 16.21 (8.58 to 26.5) | 16.88 (8.93 to 27.43) | 18.38 (9.79 to 30.06) | 20.19 (10.71 to 32.93) | 21.75 (11.51 to 35.38) |
| **Other oral disorders** |  |  |  |  |  |
| Global | 47 (28.39 to 70.88) | 47.03 (28.39 to 70.83) | 47.04 (28.43 to 70.97) | 47.04 (28.42 to 70.9) | 47.05 (28.42 to 70.91) |
| Sex |  |  |  |  |  |
| Female | 50.61 (30.55 to 76.16) | 50.64 (30.62 to 76.18) | 50.65 (30.59 to 76.27) | 50.65 (30.59 to 76.24) | 50.65 (30.66 to 76.33) |
| Male | 43.49 (26.29 to 65.48) | 43.52 (26.31 to 65.44) | 43.53 (26.3 to 65.66) | 43.54 (26.23 to 65.52) | 43.55 (26.26 to 65.71) |
| Age* |  |  |  |  |  |
| 2-4 years | 41.66 (24.88 to 62.03) | 41.68 (24.91 to 61.97) | 41.69 (24.89 to 61.97) | 41.7 (24.91 to 62.17) | 41.72 (25.01 to 62.03) |
| 5-9 years | 30.52 (17.99 to 46.72) | 30.52 (17.98 to 46.76) | 30.52 (18.01 to 46.65) | 30.54 (18.12 to 46.83) | 30.55 (18.15 to 46.92) |
| 10-14 years | 32.09 (18.87 to 49.97) | 32.09 (18.8 to 49.9) | 32.11 (18.91 to 49.96) | 32.11 (18.87 to 49.56) | 32.14 (18.93 to 49.57) |
| 15-19 years | 47.88 (28.9 to 72.84) | 47.89 (28.76 to 73.21) | 47.87 (28.8 to 72.98) | 47.88 (28.71 to 72.8) | 47.9 (28.64 to 73.17) |
| 20-24 years | 58.43 (35.37 to 87.27) | 58.44 (35.36 to 87.01) | 58.44 (35.49 to 87.17) | 58.44 (35.2 to 86.78) | 58.44 (35.26 to 86.76) |
| 25-29 years | 63.64 (39.04 to 94.71) | 63.66 (39.13 to 94.64) | 63.67 (39.01 to 95.16) | 63.7 (39.16 to 95.11) | 63.71 (39.13 to 95.03) |
| 30-34 years | 67.03 (40.97 to 100.68) | 67.03 (41 to 100.54) | 67.05 (41.1 to 100.83) | 67.06 (40.96 to 100.83) | 67.08 (41.05 to 100.71) |
| 35-39 years | 68.69 (41.84 to 103.29) | 68.71 (41.79 to 102.64) | 68.69 (41.88 to 103.23) | 68.7 (42.05 to 103.42) | 68.69 (41.87 to 103.42) |
| Sociodemographic index |  |  |  |  |  |
| Low SDI | 46.77 (28.24 to 70.36) | 46.79 (28.37 to 70.15) | 46.81 (28.34 to 70.25) | 46.83 (28.41 to 70.26) | 46.85 (28.45 to 70.35) |
| Low-middle SDI | 46.97 (28.39 to 70.83) | 46.98 (28.41 to 70.58) | 46.99 (28.46 to 70.81) | 47 (28.38 to 70.67) | 47.02 (28.45 to 70.88) |
| Middle SDI | 47.28 (28.53 to 71.32) | 47.31 (28.55 to 71.45) | 47.31 (28.59 to 71.53) | 47.29 (28.58 to 71.45) | 47.27 (28.51 to 71.28) |
| High-middle SDI | 47.28 (28.5 to 71.38) | 47.35 (28.56 to 71.33) | 47.35 (28.61 to 71.3) | 47.3 (28.49 to 71.36) | 47.27 (28.53 to 71.3) |
| High SDI | 46.32 (27.97 to 69.59) | 46.37 (27.91 to 69.67) | 46.41 (27.96 to 69.85) | 46.48 (27.96 to 69.93) | 46.53 (27.97 to 70.11) |
| **Characteristic** | 2015 | 2016 | 2017 | 2018 | 2019 |
| **Caries of deciduous teeth** |  |  |  |  |  |
| Global | 10.1 (4.31 to 19.73) | 10.1 (4.31 to 19.77) | 10.12 (4.31 to 19.86) | 10.16 (4.32 to 19.94) | 10.2 (4.33 to 19.98) |
| Sex |  |  |  |  |  |
| Female | 10.05 (4.3 to 19.62) | 10.05 (4.3 to 19.66) | 10.06 (4.29 to 19.76) | 10.1 (4.29 to 19.83) | 10.13 (4.31 to 19.87) |
| Male | 10.15 (4.32 to 19.81) | 10.15 (4.32 to 19.85) | 10.18 (4.32 to 19.95) | 10.22 (4.34 to 20.04) | 10.26 (4.37 to 20.08) |
| Age* |  |  |  |  |  |
| 2-4 years | 19.58 (8.52 to 38.69) | 19.59 (8.53 to 38.75) | 19.61 (8.5 to 38.8) | 19.64 (8.49 to 38.9) | 19.66 (8.52 to 38.84) |
| 5-9 years | 15.14 (6.6 to 28.61) | 15.15 (6.59 to 28.74) | 15.17 (6.58 to 28.91) | 15.2 (6.59 to 28.95) | 15.22 (6.59 to 28.97) |
| 10-14 years | 1.97 (0.56 to 4.57) | 1.96 (0.57 to 4.5) | 1.94 (0.56 to 4.48) | 1.93 (0.55 to 4.49) | 1.92 (0.54 to 4.44) |
| Sociodemographic index |  |  |  |  |  |
| Low SDI | 9.59 (4.13 to 18.81) | 9.56 (4.16 to 18.81) | 9.5 (4.11 to 18.7) | 9.45 (4.11 to 18.63) | 9.42 (4.06 to 18.56) |
| Low-middle SDI | 10.01 (4.26 to 19.61) | 10.02 (4.24 to 19.74) | 10.05 (4.24 to 19.82) | 10.09 (4.28 to 19.87) | 10.11 (4.29 to 19.76) |
| Middle SDI | 11.05 (4.75 to 21.65) | 11.06 (4.74 to 21.72) | 11.09 (4.74 to 21.81) | 11.15 (4.77 to 21.97) | 11.22 (4.78 to 22.13) |
| High-middle SDI | 11.02 (4.56 to 21.68) | 11.03 (4.57 to 21.8) | 11.06 (4.59 to 21.91) | 11.14 (4.61 to 22.05) | 11.24 (4.65 to 22.14) |
| High SDI | 7.41 (3.07 to 14.37) | 7.46 (3.08 to 14.46) | 7.54 (3.1 to 14.6) | 7.67 (3.15 to 14.89) | 7.82 (3.21 to 15.14) |
| **Caries of permanent teeth** |  |  |  |  |  |
| Global | 29.97 (11.85 to 60.06) | 29.8 (11.77 to 59.74) | 29.5 (11.65 to 59.22) | 29.2 (11.54 to 58.67) | 29.04 (11.46 to 58.46) |
| Sex |  |  |  |  |  |
| Female | 30.17 (11.95 to 60.41) | 30.01 (11.88 to 60.1) | 29.71 (11.75 to 59.53) | 29.41 (11.62 to 59.02) | 29.25 (11.54 to 58.7) |
| Male | 29.79 (11.74 to 59.78) | 29.61 (11.66 to 59.46) | 29.3 (11.56 to 58.94) | 29 (11.45 to 58.39) | 28.83 (11.38 to 58.27) |
| Age* |  |  |  |  |  |
| 5-9 years | 8.66 (2.84 to 20.59) | 8.67 (2.85 to 20.47) | 8.73 (2.86 to 20.51) | 8.78 (2.9 to 20.67) | 8.79 (2.92 to 20.65) |
| 10-14 years | 25.64 (9.89 to 53) | 25.41 (9.82 to 52.59) | 25.06 (9.75 to 52.22) | 24.71 (9.62 to 51.55) | 24.48 (9.51 to 51.39) |
| 15-19 years | 32.32 (12.46 to 67.31) | 32 (12.32 to 66.62) | 31.45 (12.02 to 65.46) | 30.93 (11.82 to 64.35) | 30.66 (11.67 to 63.89) |
| 20-24 years | 39.18 (14.86 to 76.21) | 38.99 (14.8 to 75.88) | 38.58 (14.69 to 75.08) | 38.17 (14.5 to 74.23) | 37.98 (14.44 to 73.87) |
| 25-29 years | 38.19 (15.84 to 72.38) | 38.03 (15.77 to 72) | 37.74 (15.67 to 71.65) | 37.46 (15.56 to 71.21) | 37.34 (15.52 to 71.06) |
| 30-34 years | 36.02 (15.15 to 70.44) | 35.76 (15.04 to 70.17) | 35.37 (14.82 to 69.36) | 34.98 (14.64 to 68.7) | 34.78 (14.48 to 68.53) |
| 35-39 years | 36.85 (14.98 to 73.15) | 36.78 (14.91 to 73.1) | 36.54 (14.84 to 72.74) | 36.26 (14.74 to 72.43) | 36.09 (14.68 to 72.23) |
| Sociodemographic index |  |  |  |  |  |
| Low SDI | 32.71 (13.2 to 64.77) | 32.54 (13.16 to 64.53) | 32.27 (13.04 to 64.22) | 31.99 (12.94 to 63.75) | 31.81 (12.85 to 63.6) |
| Low-middle SDI | 31.16 (12.24 to 63.02) | 30.83 (12.08 to 62.28) | 30.34 (11.86 to 61.27) | 29.87 (11.64 to 60.37) | 29.62 (11.53 to 60.13) |
| Middle SDI | 30.05 (11.8 to 60.58) | 29.86 (11.71 to 60.21) | 29.5 (11.57 to 59.48) | 29.14 (11.44 to 58.83) | 28.94 (11.36 to 58.54) |
| High-middle SDI | 29.13 (11.46 to 59.08) | 29.05 (11.4 to 58.88) | 28.86 (11.33 to 58.52) | 28.65 (11.23 to 57.97) | 28.55 (11.13 to 57.74) |
| High SDI | 25.45 (10.38 to 51.03) | 25.41 (10.38 to 50.91) | 25.31 (10.34 to 50.87) | 25.2 (10.28 to 50.65) | 25.14 (10.23 to 50.43) |
| **Periodontal diseases** |  |  |  |  |  |
| Global | 60 (23.68 to 129.63) | 60.06 (23.58 to 129.73) | 59.78 (23.35 to 129.45) | 59.48 (23.08 to 129.17) | 59.5 (22.97 to 129.17) |
| Sex |  |  |  |  |  |
| Female | 57.95 (22.76 to 125.02) | 58.15 (22.76 to 125.81) | 58.16 (22.66 to 126.06) | 58.17 (22.57 to 126.17) | 58.37 (22.57 to 126.53) |
| Male | 62.01 (24.45 to 133.87) | 61.94 (24.39 to 133.79) | 61.37 (24.01 to 132.97) | 60.76 (23.59 to 132.07) | 60.61 (23.35 to 131.89) |
| Age* |  |  |  |  |  |
| 15-19 years | 9.82 (3.31 to 21.27) | 9.68 (3.29 to 20.98) | 9.27 (3.16 to 19.76) | 8.85 (3 to 18.92) | 8.67 (2.95 to 18.53) |
| 20-24 years | 30.13 (10.91 to 65.01) | 30.09 (10.87 to 64.37) | 29.4 (10.6 to 64.11) | 28.63 (10.36 to 62.66) | 28.39 (10.32 to 61.48) |
| 25-29 years | 57.93 (22.6 to 124.66) | 57.99 (22.21 to 125.31) | 57.71 (21.59 to 125.88) | 57.51 (21.46 to 127.54) | 57.84 (21.48 to 128.99) |
| 30-34 years | 91.33 (36.68 to 201.1) | 91.19 (36.78 to 201.7) | 90.75 (36.46 to 201.09) | 90.32 (35.99 to 200.71) | 90.25 (35.69 to 201.05) |
| 35-39 years | 123.75 (50.26 to 263.92) | 124.4 (50.09 to 264.18) | 124.89 (50.35 to 264.39) | 125.29 (49.97 to 264.09) | 125.63 (49.72 to 263.95) |
| Sociodemographic index |  |  |  |  |  |
| Low SDI | 73.38 (28.97 to 157.05) | 73.35 (29.09 to 156.93) | 73.05 (28.96 to 156.44) | 72.72 (28.81 to 155.61) | 72.61 (28.72 to 155.28) |
| Low-middle SDI | 85.76 (33.98 to 181.87) | 85.26 (33.45 to 181.49) | 84.13 (32.77 to 179.23) | 83.02 (32.02 to 178.05) | 82.55 (31.47 to 179.19) |
| Middle SDI | 55.5 (21.82 to 119.02) | 55.49 (21.78 to 119.29) | 55.12 (21.64 to 118.38) | 54.73 (21.5 to 117.54) | 54.66 (21.42 to 117.79) |
| High-middle SDI | 39.23 (14.78 to 89.55) | 39.33 (14.76 to 90.26) | 39.33 (14.71 to 90.53) | 39.3 (14.6 to 90.51) | 39.38 (14.53 to 90.43) |
| High SDI | 38.12 (14.1 to 87.21) | 38.25 (14.1 to 87.61) | 38.34 (14.15 to 87.6) | 38.4 (14.13 to 87.83) | 38.45 (14.12 to 88.1) |
| **Edentulism** |  |  |  |  |  |
| Global | 30.87 (17.24 to 49.44) | 29.32 (16.38 to 46.72) | 26.26 (14.71 to 41.64) | 23.23 (13.01 to 36.77) | 21.82 (12.22 to 34.42) |
| Sex |  |  |  |  |  |
| Female | 34.36 (19.29 to 54.8) | 32.83 (18.52 to 52.24) | 29.23 (16.48 to 46.13) | 25.54 (14.43 to 40.09) | 23.82 (13.44 to 37.25) |
| Male | 27.43 (15.17 to 44.05) | 25.87 (14.27 to 41.49) | 23.34 (12.92 to 37.26) | 20.96 (11.6 to 33.49) | 19.85 (10.99 to 31.59) |
| Age |  |  |  |  |  |
| 20-24 years | 9.89 (4.94 to 17.34) | 9.26 (4.65 to 16.19) | 7.79 (3.91 to 13.53) | 6.27 (3.17 to 10.91) | 5.58 (2.72 to 9.53) |
| 25-29 years | 23.13 (12.54 to 37.83) | 21.68 (11.67 to 35.04) | 18.83 (10.06 to 30.48) | 16 (8.45 to 25.92) | 14.72 (7.75 to 23.69) |
| 30-34 years | 38.69 (21.52 to 61.24) | 36.37 (20.57 to 57.2) | 32.19 (18.28 to 50.45) | 28.19 (16.12 to 44.11) | 26.33 (15.09 to 41.4) |
| 35-39 years | 55.06 (31.92 to 86.36) | 53.2 (30.54 to 83.38) | 49.28 (28.38 to 76.71) | 45.33 (25.96 to 70.51) | 43.43 (24.96 to 67.3) |
| Sociodemographic index |  |  |  |  |  |
| Low SDI | 44.09 (23.72 to 71.39) | 41.69 (22.43 to 67.97) | 37.61 (20.25 to 61.26) | 33.77 (17.93 to 55.23) | 32.06 (17.12 to 52.36) |
| Low-middle SDI | 37.55 (20.25 to 61.28) | 34.44 (18.81 to 55.49) | 28.88 (15.92 to 46.24) | 23.51 (12.97 to 37.3) | 20.93 (11.52 to 33.39) |
| Middle SDI | 30.37 (17.16 to 47.93) | 28.93 (16.41 to 45.4) | 25.91 (14.77 to 40.5) | 22.88 (13 to 35.78) | 21.47 (12.28 to 33.5) |
| High-middle SDI | 20.31 (11.61 to 31.72) | 19.96 (11.37 to 31.36) | 19.09 (11.03 to 29.81) | 18.14 (10.46 to 28.25) | 17.64 (10.24 to 27.45) |
| High SDI | 22.45 (11.88 to 36.63) | 22.07 (11.75 to 35.98) | 21.02 (11.04 to 34.31) | 19.98 (10.48 to 32.72) | 19.53 (10.15 to 31.99) |
| **Other oral disorders** |  |  |  |  |  |
| Global | 47.05 (28.44 to 70.83) | 47.07 (28.44 to 70.87) | 47.1 (28.51 to 70.92) | 47.15 (28.51 to 71.06) | 47.18 (28.53 to 71.03) |
| Sex |  |  |  |  |  |
| Female | 50.67 (30.62 to 76.18) | 50.69 (30.73 to 76.26) | 50.74 (30.71 to 76.4) | 50.79 (30.7 to 76.5) | 50.83 (30.71 to 76.59) |
| Male | 43.56 (26.31 to 65.53) | 43.57 (26.33 to 65.54) | 43.59 (26.33 to 65.68) | 43.62 (26.34 to 65.67) | 43.66 (26.39 to 65.74) |
| Age* |  |  |  |  |  |
| 2-4 years | 41.74 (24.93 to 62.05) | 41.74 (25.05 to 62) | 41.75 (25.06 to 62.33) | 41.76 (24.91 to 61.9) | 41.75 (24.97 to 61.89) |
| 5-9 years | 30.56 (18.14 to 46.68) | 30.57 (18.05 to 46.68) | 30.58 (18.13 to 46.7) | 30.59 (18.04 to 46.92) | 30.6 (18.11 to 46.92) |
| 10-14 years | 32.15 (18.93 to 49.46) | 32.15 (18.9 to 49.42) | 32.16 (18.95 to 49.71) | 32.2 (18.97 to 49.63) | 32.18 (18.91 to 49.73) |
| 15-19 years | 47.9 (28.73 to 72.98) | 47.92 (28.79 to 73.05) | 47.91 (28.82 to 72.97) | 47.93 (28.82 to 73.02) | 47.94 (28.87 to 72.92) |
| 20-24 years | 58.43 (35.22 to 87.24) | 58.42 (35.32 to 86.97) | 58.44 (35.39 to 86.8) | 58.46 (35.34 to 87.38) | 58.47 (35.47 to 86.86) |
| 25-29 years | 63.73 (39.17 to 95.06) | 63.73 (39.13 to 94.96) | 63.74 (39.38 to 95.16) | 63.73 (39.32 to 94.94) | 63.7 (39.22 to 94.66) |
| 30-34 years | 67.11 (41.14 to 100.2) | 67.13 (41.19 to 100.96) | 67.17 (41.09 to 100.79) | 67.19 (41.08 to 100.95) | 67.19 (41.09 to 100.8) |
| 35-39 years | 68.67 (41.92 to 103.25) | 68.7 (41.79 to 103.21) | 68.76 (41.85 to 102.98) | 68.81 (42.08 to 103.5) | 68.84 (41.95 to 103.8) |
| Sociodemographic index |  |  |  |  |  |
| Low SDI | 46.88 (28.4 to 70.18) | 46.88 (28.38 to 70.39) | 46.9 (28.4 to 70.5) | 46.9 (28.34 to 70.51) | 46.91 (28.38 to 70.38) |
| Low-middle SDI | 47.03 (28.41 to 70.79) | 47.05 (28.51 to 70.72) | 47.07 (28.57 to 70.86) | 47.09 (28.53 to 70.87) | 47.08 (28.45 to 70.88) |
| Middle SDI | 47.27 (28.49 to 71.33) | 47.27 (28.56 to 71.33) | 47.29 (28.57 to 71.43) | 47.34 (28.58 to 71.52) | 47.41 (28.7 to 71.56) |
| High-middle SDI | 47.26 (28.54 to 71.04) | 47.25 (28.44 to 71.38) | 47.28 (28.48 to 71.26) | 47.36 (28.52 to 71.62) | 47.45 (28.61 to 71.67) |
| High SDI | 46.57 (28.07 to 70.12) | 46.65 (28.14 to 70.2) | 46.78 (28.24 to 70.38) | 46.91 (28.31 to 70.55) | 46.98 (28.3 to 70.75) |
| **Characteristic** | 2020 | 2021 |  |  |  |
| **Caries of deciduous teeth** |  |  |  |  |  |
| Global | 10.28 (4.39 to 20.18) | 10.16 (4.42 to 20.02) |  |  |  |
| Sex |  |  |  |  |  |
| Female | 10.19 (4.38 to 19.99) | 10.1 (4.39 to 19.92) |  |  |  |
| Male | 10.36 (4.4 to 20.35) | 10.22 (4.45 to 20.11) |  |  |  |
| Age* |  |  |  |  |  |
| 2-4 years | 19.89 (8.52 to 39.5) | 19.74 (8.57 to 38.87) |  |  |  |
| 5-9 years | 15.29 (6.72 to 29.12) | 15.2 (6.82 to 29.3) |  |  |  |
| 10-14 years | 1.88 (0.54 to 4.39) | 1.75 (0.51 to 4.09) |  |  |  |
| Sociodemographic index |  |  |  |  |  |
| Low SDI | 9.45 (4.06 to 18.71) | 9.41 (4.1 to 18.73) |  |  |  |
| Low-middle SDI | 10.1 (4.36 to 19.93) | 9.82 (4.26 to 19.75) |  |  |  |
| Middle SDI | 11.29 (4.82 to 22.31) | 11.27 (4.83 to 22.27) |  |  |  |
| High-middle SDI | 11.29 (4.71 to 22.27) | 11.22 (4.65 to 22.28) |  |  |  |
| High SDI | 8.44 (3.51 to 16.38) | 8.46 (3.51 to 16.35) |  |  |  |
| **Caries of permanent teeth** |  |  |  |  |  |
| Global | 29.01 (11.48 to 58.28) | 28.86 (11.38 to 57.95) |  |  |  |
| Sex |  |  |  |  |  |
| Female | 29.19 (11.58 to 58.51) | 29.04 (11.48 to 58.23) |  |  |  |
| Male | 28.85 (11.36 to 58.11) | 28.7 (11.28 to 57.68) |  |  |  |
| Age* |  |  |  |  |  |
| 5-9 years | 8.74 (2.88 to 20.37) | 8.13 (2.66 to 19.26) |  |  |  |
| 10-14 years | 24.46 (9.53 to 51.16) | 24.04 (9.25 to 50.05) |  |  |  |
| 15-19 years | 30.64 (11.68 to 63.96) | 30.77 (11.68 to 64) |  |  |  |
| 20-24 years | 37.97 (14.53 to 73.68) | 38.07 (14.53 to 74.38) |  |  |  |
| 25-29 years | 37.42 (15.57 to 71.1) | 37.46 (15.56 to 71.11) |  |  |  |
| 30-34 years | 34.79 (14.52 to 68.59) | 34.76 (14.52 to 67.97) |  |  |  |
| 35-39 years | 35.94 (14.64 to 71.48) | 35.81 (14.54 to 71.56) |  |  |  |
| Sociodemographic index |  |  |  |  |  |
| Low SDI | 31.62 (12.77 to 62.97) | 31.74 (12.88 to 63.24) |  |  |  |
| Low-middle SDI | 29.65 (11.62 to 60.06) | 29.47 (11.43 to 59.45) |  |  |  |
| Middle SDI | 28.85 (11.29 to 58.27) | 28.54 (11.2 to 57.88) |  |  |  |
| High-middle SDI | 28.65 (11.23 to 58.2) | 28.26 (11.05 to 57.24) |  |  |  |
| High SDI | 25.1 (10.17 to 50.58) | 25.19 (10.28 to 50.61) |  |  |  |
| **Periodontal diseases** |  |  |  |  |  |
| Global | 59.73 (22.97 to 129.97) | 59.89 (22.95 to 131.1) |  |  |  |
| Sex |  |  |  |  |  |
| Female | 58.57 (22.53 to 127.34) | 58.74 (22.56 to 128.32) |  |  |  |
| Male | 60.86 (23.41 to 132.66) | 61.01 (23.35 to 133.83) |  |  |  |
| Age* |  |  |  |  |  |
| 15-19 years | 8.71 (2.95 to 18.76) | 8.7 (2.95 to 18.86) |  |  |  |
| 20-24 years | 28.6 (10.4 to 61.83) | 28.65 (10.4 to 61.96) |  |  |  |
| 25-29 years | 58.56 (21.75 to 130.59) | 59.08 (21.9 to 132.76) |  |  |  |
| 30-34 years | 90.43 (35.56 to 203.22) | 90.84 (35.38 to 205.2) |  |  |  |
| 35-39 years | 125.59 (49.52 to 263.6) | 125.44 (49.43 to 265.01) |  |  |  |
| Sociodemographic index |  |  |  |  |  |
| Low SDI | 72.73 (28.81 to 155.96) | 72.71 (28.68 to 155.09) |  |  |  |
| Low-middle SDI | 82.7 (31.54 to 180.41) | 82.54 (31.38 to 181) |  |  |  |
| Middle SDI | 54.68 (21.41 to 118.65) | 54.69 (21.24 to 119.73) |  |  |  |
| High-middle SDI | 39.31 (14.39 to 90.77) | 39.5 (14.49 to 90.77) |  |  |  |
| High SDI | 38.36 (14 to 87.95) | 38.23 (13.93 to 88.12) |  |  |  |
| **Edentulism** |  |  |  |  |  |
| Global | 21.81 (12.27 to 34.32) | 21.6 (12.17 to 33.84) |  |  |  |
| Sex |  |  |  |  |  |
| Female | 23.82 (13.52 to 37.31) | 23.58 (13.36 to 36.66) |  |  |  |
| Male | 19.83 (11 to 31.46) | 19.67 (10.96 to 31.19) |  |  |  |
| Age* |  |  |  |  |  |
| 20-24 years | 5.6 (2.75 to 9.58) | 5.55 (2.76 to 9.42) |  |  |  |
| 25-29 years | 14.82 (7.79 to 23.78) | 14.74 (7.8 to 23.5) |  |  |  |
| 30-34 years | 26.26 (15.09 to 41.24) | 26.08 (14.95 to 40.87) |  |  |  |
| 35-39 years | 43.32 (25.11 to 66.88) | 42.78 (24.81 to 65.69) |  |  |  |
| Sociodemographic index |  |  |  |  |  |
| Low SDI | 32.04 (17.14 to 51.65) | 31.25 (17.14 to 50.37) |  |  |  |
| Low-middle SDI | 20.72 (11.45 to 33) | 20.37 (11.38 to 32.36) |  |  |  |
| Middle SDI | 21.49 (12.29 to 33.34) | 21.29 (12.16 to 32.93) |  |  |  |
| High-middle SDI | 17.54 (10.22 to 27.22) | 17.5 (10.13 to 27.04) |  |  |  |
| High SDI | 19.61 (10.26 to 32.13) | 19.7 (10.31 to 32.23) |  |  |  |
| **Other oral disorders** |  |  |  |  |  |
| Global | 47.17 (28.52 to 71.09) | 47.16 (28.52 to 71.02) |  |  |  |
| Sex |  |  |  |  |  |
| Female | 50.81 (30.79 to 76.33) | 50.79 (30.7 to 76.38) |  |  |  |
| Male | 43.66 (26.32 to 65.73) | 43.66 (26.39 to 65.78) |  |  |  |
| Age* |  |  |  |  |  |
| 2-4 years | 41.76 (25.01 to 62.21) | 41.77 (25.03 to 62.15) |  |  |  |
| 5-9 years | 30.61 (18.06 to 46.9) | 30.6 (17.96 to 46.92) |  |  |  |
| 10-14 years | 32.2 (19 to 50.06) | 32.19 (18.92 to 49.8) |  |  |  |
| 15-19 years | 47.89 (28.79 to 72.84) | 47.88 (28.88 to 72.68) |  |  |  |
| 20-24 years | 58.39 (35.55 to 86.86) | 58.36 (35.32 to 86.52) |  |  |  |
| 25-29 years | 63.64 (39.05 to 94.91) | 63.57 (39.27 to 94.86) |  |  |  |
| 30-34 years | 67.14 (41.18 to 100.76) | 67.07 (41.21 to 100.65) |  |  |  |
| 35-39 years | 68.77 (41.72 to 103.24) | 68.73 (41.74 to 103.56) |  |  |  |
| Sociodemographic index |  |  |  |  |  |
| Low SDI | 46.89 (28.33 to 70.49) | 46.86 (28.36 to 70.34) |  |  |  |
| Low-middle SDI | 47.07 (28.52 to 70.91) | 47.05 (28.49 to 70.77) |  |  |  |
| Middle SDI | 47.43 (28.59 to 71.61) | 47.43 (28.6 to 71.57) |  |  |  |
| High-middle SDI | 47.49 (28.61 to 71.87) | 47.49 (28.64 to 71.78) |  |  |  |
| High SDI | 46.94 (28.33 to 70.71) | 46.94 (28.32 to 70.58) |  |  |  |

^*^Rate for age groups.

## Table F. The age-standardized incidence, prevalence, and YLD rates per 100,000 populations of other oral disorders, across different sexes, age groups, and SDI quintiles, in 1990 and 2021, as well as the AAPCs in the period 1990-2021.

| Characteristic | 1990 | | | 2021 | | | AAPC | | |  |
| --- | --- | --- | --- | --- | --- | --- | --- | --- | --- | --- |
|  | ASIR | ASPR | ASYLDR | ASIR | ASPR | ASYLDR | ASIR, No. | ASPR, No. | ASYLDR, No. (95% CI) |  |
|  |  |  |  |  |  |  |  |  |  |  |
|  |  |  |  |  |  |  |  |  |  |  |
| Global | - | 1603.13 | 47.01 | - | 1606.79 | 47.16 | - | 0.01 | 0.01 |  |
|  |  | (1413.17-1797.73) | (28.47-70.86) |  | (1415.88-1801.16) | (28.52-71.02) |  | (0 to 0.01) | (0 to 0.02) |  |
| Sex |  |  |  |  |  |  |  |  |  |  |
| Female | - | 1737.52 | 50.76 | - | 1738.33 | 50.79 | - | 0 | 0 |  |
|  |  | (1525.21-1959.53) | (30.76-76.33) |  | (1527.55-1959.37) | (30.7-76.38) |  | (0 to 0.01) | (0 to 0.01) |  |
| Male | - | 1472.76 | 43.37 | - | 1480.07 | 43.66 | - | 0.02 | 0.02 |  |
|  |  | (1294.32-1658.72) | (26.19-65.31) |  | (1300.59-1667.93) | (26.39-65.78) |  | (0.01 to 0.02) | (0.02 to 0.03) |  |
| Age* |  |  |  |  |  |  |  |  |  |  |
| 2-4 years | - | 1405.2 | 41.57 | - | 1405.38 | 41.77 | - | 0 | 0.02 |  |
|  |  | (1237.58-1594.86)^a^ | (24.93-61.63)^a^ |  | (1237.97-1593.52)^a^ | (25.03-62.15)^a^ |  | (0 to 0) | (0.01 to 0.02) |  |
| 5-9 years | - | 1030.26 | 30.54 | - | 1029.44 | 30.6 | - | 0 | 0.01 |  |
|  |  | (883.06-1181.56)^a^ | (18.06-46.79)^a^ |  | (881.59-1180.36)^a^ | (17.96-46.92)^a^ |  | (0 to 0) | (0 to 0.01) |  |
| 10-14 years | - | 1087.75 | 32.19 | - | 1086.42 | 32.19 | - | 0 | 0 |  |
|  |  | (931.73-1250.87)^a^ | (18.98-49.93)^a^ |  | (930.6-1248.23)^a^ | (18.92-49.8)^a^ |  | (-0.01 to 0) | (0 to 0.01) |  |
| 15-19 years | - | 1626.03 | 47.91 | - | 1624.59 | 47.88 | - | 0 | 0 |  |
|  |  | (1430.07-1818.45)^a^ | (28.9-72.88)^a^ |  | (1427.86-1817.72)^a^ | (28.88-72.68)^a^ |  | (0 to 0) | (-0.01 to 0) |  |
| 20-24 years | - | 1989.56 | 58.4 | - | 1988.26 | 58.36 | - | 0 | 0 |  |
|  |  | (1783.16-2193.85)^a^ | (35.52-87.2)^a^ |  | (1781.19-2186.28)^a^ | (35.32-86.52)^a^ |  | (0 to 0.01) | (-0.01 to 0) |  |
| 25-29 years | - | 2176.8 | 63.63 | - | 2175.39 | 63.57 | - | 0 | 0 |  |
|  |  | (1931.32-2410.88)^a^ | (39.21-94.55)^a^ |  | (1927.01-2408.64)^a^ | (39.27-94.86)^a^ |  | (0 to 0) | (-0.01 to 0) |  |
| 30-34 years | - | 2301.81 | 67 | - | 2302.8 | 67.07 | - | 0 | 0 |  |
|  |  | (2037.77-2565.53)^a^ | (41.01-100.44)^a^ |  | (2038.31-2568.17)^a^ | (41.21-100.65)^a^ |  | (0 to 0) | (0 to 0.01) |  |
| 35-39 years | - | 2368.38 | 68.68 | - | 2370.25 | 68.73 | - | 0 | 0 |  |
|  |  | (2107.21-2660.01)^a^ | (41.85-103.75)^a^ |  | (2110.51-2663.34)^a^ | (41.74-103.56)^a^ |  | (0 to 0.01) | (0 to 0.01) |  |
| Sociodemographic index |  |  |  |  |  |  |  |  |  |  |
| Low SDI | - | 1599.4 | 46.5 | - | 1603.58 | 46.86 | - | 0.01 | 0.03 |  |
|  |  | (1410.54-1793.13) | (28.33-69.74) |  | (1414.53-1797.57) | (28.36-70.34) |  | (0.01 to 0.01) | (0.02 to 0.03) |  |
| Low-middle SDI | - | 1603.37 | 46.81 | - | 1606.57 | 47.05 | - | 0.01 | 0.02 |  |
|  |  | (1412.44-1800.86) | (28.43-70.4) |  | (1415.31-1803.98) | (28.49-70.77) |  | (0.01 to 0.01) | (0.01 to 0.02) |  |
| Middle SDI | - | 1606.39 | 47.21 | - | 1612.28 | 47.43 | - | 0.01 | 0.01 |  |
|  |  | (1411.01-1811.88) | (28.58-71.26) |  | (1416.89-1817.95) | (28.6-71.57) |  | (0.01 to 0.02) | (0 to 0.02) |  |
| High-middle SDI | - | 1605.97 | 47.28 | - | 1611.41 | 47.49 | - | 0.01 | 0.01 |  |
|  |  | (1414.62-1801.98) | (28.49-71.41) |  | (1419.25-1806.42) | (28.64-71.78) |  | (0.01 to 0.02) | (0 to 0.03) |  |
| High SDI | - | 1597.55 | 46.93 | - | 1599.12 | 46.94 | - | 0 | 0 |  |
|  |  | (1416.4-1784.71) | (28.25-70.76) |  | (1418.51-1786.55) | (28.32-70.58) |  | (-0.01 to 0.02) | (-0.02 to 0.01) |  |

^a^Rate for age groups.

## Table G. Data of decomposition analysis for oral disorders across SDI quintiles between 1990 and 2021

| **Location** | **Sex** | **Cause** | **Measure** | **Overll difference** | **Aging** | **Population** | **Epidemiological change** |
| --- | --- | --- | --- | --- | --- | --- | --- |
| Global | Both | Caries of deciduous teeth | Prevalence | 32034490.21 | -11960013.5  (-37.33%) | 74180068.59  (231.56%) | -30185564.87  (-94.23%) |
| Global | Both | Caries of deciduous teeth | Incidence | 86308120.46 | 3195668.93  (3.7%) | 176579932.01  (204.59%) | -93467480.48  (-108.3%) |
| Global | Both | Caries of deciduous teeth | YLDs (Years Lived with Disability) | 12890.57 | -4564.13  (-35.41%) | 28332.97  (219.8%) | -10878.27  (-84.39%) |
| High SDI | Both | Caries of deciduous teeth | Prevalence | -5020206.2 | -463749.07  (9.24%) | -2925198.39  (58.27%) | -1631258.75  (32.49%) |
| High SDI | Both | Caries of deciduous teeth | Incidence | -6127839.92 | 346306.09  (-5.65%) | -7673465.61  (125.22%) | 1199319.6 (-19.57%) |
| High SDI | Both | Caries of deciduous teeth | YLDs (Years Lived with Disability) | -1896.19 | -178.18  (9.4%) | -1121.07  (59.12%) | -596.95  (31.48%) |
| High-middle SDI | Both | Caries of deciduous teeth | Prevalence | -15942315.78 | -792734.11  (4.97%) | -12442597.32  (78.05%) | -2706984.35  (16.98%) |
| High-middle SDI | Both | Caries of deciduous teeth | Incidence | -28065421.69 | 4445721.24  (-15.84%) | -29626388.26  (105.56%) | -2884754.68  (10.28%) |
| High-middle SDI | Both | Caries of deciduous teeth | YLDs (Years Lived with Disability) | -6052 | -302.8  (5%) | -4767.32  (78.77%) | -981.89  (16.22%) |
| Middle SDI | Both | Caries of deciduous teeth | Prevalence | -7933665.64 | -4114413.04  (51.86%) | -3000440.43  (37.82%) | -818812.17  (10.32%) |
| Middle SDI | Both | Caries of deciduous teeth | Incidence | -14869970.12 | 3554193.15  (-23.9%) | -6926885.93  (46.58%) | -11497277.34  (77.32%) |
| Middle SDI | Both | Caries of deciduous teeth | YLDs (Years Lived with Disability) | -2829.45 | -1571.68  (55.55%) | -1147.53  (40.56%) | -110.24  (3.9%) |
| Low-middle SDI | Both | Caries of deciduous teeth | Prevalence | 11105759.61 | -6167546.12  (-55.53%) | 28868839.77  (259.94%) | -11595534.04  (-104.41%) |
| Low-middle SDI | Both | Caries of deciduous teeth | Incidence | 20493259.84 | -4368435.45  (-21.32%) | 69671962.93  (339.98%) | -44810267.64  (-218.66%) |
| Low-middle SDI | Both | Caries of deciduous teeth | YLDs (Years Lived with Disability) | 4530.23 | -2350.29  (-51.88%) | 11005.92  (242.94%) | -4125.4  (-91.06%) |
| Low SDI | Both | Caries of deciduous teeth | Prevalence | 49860838.18 | -3012146.45  (-6.04%) | 61209685.23  (122.76%) | -8336700.6  (-16.72%) |
| Low SDI | Both | Caries of deciduous teeth | Incidence | 114964242.2 | -836435.79  (-0.73%) | 145291887.22  (126.38%) | -29491209.19  (-25.65%) |
| Low SDI | Both | Caries of deciduous teeth | YLDs (Years Lived with Disability) | 19151.22 | -1145.52  (-5.98%) | 23282.65  (121.57%) | -2985.91  (-15.59%) |
| Global | Both | Caries of permanent teeth | Prevalence | 292278155.2 | 22420954.03  (7.67%) | 300142518.33  (102.69%) | -30285317.17  (-10.36%) |
| Global | Both | Caries of permanent teeth | Incidence | 501604971.9 | 7758956.37  (1.55%) | 385185982.66  (76.79%) | 108660032.91  (21.66%) |
| Global | Both | Caries of permanent teeth | YLDs (Years Lived with Disability) | 290771.09 | 21490.48  (7.39%) | 298767.29  (102.75%) | -29486.67  (-10.14%) |
| High SDI | Both | Caries of permanent teeth | Prevalence | -5958837.68 | 1300531.08  (-21.83%) | 240382  (-4.03%) | -7499750.76  (125.86%) |
| High SDI | Both | Caries of permanent teeth | Incidence | -500134.75 | -496530.94  (99.28%) | 358260.95  (-71.63%) | -361864.77  (72.35%) |
| High SDI | Both | Caries of permanent teeth | YLDs (Years Lived with Disability) | -6062.48 | 1245.33  (-20.54%) | 239.13  (-3.94%) | -7546.94  (124.49%) |
| High-middle SDI | Both | Caries of permanent teeth | Prevalence | -16259712.81 | 1590166.89  (-9.78%) | -9679158.42  (59.53%) | -8170721.28  (50.25%) |
| High-middle SDI | Both | Caries of permanent teeth | Incidence | 12653098.55 | -3038471.72  (-24.01%) | -11455223.19  (-90.53%) | 27146793.47  (214.55%) |
| High-middle SDI | Both | Caries of permanent teeth | YLDs (Years Lived with Disability) | -16382.25 | 1356.07  (-8.28%) | -9655.12  (58.94%) | -8083.2  (49.34%) |
| Middle SDI | Both | Caries of permanent teeth | Prevalence | 56822552.31 | 9157742.77  (16.12%) | 55180713.76  (97.11%) | -7515904.22  (-13.23%) |
| Middle SDI | Both | Caries of permanent teeth | Incidence | 134669578 | 1866452.92  (1.39%) | 69910726.54  (51.91%) | 62892398.49  (46.7%) |
| Middle SDI | Both | Caries of permanent teeth | YLDs (Years Lived with Disability) | 56267.69 | 8644.63  (15.36%) | 55015.32  (97.77%) | -7392.26  (-13.14%) |
| Low-middle SDI | Both | Caries of permanent teeth | Prevalence | 131503715 | 17732506.37  (13.48%) | 130465574.06  (99.21%) | -16694365.44  (-12.69%) |
| Low-middle SDI | Both | Caries of permanent teeth | Incidence | 192284484.6 | 15621844.19  (8.12%) | 172980699.96  (89.96%) | 3681940.5  (1.91%) |
| Low-middle SDI | Both | Caries of permanent teeth | YLDs (Years Lived with Disability) | 131196.65 | 17332.38  (13.21%) | 129635.07  (98.81%) | -15770.8  (-12.02%) |
| Low SDI | Both | Caries of permanent teeth | Prevalence | 126125227.7 | 4922831.37  (3.9%) | 128017141.3  (101.5%) | -6814745  (-5.4%) |
| Low SDI | Both | Caries of permanent teeth | Incidence | 162320388.3 | 4621968.89  (2.85%) | 155130539.89  (95.57%) | 2567879.47  (1.58%) |
| Low SDI | Both | Caries of permanent teeth | YLDs (Years Lived with Disability) | 125706.77 | 4853.13  (3.86%) | 126993.48  (101.02%) | -6139.85  (-4.88%) |
| Global | Both | Periodontal diseases | Prevalence | 103640649.2 | 18549698.92  (17.9%) | 67168252.93  (64.81%) | 17922697.38  (17.29%) |
| Global | Both | Periodontal diseases | Incidence | 11948171.98 | 1360451.26  (11.39%) | 8954473.35  (74.94%) | 1633247.37  (13.67%) |
| Global | Both | Periodontal diseases | YLDs (Years Lived with Disability) | 685793.68 | 121965.45  (17.78%) | 443629.96  (64.69%) | 120198.27  (17.53%) |
| High SDI | Both | Periodontal diseases | Prevalence | 1461270.5 | 1429668.22  (97.84%) | 401347.44  (27.47%) | -369745.16  (-25.3%) |
| High SDI | Both | Periodontal diseases | Incidence | 225188.44 | 135759.25  (60.29%) | 55555.27  (24.67%) | 33873.92  (15.04%) |
| High SDI | Both | Periodontal diseases | YLDs (Years Lived with Disability) | 9403.35 | 9413  (100.1%) | 2652.32  (28.21%) | -2661.97  (-28.31%) |
| High-middle SDI | Both | Periodontal diseases | Prevalence | 5341770.6 | 4813680.44  (90.11%) | -774056.97  (-14.49%) | 1302147.14  (24.38%) |
| High-middle SDI | Both | Periodontal diseases | Incidence | 508594.01 | 537459.42  (105.68%) | -115391.09  (-22.69%) | 86525.68  (17.01%) |
| High-middle SDI | Both | Periodontal diseases | YLDs (Years Lived with Disability) | 35269.09 | 31748.86  (90.02%) | -5119.53  (-14.52%) | 8639.76  (24.5%) |
| Middle SDI | Both | Periodontal diseases | Prevalence | 30905373.1 | 11100417.1  (35.92%) | 13566854.15  (43.9%) | 6238101.86  (20.18%) |
| Middle SDI | Both | Periodontal diseases | Incidence | 3444585.88 | 978301.02  (28.4%) | 1886190.06  (54.76%) | 580094.8  (16.84%) |
| Middle SDI | Both | Periodontal diseases | YLDs (Years Lived with Disability) | 204492.32 | 73155.73  (35.77%) | 89705.8  (43.87%) | 41630.8  (20.36%) |
| Low-middle SDI | Both | Periodontal diseases | Prevalence | 47303146.7 | 5121950.78  (10.83%) | 40110522.72  (84.79%) | 2070673.2  (4.38%) |
| Low-middle SDI | Both | Periodontal diseases | Incidence | 5429110.84 | 294241.22  (5.42%) | 5008272.34  (92.25%) | 126597.28  (2.33%) |
| Low-middle SDI | Both | Periodontal diseases | YLDs (Years Lived with Disability) | 313242.73 | 33627.08  (10.74%) | 264664.72  (84.49%) | 14950.92  (4.77%) |
| Low SDI | Both | Periodontal diseases | Prevalence | 18606011.49 | -52320.61  (-0.28%) | 30972805.45  (166.47%) | -12314473.34  (-66.19%) |
| Low SDI | Both | Periodontal diseases | Incidence | 2338135.73 | -4712.06  (-0.2%) | 3805211.89  (162.75%) | -1462364.09  (-62.54%) |
| Low SDI | Both | Periodontal diseases | YLDs (Years Lived with Disability) | 123233.87 | -345.05  (-0.28%) | 204057.03  (165.59%) | -80478.1  (-65.31%) |
| Global | Both | Edentulism | Prevalence | 6754995.83 | 1017098.05  (15.06%) | 4884186.27  (72.3%) | 853711.52  (12.64%) |
| Global | Both | Edentulism | Incidence | 727598.12 | 67331.89  (9.25%) | 587958.28  (80.81%) | 72307.95  (9.94%) |
| Global | Both | Edentulism | YLDs (Years Lived with Disability) | 193847.63 | 29058.24  (14.99%) | 140277.9  (72.37%) | 24511.5  (12.64%) |
| High SDI | Both | Edentulism | Prevalence | 771644.99 | 116681.27  (15.12%) | 73149.85  (9.48%) | 581813.87  (75.4%) |
| High SDI | Both | Edentulism | Incidence | 98368.22 | 13579.95  (13.81%) | 11097.74  (11.28%) | 73690.53  (74.91%) |
| High SDI | Both | Edentulism | YLDs (Years Lived with Disability) | 22016.68 | 3338.11  (15.16%) | 2100.08  (9.54%) | 16578.49  (75.3%) |
| High-middle SDI | Both | Edentulism | Prevalence | 247393.23 | 321295.77  (129.87%) | 77761.75  (31.43%) | -151664.29  (-61.3%) |
| High-middle SDI | Both | Edentulism | Incidence | 24224.86 | 25795.83  (106.48%) | 9675.68  (39.94%) | -11246.65  (-46.43%) |
| High-middle SDI | Both | Edentulism | YLDs (Years Lived with Disability) | 7067.86 | 9216.18  (130.4%) | 2239.28  (31.68%) | -4387.59  (-62.08%) |
| Middle SDI | Both | Edentulism | Prevalence | 2215788.3 | 590557.83  (26.65%) | 1260599.73  (56.89%) | 364630.75  (16.46%) |
| Middle SDI | Both | Edentulism | Incidence | 235830.96 | 39188.43  (16.62%) | 152394.95  (64.62%) | 44247.58  (18.76%) |
| Middle SDI | Both | Edentulism | YLDs (Years Lived with Disability) | 63373.43 | 16886.99  (26.65%) | 36233.64  (57.17%) | 10252.79  (16.18%) |
| Low-middle SDI | Both | Edentulism | Prevalence | 1675157.71 | 162744.83  (9.72%) | 2068257.47  (123.47%) | -555844.59  (-33.18%) |
| Low-middle SDI | Both | Edentulism | Incidence | 180657.94 | 8174.58  (4.52%) | 231308.83  (128.04%) | -58825.48  (-32.56%) |
| Low-middle SDI | Both | Edentulism | YLDs (Years Lived with Disability) | 48270.46 | 4637.91  (9.61%) | 59343.73  (122.94%) | -15711.17  (-32.55%) |
| Low SDI | Both | Edentulism | Prevalence | 1843759.26 | -74.66  (0%) | 1983684.19  (107.59%) | -139850.28  (-7.59%) |
| Low SDI | Both | Edentulism | Incidence | 188355.55 | -117.86  (-0.06%) | 200715.35  (106.56%) | -12241.93  (-6.5%) |
| Low SDI | Both | Edentulism | YLDs (Years Lived with Disability) | 53083.19 | -2.83  (-0.01%) | 56756.36  (106.92%) | -3670.34  (-6.91%) |
| Global | Both | Other oral disorders | Prevalence | 19913395.67 | 2739644.42  (13.76%) | 17019264.23  (85.47%) | 154487.02  (0.78%) |
| Global | Both | Other oral disorders | YLDs (Years Lived with Disability) | 583941.37 | 78384.71  (13.42%) | 499272.07  (85.5%) | 6284.6  (1.08%) |
| High SDI | Both | Other oral disorders | Prevalence | 77626.21 | 195853.83  (252.3%) | -120543.32  (-155.29%) | 2315.7  (2.98%) |
| High SDI | Both | Other oral disorders | YLDs (Years Lived with Disability) | 1832.61 | 5565.34  (303.68%) | -3532.97  (-192.78%) | -199.76  (-10.9%) |
| High-middle SDI | Both | Other oral disorders | Prevalence | -411342.18 | 502018.02  (-122.04%) | -937392  (227.89%) | 24031.8  (-5.84%) |
| High-middle SDI | Both | Other oral disorders | YLDs (Years Lived with Disability) | -12200.27 | 14303.72  (-117.24%) | -27577.2  (226.04%) | 1073.22  (-8.8%) |
| Middle SDI | Both | Other oral disorders | Prevalence | 4087454.51 | 1324414.94  (32.4%) | 2690174.47  (65.82%) | 72865.1  (1.78%) |
| Middle SDI | Both | Other oral disorders | YLDs (Years Lived with Disability) | 119632.03 | 37865.08  (31.65%) | 79086.38  (66.11%) | 2680.57  (2.24%) |
| Low-middle SDI | Both | Other oral disorders | Prevalence | 8477959.58 | 1335794.91  (15.76%) | 7103735.69  (83.79%) | 38428.97  (0.45%) |
| Low-middle SDI | Both | Other oral disorders | YLDs (Years Lived with Disability) | 248958.87 | 38289.98  (15.38%) | 207886.18  (83.5%) | 2782.71  (1.12%) |
| Low SDI | Both | Other oral disorders | Prevalence | 7674292.91 | 369230.99  (4.81%) | 65.82 | 36686.38  (0.48%) |
| Low SDI | Both | Other oral disorders | YLDs (Years Lived with Disability) | 225500.85 | 10632.92  (4.72%) | 212291.32  (94.14%) | 2576.62  (1.14%) |

## Table H. Data of decomposition analysis for oral disorders among age subgroups across SDI quintiles between 1990 and 2021

| **Age Subgroups** | | | **Children** | | | | **Adolescents** | | | | **Young Adults** | | | |
| --- | --- | --- | --- | --- | --- | --- | --- | --- | --- | --- | --- | --- | --- | --- |
| **location** | **cause** | **measure** | **Overll difference** | **Aging** | **Population** | **Epidemiological change** | **Overll difference** | **Aging** | **Population** | **Epidemiological change** | **Overll difference** | **Aging** | **Population** | **Epidemiological change** |
| Global | Caries of deciduous teeth | Prevalence | 33991639.58 | 2105704.07(6.19%) | 53125084.6(156.29%) | -21239149.09(-62.48%) | -1957149.37 | 549491.47(-28.08%) | 6433630.22(-328.72%) | -8940271.05(456.8%) | - | - | - | - |
| Global | Caries of deciduous teeth | Incidence | 111356084.5 | 25174642.28(22.61%) | 114553798.64(102.87%) | -28372356.44(-25.48%) | -25047964.02 | 3153185.31(-12.59%) | 36961112.34(-147.56%) | -65162261.67(260.15%) | - | - | - | - |
| Global | Caries of deciduous teeth | YLDs | 13626.59 | 809.13(5.94%) | 20291.79(148.91%) | -7474.33(-54.85%) | -736.03 | 209.8(-28.5%) | 2456.33(-333.73%) | -3402.15(462.23%) | - | - | - | - |
| High SDI | Caries of deciduous teeth | Prevalence | -4263952.72 | 283765.11(-6.65%) | -3588182.82(84.15%) | -959535.01(22.5%) | -756253.48 | 106765.33(-14.12%) | -191032.13(25.26%) | -671986.69(88.86%) | - | - | - | - |
| High SDI | Caries of deciduous teeth | Incidence | -1083137.86 | 1561758.62(-144.19%) | -8432248.26(778.5%) | 5787351.78(-534.31%) | -5044702.05 | 583823.03(-11.57%) | -1044259.58(20.7%) | -4584265.5(90.87%) | - | - | - | - |
| High SDI | Caries of deciduous teeth | YLDs | -1608.74 | 108.42(-6.74%) | -1375.31(85.49%) | -341.85(21.25%) | -287.45 | 40.87(-14.22%) | -73.13(25.44%) | -255.19(88.78%) | - | - | - | - |
| High-middle SDI | Caries of deciduous teeth | Prevalence | -14387859.51 | 258981.22(-1.8%) | -12815227.22(89.07%) | -1831613.52(12.73%) | -1554456.27 | 390693.54(-25.13%) | -1066741.95(68.62%) | -878407.86(56.51%) | - | - | - | - |
| High-middle SDI | Caries of deciduous teeth | Incidence | -14397913.17 | 5900499.06(-40.98%) | -26903310.94(186.86%) | 6604898.71(-45.87%) | -13667508.52 | 2419625.44(-17.7%) | -6586219.85(48.19%) | -9500914.11(69.51%) | - | - | - | - |
| High-middle SDI | Caries of deciduous teeth | YLDs | -5460.11 | 100.19(-1.83%) | -4910.21(89.93%) | -650.09(11.91%) | -591.89 | 149.64(-25.28%) | -408.6(69.03%) | -332.93(56.25%) | - | - | - | - |
| Middle SDI | Caries of deciduous teeth | Prevalence | -7106326.1 | 581619.45(-8.18%) | -8242569.72(115.99%) | 554624.17(-7.8%) | -827339.54 | 409153.79(-49.45%) | 137439.8(-16.61%) | -1373933.13(166.07%) | - | - | - | - |
| Middle SDI | Caries of deciduous teeth | Incidence | -9410370.78 | 10740795.03(-114.14%) | -17205414.32(182.83%) | -2945751.49(31.3%) | -5459599.33 | 2315126.73(-42.4%) | 777656.75(-14.24%) | -8552382.81(156.65%) | - | - | - | - |
| Middle SDI | Caries of deciduous teeth | YLDs | -2514.63 | 224.64(-8.93%) | -3152.57(125.37%) | 413.29(-16.44%) | -314.82 | 156.38(-49.67%) | 52.53(-16.69%) | -523.73(166.36%) | - | - | - | - |
| Low-middle SDI | Caries of deciduous teeth | Prevalence | 11755100.39 | 540084.82(4.59%) | 19433169.34(165.32%) | -8218153.77(-69.91%) | -649340.78 | -410897.38(63.28%) | 3147245.2(-484.68%) | -3385688.6(521.4%) | - | - | - | - |
| Low-middle SDI | Caries of deciduous teeth | Incidence | 26991558.72 | 6871540.48(25.46%) | 43017129.14(159.37%) | -22897110.9(-84.83%) | -6498298.88 | -2328821(35.84%) | 17876330.24(-275.09%) | -22045808.12(339.26%) | - | - | - | - |
| Low-middle SDI | Caries of deciduous teeth | YLDs | 4770.44 | 207.79(4.36%) | 7409.26(155.32%) | -2846.61(-59.67%) | -240.21 | -156.53(65.16%) | 1198.81(-499.07%) | -1282.49(533.91%) | - | - | - | - |
| Low SDI | Caries of deciduous teeth | Prevalence | 48020636.88 | 532930.22(1.11%) | 53668236.33(111.76%) | -6180529.68(-12.87%) | 1840201.3 | -180841.63(-9.83%) | 4182581.44(227.29%) | -2161538.51(-117.46%) | - | - | - | - |
| Low SDI | Caries of deciduous teeth | Incidence | 109250948.4 | 4729094.02(4.33%) | 116546377.88(106.68%) | -12024523.47(-11.01%) | 5713293.81 | -1045798.23(-18.3%) | 24482549.55(428.52%) | -17723457.5(-310.21%) | - | - | - | - |
| Low SDI | Caries of deciduous teeth | YLDs | 18449.11 | 203.55(1.1%) | 20414.89(110.66%) | -2169.33(-11.76%) | 702.11 | -68.77(-9.8%) | 1590.46(226.53%) | -819.58(-116.73%) | - | - | - | - |
| Global | Caries of permanent teeth | Prevalence | -36114 | 2888663.47(-7998.74%) | 6190405.63(-17141.29%) | -9115183.11(25240.03%) | 50927961.6 | -581390.52(-1.14%) | 65643621.49(128.9%) | -14134269.38(-27.75%) | 241386307.6 | -3150793.66(-1.31%) | 251521883.6(104.2%) | -6984782.35(-2.89%) |
| Global | Caries of permanent teeth | Incidence | 6531967.89 | 5627646.28(86.16%) | 12056416.73(184.58%) | -11152095.12(-170.73%) | 135831636.8 | -918722.98(-0.68%) | 91023384.77(67.01%) | 45726975.04(33.66%) | 359241367.2 | -13706804.12(-3.82%) | 298671100.36(83.14%) | 74277070.98(20.68%) |
| Global | Caries of permanent teeth | YLDs | 37.81 | 2913.28(7705.58%) | 6243.11(16512.95%) | -9118.58(-24118.53%) | 51115.62 | -582.34(-1.14%) | 65755.28(128.64%) | -14057.31(-27.5%) | 239617.66 | -3546.74(-1.48%) | 249422.06(104.09%) | -6257.66(-2.61%) |
| High SDI | Caries of permanent teeth | Prevalence | -677879.14 | 155723.58(-22.97%) | -443990.62(65.5%) | -389612.1(57.48%) | -5096484.99 | -100502.3(1.97%) | -1678481.59(32.93%) | -3317501.11(65.09%) | -184473.56 | -235662.64(127.75%) | 3844662.14(-2084.13%) | -3793473.06(2056.38%) |
| High SDI | Caries of permanent teeth | Incidence | -1480639.86 | 358233.39(-24.19%) | -1021463.58(68.99%) | -817409.67(55.21%) | -3294842 | -194747.17(5.91%) | -2928023.67(88.87%) | -172071.16(5.22%) | 4275347.11 | -1624273.46(-37.99%) | 5271842.34(123.31%) | 627778.23(14.68%) |
| High SDI | Caries of permanent teeth | YLDs | -685.95 | 157.27(-22.93%) | -448.41(65.37%) | -394.81(57.56%) | -5144.92 | -99.9(1.94%) | -1681.99(32.69%) | -3363.03(65.37%) | -231.62 | -254.56(109.9%) | 3812.9(-1646.2%) | -3789.96(1636.29%) |
| High-middle SDI | Caries of permanent teeth | Prevalence | -3696454.23 | 766704.6(-20.74%) | -1619789.49(43.82%) | -2843369.34(76.92%) | -13962451.34 | -252916.4(1.81%) | -9706124.23(69.52%) | -4003410.7(28.67%) | 1399192.77 | -1512472.68(-108.1%) | 4264293.74(304.77%) | -1352628.29(-96.67%) |
| High-middle SDI | Caries of permanent teeth | Incidence | -4901956.08 | 1463142.82(-29.85%) | -3100431.26(63.25%) | -3264667.64(66.6%) | 59399.23 | -441054.58(-742.53%) | -12592014.72(-21198.95%) | 13092468.53(22041.48%) | 17495655.4 | -4505344.54(-25.75%) | 4675441.5(26.72%) | 17325558.44(99.03%) |
| High-middle SDI | Caries of permanent teeth | YLDs | -3732.69 | 775.39(-20.77%) | -1638.17(43.89%) | -2869.92(76.89%) | -14057.15 | -253.04(1.8%) | -9751.14(69.37%) | -4052.97(28.83%) | 1407.6 | -1642.06(-116.66%) | 4239.12(301.16%) | -1189.46(-84.5%) |
| Middle SDI | Caries of permanent teeth | Prevalence | -3514640.42 | 1283161.02(-36.51%) | -951882.81(27.08%) | -3845918.63(109.43%) | -3450367.87 | -389441.4(11.29%) | 1360500.05(-39.43%) | -4421426.52(128.14%) | 63787560.6 | -2534573.92(-3.97%) | 65582111.62(102.81%) | 740022.9(1.16%) |
| Middle SDI | Caries of permanent teeth | Incidence | -3533478.35 | 2459197.29(-69.6%) | -1826056.98(51.68%) | -4166618.66(117.92%) | 26676286.02 | -617452.34(-2.31%) | 1836675.6(6.89%) | 25457062.76(95.43%) | 111526770.3 | -7683656.03(-6.89%) | 77527585.63(69.51%) | 41682840.69(37.37%) |
| Middle SDI | Caries of permanent teeth | YLDs | -3531.22 | 1296.16(-36.71%) | -961.54(27.23%) | -3865.85(109.48%) | -3462.28 | -391.57(11.31%) | 1365.11(-39.43%) | -4435.81(128.12%) | 63261.18 | -2778.77(-4.39%) | 65141.2(102.97%) | 898.75(1.42%) |
| Low-middle SDI | Caries of permanent teeth | Prevalence | 1528412.36 | 706563.13(46.23%) | 2101826.35(137.52%) | -1279977.13(-83.75%) | 29999331.66 | 570547.09(1.9%) | 34529302.97(115.1%) | -5100518.4(-17%) | 99975970.97 | -81387.69(-0.08%) | 110429123.02(110.46%) | -10371764.36(-10.37%) |
| Low-middle SDI | Caries of permanent teeth | Incidence | 3944835.06 | 1391779.25(35.28%) | 4139037.19(104.92%) | -1585981.38(-40.2%) | 50637035.97 | 865736.16(1.71%) | 49323454.31(97.41%) | 447845.5(0.88%) | 137702613.6 | -2096782.64(-1.52%) | 134887574.13(97.96%) | 4911822.12(3.57%) |
| Low-middle SDI | Caries of permanent teeth | YLDs | 1596.67 | 711.65(44.57%) | 2116.9(132.58%) | -1231.88(-77.15%) | 30226.01 | 571.49(1.89%) | 34523.94(114.22%) | -4869.41(-16.11%) | 99373.97 | -162.24(-0.16%) | 109258.61(109.95%) | -9722.39(-9.78%) |
| Low SDI | Caries of permanent teeth | Prevalence | 6331000 | 555054.63(8.77%) | 6225862.39(98.34%) | -449917.02(-7.11%) | 43470823.17 | 265606.94(0.61%) | 45667379.62(105.05%) | -2462163.39(-5.66%) | 76323404.51 | -1731.66(0%) | 80249787.23(105.14%) | -3924651.05(-5.14%) |
| Low SDI | Caries of permanent teeth | Incidence | 12505860.23 | 1032193.07(8.25%) | 11555215.76(92.4%) | -81548.61(-0.65%) | 61732448.56 | 368228.18(0.6%) | 59868601.88(96.98%) | 1495618.49(2.42%) | 88082079.47 | -2680.46(0%) | 86917732.26(98.68%) | 1167027.67(1.32%) |
| Low SDI | Caries of permanent teeth | YLDs | 6397.55 | 557.21(8.71%) | 6248.74(97.67%) | -408.4(-6.38%) | 43586.92 | 264.41(0.61%) | 45571.93(104.55%) | -2249.43(-5.16%) | 75722.3 | -0.16(0%) | 79224.04(104.62%) | -3501.59(-4.62%) |
| Global | Periodontal diseases | Prevalence | - | - | - | - | 2288083.35 | -126159.42(-5.51%) | 1397497.43(61.08%) | 1016745.33(44.44%) | 101352565.9 | 11907759.47(11.75%) | 72516081.74(71.55%) | 16928724.68(16.7%) |
| Global | Periodontal diseases | Incidence | - | - | - | - | 699091.11 | -42781.61(-6.12%) | 474184.38(67.83%) | 267688.34(38.29%) | 11249080.87 | 699318.93(6.22%) | 9182381.43(81.63%) | 1367380.51(12.16%) |
| Global | Periodontal diseases | YLDs | - | - | - | - | 15125.9 | -842.11(-5.57%) | 9328.84(61.67%) | 6639.17(43.89%) | 670667.78 | 78172.31(11.66%) | 478782.74(71.39%) | 113712.73(16.96%) |
| High SDI | Periodontal diseases | Prevalence | - | - | - | - | -95414.67 | -11470.96(12.02%) | -21247.67(22.27%) | -62696.04(65.71%) | 1556685.16 | 975486.63(62.66%) | 888515.9(57.08%) | -307317.37(-19.74%) |
| High SDI | Periodontal diseases | Incidence | - | - | - | - | -35722.7 | -3795.05(10.62%) | -7029.1(19.68%) | -24898.55(69.7%) | 260911.14 | 82146.34(31.48%) | 119988.7(45.99%) | 58776.1(22.53%) |
| High SDI | Periodontal diseases | YLDs | - | - | - | - | -656.87 | -76.68(11.67%) | -142.03(21.62%) | -438.15(66.7%) | 10060.22 | 6415.16(63.77%) | 5870.61(58.35%) | -2225.55(-22.12%) |
| High-middle SDI | Periodontal diseases | Prevalence | - | - | - | - | -33481.67 | -34701.58(103.64%) | -95118.22(284.09%) | 96338.13(-287.73%) | 5375252.28 | 3267818.38(60.79%) | 901272.89(16.77%) | 1206161.01(22.44%) |
| High-middle SDI | Periodontal diseases | Incidence | - | - | - | - | -13563.29 | -11563.82(85.26%) | -31691.24(233.65%) | 29691.77(-218.91%) | 522157.29 | 333152.21(63.8%) | 131726.68(25.23%) | 57278.41(10.97%) |
| High-middle SDI | Periodontal diseases | YLDs | - | - | - | - | -237.01 | -232.02(97.9%) | -635.95(268.32%) | 630.96(-266.22%) | 35506.09 | 21535(60.65%) | 5959.88(16.79%) | 8011.21(22.56%) |
| Middle SDI | Periodontal diseases | Prevalence | - | - | - | - | 216893.34 | -60178.96(-27.75%) | 19851.57(9.15%) | 257220.72(118.59%) | 30688479.76 | 7197964.86(23.45%) | 17499191.26(57.02%) | 5991323.64(19.52%) |
| Middle SDI | Periodontal diseases | Incidence | - | - | - | - | 71843.88 | -21063.3(-29.32%) | 6948.51(9.67%) | 85958.66(119.65%) | 3372742 | 528515.63(15.67%) | 2348355.64(69.63%) | 495870.73(14.7%) |
| Middle SDI | Periodontal diseases | YLDs | - | - | - | - | 1416.41 | -402.14(-28.39%) | 132.66(9.37%) | 1685.9(119.03%) | 203075.91 | 47383.31(23.33%) | 115678.01(56.96%) | 40014.59(19.7%) |
| Low-middle SDI | Periodontal diseases | Prevalence | - | - | - | - | 1252007.65 | 153180.05(12.23%) | 1045894.35(83.54%) | 52933.26(4.23%) | 46051139.05 | 2535610.78(5.51%) | 41491398.09(90.1%) | 2024130.18(4.4%) |
| Low-middle SDI | Periodontal diseases | Incidence | - | - | - | - | 401839.6 | 53016.14(13.19%) | 362312.68(90.16%) | -13489.22(-3.36%) | 5027271.24 | 78666.58(1.56%) | 4807719.34(95.63%) | 140885.32(2.8%) |
| Low-middle SDI | Periodontal diseases | YLDs | - | - | - | - | 8293.26 | 1022.2(12.33%) | 6980.07(84.17%) | 290.99(3.51%) | 304949.47 | 16595.41(5.44%) | 273646.3(89.73%) | 14707.76(4.82%) |
| Low SDI | Periodontal diseases | Prevalence | - | - | - | - | 948373.58 | 84523.06(8.91%) | 1642734.82(173.22%) | -778884.3(-82.13%) | 17657637.91 | -4170.37(-0.02%) | 29190780.84(165.32%) | -11528972.56(-65.29%) |
| Low SDI | Periodontal diseases | Incidence | - | - | - | - | 274863.74 | 27566.69(10.03%) | 537616.42(195.59%) | -290319.37(-105.62%) | 2063271.99 | -1255.22(-0.06%) | 3234832.2(156.78%) | -1170304.99(-56.72%) |
| Low SDI | Periodontal diseases | YLDs | - | - | - | - | 6312.22 | 563.57(8.93%) | 10953.86(173.53%) | -5205.21(-82.46%) | 116921.65 | -28.71(-0.02%) | 192179.46(164.37%) | -75229.1(-64.34%) |

## Table I. The age-standardized prevalence rates per 100,000 populations of untreated caries of deciduous teeth for 170 countries and territories in 1990-2021 and the AAPCs in the period 1990-2021

| **Location Name** | **AAPC (95%CI)** | 1990 | 1991 | 1992 | 1993 | 1994 | 1995 | 1996 | 1997 | 1998 | 1999 |
| --- | --- | --- | --- | --- | --- | --- | --- | --- | --- | --- | --- |
| Australia | 2.8  (2.19 - 3.4) | 13994.88 (12361.64-15928.05) | 14180.03 (12523.16-16033.88) | 14358.63 (12701.03-16258.87) | 14518.73 (12870.85-16453.54) | 14648.9 (12985.47-16637.56) | 14742.38 (13049.5-16734.83) | 14784.03 (13134.96-16715.89) | 14776.52 (13182.29-16648.49) | 14743.85 (13172.27-16580.41) | 14715.43 (13139.9-16550.97) |
| Madagascar | -2.19  (-2.39 - -1.99) | 44361.71 (36485.84-52113.82) | 44488.17 (36626.99-52264.16) | 44571.47 (36685.73-52410.06) | 44613.61 (36627.22-52646.52) | 44622.46 (36580.07-52634.62) | 44605.68 (36613.06-52570.48) | 43425.86 (36413.03-50440.05) | 40643.14 (34197.03-46932.94) | 37294.87 (31364.87-43413.36) | 34412.94 (28713.49-40711.29) |
| United States of America | 1.84  (1.34 - 2.35) | 17813.4 (11043.41-25208.81) | 17644.76 (10962.91-24934.9) | 17516.53 (10892.7-24709.41) | 17391.63 (10809.42-24542.07) | 17255.23 (10702.1-24396.37) | 17111.39 (10587.12-24239.59) | 16836.42 (10371.55-23866.51) | 16375.74 (10019-23286.7) | 15855.03 (9569.78-22636.02) | 15396.89 (9181.31-22074.89) |
| Netherlands | 1.54  (1.1 - 1.98) | 16582.77 (9672.12-25949.44) | 18380.22 (11331.89-27361.02) | 20022.21 (12871.96-28837.71) | 21415.72 (14111.25-30126.28) | 22425.73 (14957.03-31155.17) | 22914.21 (15490.98-31855.76) | 23014.68 (16045.1-31240.4) | 22987.94 (16493.23-30522.86) | 22871.99 (16663.7-30035.91) | 22736.29 (16749.29-29707.3) |
| Greece | -1.4  (-1.7 - -1.1) | 24575.56 (19619.6-29685) | 24606.49 (19567.4-30091.35) | 24664.54 (19533.92-30461.93) | 24740.89 (19502.41-30843.88) | 24798.06 (19331.85-31049.53) | 24792.01 (19031.37-31325.93) | 24825.6 (19207.3-31162.94) | 24942.26 (19647.2-30812.5) | 25039.88 (20154.68-30590) | 25002.67 (20437.06-30352.71) |
| Canada | 1.35  (1.11 - 1.6) | 16790.86 (10635.63-24361.11) | 16753.06 (10628.34-24273.35) | 16793.17 (10670.96-24302.52) | 16869.67 (10736.21-24461.48) | 16926.49 (10792.17-24570.22) | 16949.09 (10794.12-24648.4) | 16957.83 (10824.59-24583.09) | 16963.32 (10775.1-24438.87) | 16952.76 (10727.11-24386.52) | 16916.27 (10681.7-24370) |
| Norway | 1.3  (1.03 - 1.57) | 16509.72 (10115.55-23793.63) | 16360.88 (9989-23621.89) | 16251.84 (9888.74-23447.1) | 16180.32 (9818.68-23325.87) | 16128.41 (9771.19-23221.14) | 16105.85 (9731.03-23152.86) | 16133.16 (9740.62-23225.38) | 16218.89 (9776.28-23374.01) | 16334.27 (9830.6-23513.2) | 16437.68 (9874.72-23606.1) |
| Finland | 1.23  (1.14 - 1.31) | 13782.92 (10097.64-18589.88) | 14180.85 (10347.3-19059.93) | 14635.94 (10596.44-19555.55) | 15097.48 (10782.48-20308.06) | 15544.49 (10985.61-21341.19) | 15938.2 (11034.63-22518.87) | 16597.92 (11525.18-23251.72) | 17638.36 (12047.65-24592.88) | 18757.25 (12541.83-26085.41) | 19635.17 (12813.99-27511.58) |
| New Zealand | -1.21  (-1.28 - -1.14) | 17848.06 (11160.04-25621.82) | 17893.44 (11202.09-25617.83) | 17961.96 (11174.64-25714.26) | 18028.46 (11250.59-25806.1) | 18075.49 (11283.99-25836.84) | 18087.47 (11201.3-25766.59) | 18034.36 (11210.99-25680.87) | 17922.61 (11206.53-25577.73) | 17765.44 (11129.2-25272.28) | 17561.06 (10964.36-24902.14) |
| Ireland | 1.08  (0.51 - 1.66) | 21185.94 (14056.51-29979.39) | 21128.77 (13958.17-29882.19) | 21102.59 (13953.28-29981.97) | 21121.76 (13914.35-30061.85) | 21121.25 (13898.58-30111.83) | 21042.8 (13759.29-30112.7) | 20852.16 (13670.76-29850.78) | 20604.1 (13464.6-29404.84) | 20394.06 (13281.93-29189.24) | 20275.17 (13200.11-28995.24) |
| Bhutan | -1.04  (-1.13 - -0.94) | 29421.92 (20400.5-38953.01) | 29484.91 (20505.68-38936.98) | 29599.13 (20606.99-38958.69) | 29759.17 (20759.22-39235.01) | 29789.51 (20793.87-39376.8) | 29697.87 (20628.48-39402.8) | 29277.3 (20911.88-38133.36) | 28426.05 (20889.59-36394.51) | 27435.57 (20612.19-34629.36) | 26569.89 (20113.96-33402.08) |
| Switzerland | -1.03  (-1.11 - -0.95) | 14793.33 (9195.98-22113.69) | 15224.64 (9725.63-22151.27) | 15552.53 (10154.29-22262.23) | 15795.16 (10446.72-22165.95) | 15945.44 (10641.24-22219.02) | 15985.71 (10741.87-22236.32) | 15534.93 (10543.08-21355.37) | 14486.24 (9881.22-19861.71) | 13244.37 (9057.96-18260.9) | 12183.69 (8388.07-17056.41) |
| Russian Federation | 1.01  (0.74 - 1.27) | 31647.76 (22623.57-41229.19) | 31917.85 (22865.52-41529.68) | 32129.52 (23064.93-41750.41) | 32203.02 (23136.94-41806.42) | 32077.77 (23040.51-41624.93) | 31834.39 (22841.83-41306.14) | 31641.44 (22686.28-41121.52) | 31535.27 (22585.45-41001.15) | 31458.71 (22513.48-40910.03) | 31362.57 (22442.89-40797.45) |
| Kyrgyzstan | 0.97  (0.7 - 1.25) | 31384.43 (22490.8-40790.29) | 31417.08 (22600.83-40934.06) | 31452.46 (22663.68-40979.27) | 31493.48 (22704.87-41064.01) | 31542.64 (22752.25-41091.18) | 31598.74 (22838.4-41264.51) | 31674.95 (22864.34-41205.27) | 31791.77 (22852.42-41198.18) | 31887.18 (22827.78-41067.69) | 31919.97 (22764.15-40959.17) |
| Ukraine | 0.97  (0.68 - 1.26) | 32270.7 (23118.94-41419.04) | 32353.99 (23237.24-41720.16) | 32401.6 (23296.31-41956.01) | 32451.72 (23347.88-42266.19) | 32496.85 (23394.64-42153.13) | 32503.45 (23431.43-42113.43) | 32472.97 (23470.77-42056.49) | 32444.8 (23501.96-41999.39) | 32433.54 (23488.23-41948.17) | 32408.11 (23470.11-42078.97) |
| Brunei Darussalam | 0.96  (0.82 - 1.1) | 26792.24 (18422.95-35606.16) | 26757.8 (18419.91-35525.05) | 26719.43 (18476.75-35351.03) | 26675.88 (18514.53-35176.72) | 26647.65 (18456.8-35227.9) | 26638.22 (18485.91-35314.53) | 26654.95 (18484.86-35293.76) | 26691.99 (18623.96-35379.19) | 26751.98 (18743.86-35461.84) | 26820.35 (18684.71-35665.93) |
| Latvia | 0.96  (0.59 - 1.33) | 28392.46 (21175.15-36443.16) | 28676.37 (21485.53-36940.49) | 28941.49 (21717.94-37442.23) | 29205 (22007.37-37767.84) | 29427.82 (22269.86-38028.8) | 29559.99 (22367.48-38412.61) | 29536.76 (22311.32-38110.01) | 29376.45 (22136.67-37770.94) | 29090.76 (21841.43-37200.27) | 28756.32 (21558.31-36625.61) |
| Tajikistan | 0.96  (0.74 - 1.17) | 31134.18 (22555.59-40271.53) | 31260.2 (22613.84-40447.25) | 31375.16 (22675.65-40766.39) | 31481 (22864.32-40927.07) | 31568.76 (22818.95-41094.13) | 31598.13 (22774.37-41268.13) | 31595.79 (22820.15-41147.69) | 31623.08 (22864.89-41110.83) | 31641.85 (22941.88-41121.32) | 31632.01 (22991.27-41117.17) |
| Germany | -0.95  (-1.5 - -0.39) | 23763.28 (18607.11-29487.1) | 23222.22 (18202.62-28870.74) | 22744.35 (17837.41-28155.56) | 22297.45 (17464.65-27647.06) | 21956.07 (17319.21-27215.31) | 21746.47 (17193.66-26858.45) | 21652.91 (17284.68-26426.29) | 21610.92 (17397.72-26257.68) | 21635.82 (17573.07-26290.1) | 21698.44 (17747.04-26340.84) |
| Equatorial Guinea | -0.93  (-1.15 - -0.7) | 25835.13 (17259.86-34861.74) | 25913.88 (17561.88-34820.28) | 25975.05 (17673.94-34971.65) | 26012.11 (17730.03-34988.71) | 26023.88 (17724.13-34982.18) | 26018.75 (17701.89-35133.73) | 25984.56 (17672.86-34855.61) | 25903.53 (17577.4-34598.18) | 25777.6 (17451.72-34395.6) | 25614.45 (17296-34245.41) |
| Andorra | 0.92  (0.75 - 1.09) | 19753.74 (12116.03-28039.65) | 19832.44 (12243.75-28070.32) | 19842.64 (12329.64-28088.52) | 19863.18 (12409.98-28098.7) | 19954.32 (12455.82-28206.67) | 20024.72 (12472.2-28317.7) | 20008.9 (12503.55-28288.55) | 19900.03 (12481.38-27995.33) | 19773.88 (12443.14-27620.25) | 19769.29 (12455.48-27680.28) |
| Honduras | 0.92  (0.85 - 0.99) | 25848.46 (17372.46-34911.77) | 25852.56 (17431.81-34957.09) | 25856.84 (17426.26-35220.38) | 25862.8 (17417.89-35372.73) | 25870.04 (17364.82-35400.61) | 25877.96 (17268.72-35332.12) | 25883.65 (17354.29-35361.78) | 25888.55 (17411.01-35335.4) | 25900.67 (17454.19-35379.05) | 25909.31 (17428.3-35231.94) |
| Mongolia | 0.91  (0.66 - 1.17) | 31199.7 (22575.6-40645.9) | 31343.83 (22661.82-40803.74) | 31506.45 (22707.23-40748.5) | 31660.22 (22820.52-41027.82) | 31757.12 (22891.83-41198.48) | 31739 (22894.96-41256.01) | 31647.67 (22839.42-41230.1) | 31568.07 (22777.99-41190.13) | 31513.02 (22727.45-41022.25) | 31446.76 (22635.37-40819.98) |
| Chile | 0.9  (0.74 - 1.06) | 28359.87 (21219.16-36178.17) | 28448.37 (21348.58-36303.56) | 28572.46 (21504.34-36622.68) | 28641.29 (21562.98-36765.41) | 28642.54 (21506.16-36713.5) | 28606.78 (21452.1-36769.06) | 28549.17 (21404.24-36644.82) | 28473.91 (21392.64-36695.43) | 28401.08 (21335.38-36480.88) | 28351.05 (21263.12-36481.26) |
| Austria | 0.89  (0.79 - 1) | 19849.47 (12353.3-28286.52) | 19814.31 (12379.24-28231.44) | 19798.8 (12382.41-28194.19) | 19823.23 (12444.18-28237.17) | 19886.22 (12402.58-28303.29) | 19956.43 (12401.05-28322.1) | 19989.44 (12514.8-28367.47) | 19988.67 (12577.6-28407.34) | 19986.41 (12528.86-28364.28) | 19984.2 (12505.08-28331.94) |
| Belarus | 0.89  (0.65 - 1.14) | 32400.1 (22726.77-42523.77) | 32547.76 (23042.38-42528.15) | 32636.59 (23230.82-42530.57) | 32705.95 (23421.39-42407.17) | 32773.25 (23419.8-42427.7) | 32827.43 (23408.16-42534.03) | 32837.36 (23518.16-42591.15) | 32761.87 (23502.53-42616) | 32567.89 (23190.85-42570.31) | 32320.07 (22859.5-42527.54) |
| Kazakhstan | 0.88  (0.49 - 1.26) | 31087.07 (22261.98-40277.09) | 31183.87 (22319.19-40320.91) | 31238.44 (22378.9-40312.28) | 31328.04 (22425-40373.52) | 31447.34 (22424.25-40497.82) | 31560.46 (22516.29-40848.68) | 31689.03 (22638.54-40984.89) | 31791.94 (22772.18-41096.81) | 31799.54 (22843.27-41288.23) | 31713.53 (22822.47-41297.82) |
| Slovakia | 0.88  (0.59 - 1.18) | 31260.55 (22850.92-40303.81) | 31270.08 (22878.78-40305.66) | 31349.37 (22825.84-40329.87) | 31490.02 (22803.1-40504.32) | 31683.83 (22920.77-40847.01) | 31851.69 (23018.49-41131.04) | 31861.48 (22973.01-41099.77) | 31721.96 (22816.09-40845.42) | 31557.79 (22610.56-40701.2) | 31466.42 (22537.85-40793.13) |
| Rwanda | -0.84  (-1 - -0.68) | 27047.44 (18308.43-36677.08) | 27127.16 (18471.88-36620.26) | 27152.85 (18478.51-36501.97) | 27124.42 (18436.32-36340.04) | 27265.97 (18438.25-36438.15) | 27496.06 (18442.8-36793.48) | 26941.03 (18981.57-35288.24) | 25749.78 (18809.74-33354.93) | 24806.45 (18317.8-31791.84) | 24298.69 (18100.35-30717.06) |
| Iceland | 0.83  (0.61 - 1.05) | 19844.68 (12260.65-28456.88) | 19889.47 (12311.29-28427.26) | 19953.45 (12436.19-28478.59) | 20026.58 (12561.83-28462.82) | 20018.38 (12567.3-28373.12) | 20041.91 (12535.74-28323.42) | 20046.56 (12543.93-28381.01) | 20043.81 (12524.25-28395.52) | 20006.93 (12484.55-28299.32) | 19971.99 (12472.45-28293.16) |
| Spain | 0.82  (0.52 - 1.13) | 19339.1 (13879.89-26113.06) | 19101.97 (13709.52-25729.58) | 18901.49 (13669.42-25451.44) | 18753.83 (13687.33-25409.35) | 18671.98 (13536.44-25513.42) | 18660.25 (13430.15-25582.69) | 18667.2 (13393.16-25626.56) | 18662.81 (13481.63-25710.28) | 18657.81 (13416.63-25856.87) | 18669.18 (13301.86-25888.12) |
| Eritrea | -0.81  (-0.89 - -0.74) | 26808.73 (18125.02-36212.77) | 26925.7 (18250.5-36147.82) | 27070.98 (18413.26-36388.33) | 27052.25 (18479.63-36431.45) | 26922.82 (18431.52-36046.14) | 26885.06 (18380.29-35978.99) | 26949.62 (18440.23-36223.2) | 27054.98 (18440.4-36561.38) | 27115.41 (18377.01-36616.1) | 27104.97 (18324.19-36492.44) |
| Djibouti | -0.8  (-0.88 - -0.72) | 26409.42 (17823.7-35858.03) | 26380.25 (17866.23-35500.53) | 26547.62 (18017.08-35398.12) | 26819.62 (18264.91-35614.86) | 26862.29 (18267.9-35683.02) | 26723.84 (18117.52-35689.69) | 26627.06 (18094.39-35476.05) | 26581.13 (18096.91-35471.2) | 26561.29 (18108.3-35470.36) | 26537.94 (18127.79-35478.25) |
| Democratic Republic of the Congo | -0.76  (-0.84 - -0.67) | 25813.34 (17501.81-34607.95) | 25929.5 (17631.99-34808.9) | 26035.09 (17754.84-35049.46) | 26123.28 (17857.37-35216.18) | 26190.35 (17942.94-35181.63) | 26227.77 (17808.79-35096.45) | 26245.27 (17912.07-35166.58) | 26263.18 (17945.03-35080.67) | 26278.38 (17887.31-35009.57) | 26283.21 (17888.15-35113.44) |
| Poland | 0.76  (0.33 - 1.19) | 31654.23 (23294.25-40570.33) | 31906.52 (23690.1-40745.79) | 32195.29 (24144.2-40961.2) | 32490.7 (24497.91-41224.35) | 32738.32 (24754.81-41500.55) | 32891.32 (24955.1-41740.77) | 32910.16 (24965.73-41714.72) | 32838.7 (24893.73-41604.73) | 32745.24 (24793.94-41515.8) | 32652.94 (24663.12-41416.57) |
| Burundi | -0.75  (-0.93 - -0.57) | 26512.47 (18051.63-35350.25) | 26685.54 (18085.52-35600.51) | 26835.11 (18091.1-35690.25) | 27002.3 (18184.66-35894.03) | 27120.98 (18283.49-36189.71) | 27045.15 (18174.93-36255.57) | 27011.61 (18205.36-36033.16) | 27132.9 (18326.29-36102.8) | 27170.36 (18489.92-36253.86) | 27090.96 (18513.75-36425.72) |
| Mozambique | -0.67  (-0.7 - -0.64) | 27147.66 (18597.46-36521.24) | 27130.54 (18569.43-36571.04) | 27136.99 (18529.03-36738.84) | 27156.59 (18510.44-36792.28) | 27127.76 (18449.29-36838.06) | 27090.29 (18377.03-36867.25) | 27142.61 (18404.53-36813.81) | 27233.98 (18444.03-36781.81) | 27274.13 (18476.69-36735.97) | 27265.11 (18399.2-36776.26) |
| Lesotho | -0.65  (-0.76 - -0.53) | 24309.26 (15887.43-33443) | 24284.9 (15956.1-33461.92) | 24265.95 (15996.63-33356.58) | 24251.38 (15986.97-33524.64) | 24240.42 (16037.91-33573.65) | 24233.48 (16019.64-33704.7) | 24225.25 (16022.95-33547.19) | 24214.7 (16063.08-33542.21) | 24209.89 (16076.12-33630.67) | 24215.19 (16078.62-33627.21) |
| Pakistan | -0.56  (-0.76 - -0.36) | 31259.46 (22601.11-40312.97) | 31237.25 (22618.37-40389.52) | 31221.68 (22600.15-40403.41) | 31213.42 (22584.19-40393.35) | 31209.08 (22581.49-40411.95) | 31206.64 (22562.46-40365.01) | 30938.99 (22556.49-39893.08) | 30289.35 (22059.98-39147.29) | 29481.58 (21459.95-38055.73) | 28741.89 (20854.79-37058.93) |
| Trinidad and Tobago | -0.5  (-0.59 - -0.42) | 26112.52 (17869.03-35173.35) | 26147.19 (17875.04-35229.91) | 26147.2 (17841.99-35202.17) | 26133.36 (17779.08-35170.53) | 26116.12 (17725.44-35098.43) | 26090.74 (17619.79-35107.04) | 26045.75 (17623.06-34861.51) | 25972.77 (17560.49-34715.8) | 25878.83 (17486.75-34660.65) | 25775.29 (17393.71-34567.71) |
| Ecuador | -0.49  (-0.56 - -0.41) | 32957.25 (24214.39-41916.48) | 32850.67 (24153.91-41623.71) | 32849.8 (24126.21-41538.24) | 32933.49 (24120.36-41468.23) | 33058.05 (24215.35-41556.03) | 33183.22 (24276.67-41744.84) | 33271.08 (24250.26-41925.68) | 33297.12 (24206.57-41995.53) | 33268.33 (24082.43-41973.16) | 33229.46 (24046.63-42080.34) |
| Panama | -0.48  (-0.52 - -0.44) | 25728.47 (16815.7-34850.38) | 25709.5 (16922.74-34623.98) | 25699 (17132.98-34619.96) | 25680.69 (17287.28-34643.62) | 25632.27 (17297.31-34773.72) | 25549.41 (17246.7-34744.28) | 25445.53 (17148.32-34607.65) | 25365.98 (17096.08-34409.78) | 25357.37 (17074.51-34519.76) | 25399.04 (16997.19-34624.44) |
| Kuwait | 0.47  (0.3 - 0.64) | 22572.73 (16048.05-29907.14) | 23187.97 (16826.48-30414.17) | 23444.43 (17160.5-30640.54) | 23341.07 (17353.69-30373.33) | 23157.19 (17298.68-30298.95) | 23119.43 (17198.62-30441.37) | 23239.44 (17307.01-30514.48) | 23394.52 (17399.41-30605.13) | 23482.66 (17424.01-30575.14) | 23547.45 (17400.62-30640.2) |
| Nicaragua | -0.43  (-0.51 - -0.36) | 25840.27 (17133.66-35081.88) | 25861.62 (17122.86-34904.02) | 25896.4 (17101.93-34769.3) | 25941.01 (17114.68-34777.16) | 25981.98 (17115.87-34844.87) | 26006.54 (17119.09-35016.48) | 26016.56 (17140.26-34954.25) | 26023.82 (17194.42-34959.41) | 26038.65 (17227.13-34879.12) | 26048.26 (17267.52-35065.35) |
| Oman | -0.42  (-0.45 - -0.4) | 31190.97 (22140.04-39724.45) | 31211.07 (22169.56-39718.81) | 31236.49 (22229.76-39789.71) | 31275.09 (22255.22-39887.44) | 31325.28 (22181.07-39985.26) | 31385.43 (22132.75-40149.71) | 31452.78 (22245.72-40217.21) | 31510.12 (22319.96-40360.16) | 31538.07 (22377.42-40413.57) | 31513.74 (22390.27-40398.12) |
| Argentina | -0.41  (-0.62 - -0.19) | 29166.59 (21090.64-36743.41) | 27960.67 (19930.9-36001.76) | 27024.47 (18714.51-35543) | 26374.89 (17802.11-35090.44) | 25979.92 (17287.4-35085.24) | 25824.98 (16935.7-35466.19) | 26069.54 (17576.83-35455.23) | 26708.95 (18308.72-35630.25) | 27507.59 (19188.46-35886.88) | 28210.38 (19785.77-36308.75) |
| Venezuela (Bolivarian Republic of) | -0.41  (-0.5 - -0.33) | 25141.48 (16574.2-34661.84) | 25133.28 (16616.72-34478.09) | 25190 (16717.62-34527.75) | 25273.32 (16804.9-34583.04) | 25341.36 (16829.96-34744.95) | 25372.51 (16863.15-34759.53) | 25374.69 (16842.86-34687.71) | 25368.91 (16862.01-34662.21) | 25389.46 (16944.34-34649.38) | 25426.53 (17025.47-34794.6) |
| France | 0.41  (0.32 - 0.51) | 19193.3 (14358.34-24682.68) | 19331.09 (14513.1-24637.74) | 19450.96 (14706.87-24661.1) | 19547.93 (14803.38-24805.13) | 19601.3 (14865.95-24883.9) | 19602.12 (14795.17-24929.03) | 18864.67 (14299.01-23899.51) | 17135.23 (12700.3-22030.51) | 15055.5 (10749.87-20136.38) | 13261.58 (8990.89-18701.68) |
| Japan | 0.39  (-0.03 - 0.82) | 26765.83 (19977.99-34061.37) | 26689.9 (19923-33921.36) | 26583.52 (19856.39-33755.42) | 26463.01 (19797.28-33585.29) | 26345.4 (19719.72-33421.57) | 26261.59 (19663.13-33332.55) | 26127.28 (19738.91-32940.65) | 25852.5 (19615.56-32549.17) | 25508.63 (19360.93-32085.71) | 25175.77 (19078.82-31639.57) |
| Mexico | 0.39  (0.27 - 0.5) | 24148.33 (16176.32-32780.35) | 24178.95 (16186.69-32823.85) | 24224.13 (16190.74-32905.58) | 24296.25 (16230.85-33040.12) | 24396.51 (16276.29-33196.95) | 24512.62 (16320.2-33335.54) | 24658.93 (16470.57-33481.87) | 24839.38 (16668.41-33620.61) | 25021.74 (16857.59-33791.84) | 25170 (16932.04-33984.25) |
| Singapore | -0.36  (-0.49 - -0.24) | 24964.76 (17177.29-33247.2) | 23956.36 (17160.72-31341.56) | 23125.04 (16897.48-29855.32) | 22496.66 (16612.68-28796.39) | 22088.43 (16307.88-28246.29) | 21979.96 (16046.23-28464.33) | 22025.92 (16167.65-28574.15) | 22002.76 (16161.22-28577.16) | 21864.44 (16055.65-28410.11) | 21614.74 (15831.1-28102.65) |
| Haiti | -0.33  (-0.34 - -0.31) | 29106.1 (20231.59-37985.12) | 29150.55 (20283.83-38086.48) | 29190.68 (20316.87-38170.07) | 29213.73 (20305.05-38229.67) | 29214.63 (20328.2-38292.87) | 29203.06 (20350.07-38218.83) | 29180.88 (20391.73-38111.6) | 29153.59 (20427.68-37986.35) | 29124.08 (20435.25-37975.27) | 29091.38 (20440.41-38015.33) |
| Israel | 0.32  (0.27 - 0.38) | 24848.11 (17587.29-33037.84) | 24768.09 (17525.95-32812.09) | 24668.19 (17403.94-32585.41) | 24574.58 (17297.83-32565.84) | 24488.21 (17035.39-32486.51) | 24392.9 (16881.03-32561.82) | 24152.8 (16511.93-32284.06) | 23746.32 (16161.38-31952.76) | 23323.92 (15684.6-31717.53) | 23025.43 (15185.72-32018.12) |
| Qatar | 0.31  (0.23 - 0.39) | 27209 (18776.93-36447.55) | 27281.05 (18705.1-36473.8) | 27359.88 (18635.65-36572.91) | 27451.3 (18663.87-36652.04) | 27544.28 (18706.42-36669.65) | 27608.39 (18745.91-36850.36) | 27621.4 (18736.41-36828.58) | 27584.51 (18772.21-36724.73) | 27535.43 (18847.65-36660.02) | 27503.71 (18701.94-36787.98) |
| Republic of Korea | 0.31  (-0.11 - 0.73) | 37146.82 (28423.15-46220.49) | 36757.44 (28193.79-46006.45) | 36516.23 (28044.36-45869.86) | 36451.07 (27989.75-46052.28) | 36553.05 (28019.18-46275.36) | 36713.37 (28089.07-46404.88) | 37039.63 (28434.06-46626.58) | 37550.5 (28965.42-47224.35) | 38137.07 (29413.15-47491.54) | 38660.35 (29968.53-47869.96) |
| Italy | -0.25  (-0.32 - -0.18) | 20496.61 (14339.58-27538.21) | 20160.7 (14284.31-26825.23) | 19843.63 (14249.82-26350.92) | 19574.38 (14071.71-25716.91) | 19367.3 (13912.19-25352.33) | 19219.26 (13806.86-25270.35) | 19016.52 (13733.41-24939.44) | 18721.44 (13575.54-24510.92) | 18409.36 (13375.26-24011.92) | 18141.38 (13203.46-23589.61) |
| Morocco | -0.25  (-0.28 - -0.21) | 27099.48 (18461.98-36490.4) | 27042.79 (18351.1-36367.27) | 26986.74 (18251.93-36081.95) | 26935.27 (18203.55-36139.97) | 26893.68 (18059.47-36310.01) | 26862.97 (18000.86-36427.45) | 26846.67 (18079.81-36392.83) | 26840.08 (18109.35-36390.49) | 26831.92 (18102.12-36337.3) | 26812.08 (18068.89-36402.65) |
| Lithuania | 0.25  (-0.32 - 0.82) | 45682.02 (35131.08-54088.43) | 43814.29 (33905.15-52175.19) | 42500.67 (32873.59-50956.28) | 41719.98 (32468.04-50316.75) | 41346.22 (31966.22-50096.88) | 41242.6 (31940.07-50480.29) | 41706.77 (33031.85-50366.46) | 42812.65 (35031.59-50826) | 44136.52 (36915.76-51651.67) | 45249.79 (38317.66-52407.67) |
| United Arab Emirates | 0.25  (0.08 - 0.41) | 27814.76 (19165.15-36969.7) | 27977.58 (19372.17-37271.73) | 28121.63 (19460.57-37539.25) | 28234.76 (19546.58-37762.09) | 28308.53 (19507.68-37788.17) | 28346.83 (19508.88-37919.09) | 28080.95 (20217.88-36507.01) | 27413.39 (20567.76-34926) | 26607.01 (20209.95-33597.5) | 25913 (19729.17-32531.39) |
| Algeria | -0.24  (-0.27 - -0.2) | 26964.54 (18095.28-36576.55) | 26940.57 (18029.13-36736.72) | 26925.84 (18037.69-36778.33) | 26929.88 (18032.16-36647.91) | 26951.94 (18047.82-36621.01) | 26982.95 (18028.6-36603.2) | 27011.86 (18037.08-36563.5) | 27030.89 (18036.9-36603.24) | 27038.1 (18064.19-36668.74) | 27033.73 (18069.89-36840.59) |
| Egypt | -0.24  (-0.31 - -0.17) | 26925.45 (18087.85-36563.17) | 26978.24 (18086.05-36522.03) | 27028.6 (18134.13-36423.13) | 27071.84 (18188.48-36494.52) | 27089.47 (18127.38-36520.96) | 27071.2 (18069.2-36599.27) | 27012.89 (18110.94-36446.66) | 26922.62 (18026.64-36417.83) | 26826.9 (17894.95-36495.34) | 26756.71 (17781.48-36563.36) |
| Iraq | -0.21  (-0.29 - -0.14) | 26664.18 (17453.92-36687.11) | 26775.69 (17731.19-36582.26) | 26845.63 (17888.07-36409.7) | 26857.66 (17933.44-36196.4) | 26856.98 (17970.79-36124.8) | 26879.77 (18049.46-36226.21) | 26903.94 (18108.66-36104.07) | 26894.27 (18192.18-35998.89) | 26860.47 (18159.13-35974.53) | 26823.33 (18085.17-35983.84) |
| Portugal | -0.21  (-0.29 - -0.12) | 20359.79 (13694.99-28455.99) | 20215.62 (13668.17-28162.02) | 20100.68 (13658.88-27992.33) | 20024.32 (13627.02-27773.27) | 19990.14 (13619.24-27873.99) | 19966.92 (13597.21-27973.45) | 19915.23 (13521.85-27845.72) | 19836.62 (13417.91-27729.04) | 19770.99 (13341.69-27570.36) | 19727.92 (13286.26-27466.72) |
| Afghanistan | -0.2  (-0.26 - -0.13) | 26866.41 (18324.84-36297.38) | 26941.65 (18342.71-36232.19) | 26934.97 (18238.57-36148.51) | 26828.82 (18050.12-36089.42) | 26844.88 (18063.56-36279.82) | 27023.83 (18181.1-36803.97) | 27239.36 (18285.45-36964.47) | 27394.88 (18361.05-37004.16) | 27474.13 (18412.6-37162.52) | 27527.21 (18483.54-37274.28) |
| Paraguay | 0.19  (0.07 - 0.31) | 27429.86 (18345.37-36865.56) | 27383.69 (18407.47-36737.23) | 27380.56 (18448.66-36629.59) | 27413.53 (18474.4-36692.73) | 27466.51 (18525.4-36701.24) | 27526.02 (18602.9-36932.38) | 27968.22 (19309.01-36994.06) | 28944.04 (20323.25-37885.14) | 30103.75 (21477.9-39138.96) | 31093.76 (22185.97-40567.4) |
| Serbia | 0.19  (-0.93 - 1.32) | 31973.05 (22623.5-40926.68) | 31961.84 (22683.23-41011.61) | 31996.58 (22728.13-41228.64) | 32028.01 (22809.35-41303.01) | 32120.33 (22896.2-41645.24) | 32256.02 (23061.57-41919.56) | 32912.7 (24067.07-42040.3) | 34365.6 (25799.36-43157.91) | 36107.76 (27640.61-44843.3) | 37520.14 (29070.47-46479.63) |
| Romania | 0.17  (0 - 0.33) | 47235.9 (39336.99-55517.88) | 47651.68 (39777.26-55831.08) | 47803.84 (39858.34-55921.3) | 47683 (39733.51-55688.51) | 47444.38 (39544.75-55383.89) | 47196.86 (39302.99-55105.72) | 47014.09 (39135.21-54862.67) | 46824.28 (39031.45-54747.29) | 46677 (38908.57-54682.26) | 46622 (38870.53-54716.58) |
| Libya | -0.15  (-0.2 - -0.09) | 26393.05 (17623.74-35832.24) | 26477.9 (17728.58-35917.4) | 26526.54 (17901.51-35984.46) | 26545.92 (17985.35-36103.7) | 26538.44 (17948.17-36153.87) | 26512.55 (17877.27-36305.74) | 26449.66 (17820.28-35926.21) | 26365.96 (17739.74-35573.97) | 26290.95 (17739.22-35602.8) | 26225.8 (17585.19-35627) |
| Brazil | 0.15  (0.07 - 0.22) | 25298.25 (17311.35-33628.92) | 25086.47 (17152.02-33422.09) | 24843.51 (17008.02-33184.36) | 24592.79 (16867.56-32806.6) | 24365.67 (16751.63-32564.15) | 24183.81 (16628.54-32418.09) | 23982.06 (16468.11-32204.62) | 23724.94 (16280.11-31897.71) | 23467.1 (16106.11-31563.05) | 23264.85 (15945.47-31302.99) |
| Jordan | 0.15  (0.11 - 0.18) | 24830.93 (18553.44-32844.04) | 24956.17 (18825.61-32896.63) | 25073.72 (19032.08-32736.41) | 25184.37 (19106.44-32903.45) | 25281.71 (19123.01-33072.59) | 25353.32 (19120.34-33239.89) | 25411.84 (19250.66-33459) | 25467.24 (19376.45-33660.91) | 25503.74 (19526.16-33727.81) | 25514.55 (19511.7-34065.05) |
| India | -0.14  (-0.17 - -0.1) | 28555.46 (20089.9-37383.75) | 28661.72 (20225.2-37482.11) | 28773.5 (20376.37-37592.47) | 28882.17 (20530.9-37703.18) | 28980.16 (20644.87-37774.69) | 29059.59 (20721.44-37869.32) | 29185.72 (20852.51-37934.01) | 29395.57 (21105.05-38082.26) | 29632.04 (21338.56-38320.41) | 29840.06 (21526.21-38492.01) |
| Saint Lucia | -0.14  (-0.16 - -0.11) | 28988.99 (20316.14-38451.96) | 29033.89 (20439.97-38228.35) | 29086.16 (20457.81-38293.51) | 29156.34 (20481.11-38249.87) | 29239.07 (20415.57-38207.45) | 29323.18 (20425.47-38456.66) | 29397.7 (20607.35-38225.06) | 29453.22 (20811.39-38078.37) | 29476.43 (20900.57-37890.36) | 29465.58 (20964.1-37874.73) |
| Bahamas | 0.11  (0 - 0.22) | 27023.89 (18930.98-35638.37) | 26815.77 (18748.77-35352.48) | 26709.27 (18722.62-35124.19) | 26854.35 (18830.29-35131.78) | 27308.4 (19128.72-35625.19) | 27886.14 (19521.85-36468.06) | 28262.71 (19806.22-36956.52) | 28264.02 (19860.45-36996.96) | 27984.39 (19716.35-36621.89) | 27598.93 (19293.37-36096.32) |
| Palestine | -0.1  (-0.41 - 0.22) | 24181.8 (17090.15-31869.02) | 23659.91 (16589.27-31305.45) | 23225.03 (16175.09-30974.69) | 22893.83 (15869.28-30588.88) | 22684.47 (15654.15-30377.75) | 22611.77 (15498.98-30317.9) | 22683.65 (15591.55-30360.62) | 22858.25 (15857.52-30543.08) | 23074.8 (16117.13-30740.56) | 23271.23 (16352.92-31007.2) |
| Peru | -0.09  (-0.11 - -0.08) | 28006.51 (19347.84-37043.65) | 28003.24 (19345.13-36974.26) | 27989.93 (19480.86-36875.07) | 27965.11 (19514.84-36736.26) | 27954.55 (19412.41-36671.19) | 27966.58 (19400.38-36614.29) | 27983.82 (19436.71-36593.24) | 27985.27 (19445.68-36472.34) | 27966.01 (19462.69-36415.53) | 27935.72 (19375.51-36409.68) |
| Malta | -0.08  (-0.12 - -0.04) | 20404.83 (12926.34-28925.29) | 20437.41 (12951.7-28891.51) | 20469.45 (12943.66-28981.16) | 20497.92 (12926.47-29017.98) | 20514.24 (12905.97-29060.95) | 20511.67 (12863.56-28886.34) | 20447.65 (12884.81-29024.03) | 20361.85 (12924.2-28841.4) | 20305.13 (12921.36-28797.08) | 20301 (13022.31-28685.11) |
| Niue | -0.08  (-0.12 - -0.05) | 31722.12 (22679.12-41081.72) | 31675.87 (22654.27-41082.08) | 31656.82 (22718.85-40866.5) | 31597.57 (22607.54-40772.69) | 31500.31 (22415.81-40908.18) | 31413.46 (22345.35-40806.45) | 31360.79 (22284.59-40835.55) | 31330.02 (22226.81-40768) | 31313.81 (22189.78-40795.01) | 31338.92 (22179.09-40867.14) |
| Bolivia (Plurinational State of) | -0.07  (-0.1 - -0.04) | 28017.96 (19600.94-36771.26) | 28098 (19673.02-37009.86) | 28163.95 (19712.37-37001.24) | 28203.21 (19712.39-37198.8) | 28225.9 (19695.25-37401.89) | 28247.8 (19696.43-37536.61) | 28258.92 (19712.87-37455.78) | 28246.66 (19736.43-37191.43) | 28216.18 (19799.3-37072.49) | 28174.36 (19698.47-37038.1) |
| Ghana | -0.07  (-0.1 - -0.05) | 25031.27 (16600.68-34088.49) | 24965.14 (16625.4-34031.87) | 24910.42 (16629.8-34034.15) | 24873.48 (16597.74-34053.5) | 24856.09 (16561.45-34089.22) | 24857.43 (16540.73-34115.94) | 24884 (16571.66-34094.38) | 24934.34 (16625.03-34138.23) | 24991.66 (16663.42-34274.96) | 25038.3 (16626.9-34293.22) |
| Grenada | -0.07  (-0.09 - -0.06) | 29378.92 (20465.15-38366) | 29317.07 (20481.78-38338.15) | 29243.13 (20530.63-38332.68) | 29150.7 (20556.61-38380.67) | 29052.53 (20449.8-38137.03) | 28980.01 (20406.26-38140.38) | 28935.33 (20305.38-37898.91) | 28895.52 (20201.87-37826.45) | 28850.33 (20088.25-37706.03) | 28803.84 (19947.14-37661.48) |
| Zambia | 0.07  (0.04 - 0.1) | 26277.95 (17790.1-35320.45) | 26332.37 (17893.6-35184.92) | 26392.83 (17918.23-35097.02) | 26450.43 (17882.18-35220.84) | 26503.16 (17920.93-35391.25) | 26552.04 (17894.34-35740.37) | 26604.19 (18008.95-35472.33) | 26665.18 (18095.45-35507.96) | 26727.65 (18123.29-35516.91) | 26783.87 (18133.04-35623.8) |
| Antigua and Barbuda | -0.06  (-0.09 - -0.03) | 28905.89 (20176.7-38450.5) | 28924.52 (20248.94-38357.43) | 28940.87 (20306.18-38341.31) | 28945.35 (20249.03-38179.03) | 28930.4 (20183.42-38102.97) | 28901.59 (20152.02-38001.04) | 28856.84 (20139.61-37790.84) | 28802.23 (20150.68-37692.16) | 28752.71 (20069.97-37567.42) | 28711.3 (19900.1-37504.42) |
| Seychelles | -0.06  (-0.08 - -0.04) | 30211.1 (21164.49-39826.27) | 30158.24 (21141.53-39410.57) | 30103.54 (21135.6-39422.33) | 30071.96 (21128.07-39493.62) | 30077.98 (21122.55-39503.89) | 30123.1 (21083.38-39707.99) | 30198.72 (21102.76-39722.93) | 30284.49 (21099.56-39716.6) | 30348.12 (21118.5-39819.98) | 30358.65 (21075.39-39833.91) |
| Dominica | 0.06  (0 - 0.11) | 28691.56 (20023.82-37795.67) | 28691.85 (20122.36-37576.42) | 28693.66 (20128.57-37587.13) | 28760.38 (20213.44-37730.81) | 28915.94 (20331.13-37870.26) | 29121.48 (20440.21-38164.81) | 29309.62 (20608.53-38370.51) | 29432.67 (20673.4-38467.12) | 29458.79 (20616.91-38430.26) | 29396.95 (20450.51-38302.57) |
| Bahrain | 0.05  (0 - 0.1) | 26119.99 (17410.01-35551.95) | 26242.97 (17499.09-35582.9) | 26290.01 (17531.21-35640.9) | 26274.65 (17485.89-35609.65) | 26292.01 (17532.89-35733.89) | 26329.14 (17535.89-35893.74) | 26387.07 (17569.62-35978.27) | 26403.6 (17643.98-35889.32) | 26437.72 (17740.43-35814.89) | 26513.28 (17796.63-35846.94) |
| Turkey | 0.04  (-0.02 - 0.09) | 27057.72 (18191.33-36396.8) | 27350.07 (18715.03-36672.72) | 27625.52 (19071.53-36977.71) | 27884.05 (19436.24-37150.82) | 28120.29 (19689.68-37582.67) | 28325.48 (19873.12-38050.73) | 28574.57 (20444.21-37811.98) | 28902.35 (21204.04-37564.55) | 29245.19 (21856.82-37160) | 29524.07 (22362.08-36894.18) |
| Mauritania | -0.03  (-0.06 - -0.01) | 24944.96 (16414.42-34250.87) | 24907.45 (16428.51-34053.43) | 24888.88 (16379.68-34040.38) | 24884.12 (16432.13-34140.77) | 24889.03 (16514.11-34232.32) | 24898.47 (16547.98-34264.62) | 24923.03 (16544.65-34249.53) | 24968.45 (16573-34282.67) | 25019.64 (16610.07-34261.71) | 25060.14 (16693.77-34301.22) |
| El Salvador | 0.03  (0.01 - 0.04) | 25456.68 (17018.59-34664.35) | 25455.89 (16968.48-34632.6) | 25458.94 (16973.89-34660.98) | 25476.9 (17007.66-34895.93) | 25523.66 (16907.43-35070.23) | 25600.29 (16806.59-35196.95) | 25689.3 (17009.19-35331.75) | 25779.77 (17201.85-35412.21) | 25870.37 (17206.68-35350.17) | 25951.11 (17369.3-35362.03) |
| Uganda | 0.03  (-0.1 - 0.17) | 25934.89 (17404.63-34660.68) | 24641 (17140.91-32447.5) | 23556.47 (16932.33-30786.85) | 22715.28 (16457.54-29687.83) | 22152.17 (16000.83-29059.6) | 21904.19 (15689.37-28988.73) | 22069.52 (16045.12-29084.11) | 22577.2 (16497.21-29573.21) | 23209.01 (16993.12-30095.2) | 23740.95 (17362.04-30601.15) |
| Luxembourg | -0.02  (-0.05 - 0.01) | 19657.63 (12295.94-27569.42) | 19678.34 (12379.49-27560.34) | 19702.69 (12376.76-27607.69) | 19726.83 (12339.96-27681.18) | 19728.89 (12287.74-27718.37) | 19745.39 (12299.47-27718.15) | 19760.72 (12273.68-27881.57) | 19776.51 (12356.5-27940.44) | 19775.61 (12437.18-27916.85) | 19774.09 (12481.42-27978.53) |
| Guatemala | 0.02  (-0.02 - 0.07) | 25500.32 (16991.68-34513.43) | 25536.32 (17125.12-34472.35) | 25597.26 (17257.95-34548.11) | 25667.88 (17289.47-34690.46) | 25727.65 (17315.15-34777.13) | 25741.78 (17244.06-34791.78) | 25712.8 (17282-34637.69) | 25690.96 (17222.94-34570.34) | 25707.85 (17194.5-34501.68) | 25732.5 (17185.18-34495.36) |
| Republic of Moldova | 0.02  (0 - 0.04) | 32278.35 (22855.76-42259.06) | 32476.41 (23090.36-42457.35) | 32627.37 (23258.77-42492.62) | 32740.13 (23333.07-42553.44) | 32830.68 (23320.23-42769.53) | 32905.59 (23360.35-43038.89) | 32960.58 (23413.57-43053.65) | 32992.67 (23455.52-43244.64) | 33001.67 (23485.93-43292.09) | 32983.95 (23461.43-43289.94) |
| United States Virgin Islands | 0.02  (0 - 0.04) | 28397.99 (19788.83-37128.03) | 28366.95 (19835.88-36977.35) | 28343.02 (19842.5-36977.58) | 28330.93 (19881.66-37073.12) | 28331.56 (19790.54-37266.47) | 28342.41 (19699.16-37478.18) | 28341.46 (19751.02-37313.12) | 28313.63 (19780.42-37343.9) | 28267.63 (19618.92-37294.95) | 28221.18 (19608.78-37061.77) |
| Belize | -0.01  (-0.03 - 0.01) | 28890.9 (20268.94-38201.37) | 28945.31 (20352.04-38024.6) | 29014.66 (20361.34-38062.76) | 29090.12 (20376.17-38080.26) | 29151 (20410.94-38182.29) | 29179.04 (20383.56-38323.92) | 29167.03 (20416.62-38240.51) | 29113.91 (20438.27-38111.35) | 29030.29 (20409.24-38128.77) | 28936.57 (20328.7-38137.24) |
| Costa Rica | -0.01  (-0.04 - 0.02) | 25820.54 (16965.97-34957.22) | 25775.71 (16944.02-34834.81) | 25746.89 (16957.67-34743.74) | 25718.11 (16996.79-34596.58) | 25685.2 (16974.1-34479.55) | 25663.05 (16996.66-34414.27) | 25666.64 (17042.24-34473.21) | 25690.32 (17095.87-34664.29) | 25708.54 (17121.32-34849.24) | 25709.35 (17119.61-34971.93) |
| Suriname | -0.01  (-0.13 - 0.1) | 30937.38 (21602.93-40511.78) | 30853.88 (21698.91-40061.17) | 30797.08 (21818.32-39796.55) | 30779.29 (21839.27-39584.92) | 30784.05 (21825.46-39650.1) | 30791.11 (21726.71-39645.84) | 31512.34 (22593.3-40166.45) | 33224.98 (24183.26-41860.46) | 35276.97 (26061.15-44210.2) | 37016.3 (27516.53-46455.08) |
| Uruguay | -0.01  (-0.09 - 0.07) | 28166.33 (19600.67-37045.05) | 28260.05 (19686.51-37092.24) | 28346.91 (19806.23-37226.5) | 28353 (19850.12-37282.53) | 28291.83 (19738.2-37108.75) | 28217.84 (19515.24-37071.04) | 28165.65 (19586.51-36932.16) | 28171.93 (19620.18-36893.14) | 28237.78 (19611.22-36956.43) | 28310.28 (19596.59-37023.54) |
| Uzbekistan | -0.01  (-0.02 - -0.01) | 31151.51 (22482.63-40141.56) | 31241.73 (22728.53-40419.12) | 31333.11 (22756.39-40455.44) | 31428.5 (22795.19-40560.71) | 31518.38 (22889.46-40729.92) | 31590.48 (22933.42-40952.65) | 31644.48 (22945.26-40912.81) | 31686.84 (22895.58-40983.84) | 31714.26 (22940.66-40976.5) | 31720.07 (23012.97-41039.49) |
| San Marino | 0.01  (-0.01 - 0.03) | 19859.8 (12393.78-28489.39) | 19864.38 (12426.23-28285.36) | 19860.15 (12396.76-28072.08) | 19852 (12399.22-27997.03) | 19845.16 (12427.37-27967.11) | 19839.9 (12424.68-28012.9) | 19810.43 (12441.5-27939.4) | 19745.05 (12405.97-27840.4) | 19666.59 (12325.77-27718.47) | 19600.15 (12303.19-27707.6) |
| Tuvalu | 0.01  (0 - 0.01) | 31312.37 (22301.34-40614.24) | 31261.94 (22267.69-40509.35) | 31220.14 (22207.05-40591.73) | 31188.49 (22100.36-40522.4) | 31174.42 (22063.48-40667.14) | 31190.58 (21997.82-40856.34) | 31237.14 (22043.25-40665.28) | 31295 (22006.32-40497.46) | 31343.07 (22147.97-40458.38) | 31373.91 (22241.71-40505.43) |
| Guyana | 0  (-0.02 - 0.01) | 28690.23 (20132.39-37934.28) | 28744.74 (20268.01-37848.71) | 28794.85 (20380.3-37661.65) | 28850.7 (20430.07-37719.6) | 28921.39 (20383.85-37899.7) | 29007.85 (20346.77-38230.49) | 29072.62 (20397.04-38054.66) | 29099.31 (20420.7-38012.59) | 29105.79 (20390.49-38072.99) | 29112.77 (20402.54-38237.36) |
| Jamaica | 0  (-0.02 - 0.02) | 28979.07 (20231.92-38546.65) | 28994.5 (20276.95-38213.62) | 29033.38 (20284.17-37994.63) | 29090.39 (20292.51-38034.06) | 29154.79 (20307.12-38171.38) | 29215.61 (20369.04-38257.2) | 29262.93 (20461.34-38334.4) | 29296.33 (20486.1-38498.14) | 29323.05 (20519.43-38615.98) | 29347.74 (20565.16-38692.55) |
| Tunisia | 0  (-0.02 - 0.02) | 27158.9 (18225.09-36599.94) | 27126.08 (18217.43-36450.31) | 27098.45 (18197.6-36420.04) | 27078.3 (18112.77-36392.34) | 27069.41 (18102.71-36597.9) | 27071.75 (18095.54-36708.89) | 27082.33 (18078.26-36507.67) | 27088.5 (18008.43-36433.47) | 27078.75 (17977.12-36784.69) | 27048.69 (17942.57-36953.05) |
| Albania | NA | 0 (0-0) | 0 (0-0) | 0 (0-0) | 0 (0-0) | 0 (0-0) | 0 (0-0) | 0 (0-0) | 0 (0-0) | 0 (0-0) | 0 (0-0) |
| American Samoa | NA | 0 (0-0) | 0 (0-0) | 0 (0-0) | 0 (0-0) | 0 (0-0) | 0 (0-0) | 0 (0-0) | 0 (0-0) | 0 (0-0) | 0 (0-0) |
| Angola | NA | 0 (0-0) | 0 (0-0) | 0 (0-0) | 0 (0-0) | 0 (0-0) | 0 (0-0) | 0 (0-0) | 0 (0-0) | 0 (0-0) | 0 (0-0) |
| Bangladesh | NA | 0 (0-0) | 0 (0-0) | 0 (0-0) | 0 (0-0) | 0 (0-0) | 0 (0-0) | 0 (0-0) | 0 (0-0) | 0 (0-0) | 0 (0-0) |
| Barbados | NA | 0 (0-0) | 0 (0-0) | 0 (0-0) | 0 (0-0) | 0 (0-0) | 0 (0-0) | 0 (0-0) | 0 (0-0) | 0 (0-0) | 0 (0-0) |
| Belgium | NA | 0 (0-0) | 0 (0-0) | 0 (0-0) | 0 (0-0) | 0 (0-0) | 0 (0-0) | 0 (0-0) | 0 (0-0) | 0 (0-0) | 0 (0-0) |
| Benin | NA | 0 (0-0) | 0 (0-0) | 0 (0-0) | 0 (0-0) | 0 (0-0) | 0 (0-0) | 0 (0-0) | 0 (0-0) | 0 (0-0) | 0 (0-0) |
| Bermuda | NA | 0 (0-0) | 0 (0-0) | 0 (0-0) | 0 (0-0) | 0 (0-0) | 0 (0-0) | 0 (0-0) | 0 (0-0) | 0 (0-0) | 0 (0-0) |
| Bosnia and Herzegovina | NA | 0 (0-0) | 0 (0-0) | 0 (0-0) | 0 (0-0) | 0 (0-0) | 0 (0-0) | 0 (0-0) | 0 (0-0) | 0 (0-0) | 0 (0-0) |
| Botswana | NA | 0 (0-0) | 0 (0-0) | 0 (0-0) | 0 (0-0) | 0 (0-0) | 0 (0-0) | 0 (0-0) | 0 (0-0) | 0 (0-0) | 0 (0-0) |
| Bulgaria | NA | 0 (0-0) | 0 (0-0) | 0 (0-0) | 0 (0-0) | 0 (0-0) | 0 (0-0) | 0 (0-0) | 0 (0-0) | 0 (0-0) | 0 (0-0) |
| Burkina Faso | NA | 0 (0-0) | 0 (0-0) | 0 (0-0) | 0 (0-0) | 0 (0-0) | 0 (0-0) | 0 (0-0) | 0 (0-0) | 0 (0-0) | 0 (0-0) |
| Cabo Verde | NA | 0 (0-0) | 0 (0-0) | 0 (0-0) | 0 (0-0) | 0 (0-0) | 0 (0-0) | 0 (0-0) | 0 (0-0) | 0 (0-0) | 0 (0-0) |
| Cameroon | NA | 0 (0-0) | 0 (0-0) | 0 (0-0) | 0 (0-0) | 0 (0-0) | 0 (0-0) | 0 (0-0) | 0 (0-0) | 0 (0-0) | 0 (0-0) |
| Central African Republic | NA | 0 (0-0) | 0 (0-0) | 0 (0-0) | 0 (0-0) | 0 (0-0) | 0 (0-0) | 0 (0-0) | 0 (0-0) | 0 (0-0) | 0 (0-0) |
| Chad | NA | 0 (0-0) | 0 (0-0) | 0 (0-0) | 0 (0-0) | 0 (0-0) | 0 (0-0) | 0 (0-0) | 0 (0-0) | 0 (0-0) | 0 (0-0) |
| Colombia | NA | 0 (0-0) | 0 (0-0) | 0 (0-0) | 0 (0-0) | 0 (0-0) | 0 (0-0) | 0 (0-0) | 0 (0-0) | 0 (0-0) | 0 (0-0) |
| Comoros | NA | 0 (0-0) | 0 (0-0) | 0 (0-0) | 0 (0-0) | 0 (0-0) | 0 (0-0) | 0 (0-0) | 0 (0-0) | 0 (0-0) | 0 (0-0) |
| Congo | NA | 0 (0-0) | 0 (0-0) | 0 (0-0) | 0 (0-0) | 0 (0-0) | 0 (0-0) | 0 (0-0) | 0 (0-0) | 0 (0-0) | 0 (0-0) |
| Cook Islands | NA | 0 (0-0) | 0 (0-0) | 0 (0-0) | 0 (0-0) | 0 (0-0) | 0 (0-0) | 0 (0-0) | 0 (0-0) | 0 (0-0) | 0 (0-0) |
| Coted'Ivoire | NA | 0 (0-0) | 0 (0-0) | 0 (0-0) | 0 (0-0) | 0 (0-0) | 0 (0-0) | 0 (0-0) | 0 (0-0) | 0 (0-0) | 0 (0-0) |
| Croatia | NA | 0 (0-0) | 0 (0-0) | 0 (0-0) | 0 (0-0) | 0 (0-0) | 0 (0-0) | 0 (0-0) | 0 (0-0) | 0 (0-0) | 0 (0-0) |
| Cuba | NA | 0 (0-0) | 0 (0-0) | 0 (0-0) | 0 (0-0) | 0 (0-0) | 0 (0-0) | 0 (0-0) | 0 (0-0) | 0 (0-0) | 0 (0-0) |
| Cyprus | NA | 0 (0-0) | 0 (0-0) | 0 (0-0) | 0 (0-0) | 0 (0-0) | 0 (0-0) | 0 (0-0) | 0 (0-0) | 0 (0-0) | 0 (0-0) |
| Dominican Republic | NA | 0 (0-0) | 0 (0-0) | 0 (0-0) | 0 (0-0) | 0 (0-0) | 0 (0-0) | 0 (0-0) | 0 (0-0) | 0 (0-0) | 0 (0-0) |
| Eswatini | NA | 0 (0-0) | 0 (0-0) | 0 (0-0) | 0 (0-0) | 0 (0-0) | 0 (0-0) | 0 (0-0) | 0 (0-0) | 0 (0-0) | 0 (0-0) |
| Ethiopia | NA | 0 (0-0) | 0 (0-0) | 0 (0-0) | 0 (0-0) | 0 (0-0) | 0 (0-0) | 0 (0-0) | 0 (0-0) | 0 (0-0) | 0 (0-0) |
| Gabon | NA | 0 (0-0) | 0 (0-0) | 0 (0-0) | 0 (0-0) | 0 (0-0) | 0 (0-0) | 0 (0-0) | 0 (0-0) | 0 (0-0) | 0 (0-0) |
| Gambia | NA | 0 (0-0) | 0 (0-0) | 0 (0-0) | 0 (0-0) | 0 (0-0) | 0 (0-0) | 0 (0-0) | 0 (0-0) | 0 (0-0) | 0 (0-0) |
| Greenland | NA | 0 (0-0) | 0 (0-0) | 0 (0-0) | 0 (0-0) | 0 (0-0) | 0 (0-0) | 0 (0-0) | 0 (0-0) | 0 (0-0) | 0 (0-0) |
| Guam | NA | 0 (0-0) | 0 (0-0) | 0 (0-0) | 0 (0-0) | 0 (0-0) | 0 (0-0) | 0 (0-0) | 0 (0-0) | 0 (0-0) | 0 (0-0) |
| Guinea | NA | 0 (0-0) | 0 (0-0) | 0 (0-0) | 0 (0-0) | 0 (0-0) | 0 (0-0) | 0 (0-0) | 0 (0-0) | 0 (0-0) | 0 (0-0) |
| Guinea-Bissau | NA | 0 (0-0) | 0 (0-0) | 0 (0-0) | 0 (0-0) | 0 (0-0) | 0 (0-0) | 0 (0-0) | 0 (0-0) | 0 (0-0) | 0 (0-0) |
| Hungary | NA | 0 (0-0) | 0 (0-0) | 0 (0-0) | 0 (0-0) | 0 (0-0) | 0 (0-0) | 0 (0-0) | 0 (0-0) | 0 (0-0) | 0 (0-0) |
| Iran (Islamic Republic of) | NA | 0 (0-0) | 0 (0-0) | 0 (0-0) | 0 (0-0) | 0 (0-0) | 0 (0-0) | 0 (0-0) | 0 (0-0) | 0 (0-0) | 0 (0-0) |
| Kenya | NA | 0 (0-0) | 0 (0-0) | 0 (0-0) | 0 (0-0) | 0 (0-0) | 0 (0-0) | 0 (0-0) | 0 (0-0) | 0 (0-0) | 0 (0-0) |
| Lebanon | NA | 0 (0-0) | 0 (0-0) | 0 (0-0) | 0 (0-0) | 0 (0-0) | 0 (0-0) | 0 (0-0) | 0 (0-0) | 0 (0-0) | 0 (0-0) |
| Liberia | NA | 0 (0-0) | 0 (0-0) | 0 (0-0) | 0 (0-0) | 0 (0-0) | 0 (0-0) | 0 (0-0) | 0 (0-0) | 0 (0-0) | 0 (0-0) |
| Malawi | NA | 0 (0-0) | 0 (0-0) | 0 (0-0) | 0 (0-0) | 0 (0-0) | 0 (0-0) | 0 (0-0) | 0 (0-0) | 0 (0-0) | 0 (0-0) |
| Mali | NA | 0 (0-0) | 0 (0-0) | 0 (0-0) | 0 (0-0) | 0 (0-0) | 0 (0-0) | 0 (0-0) | 0 (0-0) | 0 (0-0) | 0 (0-0) |
| Mauritius | NA | 0 (0-0) | 0 (0-0) | 0 (0-0) | 0 (0-0) | 0 (0-0) | 0 (0-0) | 0 (0-0) | 0 (0-0) | 0 (0-0) | 0 (0-0) |
| Monaco | NA | 0 (0-0) | 0 (0-0) | 0 (0-0) | 0 (0-0) | 0 (0-0) | 0 (0-0) | 0 (0-0) | 0 (0-0) | 0 (0-0) | 0 (0-0) |
| Namibia | NA | 0 (0-0) | 0 (0-0) | 0 (0-0) | 0 (0-0) | 0 (0-0) | 0 (0-0) | 0 (0-0) | 0 (0-0) | 0 (0-0) | 0 (0-0) |
| Nauru | NA | 0 (0-0) | 0 (0-0) | 0 (0-0) | 0 (0-0) | 0 (0-0) | 0 (0-0) | 0 (0-0) | 0 (0-0) | 0 (0-0) | 0 (0-0) |
| Nepal | NA | 0 (0-0) | 0 (0-0) | 0 (0-0) | 0 (0-0) | 0 (0-0) | 0 (0-0) | 0 (0-0) | 0 (0-0) | 0 (0-0) | 0 (0-0) |
| Niger | NA | 0 (0-0) | 0 (0-0) | 0 (0-0) | 0 (0-0) | 0 (0-0) | 0 (0-0) | 0 (0-0) | 0 (0-0) | 0 (0-0) | 0 (0-0) |
| Nigeria | NA | 0 (0-0) | 0 (0-0) | 0 (0-0) | 0 (0-0) | 0 (0-0) | 0 (0-0) | 0 (0-0) | 0 (0-0) | 0 (0-0) | 0 (0-0) |
| Northern Mariana Islands | NA | 0 (0-0) | 0 (0-0) | 0 (0-0) | 0 (0-0) | 0 (0-0) | 0 (0-0) | 0 (0-0) | 0 (0-0) | 0 (0-0) | 0 (0-0) |
| Palau | NA | 0 (0-0) | 0 (0-0) | 0 (0-0) | 0 (0-0) | 0 (0-0) | 0 (0-0) | 0 (0-0) | 0 (0-0) | 0 (0-0) | 0 (0-0) |
| Puerto Rico | NA | 0 (0-0) | 0 (0-0) | 0 (0-0) | 0 (0-0) | 0 (0-0) | 0 (0-0) | 0 (0-0) | 0 (0-0) | 0 (0-0) | 0 (0-0) |
| Saint Kitts and Nevis | NA | 0 (0-0) | 0 (0-0) | 0 (0-0) | 0 (0-0) | 0 (0-0) | 0 (0-0) | 0 (0-0) | 0 (0-0) | 0 (0-0) | 0 (0-0) |
| Saint Vincent and the Grenadines | NA | 0 (0-0) | 0 (0-0) | 0 (0-0) | 0 (0-0) | 0 (0-0) | 0 (0-0) | 0 (0-0) | 0 (0-0) | 0 (0-0) | 0 (0-0) |
| Sao Tome and Principe | NA | 0 (0-0) | 0 (0-0) | 0 (0-0) | 0 (0-0) | 0 (0-0) | 0 (0-0) | 0 (0-0) | 0 (0-0) | 0 (0-0) | 0 (0-0) |
| Saudi Arabia | NA | 0 (0-0) | 0 (0-0) | 0 (0-0) | 0 (0-0) | 0 (0-0) | 0 (0-0) | 0 (0-0) | 0 (0-0) | 0 (0-0) | 0 (0-0) |
| Senegal | NA | 0 (0-0) | 0 (0-0) | 0 (0-0) | 0 (0-0) | 0 (0-0) | 0 (0-0) | 0 (0-0) | 0 (0-0) | 0 (0-0) | 0 (0-0) |
| Sierra Leone | NA | 0 (0-0) | 0 (0-0) | 0 (0-0) | 0 (0-0) | 0 (0-0) | 0 (0-0) | 0 (0-0) | 0 (0-0) | 0 (0-0) | 0 (0-0) |
| Somalia | NA | 0 (0-0) | 0 (0-0) | 0 (0-0) | 0 (0-0) | 0 (0-0) | 0 (0-0) | 0 (0-0) | 0 (0-0) | 0 (0-0) | 0 (0-0) |
| South Africa | NA | 0 (0-0) | 0 (0-0) | 0 (0-0) | 0 (0-0) | 0 (0-0) | 0 (0-0) | 0 (0-0) | 0 (0-0) | 0 (0-0) | 0 (0-0) |
| South Sudan | NA | 0 (0-0) | 0 (0-0) | 0 (0-0) | 0 (0-0) | 0 (0-0) | 0 (0-0) | 0 (0-0) | 0 (0-0) | 0 (0-0) | 0 (0-0) |
| Sudan | NA | 0 (0-0) | 0 (0-0) | 0 (0-0) | 0 (0-0) | 0 (0-0) | 0 (0-0) | 0 (0-0) | 0 (0-0) | 0 (0-0) | 0 (0-0) |
| Syrian Arab Republic | NA | 0 (0-0) | 0 (0-0) | 0 (0-0) | 0 (0-0) | 0 (0-0) | 0 (0-0) | 0 (0-0) | 0 (0-0) | 0 (0-0) | 0 (0-0) |
| Togo | NA | 0 (0-0) | 0 (0-0) | 0 (0-0) | 0 (0-0) | 0 (0-0) | 0 (0-0) | 0 (0-0) | 0 (0-0) | 0 (0-0) | 0 (0-0) |
| Tokelau | NA | 0 (0-0) | 0 (0-0) | 0 (0-0) | 0 (0-0) | 0 (0-0) | 0 (0-0) | 0 (0-0) | 0 (0-0) | 0 (0-0) | 0 (0-0) |
| United Kingdom | NA | 0 (0-0) | 0 (0-0) | 0 (0-0) | 0 (0-0) | 0 (0-0) | 0 (0-0) | 0 (0-0) | 0 (0-0) | 0 (0-0) | 0 (0-0) |
| United Republic of Tanzania | NA | 0 (0-0) | 0 (0-0) | 0 (0-0) | 0 (0-0) | 0 (0-0) | 0 (0-0) | 0 (0-0) | 0 (0-0) | 0 (0-0) | 0 (0-0) |
| Yemen | NA | 0 (0-0) | 0 (0-0) | 0 (0-0) | 0 (0-0) | 0 (0-0) | 0 (0-0) | 0 (0-0) | 0 (0-0) | 0 (0-0) | 0 (0-0) |
| Zimbabwe | NA | 0 (0-0) | 0 (0-0) | 0 (0-0) | 0 (0-0) | 0 (0-0) | 0 (0-0) | 0 (0-0) | 0 (0-0) | 0 (0-0) | 0 (0-0) |
| **Location Name** | 2000 | 2001 | 2002 | 2003 | 2004 | 2005 | 2006 | 2007 | 2008 | 2009 | 2010 |
| Australia | 14720.38 (13120.92-16557.52) | 14772.31 (13216.79-16511.6) | 14848.02 (13292.97-16577.57) | 14939.17 (13381.77-16690.91) | 15034.31 (13440.64-16815.3) | 15131.36 (13486.58-16995.99) | 15275.31 (13635.29-17122.26) | 15486.82 (13834.43-17389.8) | 15736.72 (14039.28-17687.82) | 15982.85 (14208.42-17997.93) | 16144.31 (14286.4-18368.41) |
| Madagascar | 33034.36 (27398.57-39542.48) | 31760.33 (26103.75-38104.69) | 29130.54 (23326.79-35568.61) | 26080.68 (19759.08-33317.08) | 23541.79 (16695.26-31773.91) | 22446.38 (14483.57-31535.56) | 22395.27 (14508.71-31265.23) | 22364.58 (14477.82-31115.98) | 22349.97 (14436.63-31156.31) | 22347.16 (14414.81-31005.29) | 22352.8 (14399.82-31131.48) |
| United States of America | 15123.44 (8947.96-21762.93) | 15020.8 (8888.9-21591.78) | 14974.95 (8872.83-21525.31) | 14949.11 (8859.94-21471.15) | 14923.99 (8839.06-21383.39) | 14888.5 (8815.44-21298.14) | 14857.09 (8811.48-21287.18) | 14858.37 (8823.79-21307.59) | 14897.26 (8853.82-21343.57) | 14958.5 (8944.62-21421.2) | 15009.31 (9009.47-21530.73) |
| Netherlands | 22640.78 (16647.41-29455.96) | 22603.87 (16654.72-29386.9) | 22578.87 (16624.3-29322.66) | 22560.97 (16602.12-29297.64) | 22541.51 (16557.05-29376.44) | 22542.12 (16568.77-29413.52) | 22534.29 (16505.82-29530.13) | 22487 (16415.02-29444.92) | 22403.17 (16333.89-29290.56) | 22315.9 (16291.05-29237.33) | 22272.48 (16258.88-29294.52) |
| Greece | 24694.48 (20291.13-29921.27) | 24121.97 (19925.9-29218.41) | 23459.82 (19391.15-28424.05) | 22823.24 (18780.96-27655.77) | 22315.79 (18314.93-27131.83) | 22050.77 (18022.26-26862.98) | 21964.02 (17970.82-26730.43) | 21911.33 (17963.23-26675.85) | 21875.54 (17947.85-26607.38) | 21857.78 (17930.65-26643.71) | 21852.58 (17890.1-26666.44) |
| Canada | 16866.32 (10578.62-24360.08) | 16809.46 (10553.59-24176.92) | 16760.07 (10567.6-24108.55) | 16712.73 (10570.37-24056.81) | 16666.19 (10574.8-24030.59) | 16614.36 (10549.29-24026.98) | 16549.86 (10498.26-23880.49) | 16462.92 (10407.16-23666.68) | 16398.2 (10342.92-23615.46) | 16390.51 (10347.22-23697.87) | 16451.62 (10394.61-23859.92) |
| Norway | 16482.87 (9872.87-23632.28) | 16476.58 (9896.18-23685.89) | 16451.66 (9894.26-23724.04) | 16407.21 (9881.01-23721.8) | 16344.53 (9849.81-23657.26) | 16284.55 (9811.28-23555.74) | 16239.83 (9798.96-23488.03) | 16200.25 (9784.38-23393.49) | 16165.48 (9769.32-23317.06) | 16143.46 (9764.32-23262.2) | 16149.15 (9783.24-23213.72) |
| Finland | 19986.76 (12582.7-28217.9) | 19990.4 (12567.33-28304.36) | 19976.78 (12543.32-28236.79) | 19941.11 (12510.68-28263.33) | 19885.07 (12441.94-28263.47) | 19852.41 (12410.73-28316.6) | 19863.68 (12463.42-28121.12) | 19903.75 (12428.03-28019.01) | 19947.69 (12490.33-27934.65) | 19975.12 (12440.21-27907.96) | 19997.76 (12471.78-27999.14) |
| New Zealand | 17313.43 (10783.74-24544.11) | 16731.36 (10391.94-23633.97) | 15712.31 (9666.74-22387.96) | 14553.49 (8750.64-21196.67) | 13595.85 (7862.48-20151.16) | 13162.7 (7479.1-19736.65) | 13099.34 (7447.6-19616.05) | 13045.53 (7415.39-19454.64) | 13019.4 (7426.21-19457.61) | 13032.29 (7459.03-19442.34) | 13065.83 (7491.22-19657.72) |
| Ireland | 20244.68 (13215.69-29083.88) | 20383 (13371.45-29101.91) | 20686.93 (13549.4-29509.67) | 21060.14 (13780.01-30006.46) | 21397.68 (13915.27-30236.2) | 21572.25 (13704.32-30645.28) | 21580.68 (13737.59-30527.33) | 21522.68 (13702.92-30329.77) | 21460.88 (13621.31-30156.95) | 21473.16 (13554.28-30176.54) | 21558.04 (13560.13-30319.51) |
| Bhutan | 26132.93 (19698.87-32841) | 25674.48 (19367.92-32384) | 24735.01 (18495.2-31545.01) | 23645.55 (17122.69-31160.07) | 22722.13 (15634.19-30781.48) | 22280.01 (14409.7-31338.21) | 22176.22 (14418.98-31026.8) | 22062.52 (14397.7-30732.54) | 21951.45 (14295.78-30325.89) | 21855.85 (14251.61-30031.22) | 21776.77 (14223.06-29920.04) |
| Switzerland | 11705.66 (8062.38-16524.05) | 11681.06 (8114.74-16401.17) | 11688.81 (8230.93-16271.82) | 11694.69 (8285.31-16197.61) | 11693.6 (8303.94-16207.1) | 11681.08 (8309.31-16147.88) | 11671.1 (8297.68-16122.04) | 11659.66 (8300.08-16009.44) | 11642.64 (8316.61-15964.12) | 11621.3 (8300.32-15905.46) | 11601.61 (8238.81-15925.85) |
| Russian Federation | 31219.78 (22337.64-40636.93) | 31120.93 (22340.7-40470.78) | 31151.59 (22465.91-40450.18) | 31310.33 (22686.89-40575.13) | 31577.54 (22945.54-40834.91) | 31801.29 (23092.42-41067.89) | 31852.69 (23116.11-41155.96) | 31734.17 (22991.53-41003.81) | 31541.94 (22811.53-40777.19) | 31439.69 (22711.71-40655) | 31480.33 (22716.56-40694.66) |
| Kyrgyzstan | 31862.49 (22735.33-40961.65) | 31760.97 (22707.71-40783.19) | 31646.15 (22678.81-40757.13) | 31508.77 (22711.03-40729.82) | 31337.42 (22588.44-40704.96) | 31175.07 (22495.51-40623.51) | 31039.84 (22416.99-40452.49) | 30934.5 (22372.93-40163.16) | 30829.09 (22364.57-39926.02) | 30743.64 (22367.56-39750.6) | 30709.35 (22254.96-39903.84) |
| Ukraine | 32315.12 (23417.64-41978.69) | 32132.66 (23292.57-41637.23) | 31885.53 (23077.18-41203.73) | 31627.66 (22910.75-40766.89) | 31442.05 (22754.94-40533.22) | 31348.3 (22618.88-40439.26) | 31289.91 (22665.13-40599.03) | 31232.39 (22694.9-40629.96) | 31211.81 (22687.03-40648.57) | 31289.48 (22718.82-40737.1) | 31448.05 (22842.82-40972.85) |
| Brunei Darussalam | 26882.41 (18631.76-35893.85) | 26926.78 (18641.77-35813.4) | 26969.04 (18679.6-35681.77) | 26990.92 (18699.52-35609.44) | 27005.19 (18663.31-35611.3) | 26995.41 (18696.4-35494.46) | 26974.42 (18684.52-35542.65) | 26938.13 (18685.69-35509.87) | 26877.59 (18725.71-35530.56) | 26807.89 (18669.47-35523.19) | 26725.34 (18554.64-35451.7) |
| Latvia | 28465.23 (21175.55-36575.29) | 28405.29 (21130.46-36375.42) | 28610.38 (20943.41-37151.4) | 28913.46 (20676.92-37618.59) | 29144.32 (20169.88-38220.58) | 29188.88 (19591.25-38589.54) | 29048.71 (19458.61-38386.83) | 28886.09 (19453.46-38273.32) | 28833.24 (19544.58-38408.14) | 28952.13 (19712.23-38844.7) | 29170.51 (19836.24-39247.8) |
| Tajikistan | 31582.83 (23035.66-41005.53) | 31575.21 (23060.97-40827.77) | 31606.19 (23058.24-40995.73) | 31585.32 (23016.98-40997.31) | 31521.11 (22943.75-40848.36) | 31406.05 (22818.47-40789) | 31299.4 (22695.01-40652.49) | 31167.91 (22562.58-40375.91) | 31025.46 (22382.47-40091.9) | 30911.46 (22280.12-40041.76) | 30859.07 (22188.73-40147.74) |
| Germany | 21731.77 (17799.1-26508.57) | 21188.14 (17415.93-25561.38) | 19883.29 (16213.3-24069.33) | 18312.96 (14689.91-22471.85) | 16978.54 (13387.45-21216.33) | 16383.81 (12733.14-20845.17) | 16350.35 (12705.17-20767.31) | 16322.52 (12683.86-20717.05) | 16297.6 (12684.89-20736.58) | 16282.33 (12669.54-20701.55) | 16260.99 (12661.84-20677.14) |
| Equatorial Guinea | 25416.78 (17115.85-33968.52) | 24629.73 (17785.7-31528.78) | 23060.29 (16964.48-29300.81) | 21250.4 (15591.48-27217.16) | 19739.61 (13584.37-26453.21) | 19071.35 (11880.84-27349.63) | 19018.32 (11908.98-27255.67) | 18987.32 (11979.38-27228.47) | 18977.06 (11996.87-27196.23) | 18981.79 (12014.21-27242.43) | 18995.79 (11984.11-27421.16) |
| Andorra | 19867.96 (12567.66-27958) | 20099.06 (12706.72-28302.41) | 20057.77 (12617.8-28282.09) | 19965.9 (12507.93-28183.39) | 19858.46 (12366.65-28080.47) | 19880.88 (12308.96-28104.1) | 19930.6 (12354.6-28310.56) | 20006.38 (12465.1-28364.24) | 19992.02 (12488.97-28418.14) | 19958.76 (12517.62-28367.36) | 20069.41 (12600.64-28591.79) |
| Honduras | 25905.1 (17283.9-35106.45) | 25905.52 (17281.91-35024.03) | 25910.28 (17321.23-34998.16) | 25912.26 (17220.31-34971.55) | 25902.94 (17279.26-35085.72) | 25879.31 (17350.01-34985.74) | 25848.47 (17258.08-34894.79) | 25814.63 (17212.71-34946.76) | 25786.17 (17076-34917.25) | 25764.75 (17040.59-34870.41) | 25750.91 (17013.32-34815.09) |
| Mongolia | 31343.31 (22463.48-40644.29) | 31279.7 (22458.97-40548.21) | 31267.59 (22444.98-40374.52) | 31239.66 (22406.5-40273.19) | 31156.99 (22391.71-40159.78) | 31000.85 (22336.34-39955.19) | 30788.8 (22043.55-39688.19) | 30543.52 (21797.32-39291.81) | 30322.33 (21681.64-38901.21) | 30199.57 (21732.66-38764.42) | 30199.01 (21750.41-38813.92) |
| Chile | 28301.89 (21216.23-36533.47) | 28232.45 (21148.03-36459.82) | 28168.05 (20900.03-36321.09) | 28115.52 (20582.2-36256.95) | 28048.95 (20274.96-36193.53) | 27980.23 (20052.03-36101.02) | 27887.61 (20002.41-35961.89) | 27746.19 (19960.92-35679.08) | 27597.12 (19883.37-35541.5) | 27509.49 (19857.04-35406.47) | 27529.68 (19898.35-35543.08) |
| Austria | 19964.33 (12444.63-28321.8) | 19924.84 (12462.44-28249.97) | 19863.43 (12444.2-28174.27) | 19806.51 (12389.61-28080.51) | 19777.8 (12289.91-27946.82) | 19777.84 (12227.61-27928.28) | 19808.29 (12374.8-27921.8) | 19836.61 (12439.69-27958.28) | 19843.36 (12494.45-27968.87) | 19831.08 (12478.18-27965.62) | 19806.83 (12457.14-28017.39) |
| Belarus | 32139.01 (22623.49-42418.94) | 32078.1 (22661.65-42210.29) | 32092.76 (22720.7-42154.55) | 32072.18 (22805.37-42016.97) | 31964.29 (22793.34-41784.27) | 31785.54 (22672.45-41595.43) | 31531.55 (22515.9-41225.88) | 31270.79 (22356.74-40909.97) | 31122.59 (22143.39-40727.42) | 31137.37 (22080.45-40725.25) | 31259.97 (22142.9-40890) |
| Kazakhstan | 31552.7 (22779.75-41043.01) | 31338.98 (22524.15-40867.01) | 31086.5 (22317.16-40518.98) | 30797.17 (22079.08-40164.16) | 30537.95 (21824.69-40064.67) | 30374.82 (21693.07-39841.64) | 30261.46 (21561.61-39624.12) | 30136.18 (21526.15-39256.28) | 30031.94 (21476.31-39019.07) | 30028.29 (21385.97-38881.37) | 30143.61 (21375.78-39007.17) |
| Slovakia | 31477.95 (22528.03-40970.32) | 31552.52 (22658.67-40838.61) | 31562.18 (22648.16-40875.44) | 31415.4 (22604.4-40650.24) | 31182.85 (22463.81-40503.97) | 31019.39 (22382.74-40496.74) | 30966.12 (22290.85-40237.2) | 30897.33 (22182.31-40032.41) | 30707.04 (22023-39724.5) | 30491.82 (21826.94-39657.39) | 30454.69 (21812.96-39634.82) |
| Rwanda | 24132.89 (17941-30698.03) | 23756.17 (17804.5-30189.91) | 22880.25 (16905-29381.02) | 21933.51 (15726.83-28883.9) | 21199.28 (14423.29-28799.29) | 20918.75 (13506.21-29267.36) | 20955.51 (13522.84-29341.78) | 20992.09 (13524.93-29455.56) | 21030.26 (13504.55-29608.01) | 21069.96 (13549.06-29627.31) | 21094.11 (13582.43-29749.21) |
| Iceland | 19936.28 (12413.77-28318.18) | 19907.84 (12452.82-28256.81) | 19845.06 (12440.03-28137.37) | 19829.73 (12519.52-28147.4) | 19773.56 (12513.81-28169.47) | 19708.17 (12510.3-28148.03) | 19628.14 (12446.17-27913.17) | 19578.86 (12419.34-27617.54) | 19537.77 (12346.06-27584.74) | 19504.94 (12239.26-27500.18) | 19503.83 (12286-27576.38) |
| Spain | 18708.41 (13244.04-26013.69) | 18946.72 (13448-26412.47) | 19451.27 (13811.17-27100.03) | 20035.48 (14190.2-27783.96) | 20520.75 (14518.01-28461.75) | 20724.05 (14639.23-28795.8) | 20660.33 (14599.77-28667.04) | 20495.7 (14433.41-28440.83) | 20293.18 (14252.02-27917.81) | 20122.35 (14043.83-27557.55) | 20050.27 (13875.21-27487.73) |
| Eritrea | 27067.94 (18171.12-36647.32) | 26727.52 (18777.91-35522.88) | 25978.87 (18867.61-34072.27) | 25087.46 (18609.91-32161.36) | 24325.63 (18129.38-30766.07) | 23990.33 (18037.21-30593.89) | 23661.27 (17727.89-30092.3) | 22896.23 (17054.87-29829.24) | 21974.07 (15661.4-28993.36) | 21190.76 (14387.79-28924.74) | 20838.69 (13165.78-29848.22) |
| Djibouti | 26504.51 (18093.15-35538.42) | 25867.23 (19033.36-33414.17) | 24390.87 (18293.83-30933.46) | 22635.59 (16913.3-29052.96) | 21167.05 (15079.69-28762.65) | 20548.32 (13043.04-29965.49) | 20534.07 (12968.56-29540.99) | 20506.65 (13044.84-29194.61) | 20481.24 (13032.13-29112.83) | 20477.15 (13108.22-29161.31) | 20490.7 (13073.56-29372.13) |
| Democratic Republic of the Congo | 26271.63 (17826.63-35096.16) | 25959.29 (18405.63-34021.01) | 25246.56 (18391.22-32573.93) | 24403.28 (18073.23-31246.58) | 23695.56 (17679.9-30079.28) | 23390.6 (17577.81-29606.5) | 23052.45 (17223.02-29236.45) | 22266.66 (16469.89-28542.9) | 21334.52 (15346.05-27702.87) | 20557.85 (13913.15-27685.62) | 20240.19 (12964.62-28301.08) |
| Poland | 32566.52 (24528.35-41335.55) | 32495.63 (24440.03-41219.31) | 32437.07 (24332.86-41242.33) | 32342.5 (24176.12-41236.02) | 32164.94 (23941.53-41122.66) | 31933.56 (23709.31-40901.75) | 31693.18 (23501.36-40623.14) | 31463.6 (23336.58-40413.8) | 31288.79 (23216.09-40244.16) | 31253.05 (23210.38-40205.5) | 31397.4 (23335.66-40382.3) |
| Burundi | 26969.94 (18437.16-36098.56) | 26273.56 (19252.92-33708.54) | 24789.91 (18764.84-31184.69) | 23037.54 (17204.71-29133.39) | 21568.51 (15420.1-28515.88) | 20935.7 (13736.59-29463.93) | 20924.58 (13627.44-29358.89) | 20940.3 (13571.89-29334.93) | 20966.46 (13547.33-29596.58) | 20988.69 (13520.48-30005.24) | 21009.13 (13524.5-30527.8) |
| Mozambique | 27241.43 (18309.4-36842.49) | 27213.54 (18326.33-36758.32) | 27176.15 (18317.88-36539.35) | 27135.26 (18320.8-36429.65) | 27106.85 (18320.57-36401.79) | 27105.83 (18276.3-36490.11) | 27120.21 (18340-36269.67) | 27128.4 (18325.97-36068.36) | 27134.31 (18322.29-35987.77) | 27142.2 (18326.02-35972.75) | 27155.17 (18315.61-35948.7) |
| Lesotho | 24227.75 (16038.19-33563.89) | 24246.29 (16106.63-33519.66) | 24270.53 (16166.55-33468.34) | 24295.88 (16169.95-33296.41) | 24314.81 (16053.74-33249.92) | 24316.33 (15932.27-33323.63) | 23870.25 (16844.39-31373.56) | 22822.2 (16554-29361.94) | 21570.81 (15494.78-28266.52) | 20504.88 (14013.52-28258.49) | 20021.73 (12295.98-29544.22) |
| Pakistan | 28290.8 (20444.92-36463.51) | 27933.96 (20608.89-35656.65) | 27419.79 (20551.56-34611.08) | 26883.67 (20407.21-33721.2) | 26461.36 (20245.3-33074.72) | 26275.67 (20192.46-32741.02) | 26023.8 (20288.41-32161.18) | 25461.86 (19987.25-31151.61) | 24798.34 (19625.49-30266.85) | 24248.73 (19238.59-29595.66) | 24024.85 (19123.05-29353.57) |
| Trinidad and Tobago | 25669.91 (17343.31-34304.76) | 24974.97 (16920.48-33410.72) | 23458.14 (15672.09-31641.94) | 21652.75 (14268.63-29911.18) | 20094.34 (12911.44-28547.94) | 19317.11 (12131.85-28068.95) | 19131.26 (11993.12-27878.99) | 18990.88 (11886.82-27912.22) | 18897.49 (11754.71-27872.55) | 18844.39 (11641.01-27950.05) | 18817.43 (11588.48-28038.76) |
| Ecuador | 33230.09 (23943.67-42280.15) | 32970.56 (24799.58-40725.87) | 32272.67 (25535.81-38610) | 31339.43 (25903.89-36386.72) | 30398.88 (25956.68-34810.88) | 29712.66 (25883.26-33811.09) | 29286.22 (25787.53-33112.29) | 28949.73 (25597.62-32644.02) | 28716.49 (25344.32-32450.88) | 28572.28 (25105.32-32409.17) | 28480.08 (24868.35-32374.78) |
| Panama | 25460.28 (16906.35-34792.72) | 25545.88 (16990.98-34614.37) | 25635.04 (17081.53-34501.45) | 25685.76 (17101.74-34537.75) | 25669.87 (16978.63-34474.31) | 25584.17 (16832.75-34382.33) | 25159.73 (17822.97-32501.92) | 24328.09 (17904.42-30760.27) | 23382.4 (17135.96-29705.97) | 22592.98 (16057.57-29734.42) | 22213.6 (14147.21-31205.01) |
| Kuwait | 23581.16 (17373-30670.92) | 23694.37 (17586.65-30757.31) | 23965.29 (17714.9-31144.16) | 24310.82 (17600.48-31595.95) | 24624.93 (17601.48-32241) | 24760.3 (17521.91-32734.87) | 24756.97 (17522.26-32695.06) | 24767.66 (17497.7-32746.94) | 24779.27 (17484.46-32676.74) | 24790.32 (17505.49-32716.77) | 24808.37 (17509.4-32709.41) |
| Nicaragua | 26031.95 (17278.72-35176.28) | 25851.37 (17959.22-34137.23) | 25466.15 (18309.05-33219.28) | 25012.86 (18419.01-32224.38) | 24617.4 (18387.3-31422.83) | 24407.93 (18213.85-31321.1) | 24187.28 (17988.9-31102.91) | 23763.33 (17631.89-30985.9) | 23278.67 (16619.93-31101.93) | 22873.07 (15611.45-31415.29) | 22686.25 (14709.69-32124.83) |
| Oman | 31426.67 (22334.39-40308.82) | 30878.58 (22307.44-39756.07) | 29735.66 (21308.98-38420.67) | 28401.58 (19901.69-37388.99) | 27265.14 (18700.18-36524.36) | 26711.45 (17774.39-36327.49) | 26606.59 (17763.49-36116.83) | 26552.52 (17766.17-36094) | 26542.37 (17742.53-35960.75) | 26554.81 (17761.37-35921.62) | 26565.68 (17720.87-35918.38) |
| Argentina | 28543.5 (19997.78-36667.94) | 28529.95 (20065.49-36658.33) | 28390.07 (19981.39-36471.43) | 28157.59 (19797.18-36178.99) | 27934.15 (19584.01-35869.57) | 27858.56 (19584.18-35847.88) | 27911.59 (19606.97-35950.36) | 27925.74 (19725.03-36027.22) | 27843.44 (19601.7-35964.08) | 27740.25 (19471.98-35867.64) | 27697.53 (19405.14-35836.71) |
| Venezuela (Bolivarian Republic of) | 25425.66 (17009.58-34766.44) | 25231.54 (17508.71-33891.78) | 24786.51 (17604.7-32589.18) | 24186.23 (17683.25-31490.43) | 23603.33 (17380.76-30284.44) | 23298.78 (17215.87-29733.9) | 23161.11 (17133.11-29577) | 22945.64 (16841.78-29767.19) | 22652.85 (16082.6-30087.03) | 22333.19 (14985.6-30624.15) | 22123.84 (14071.59-31306.94) |
| France | 12403.67 (7975.83-18156.51) | 12272.47 (7861.51-18075.35) | 12201.35 (7797.24-18011.71) | 12159.87 (7770.93-17956.03) | 12120.28 (7742.75-18009.72) | 12069.34 (7617.72-18062.28) | 12016.6 (7584.1-18001.3) | 11983.82 (7531.95-18030.5) | 11967.36 (7442.04-18078.42) | 11962.67 (7374.84-18068.93) | 11967.1 (7323-18185.06) |
| Japan | 24935.99 (18834.7-31598) | 24622.26 (18594.33-31277.9) | 24112.14 (18154.65-30761) | 23534.45 (17639.72-30189.36) | 23007.52 (17141.02-29699.43) | 22637.17 (16788.09-29289.46) | 22355.16 (16592.51-28980.03) | 22030.49 (16320.75-28665.88) | 21737.23 (16046.81-28369.9) | 21505.63 (15840.83-28170.85) | 21335.53 (15698-28015.35) |
| Mexico | 25240.85 (16956.92-34062.2) | 25248.44 (16965.52-34061.14) | 25248.19 (16970.96-34103.37) | 25259.26 (16982.59-34159.54) | 25282.01 (17005.72-34195.69) | 25295.28 (17031.64-34210.31) | 25449.41 (17213.59-34376.64) | 25819.02 (17601.86-34655.34) | 26279 (18034.04-34959.65) | 26688.02 (18425.9-35318.86) | 26872.66 (18700.45-35577.84) |
| Singapore | 21318.35 (15559.53-27695.08) | 20976.99 (15413.27-27207.24) | 20617.05 (15073.78-26694.45) | 20255.35 (14712.22-26342.74) | 19890.86 (14377.56-26047.1) | 19626.8 (14138.34-25823.28) | 19479.11 (14021.55-25594.97) | 19338.82 (13887.38-25347.19) | 19186.09 (13733.63-25131.7) | 19039.25 (13560.73-24946.88) | 18897.29 (13453.67-24685.42) |
| Haiti | 29057.67 (20391.21-38072.8) | 29012.39 (20292.84-38044.02) | 28954.51 (20234.79-37998.79) | 28901.91 (20240.31-37900.4) | 28867.34 (20185.87-37913.5) | 28858.84 (20183.74-37993.07) | 28883.68 (20198.58-37952.65) | 28937.96 (20225.55-37983.79) | 29006.58 (20316.99-37982.46) | 29071.79 (20410.25-38083.16) | 29105.57 (20441.81-38089.4) |
| Israel | 22973.02 (14949.84-32376.12) | 23513.95 (15635.91-32541.88) | 24650.64 (16965.9-33143.48) | 25966.3 (18313.64-34045.21) | 27093.34 (19109.41-35258.26) | 27624.88 (19263.3-35872.87) | 27694.91 (19346.97-35990.6) | 27713.96 (19478.9-36200.86) | 27684.75 (19466.2-36140.76) | 27649.42 (19446.12-36142.56) | 27634.12 (19426.35-36049.6) |
| Qatar | 27505.98 (18573.98-36964.43) | 27790.96 (19202.92-36783.49) | 28439.06 (20353.74-37020.93) | 29188.94 (21354-37529.15) | 29782.31 (22014.97-38185.72) | 29968.41 (21966.59-38603.42) | 29834.65 (21966.65-38360.01) | 29652.59 (21869.72-37930.02) | 29464.78 (21769.59-37679.37) | 29314.79 (21707.8-37380.97) | 29243.77 (21647.49-37285.89) |
| Republic of Korea | 38969.45 (30224.22-48235.5) | 38797 (30402.98-47366.75) | 38206.22 (30473.18-46259.72) | 37324.56 (30081.11-45030.2) | 36406.85 (29204.66-44079.91) | 35787.84 (28480.11-43643.24) | 35478.68 (28260.64-43216.54) | 35185.87 (28043.13-42819.17) | 34982.47 (27914.77-42693.87) | 34923.99 (27861.15-42738.35) | 34917.65 (27839.63-42740.05) |
| Italy | 17945.53 (13079.16-23323.59) | 17752.27 (12896.63-23098.17) | 17490.74 (12644.96-22823.11) | 17214.73 (12384.24-22546.54) | 16975.28 (12181.79-22230.23) | 16856.46 (12068.49-22044.58) | 16833.26 (12057.2-22026.37) | 16827.83 (12058.18-22053.65) | 16819 (12056.75-22125.32) | 16816.67 (12052.75-22131.77) | 16819.28 (12052.37-22129.76) |
| Morocco | 26772.44 (17985.63-36524.5) | 26628.09 (18782.42-35431.93) | 26358.01 (19129.89-34426.49) | 26042.62 (19399.46-33333.54) | 25762.27 (19155.57-32592.79) | 25598.94 (19129.32-32297.01) | 25430.4 (19082.35-32075.13) | 25140.67 (18744.77-32220.07) | 24823.77 (17960.06-32383.66) | 24574.77 (17102.44-32881.42) | 24488.7 (16353.61-33813.11) |
| Lithuania | 45680.13 (38566.7-52893.38) | 45577.46 (38591.4-52656.06) | 45389.12 (38512.14-52334.28) | 45055.77 (38340.73-51900.94) | 44607.42 (38093.53-51363.32) | 44132.06 (37692.36-50805.1) | 43644.81 (37339.35-50209.79) | 43135.16 (36871.49-49544.39) | 42695.84 (36446.38-49015.42) | 42467.73 (36184.62-48791.94) | 42526.73 (36226.58-48840.15) |
| United Arab Emirates | 25579.47 (19366.32-32057.65) | 25675.7 (19999.93-31673.96) | 26036.62 (20569.64-31919.64) | 26562.56 (21016.35-32574.94) | 27173.2 (21530.06-33422.27) | 27772.61 (21244.77-34445.82) | 28351.31 (22288.08-34656.23) | 28801.96 (23218.16-34733.03) | 28995.12 (23819.05-34781) | 29027.57 (24073.7-34564.05) | 29082.33 (24121.44-34562.59) |
| Algeria | 27009.25 (18071.07-36929.86) | 26854.7 (18901.04-35684.58) | 26526.85 (19192.22-34776.71) | 26116.86 (19346.18-33662.07) | 25731.61 (19190.16-32709.47) | 25488.11 (19101.68-32242.99) | 25275.43 (18826.13-31667.93) | 24964.8 (18563.41-31809.41) | 24642.09 (17795.69-31834.05) | 24389.61 (16903-32333.29) | 24290 (15893.05-33419.16) |
| Egypt | 26724.69 (17692.03-36767.77) | 26709.97 (17780.79-36469.5) | 26681.75 (17875.69-36350.36) | 26632.86 (17953.35-36016.05) | 26563.31 (18004.93-35889.05) | 26487.21 (17884-35748.74) | 26218.98 (19042.28-34103.67) | 25666.57 (19159.53-32643.29) | 24990.34 (18917.61-31557.58) | 24379.58 (17830.2-31747.74) | 24044.27 (15922.37-32903.62) |
| Iraq | 26799.6 (18030.6-36049.16) | 26801.08 (18107.15-36085) | 26819.77 (18155.09-36059.06) | 26846.02 (18219.9-36319.53) | 26869.18 (18206.56-36654.3) | 26873.15 (18150.82-36903.46) | 26675.05 (19627.52-34752.89) | 26225.39 (19862.43-33044.45) | 25645.08 (19370.32-31981.19) | 25087.79 (18162.42-32040.18) | 24832.95 (16480.02-33353.33) |
| Portugal | 19704.44 (13257.92-27436.04) | 19652.34 (13233.78-27342.43) | 19543.08 (13244.69-27113) | 19392.94 (13197.52-26905.3) | 19234.06 (13211.02-26391.37) | 19134.97 (13104.79-26487.15) | 19084.13 (13129.59-26229.67) | 19050.77 (13116.73-26328.93) | 19016.9 (13082.02-26435.65) | 18992.09 (13028.63-26483.11) | 18994.57 (12930.15-26545.59) |
| Afghanistan | 27602.63 (18535.67-37281.53) | 27457.31 (20102.54-35442.46) | 26886.19 (20364.78-33800.14) | 26108.89 (19887.59-32966.08) | 25533.55 (18871.73-33297.6) | 25396.54 (17237.54-35017.83) | 25480.8 (17263.71-34908.23) | 25540.55 (17278.88-34956.37) | 25594.95 (17261.36-35028.97) | 25605.22 (17170.6-34907.92) | 25581.62 (17040.43-34758.93) |
| Paraguay | 31557.85 (22359.98-42226.17) | 31626.22 (22283.65-42258.84) | 31668.17 (22240.63-42352.41) | 31687.75 (22200.93-42335.63) | 31689.26 (22147.54-42407.38) | 31676.28 (22163.8-42351.14) | 31572.71 (22169.98-41748.22) | 31349.95 (22294.68-41129.39) | 31084.01 (22249.5-40619.25) | 30851.64 (22313.54-40200.24) | 30726.44 (22385.69-39988.01) |
| Serbia | 37999.43 (29412.35-47264.85) | 37521.12 (29075.87-46665.2) | 36706.24 (28576.71-45516.57) | 35886.73 (28021.22-44312.65) | 35220.35 (27503.01-43653.49) | 34879.91 (27130.88-43297.42) | 34772.3 (27169.54-43073.19) | 34629.64 (27153.74-42766.21) | 34449.87 (27078.73-42638.71) | 34347.89 (26860.91-42642.77) | 34418.04 (26855.4-42764.27) |
| Romania | 46642.58 (38825.84-54822.81) | 45723.72 (38070.74-53651.53) | 43445.49 (35723.62-51315.53) | 40661.01 (32659.85-48848.21) | 38285.11 (29922.64-47069.86) | 37162.93 (28546.16-46498.85) | 36937.48 (28248.8-46183.82) | 36756.92 (27978.34-45998.37) | 36685.18 (27887.35-45765.67) | 36759.35 (27963.2-45846.38) | 36941.79 (28093.82-46087.21) |
| Libya | 26193.72 (17478.93-35586.97) | 26209.95 (17450.26-35659.19) | 26267.81 (17574.86-35884.53) | 26350.94 (17594.4-35827.36) | 26437.57 (17595.75-35939.72) | 26495.22 (17660.26-36057.3) | 26303.31 (19069.28-34387.08) | 25800.53 (19274.09-32987.07) | 25202.56 (18933.24-31897.02) | 24715.17 (18015.84-32386.33) | 24542.29 (16235.16-34063.45) |
| Brazil | 23174.48 (15882.48-31218.94) | 23190.55 (15887.88-31175.5) | 23245.75 (15965.44-31131.94) | 23304.9 (16078.81-31066.35) | 23342.74 (16131.41-31003.86) | 23336.8 (16169.45-30965.58) | 23277.13 (16133.08-30954.4) | 23186.14 (16055.84-30905.66) | 23091.88 (15978.13-30836.33) | 23015.93 (15903.63-30776.99) | 22979.6 (15849.86-30737.98) |
| Jordan | 25500.13 (19469.9-34230.81) | 25456.84 (19527.85-34112.77) | 25402.27 (19488.6-33883.47) | 25345.73 (19455.64-33715.28) | 25295.23 (19331.22-33621.08) | 25261.47 (19192.87-33423.81) | 25184.28 (19257.46-33085.2) | 25048.58 (19172.81-33022.44) | 24974.75 (19100.03-32957.09) | 24973.85 (18957.25-32829.63) | 24990.69 (18807.53-32733.61) |
| India | 29956.92 (21617.43-38584.49) | 29968.76 (21594.55-38676.33) | 29918.03 (21476.05-38691.52) | 29830.13 (21313.32-38559.59) | 29738.72 (21111.85-38504.66) | 29678.61 (21000.54-38505.7) | 29631.33 (20950.23-38460.29) | 29565.22 (20911.72-38404.62) | 29486.24 (20851.17-38333.3) | 29407.08 (20773.53-38288.85) | 29350.21 (20700.99-38257.97) |
| Saint Lucia | 29435.39 (20923.36-38052.68) | 29378.88 (20843.76-37881.64) | 29299.12 (20683.41-38004.17) | 29223.07 (20555.83-38134.26) | 29166.28 (20446.46-38346.32) | 29141.23 (20367.83-38539.45) | 29131.31 (20414.89-38483.84) | 29107.95 (20354.4-38255.42) | 29073.22 (20247.49-38039.93) | 29037.52 (20237.95-37944.2) | 29008.02 (20227.95-37904.46) |
| Bahamas | 27235.32 (18940.11-35626.13) | 26968.43 (18809.22-35269.19) | 26863.24 (18761.67-35204.89) | 26924.15 (18761.9-35417.95) | 27063.72 (18812.63-35753.54) | 27151.88 (18850.14-35969.9) | 27118.4 (18819.12-35783.21) | 27007.87 (18780.94-35484.52) | 26924.79 (18729-35326.68) | 26935.35 (18761.97-35436.89) | 27034.63 (18836.25-35564.48) |
| Palestine | 23386.66 (16515.37-31249.36) | 23440.81 (16656.6-31066.01) | 23490.6 (16773.15-30906.24) | 23537.55 (16842.06-30826.68) | 23578.03 (16909.75-30750.97) | 23609 (16857.29-30835.45) | 23631.22 (16903.99-30850.06) | 23651.87 (16877.39-30935.22) | 23671.3 (16895.36-31037.15) | 23692.06 (16953.78-31116.97) | 23729.94 (16983.69-31333.84) |
| Peru | 27906.01 (19275.88-36561.54) | 27867.79 (19243.48-36526.56) | 27811.75 (19207.89-36352.27) | 27750.89 (19240.83-36353.57) | 27692.64 (19184.01-36400.63) | 27640.22 (19048.38-36320.26) | 27586.41 (19121.94-36153.96) | 27525.5 (19162.56-35954.93) | 27473.52 (19122.44-35853.06) | 27432.18 (19131.22-35784.38) | 27397.8 (19106.02-35775.12) |
| Malta | 20327.51 (13011.15-28964.68) | 20355.08 (12953.47-28686.63) | 20370.36 (12920.46-28691.15) | 20341.5 (12907.87-28548.4) | 20292.93 (12781.34-28509.35) | 20234.32 (12669.56-28573.16) | 20152.35 (12644.44-28417.81) | 20040.22 (12581.07-28337.28) | 19904.39 (12555.7-28148.72) | 19794.86 (12520.76-28006.73) | 19755.4 (12515.19-27944.57) |
| Niue | 31388.43 (22219.84-40973.64) | 31407.31 (22219.2-40989.02) | 31366.13 (22201.17-40703.98) | 31289.65 (22154.16-40644.36) | 31265.1 (22194.67-40726.31) | 31306.76 (22197.8-40866.84) | 31383.25 (22256.21-40842.04) | 31452.38 (22338.27-40808.62) | 31494.27 (22344.76-40810.47) | 31495.29 (22261.91-40812.86) | 31449.1 (22152.03-40915.72) |
| Bolivia (Plurinational State of) | 28133.94 (19583.76-37125.93) | 28093.24 (19507.47-36834.9) | 28049.95 (19440.28-36716.05) | 28010.96 (19411.56-36895.06) | 27976.11 (19372.69-37096.6) | 27941.22 (19304.5-37235.7) | 27895.57 (19353.49-37076.68) | 27834.45 (19439.71-36799.97) | 27767.49 (19323.53-36702.3) | 27704.6 (19207.24-36813.92) | 27660.2 (19109.21-36970.33) |
| Ghana | 25062.4 (16578.19-34228.57) | 25062.8 (16651.29-34242.86) | 25046.63 (16629.37-34243.49) | 25014.11 (16606.82-34265.73) | 24971.74 (16531.64-34101.1) | 24931.85 (16443.88-34083.12) | 24887.48 (16469.41-33915.47) | 24829.89 (16554.82-34036.05) | 24769.47 (16558.76-34050.42) | 24717.73 (16490.81-34139.35) | 24685.4 (16404.13-34179.34) |
| Grenada | 28776.81 (19831.13-37660.92) | 28773.87 (19890.94-37633.88) | 28778.44 (20028.06-37602.47) | 28768.77 (20104.45-37670.84) | 28735.21 (20078.31-37703.46) | 28696.98 (20025.3-37847.55) | 28671.29 (20148.09-37686.08) | 28664.16 (20159.65-37693.29) | 28672.12 (20112.3-37857.56) | 28692.81 (20242.6-38089.24) | 28720.78 (20336.26-38265.05) |
| Zambia | 26823.51 (18103.94-35734.61) | 26850.5 (18178.8-35770.57) | 26862.94 (18168.9-35815.85) | 26856.24 (18156-35966.41) | 26847.53 (18116.73-36018.61) | 26839.07 (18048.88-36075.67) | 26817.41 (18112.81-36000.41) | 26777.43 (18111.7-35809.66) | 26728.24 (18034.47-35752.24) | 26683.16 (17926.12-35766.95) | 26656.48 (17917.07-35971.46) |
| Antigua and Barbuda | 28690.82 (19872.65-37682.65) | 28694.4 (19912.76-37524.26) | 28712.21 (20114.26-37538.92) | 28734.38 (20170.09-37521.42) | 28747.75 (20165.55-37619.57) | 28745.83 (20038.85-37704.96) | 28720 (20039.38-37660.86) | 28672.75 (19992.17-37527.35) | 28624.05 (20004.87-37528.54) | 28609.87 (19973.58-37398) | 28640.64 (19892.48-37495.24) |
| Seychelles | 30306.47 (21009.11-39777.79) | 30223.77 (20953.55-39752.02) | 30142.64 (20950.48-39670.65) | 30071.21 (20839.15-39574.15) | 30012.18 (20695.73-39567.38) | 29961.33 (20648.12-39681.21) | 29912.67 (20641.09-39360.66) | 29864.69 (20630.01-39079.19) | 29836.37 (20699.71-39114.17) | 29846.06 (20777.42-39141.7) | 29880.61 (20793.67-39327.93) |
| Dominica | 29309.09 (20229.69-38151.98) | 29259.7 (20333.64-38081.38) | 29259.93 (20409.8-38180.84) | 29284.85 (20400.35-38223.25) | 29307.94 (20362.55-38349.32) | 29330.36 (20386.12-38533.6) | 29339.52 (20433.1-38383.59) | 29309.97 (20403.05-38212.06) | 29231.92 (20466.22-38186.97) | 29119.4 (20544.94-38162.43) | 29024.13 (20448.19-38043.03) |
| Bahrain | 26599.41 (17814.89-36071.58) | 26629.59 (17888.02-36098.87) | 26583.03 (17809.26-36022.43) | 26498.63 (17623.79-35965.89) | 26373.19 (17443.8-35904.91) | 26267.07 (17373.81-35893.59) | 26189.08 (17358.82-35505.76) | 26120.68 (17416.68-35215.87) | 25993.36 (17411.02-35172.38) | 25832.3 (17334.08-34981.53) | 25719.21 (17257.24-34865.76) |
| Turkey | 29659.46 (22479.74-36927.55) | 29682.28 (22480.99-36931.87) | 29686.51 (22401.71-37030.22) | 29660.91 (22333.87-37129.33) | 29601.87 (22252.16-37121.98) | 29525.66 (22108.07-37091.19) | 29438.47 (22130-36971.75) | 29337.18 (22137.54-36856.72) | 29230.67 (22105.76-36694.36) | 29125.75 (22025.16-36565.83) | 29028.99 (21944.89-36625.01) |
| Mauritania | 25072.87 (16685.07-34476.78) | 25050.61 (16702.4-34289.96) | 25009.19 (16705.31-34290.87) | 24964.12 (16714.75-34316.78) | 24927.28 (16742.8-34296.07) | 24907.04 (16690.5-34229.65) | 24871.76 (16677.5-34217.39) | 24801 (16624.51-34057.32) | 24721.33 (16511.56-33930.37) | 24657.04 (16410.37-33772.83) | 24628.95 (16343.24-33804.02) |
| El Salvador | 26008.82 (17420.93-35446.93) | 26018.47 (17423.05-35296.21) | 25971.87 (17341.02-35192.33) | 25890.51 (17290.92-35029) | 25798.76 (17120.3-34793.9) | 25714.53 (17065.66-34778.27) | 25636.73 (17125.62-34582.56) | 25553.69 (17075.5-34490.96) | 25475.24 (17072.22-34469.83) | 25420.05 (17059.81-34504.45) | 25392.59 (17056.02-34626.02) |
| Uganda | 23949.43 (17510.07-30852.82) | 23913.99 (17459.57-30681.56) | 23866.08 (17428.02-30584.44) | 23811.76 (17386.77-30634.96) | 23759.4 (17294.25-30720.35) | 23717.07 (17226.35-30709.57) | 23652.23 (17188.08-30652.29) | 23549.58 (17199.35-30419.52) | 23440.52 (17155.75-30297.09) | 23357.99 (17079.76-30183.23) | 23335.37 (17027.97-30326.01) |
| Luxembourg | 19764.76 (12450.38-27937.46) | 19753.41 (12437.07-27991.45) | 19721.49 (12413.22-27940) | 19697.08 (12308.04-27967.04) | 19659.55 (12230.34-27842.21) | 19616.1 (12134.09-27763.89) | 19594.52 (12164.6-27689.18) | 19598.46 (12228.59-27719.52) | 19605.83 (12232.54-27780.5) | 19609.72 (12273.26-27759.8) | 19606.51 (12301.54-27796.57) |
| Guatemala | 25717.06 (17087.57-34723.12) | 25671.64 (17094.39-34545.14) | 25651.38 (17157.82-34543.97) | 25675.43 (17114.86-34873.81) | 25721.66 (17191.57-35058.59) | 25766.76 (17118.45-35143.94) | 25777.05 (17150.82-35118.34) | 25746.56 (17163.85-34938.61) | 25719.3 (17135.86-34796.92) | 25712.86 (17121.61-34712.65) | 25698.57 (17075.3-34967.69) |
| Republic of Moldova | 32927.8 (23275.59-43308.98) | 32843.55 (23187.51-43157.21) | 32749.53 (23068.75-42926.25) | 32650.99 (22922.37-42810.96) | 32550.91 (22804.16-42648.68) | 32448.69 (22676.29-42618.18) | 32347.3 (22694.98-42351.97) | 32260.62 (22699.78-42229.43) | 32200.11 (22623.31-42221.76) | 32172.83 (22591.4-42238.42) | 32181.68 (22565.95-42315.17) |
| United States Virgin Islands | 28189.8 (19637.5-37058.01) | 28177.45 (19649.9-37015.69) | 28178.45 (19650.47-37055.66) | 28191.99 (19653.94-36957.15) | 28212.45 (19610.46-36925.49) | 28231.76 (19571.89-36954.87) | 28226.2 (19692.05-37087.41) | 28189.77 (19797.73-37054.62) | 28139.48 (19826.74-36933.52) | 28094.09 (19775.58-37048.44) | 28073.92 (19657.79-37039.65) |
| Belize | 28861.05 (20210.03-38129.03) | 28810.09 (20186.32-38005.15) | 28779.73 (20197.65-37797.09) | 28777.91 (20255.69-37830.88) | 28808.26 (20300.46-37960.68) | 28866.94 (20297.95-38150.56) | 28929.73 (20432.59-37987.61) | 28966.96 (20459.79-37924.92) | 28972.79 (20370.67-37870.82) | 28958.91 (20318.21-37883.6) | 28940.38 (20262.56-38002.23) |
| Costa Rica | 25709.36 (17141.03-35206.29) | 25732.46 (17172.02-35217.8) | 25764.37 (17105.13-35125.67) | 25752.35 (17107.61-35159.8) | 25678.69 (17061.12-35178.59) | 25579.88 (16873.95-35137.7) | 25477.23 (16858.11-34957.35) | 25371.74 (16851.14-34621.99) | 25282.37 (16828.23-34574.91) | 25245.12 (16824.44-34532.64) | 25262.98 (16848.17-34709.91) |
| Suriname | 37797.8 (28169.03-47595.52) | 37852.42 (28119.65-47796.38) | 37836.04 (27925.77-48115.83) | 37769.79 (27711.22-48103.46) | 37686.55 (27602.96-48200.55) | 37623.96 (27406.87-48366.97) | 37568.95 (27442.41-48252.64) | 37509.65 (27522.95-48100.84) | 37461.67 (27609.8-47973.22) | 37432.33 (27708.1-47948.99) | 37422.29 (27770.31-47930.99) |
| Uruguay | 28334.3 (19598.99-37144.56) | 28289.2 (19653.44-37072.81) | 28211.49 (19742.38-36990.34) | 28156.86 (19713.58-37142.26) | 28132.44 (19687.17-37265.35) | 28135.03 (19666.07-37362.91) | 28174.35 (19742.83-37344.06) | 28243.57 (19699.93-37385.71) | 28327.14 (19744.96-37407.7) | 28395.33 (19770.19-37616.39) | 28394.92 (19703.62-37729.2) |
| Uzbekistan | 31698.29 (22985.8-41096.37) | 31646.18 (22994.91-40932.57) | 31572.79 (22943.21-40620.93) | 31486.91 (22894.76-40601.25) | 31385.21 (22808.41-40543.24) | 31267.47 (22702.31-40482.89) | 31142.77 (22536.35-40290.78) | 31028.7 (22359.87-40168.86) | 30945 (22264.2-40158.96) | 30900.2 (22180.76-40125.62) | 30890.27 (22153.23-40290.61) |
| San Marino | 19569.52 (12274.74-27862.87) | 19557.22 (12254.98-27731.19) | 19536.99 (12261.31-27655.8) | 19517.96 (12309.88-27713.57) | 19508.48 (12287.59-27777.7) | 19517.31 (12252.4-27825.27) | 19558.3 (12268.38-27757.27) | 19625.59 (12323.79-27632.22) | 19701.42 (12413.86-27684.03) | 19770.27 (12438.11-27759.48) | 19816.13 (12484.66-27892.87) |
| Tuvalu | 31392.97 (22331.03-40639.45) | 31411.98 (22381.37-40632.13) | 31438.9 (22386.05-40650.91) | 31473.7 (22386.56-40653.51) | 31511.31 (22344.33-40818.66) | 31548.27 (22309.32-40933.36) | 31577.23 (22462.47-40956.99) | 31591.16 (22572.26-40972.07) | 31590.17 (22595.08-41081.13) | 31579.93 (22594.9-40989.21) | 31563.37 (22596.6-41185.96) |
| Guyana | 29134.07 (20395.83-38349.24) | 29171 (20450.63-38351.73) | 29208.03 (20459.31-38239.55) | 29242.3 (20481.88-38249.21) | 29258.63 (20496.19-38341.02) | 29236.62 (20484.02-38450.03) | 29170.06 (20492.52-38097.05) | 29076.24 (20397.18-37972.57) | 28977.43 (20286.93-37866.67) | 28889.82 (20239.01-37829.76) | 28825.42 (20175.93-37801.7) |
| Jamaica | 29372.1 (20558.93-38783.22) | 29393.64 (20717.97-38627.72) | 29405.44 (20800.12-38591.33) | 29400.93 (20858.97-38470.46) | 29370.72 (20848.74-38309.52) | 29314.36 (20799.91-38211.39) | 29223.6 (20685.71-38071) | 29107.62 (20501.9-38168.89) | 29002.13 (20291.45-38247.09) | 28941.87 (20153.28-38326.62) | 28940.1 (20161.49-38437.73) |
| Tunisia | 27000.6 (17865.17-36666.87) | 26951.69 (17899.82-36646.4) | 26918.46 (17863.66-36545.45) | 26892.4 (17838.19-36443.13) | 26861.01 (17866.33-36465.4) | 26815.2 (17834.62-36525.7) | 26742.62 (17900.31-36434.43) | 26650.11 (17945.19-36307.8) | 26558.68 (17859.16-36173.89) | 26487.36 (17716.12-36039.22) | 26456.22 (17593.15-36039.02) |
| Albania | 0 (0-0) | 0 (0-0) | 0 (0-0) | 0 (0-0) | 0 (0-0) | 0 (0-0) | 0 (0-0) | 0 (0-0) | 0 (0-0) | 0 (0-0) | 0 (0-0) |
| American Samoa | 0 (0-0) | 0 (0-0) | 0 (0-0) | 0 (0-0) | 0 (0-0) | 0 (0-0) | 0 (0-0) | 0 (0-0) | 0 (0-0) | 0 (0-0) | 0 (0-0) |
| Angola | 0 (0-0) | 0 (0-0) | 0 (0-0) | 0 (0-0) | 0 (0-0) | 0 (0-0) | 0 (0-0) | 0 (0-0) | 0 (0-0) | 0 (0-0) | 0 (0-0) |
| Bangladesh | 0 (0-0) | 0 (0-0) | 0 (0-0) | 0 (0-0) | 0 (0-0) | 0 (0-0) | 0 (0-0) | 0 (0-0) | 0 (0-0) | 0 (0-0) | 0 (0-0) |
| Barbados | 0 (0-0) | 0 (0-0) | 0 (0-0) | 0 (0-0) | 0 (0-0) | 0 (0-0) | 0 (0-0) | 0 (0-0) | 0 (0-0) | 0 (0-0) | 0 (0-0) |
| Belgium | 0 (0-0) | 0 (0-0) | 0 (0-0) | 0 (0-0) | 0 (0-0) | 0 (0-0) | 0 (0-0) | 0 (0-0) | 0 (0-0) | 0 (0-0) | 0 (0-0) |
| Benin | 0 (0-0) | 0 (0-0) | 0 (0-0) | 0 (0-0) | 0 (0-0) | 0 (0-0) | 0 (0-0) | 0 (0-0) | 0 (0-0) | 0 (0-0) | 0 (0-0) |
| Bermuda | 0 (0-0) | 0 (0-0) | 0 (0-0) | 0 (0-0) | 0 (0-0) | 0 (0-0) | 0 (0-0) | 0 (0-0) | 0 (0-0) | 0 (0-0) | 0 (0-0) |
| Bosnia and Herzegovina | 0 (0-0) | 0 (0-0) | 0 (0-0) | 0 (0-0) | 0 (0-0) | 0 (0-0) | 0 (0-0) | 0 (0-0) | 0 (0-0) | 0 (0-0) | 0 (0-0) |
| Botswana | 0 (0-0) | 0 (0-0) | 0 (0-0) | 0 (0-0) | 0 (0-0) | 0 (0-0) | 0 (0-0) | 0 (0-0) | 0 (0-0) | 0 (0-0) | 0 (0-0) |
| Bulgaria | 0 (0-0) | 0 (0-0) | 0 (0-0) | 0 (0-0) | 0 (0-0) | 0 (0-0) | 0 (0-0) | 0 (0-0) | 0 (0-0) | 0 (0-0) | 0 (0-0) |
| Burkina Faso | 0 (0-0) | 0 (0-0) | 0 (0-0) | 0 (0-0) | 0 (0-0) | 0 (0-0) | 0 (0-0) | 0 (0-0) | 0 (0-0) | 0 (0-0) | 0 (0-0) |
| Cabo Verde | 0 (0-0) | 0 (0-0) | 0 (0-0) | 0 (0-0) | 0 (0-0) | 0 (0-0) | 0 (0-0) | 0 (0-0) | 0 (0-0) | 0 (0-0) | 0 (0-0) |
| Cameroon | 0 (0-0) | 0 (0-0) | 0 (0-0) | 0 (0-0) | 0 (0-0) | 0 (0-0) | 0 (0-0) | 0 (0-0) | 0 (0-0) | 0 (0-0) | 0 (0-0) |
| Central African Republic | 0 (0-0) | 0 (0-0) | 0 (0-0) | 0 (0-0) | 0 (0-0) | 0 (0-0) | 0 (0-0) | 0 (0-0) | 0 (0-0) | 0 (0-0) | 0 (0-0) |
| Chad | 0 (0-0) | 0 (0-0) | 0 (0-0) | 0 (0-0) | 0 (0-0) | 0 (0-0) | 0 (0-0) | 0 (0-0) | 0 (0-0) | 0 (0-0) | 0 (0-0) |
| Colombia | 0 (0-0) | 0 (0-0) | 0 (0-0) | 0 (0-0) | 0 (0-0) | 0 (0-0) | 0 (0-0) | 0 (0-0) | 0 (0-0) | 0 (0-0) | 0 (0-0) |
| Comoros | 0 (0-0) | 0 (0-0) | 0 (0-0) | 0 (0-0) | 0 (0-0) | 0 (0-0) | 0 (0-0) | 0 (0-0) | 0 (0-0) | 0 (0-0) | 0 (0-0) |
| Congo | 0 (0-0) | 0 (0-0) | 0 (0-0) | 0 (0-0) | 0 (0-0) | 0 (0-0) | 0 (0-0) | 0 (0-0) | 0 (0-0) | 0 (0-0) | 0 (0-0) |
| Cook Islands | 0 (0-0) | 0 (0-0) | 0 (0-0) | 0 (0-0) | 0 (0-0) | 0 (0-0) | 0 (0-0) | 0 (0-0) | 0 (0-0) | 0 (0-0) | 0 (0-0) |
| Coted'Ivoire | 0 (0-0) | 0 (0-0) | 0 (0-0) | 0 (0-0) | 0 (0-0) | 0 (0-0) | 0 (0-0) | 0 (0-0) | 0 (0-0) | 0 (0-0) | 0 (0-0) |
| Croatia | 0 (0-0) | 0 (0-0) | 0 (0-0) | 0 (0-0) | 0 (0-0) | 0 (0-0) | 0 (0-0) | 0 (0-0) | 0 (0-0) | 0 (0-0) | 0 (0-0) |
| Cuba | 0 (0-0) | 0 (0-0) | 0 (0-0) | 0 (0-0) | 0 (0-0) | 0 (0-0) | 0 (0-0) | 0 (0-0) | 0 (0-0) | 0 (0-0) | 0 (0-0) |
| Cyprus | 0 (0-0) | 0 (0-0) | 0 (0-0) | 0 (0-0) | 0 (0-0) | 0 (0-0) | 0 (0-0) | 0 (0-0) | 0 (0-0) | 0 (0-0) | 0 (0-0) |
| Dominican Republic | 0 (0-0) | 0 (0-0) | 0 (0-0) | 0 (0-0) | 0 (0-0) | 0 (0-0) | 0 (0-0) | 0 (0-0) | 0 (0-0) | 0 (0-0) | 0 (0-0) |
| Eswatini | 0 (0-0) | 0 (0-0) | 0 (0-0) | 0 (0-0) | 0 (0-0) | 0 (0-0) | 0 (0-0) | 0 (0-0) | 0 (0-0) | 0 (0-0) | 0 (0-0) |
| Ethiopia | 0 (0-0) | 0 (0-0) | 0 (0-0) | 0 (0-0) | 0 (0-0) | 0 (0-0) | 0 (0-0) | 0 (0-0) | 0 (0-0) | 0 (0-0) | 0 (0-0) |
| Gabon | 0 (0-0) | 0 (0-0) | 0 (0-0) | 0 (0-0) | 0 (0-0) | 0 (0-0) | 0 (0-0) | 0 (0-0) | 0 (0-0) | 0 (0-0) | 0 (0-0) |
| Gambia | 0 (0-0) | 0 (0-0) | 0 (0-0) | 0 (0-0) | 0 (0-0) | 0 (0-0) | 0 (0-0) | 0 (0-0) | 0 (0-0) | 0 (0-0) | 0 (0-0) |
| Greenland | 0 (0-0) | 0 (0-0) | 0 (0-0) | 0 (0-0) | 0 (0-0) | 0 (0-0) | 0 (0-0) | 0 (0-0) | 0 (0-0) | 0 (0-0) | 0 (0-0) |
| Guam | 0 (0-0) | 0 (0-0) | 0 (0-0) | 0 (0-0) | 0 (0-0) | 0 (0-0) | 0 (0-0) | 0 (0-0) | 0 (0-0) | 0 (0-0) | 0 (0-0) |
| Guinea | 0 (0-0) | 0 (0-0) | 0 (0-0) | 0 (0-0) | 0 (0-0) | 0 (0-0) | 0 (0-0) | 0 (0-0) | 0 (0-0) | 0 (0-0) | 0 (0-0) |
| Guinea-Bissau | 0 (0-0) | 0 (0-0) | 0 (0-0) | 0 (0-0) | 0 (0-0) | 0 (0-0) | 0 (0-0) | 0 (0-0) | 0 (0-0) | 0 (0-0) | 0 (0-0) |
| Hungary | 0 (0-0) | 0 (0-0) | 0 (0-0) | 0 (0-0) | 0 (0-0) | 0 (0-0) | 0 (0-0) | 0 (0-0) | 0 (0-0) | 0 (0-0) | 0 (0-0) |
| Iran (Islamic Republic of) | 0 (0-0) | 0 (0-0) | 0 (0-0) | 0 (0-0) | 0 (0-0) | 0 (0-0) | 0 (0-0) | 0 (0-0) | 0 (0-0) | 0 (0-0) | 0 (0-0) |
| Kenya | 0 (0-0) | 0 (0-0) | 0 (0-0) | 0 (0-0) | 0 (0-0) | 0 (0-0) | 0 (0-0) | 0 (0-0) | 0 (0-0) | 0 (0-0) | 0 (0-0) |
| Lebanon | 0 (0-0) | 0 (0-0) | 0 (0-0) | 0 (0-0) | 0 (0-0) | 0 (0-0) | 0 (0-0) | 0 (0-0) | 0 (0-0) | 0 (0-0) | 0 (0-0) |
| Liberia | 0 (0-0) | 0 (0-0) | 0 (0-0) | 0 (0-0) | 0 (0-0) | 0 (0-0) | 0 (0-0) | 0 (0-0) | 0 (0-0) | 0 (0-0) | 0 (0-0) |
| Malawi | 0 (0-0) | 0 (0-0) | 0 (0-0) | 0 (0-0) | 0 (0-0) | 0 (0-0) | 0 (0-0) | 0 (0-0) | 0 (0-0) | 0 (0-0) | 0 (0-0) |
| Mali | 0 (0-0) | 0 (0-0) | 0 (0-0) | 0 (0-0) | 0 (0-0) | 0 (0-0) | 0 (0-0) | 0 (0-0) | 0 (0-0) | 0 (0-0) | 0 (0-0) |
| Mauritius | 0 (0-0) | 0 (0-0) | 0 (0-0) | 0 (0-0) | 0 (0-0) | 0 (0-0) | 0 (0-0) | 0 (0-0) | 0 (0-0) | 0 (0-0) | 0 (0-0) |
| Monaco | 0 (0-0) | 0 (0-0) | 0 (0-0) | 0 (0-0) | 0 (0-0) | 0 (0-0) | 0 (0-0) | 0 (0-0) | 0 (0-0) | 0 (0-0) | 0 (0-0) |
| Namibia | 0 (0-0) | 0 (0-0) | 0 (0-0) | 0 (0-0) | 0 (0-0) | 0 (0-0) | 0 (0-0) | 0 (0-0) | 0 (0-0) | 0 (0-0) | 0 (0-0) |
| Nauru | 0 (0-0) | 0 (0-0) | 0 (0-0) | 0 (0-0) | 0 (0-0) | 0 (0-0) | 0 (0-0) | 0 (0-0) | 0 (0-0) | 0 (0-0) | 0 (0-0) |
| Nepal | 0 (0-0) | 0 (0-0) | 0 (0-0) | 0 (0-0) | 0 (0-0) | 0 (0-0) | 0 (0-0) | 0 (0-0) | 0 (0-0) | 0 (0-0) | 0 (0-0) |
| Niger | 0 (0-0) | 0 (0-0) | 0 (0-0) | 0 (0-0) | 0 (0-0) | 0 (0-0) | 0 (0-0) | 0 (0-0) | 0 (0-0) | 0 (0-0) | 0 (0-0) |
| Nigeria | 0 (0-0) | 0 (0-0) | 0 (0-0) | 0 (0-0) | 0 (0-0) | 0 (0-0) | 0 (0-0) | 0 (0-0) | 0 (0-0) | 0 (0-0) | 0 (0-0) |
| Northern Mariana Islands | 0 (0-0) | 0 (0-0) | 0 (0-0) | 0 (0-0) | 0 (0-0) | 0 (0-0) | 0 (0-0) | 0 (0-0) | 0 (0-0) | 0 (0-0) | 0 (0-0) |
| Palau | 0 (0-0) | 0 (0-0) | 0 (0-0) | 0 (0-0) | 0 (0-0) | 0 (0-0) | 0 (0-0) | 0 (0-0) | 0 (0-0) | 0 (0-0) | 0 (0-0) |
| Puerto Rico | 0 (0-0) | 0 (0-0) | 0 (0-0) | 0 (0-0) | 0 (0-0) | 0 (0-0) | 0 (0-0) | 0 (0-0) | 0 (0-0) | 0 (0-0) | 0 (0-0) |
| Saint Kitts and Nevis | 0 (0-0) | 0 (0-0) | 0 (0-0) | 0 (0-0) | 0 (0-0) | 0 (0-0) | 0 (0-0) | 0 (0-0) | 0 (0-0) | 0 (0-0) | 0 (0-0) |
| Saint Vincent and the Grenadines | 0 (0-0) | 0 (0-0) | 0 (0-0) | 0 (0-0) | 0 (0-0) | 0 (0-0) | 0 (0-0) | 0 (0-0) | 0 (0-0) | 0 (0-0) | 0 (0-0) |
| Sao Tome and Principe | 0 (0-0) | 0 (0-0) | 0 (0-0) | 0 (0-0) | 0 (0-0) | 0 (0-0) | 0 (0-0) | 0 (0-0) | 0 (0-0) | 0 (0-0) | 0 (0-0) |
| Saudi Arabia | 0 (0-0) | 0 (0-0) | 0 (0-0) | 0 (0-0) | 0 (0-0) | 0 (0-0) | 0 (0-0) | 0 (0-0) | 0 (0-0) | 0 (0-0) | 0 (0-0) |
| Senegal | 0 (0-0) | 0 (0-0) | 0 (0-0) | 0 (0-0) | 0 (0-0) | 0 (0-0) | 0 (0-0) | 0 (0-0) | 0 (0-0) | 0 (0-0) | 0 (0-0) |
| Sierra Leone | 0 (0-0) | 0 (0-0) | 0 (0-0) | 0 (0-0) | 0 (0-0) | 0 (0-0) | 0 (0-0) | 0 (0-0) | 0 (0-0) | 0 (0-0) | 0 (0-0) |
| Somalia | 0 (0-0) | 0 (0-0) | 0 (0-0) | 0 (0-0) | 0 (0-0) | 0 (0-0) | 0 (0-0) | 0 (0-0) | 0 (0-0) | 0 (0-0) | 0 (0-0) |
| South Africa | 0 (0-0) | 0 (0-0) | 0 (0-0) | 0 (0-0) | 0 (0-0) | 0 (0-0) | 0 (0-0) | 0 (0-0) | 0 (0-0) | 0 (0-0) | 0 (0-0) |
| South Sudan | 0 (0-0) | 0 (0-0) | 0 (0-0) | 0 (0-0) | 0 (0-0) | 0 (0-0) | 0 (0-0) | 0 (0-0) | 0 (0-0) | 0 (0-0) | 0 (0-0) |
| Sudan | 0 (0-0) | 0 (0-0) | 0 (0-0) | 0 (0-0) | 0 (0-0) | 0 (0-0) | 0 (0-0) | 0 (0-0) | 0 (0-0) | 0 (0-0) | 0 (0-0) |
| Syrian Arab Republic | 0 (0-0) | 0 (0-0) | 0 (0-0) | 0 (0-0) | 0 (0-0) | 0 (0-0) | 0 (0-0) | 0 (0-0) | 0 (0-0) | 0 (0-0) | 0 (0-0) |
| Togo | 0 (0-0) | 0 (0-0) | 0 (0-0) | 0 (0-0) | 0 (0-0) | 0 (0-0) | 0 (0-0) | 0 (0-0) | 0 (0-0) | 0 (0-0) | 0 (0-0) |
| Tokelau | 0 (0-0) | 0 (0-0) | 0 (0-0) | 0 (0-0) | 0 (0-0) | 0 (0-0) | 0 (0-0) | 0 (0-0) | 0 (0-0) | 0 (0-0) | 0 (0-0) |
| United Kingdom | 0 (0-0) | 0 (0-0) | 0 (0-0) | 0 (0-0) | 0 (0-0) | 0 (0-0) | 0 (0-0) | 0 (0-0) | 0 (0-0) | 0 (0-0) | 0 (0-0) |
| United Republic of Tanzania | 0 (0-0) | 0 (0-0) | 0 (0-0) | 0 (0-0) | 0 (0-0) | 0 (0-0) | 0 (0-0) | 0 (0-0) | 0 (0-0) | 0 (0-0) | 0 (0-0) |
| Yemen | 0 (0-0) | 0 (0-0) | 0 (0-0) | 0 (0-0) | 0 (0-0) | 0 (0-0) | 0 (0-0) | 0 (0-0) | 0 (0-0) | 0 (0-0) | 0 (0-0) |
| Zimbabwe | 0 (0-0) | 0 (0-0) | 0 (0-0) | 0 (0-0) | 0 (0-0) | 0 (0-0) | 0 (0-0) | 0 (0-0) | 0 (0-0) | 0 (0-0) | 0 (0-0) |
| **Location Name** | 2011 | 2012 | 2013 | 2014 | 2015 | 2016 | 2017 | 2018 | 2019 | 2020 | 2021 |
| Australia | 16154.82 (14240-18521.06) | 16053.07 (13931.76-18546.64) | 15919.77 (13536.2-18693.84) | 15832.24 (13154.91-19086.61) | 15829.5 (12889.73-19386.27) | 15938.21 (12980.09-19642.7) | 16222.81 (13156.03-20037.3) | 16785.05 (13478.29-20898.55) | 17720.9 (13926.4-22354.84) | 30550.57 (24212.02-36250.9) | 31798.17 (25062.17-38027.35) |
| Madagascar | 22366.58 (14504.06-31010.27) | 22390.91 (14550.66-31145.36) | 22424.98 (14618.49-31351.81) | 22463.98 (14657.79-31542.59) | 22500.78 (14667.96-31573.22) | 22521.73 (14741.46-31648.72) | 22524.88 (14759.88-31515.12) | 22524.36 (14638.78-31426.13) | 22535.27 (14649.28-31761.38) | 22484.9 (14791.88-31919.21) | 22411.07 (14516.05-31277.2) |
| United States of America | 15107.41 (9155.47-21595.75) | 15292.97 (9358.03-21749.87) | 15518.64 (9597.77-21891.61) | 15738.67 (9839.62-22022.74) | 15904.62 (10065.42-22162.42) | 16185.06 (10367.22-22390.95) | 22298.93 (15342.22-29692.94) | 23450.11 (16438.67-30969.2) | 24843.66 (17591.1-32497.96) | 29652.42 (21430.62-38129.7) | 29623.13 (21560.4-38057.12) |
| Netherlands | 22293.17 (16260.35-29306.73) | 22344.56 (16283.5-29308.67) | 22386.83 (16370.25-29308) | 22387.03 (16382.5-29320.89) | 22362.27 (16320.07-29380.15) | 22341.56 (16376.72-29286.09) | 30065.52 (23094.76-38265.88) | 30066.3 (23154.91-38233.21) | 30045.34 (22982.61-38304.12) | 29990.22 (22818.99-38100.01) | 26057.46 (20248.57-32325.44) |
| Greece | 21704.86 (17747.57-26439.98) | 21303.51 (17375.32-25962.19) | 20735.89 (16879.92-25179.32) | 20121.88 (16353.82-24457.03) | 19607.74 (15916.74-23925.19) | 18201.88 (14783.41-22291.73) | 18005.47 (14081.74-22510.42) | 16459.07 (12241.62-21245.36) | 15759.65 (11411.41-20906.91) | 15772.49 (11617.49-20887.66) | 15841.38 (11557.79-20763.7) |
| Canada | 16814.61 (10678.2-24215.54) | 17547.16 (11290.08-24859.57) | 18389.34 (11911.22-25853.75) | 19101.56 (12247.68-26871.26) | 19428.38 (12319.21-27478.18) | 19474.54 (12382.61-27359.64) | 26055.07 (17464.65-35280.05) | 26083.9 (17452.47-35429.87) | 26084.46 (17467.98-35473.75) | 25825.52 (17054.62-35408.31) | 25975.4 (17108.37-35349.5) |
| Norway | 16414.25 (9991.18-23566.77) | 16996.75 (10444.94-24342.29) | 17684.59 (10953.99-25267.3) | 18246.67 (11402.31-25989.77) | 18477.43 (11566.99-26336.3) | 18460.11 (11589.43-26275.34) | 24590.26 (16260.15-33922.33) | 24558.24 (16209.94-33835.05) | 24533.98 (16173.53-33902.55) | 24679.67 (16194.65-33972.53) | 24365.8 (16069.34-33492.27) |
| Finland | 20010.13 (12509.6-28037.39) | 20012.35 (12603.46-28075.25) | 19998.45 (12638.09-28180.31) | 19980.69 (12757.21-28185.36) | 19988.61 (12822.13-28238.05) | 20036.34 (12839.11-28188.54) | 20097.11 (12793.22-28346.62) | 20140.03 (12730.66-28422.72) | 20128.36 (12680.17-28428.76) | 20022.26 (12678.36-28451.04) | 19973.14 (12725.1-28039.47) |
| New Zealand | 13102.87 (7484.03-19567.77) | 13140.9 (7492.12-19697.9) | 13170.38 (7504.24-19851.11) | 13183.06 (7471.33-19899.09) | 13175.02 (7462.79-19914.28) | 13168.24 (7452.99-19921.82) | 13178.8 (7496.39-19845.04) | 13185.7 (7564.08-19751.06) | 13172.25 (7618.23-19708.42) | 12422.06 (6515.6-19340.65) | 12360.19 (6381.59-19206.46) |
| Ireland | 21657.25 (13805.75-30464.69) | 21752.43 (14007.57-30454.24) | 21835.25 (14168.1-30538.05) | 21894.64 (14226.53-30623.32) | 21908.47 (14152.59-30686.04) | 21825.62 (14140.4-30618.63) | 29092.29 (19631.06-39831.95) | 28861.26 (19388.65-39426.08) | 28748.22 (19074.88-39471.14) | 29062.52 (19145.62-39956.43) | 29068.78 (19370.67-40572.05) |
| Bhutan | 21705.47 (14167.6-29936.44) | 21639.34 (14090.94-30069.38) | 21583.58 (13955.83-29937.62) | 21542.98 (13871.81-29765.57) | 21522.29 (13815.49-29888.61) | 21519.66 (13884.92-29780.07) | 21525.92 (13915.15-29788.9) | 21530.03 (13836.68-29836.63) | 21523.89 (13824.6-29903.92) | 21347.18 (13877.24-29693.94) | 21384.4 (13924.04-29550.1) |
| Switzerland | 11573.22 (8233.62-15913.65) | 11526.08 (8225.73-15916.2) | 11457.62 (8154.95-15987.86) | 11375.82 (8037.58-16062.29) | 11290.6 (7900.76-16151.43) | 11190.93 (7788.51-16174.86) | 11073.57 (7533.24-16205.09) | 10971.15 (7323.48-16324.33) | 10914.19 (7177.82-16414.3) | 10892.98 (7176.92-16160.85) | 10878.97 (7204.95-16270.15) |
| Russian Federation | 31579.18 (22785.68-40859.09) | 31648.83 (22834.07-40998.61) | 31693.52 (22885.96-41111.99) | 31747.34 (22939.6-41233.62) | 31839.44 (22982.66-41361.82) | 31944.15 (23056.25-41379.68) | 43829.12 (32828.89-55150.68) | 43914.73 (32878.24-55131.81) | 43917.37 (32877.18-55099.65) | 43826.88 (32936.82-54971.18) | 43610.15 (33794.32-53727.38) |
| Kyrgyzstan | 30743.13 (22300.54-39873.28) | 30838.32 (22316.32-39954.93) | 30971.17 (22364.05-40005.21) | 31106.56 (22452.02-40302.23) | 31218.62 (22591.18-40501.65) | 31300.5 (22612.52-40617.34) | 42875.08 (32152.93-54286.52) | 42963.89 (32308.16-54589.34) | 43070.06 (32330.27-54526.66) | 43286.43 (32491.45-54717.58) | 43220.29 (32246.44-54239.98) |
| Ukraine | 31575.71 (22883.37-41016.63) | 31639.32 (22891.06-40924.84) | 31739.62 (22984.21-40923.8) | 31954.82 (23169.31-41207.77) | 32230.4 (23347.95-41649.74) | 32436.08 (23425.61-41687.9) | 44523.71 (33416.72-56157.61) | 44527.63 (33467.44-56245.64) | 44489.63 (33379.8-56157.07) | 44355.54 (33529.31-55645.7) | 44164.09 (33180.99-55442.38) |
| Brunei Darussalam | 26708.22 (18593.2-35356.93) | 26707.85 (18619.22-35454.52) | 26717.77 (18628.78-35379.3) | 26733.67 (18587.08-35402.97) | 26758.21 (18517.57-35396.74) | 26770.75 (18514.84-35542.14) | 36376.73 (26205.91-47189.08) | 36313.77 (26067.17-46966.73) | 36247.67 (25957.07-46696.19) | 36121.57 (26119.73-46529.34) | 36384.64 (26439.62-47202.06) |
| Latvia | 29200.97 (19847.41-39082.33) | 28964.69 (19676.42-38629.14) | 28604.81 (19453.11-38027.04) | 28290.4 (19112.83-37621.4) | 28181.56 (18950.19-37446.94) | 28302.62 (19125.5-37636.4) | 38830.86 (27098.24-50169.51) | 39205.24 (27382.28-50672.25) | 39428.86 (27400.62-51311.2) | 39526.35 (27661.54-51459.15) | 39243.84 (27324.53-51312.55) |
| Tajikistan | 30868.51 (22193.67-40089.02) | 30909.73 (22293.42-40182.86) | 30969.59 (22396.56-40316.85) | 31037.13 (22480.25-40455.54) | 31095.69 (22568.91-40565.92) | 31154.43 (22561.41-40523.55) | 42639.94 (32157.72-53708.19) | 42741.72 (32221.49-53890.75) | 42820.24 (32350.9-54238.07) | 42715.75 (32110.18-54070.01) | 42866.67 (32404.22-54007.57) |
| Germany | 16073.63 (12507.46-20416.45) | 15657.79 (12162.71-19871.02) | 15125.85 (11680.4-19238.58) | 14618.5 (11179.7-18737.48) | 14252.64 (10765.94-18416.4) | 14009.46 (10546.78-18195.81) | 17590.55 (14177.85-21920.62) | 17360.99 (13867.03-21757.7) | 17280.67 (13653.92-21781.89) | 17373.17 (13668.64-21850.53) | 17298 (13427.15-23184.68) |
| Equatorial Guinea | 19012.77 (11983.2-27491.36) | 19029.25 (11953.48-27560.91) | 19045.77 (11889.41-27599.28) | 19067.3 (11849.61-27805.52) | 19101.99 (11842.65-27943.94) | 19195.55 (11855.24-28031.45) | 19346.7 (12006.39-28247.78) | 19492.25 (12133.3-28296.4) | 19570.02 (12169.9-28449.64) | 19255.3 (12072.09-27756.64) | 19356.92 (11906.42-27993.66) |
| Andorra | 20115.81 (12661.94-28537.32) | 20235.92 (12721.87-28776.83) | 20319.47 (12722.83-28939.91) | 20374.16 (12729.72-29050.86) | 20292.77 (12711.39-29109.53) | 20237.28 (12713.74-28863.42) | 27021.03 (17807.49-37494.62) | 26814.84 (17635.76-37289.72) | 26540.37 (17447.51-36916.08) | 26457.68 (17318.13-36118.48) | 26575.75 (17583.9-36155.05) |
| Honduras | 25730.36 (17036.71-34839.77) | 25693.88 (17074.71-34753.23) | 25651.19 (17067.33-34686.22) | 25615.81 (17029.2-34894.87) | 25600.43 (16999.65-35028.46) | 25608.55 (17040.42-34963.77) | 34681.53 (23958.62-46117.01) | 34721.37 (23919-46322.64) | 34750.7 (23970.18-46403.44) | 34732.26 (24312.85-45979.11) | 34750.35 (24356.78-46314.74) |
| Mongolia | 30256.7 (21738.89-38880.36) | 30319.34 (21737.26-39078.48) | 30404.89 (21830.75-39338.29) | 30526.99 (21868.65-39623.76) | 30679.4 (21944.48-39938.76) | 30839.57 (22082.69-40158.64) | 42368.19 (31578.94-53403.65) | 42547.79 (31709.31-53745.74) | 42667.48 (31655.24-54189.38) | 42686.2 (31710.99-54498) | 42411.88 (31704.43-53645.81) |
| Chile | 27621.08 (19914.99-35680.48) | 27702.06 (19939.24-35871.94) | 27725.67 (19884.42-35944.49) | 27715.72 (19797.7-35933.77) | 27764.35 (19763.61-35858.57) | 27899.41 (19890.07-36044.07) | 38062.89 (28385.39-47886.34) | 38127.32 (28523.02-47824.03) | 38124.9 (28543.01-47729.95) | 38202.12 (28530.07-47772.25) | 38247.98 (28283.31-48308.56) |
| Austria | 19786 (12429.58-27985.65) | 19780 (12403.57-27888.53) | 19776.99 (12290.91-27914.49) | 19748.05 (12122.08-28058.66) | 19695.16 (11997.21-27936.15) | 19632.25 (12060.6-27880.1) | 26166.56 (17156.64-35811.29) | 26206.55 (17204.33-35925.44) | 26275.02 (17336.97-36051.99) | 26310.06 (17219.8-36210.87) | 26446.02 (17194.13-36347.39) |
| Belarus | 31358.97 (22169.16-41015.63) | 31370.3 (22170.44-40996.01) | 31377.47 (22147.13-41036.16) | 31453.43 (22144.7-41125.88) | 31582.5 (22254.41-41476.28) | 31737.96 (22365.35-41599.31) | 43613.84 (31980.45-55901.72) | 43844.7 (32234.94-56468.34) | 43910.74 (32124.12-56780.7) | 43690.17 (32134.32-56041.32) | 43713.61 (32070.92-55917.02) |
| Kazakhstan | 30290.17 (21583.34-39243.69) | 30396.77 (21726.6-39372.98) | 30461.02 (21672.96-39450.61) | 30510.37 (21624.92-39452.57) | 30571.81 (21720.15-39401.72) | 30588.6 (21742.7-39582.02) | 41714.49 (31034.76-52767.55) | 41564.28 (31037.52-52574.4) | 41414.87 (31030.75-52535.84) | 41809.89 (31059.92-52418.73) | 41905.97 (31089.54-53006.57) |
| Slovakia | 30665.1 (21993.17-39830.71) | 30998.4 (22261.14-40257.9) | 31232.17 (22560.95-40585.71) | 31232.45 (22476.58-40584.75) | 31056.73 (22264.22-40352.84) | 30808.55 (22108.59-39917.67) | 41761.55 (31422.45-52445.48) | 41649.39 (31340.93-52317.19) | 41680.5 (31127.9-52256.84) | 41770.65 (31185.2-52618.79) | 41970.35 (31318.06-53114.59) |
| Rwanda | 21069.95 (13572.36-29682.25) | 20998.68 (13497.97-29569.87) | 20916.24 (13365.87-29413.81) | 20847.15 (13239.36-29356.9) | 20811.7 (13152.12-29518.02) | 20800.37 (13098.95-29489.33) | 20786.63 (13058.8-29521.56) | 20777.07 (13047.37-29524.5) | 20778.69 (13009.66-29424.53) | 20831.72 (13168.11-30395.43) | 20739.78 (13116.7-29570.03) |
| Iceland | 19587.65 (12360.98-27601.44) | 19739.22 (12497.7-27795.38) | 19888.45 (12570.68-27985.87) | 19955.96 (12585.72-28203.24) | 19967.37 (12561.47-28333.8) | 19905.36 (12531.78-28127.15) | 26453.26 (17424.62-36086.67) | 26243.84 (17180.41-35928.27) | 26053.38 (17027.59-35766.58) | 25907.59 (17213.1-35882.12) | 26228.36 (17292.95-36037.78) |
| Spain | 20097.37 (14000.28-27368.89) | 20188.94 (14182.66-27320.99) | 20295.63 (14223.42-27209.7) | 20353.47 (14095.94-27367.12) | 20350.16 (13779.24-27424.99) | 20049.16 (13472.56-27203.26) | 25963.99 (17731.22-34234.92) | 25127.51 (16391.37-34079.01) | 24747.42 (15720.79-34278.53) | 24982.46 (15908.29-34372.46) | 24749.69 (15245.29-34230.05) |
| Eritrea | 20807.61 (13195.71-29656.52) | 20789.32 (13212.48-29554.2) | 20779.54 (13212.19-29562.3) | 20782.18 (13233.17-29512.59) | 20800.83 (13222.71-29694.05) | 20835.46 (13226.97-29641.67) | 20878.43 (13252.25-29787.39) | 20919.43 (13243.52-30059.1) | 20945.87 (13259.23-30199.48) | 20809.25 (13073.73-29725.09) | 20763.24 (13246.95-29692.15) |
| Djibouti | 20504.66 (13086.95-29259.83) | 20511.8 (13051.37-29193.45) | 20519.37 (13066.51-29087.67) | 20528.7 (13068.78-29214.45) | 20542.44 (12999.36-29239.32) | 20540.18 (13101.28-29264.79) | 20507.1 (13105.47-29239.92) | 20469.71 (13131.19-29218.74) | 20455.92 (13101.37-29395.35) | 20527.73 (13000.37-29186.58) | 20563.55 (12988.79-29470.62) |
| Democratic Republic of the Congo | 20257.22 (12998.47-28359.47) | 20280.2 (13020.61-28438.23) | 20307.72 (13030.32-28705.03) | 20338.13 (13050.15-28814.87) | 20369.68 (13041.41-29027.33) | 20413.15 (13098.95-29018.42) | 20467.78 (13090.71-29215.32) | 20516.65 (13146.23-29381.79) | 20544.79 (13130.86-29467.64) | 20240.42 (12956.96-28794.36) | 20366.85 (12890.65-29375.44) |
| Poland | 31504.11 (23349.58-40532.31) | 31367.56 (23048.11-40477.72) | 31030.01 (22589.59-40152.75) | 30629.24 (22088.73-39757.99) | 30341.43 (21799.37-39504.86) | 30167.3 (21664.3-39285.37) | 41040.01 (30621.35-51887.48) | 41102.31 (30632.03-51872.62) | 41395.27 (30842.43-52115.03) | 41644.61 (31126.39-52506.94) | 41868.33 (31200.78-52836.33) |
| Burundi | 21054.05 (13577.38-30362.05) | 21111.13 (13615.19-30311.45) | 21130.48 (13631.11-30304.14) | 21121.43 (13604.24-30128.53) | 21131.04 (13557.28-30102.45) | 21135.42 (13482.36-29898.24) | 21090.32 (13424.85-29702.28) | 21008.85 (13444.12-29528.81) | 20957.64 (13285.36-29582.25) | 21182.66 (13712.29-30871.41) | 20981.93 (13585.25-30129.16) |
| Mozambique | 27163.7 (18271.42-36048.85) | 27161.86 (18249.66-36308.79) | 27154.02 (18222.37-36428.42) | 27145.44 (18222.91-36516.32) | 27143.28 (18167.78-36732.09) | 27152.99 (18231.81-36721.15) | 27170.76 (18351.95-36701.28) | 27192.62 (18378.66-36824.63) | 27210.47 (18392.18-37037.02) | 27206.36 (18443.76-36713) | 20888.16 (13358.76-29806.05) |
| Lesotho | 19979.26 (12314.25-29530.33) | 19962.18 (12296.58-29361.05) | 19963.74 (12263.53-29198.54) | 19978.06 (12299.1-29139.75) | 19996.29 (12338.8-29218.43) | 19995.08 (12340.17-29161.29) | 19970.62 (12340.17-29213.23) | 19942.38 (12241.81-29333.11) | 19932.27 (12174.27-29368.2) | 20214.58 (12388.36-29995.1) | 19693.41 (12257.86-29473.48) |
| Pakistan | 23949.95 (19141.99-29128.45) | 23760.78 (18991.97-28793.63) | 23532.24 (18771.5-28514.18) | 23347.26 (18678.75-28352.67) | 23282.5 (18626.2-28244.88) | 23725.67 (18939.28-28896.14) | 24686.64 (19399.94-30198.91) | 25682.53 (19857.89-31973.49) | 26231.06 (20040.11-33276.9) | 26272.59 (20077.96-33460.21) | 26321.99 (20006.95-33185.82) |
| Trinidad and Tobago | 18798.59 (11566.4-27950.69) | 18781.36 (11582.4-27798.38) | 18769.74 (11617.39-27732.47) | 18767.84 (11657.03-27694.87) | 18777.11 (11637.14-27569.13) | 18775.9 (11636.26-27715.53) | 18751.98 (11620.93-27676.8) | 18718.4 (11623.62-27570.3) | 18694.27 (11607.96-27513.43) | 18791.1 (11605.88-28135.07) | 23245.02 (15457.32-31883.12) |
| Ecuador | 28399.4 (24822.54-32233.24) | 28318.96 (24767.02-32063.69) | 28239.75 (24716.87-31924.86) | 28165.33 (24639.1-31818.53) | 28104.99 (24598.71-31809.46) | 28055.1 (24548.7-31753.59) | 28016.36 (24561.86-31669.13) | 28006.16 (24491.27-31677.6) | 28032.83 (24483.09-31775.28) | 28129.4 (24549.64-31907.68) | 28246.48 (24701.98-31938.49) |
| Panama | 22126.4 (14132.91-31088.08) | 22065.91 (14175.66-31292.02) | 22067.27 (14186.09-31642.27) | 22115.07 (14229.14-32013.99) | 22165.04 (14241.23-32237.25) | 22176.07 (14234.6-31982.59) | 22147.29 (14199.44-31625.3) | 22114.89 (14165.33-31318.07) | 22129.09 (14133.47-31361.55) | 22213.48 (14121.75-31476.55) | 22260.23 (14228.2-31705.36) |
| Kuwait | 24871.25 (17724.41-32752.54) | 25009.37 (17548.13-33241.08) | 25202.39 (17486.44-34079.45) | 25401.33 (17084.2-34618.17) | 25547.04 (16695.77-35097.6) | 25639.98 (16826.84-35052.11) | 25727.34 (16883.21-35163.81) | 25822.18 (17008.54-35290.85) | 25925.04 (17078.83-35535.23) | 26204.73 (17636.86-35920.74) | 25915.36 (17390.21-35703.92) |
| Nicaragua | 22654.77 (14707.47-32148.89) | 22627.26 (14592.36-32156.25) | 22611.18 (14449.84-32043.82) | 22610.51 (14385.35-32017.38) | 22626.13 (14297.06-32061.73) | 22673.01 (14519.68-32031.22) | 22746.86 (14723.77-31998.23) | 22820.25 (14811.49-32033.24) | 22863.39 (14774.06-32100.98) | 22657.09 (14515.18-32894.26) | 22648.65 (14573.91-31875.28) |
| Oman | 26580.61 (17739.93-35866.36) | 26619.23 (17919.39-35867.56) | 26680.66 (17990.51-35951.08) | 26760.37 (18051.96-36028.96) | 26846.9 (18020.04-36187.26) | 26928.67 (18104.86-36203.89) | 27004.05 (18153.61-36437.44) | 27063.26 (18067.14-36494.91) | 27121.85 (18010.83-36760.6) | 27263.9 (18280.28-37523.89) | 27360.21 (18278.92-37876.11) |
| Argentina | 27700.69 (19436.05-35807.42) | 27688.38 (19496.86-35828.43) | 27615.66 (19520.74-35672.78) | 27514.79 (19501.45-35772.23) | 27497.79 (19531.07-35917.76) | 27630.5 (19539.08-35930.97) | 27833.99 (19688.34-36096.57) | 28013.81 (19778.86-36329.33) | 28164.8 (19861.07-36594.21) | 25689.31 (16729.86-34808.81) | 25690.66 (16875.81-35327.77) |
| Venezuela (Bolivarian Republic of) | 22031.53 (14079.81-31178.39) | 21969.52 (14071.91-31027.51) | 21954.61 (14099.25-30960.06) | 21947.99 (14140.24-30846.3) | 21911.39 (14117.79-30754.11) | 21882.43 (14175.35-30660.51) | 21936.51 (14180.89-30843.94) | 22112.97 (14285.1-31238.46) | 22327.97 (14357.85-31718.41) | 22220.24 (14264.89-31464.51) | 22149.49 (14035.27-31697.6) |
| France | 12204.26 (7466.87-18239.33) | 12780.31 (7853.2-18740.19) | 13522.92 (8203.82-19742.54) | 14264.27 (8362.85-20891.9) | 14843.68 (8506.16-21900.11) | 15355.54 (8685.92-22495.23) | 20901.28 (12532.53-29402.16) | 21576.39 (12619-30507.53) | 21985.2 (12450.99-31248.13) | 22118.98 (12814.17-31146.79) | 22154.44 (12632.36-31551.69) |
| Japan | 21043.72 (15495.65-27662.7) | 20560.24 (15057.54-27047.16) | 20030.07 (14556.69-26416.1) | 19603.74 (14229.93-25836.8) | 19425.07 (14081.76-25595.19) | 19405.89 (14066.93-25604.35) | 26859.78 (19835.13-34893.07) | 26907.93 (19879.91-34894.18) | 27070.56 (19997.46-35070.52) | 29212.12 (21719.9-37476.61) | 29180.64 (21645.6-37636.83) |
| Mexico | 26872.99 (18692.69-35565.79) | 26867.02 (18677.06-35547.36) | 26877.78 (18679.53-35564.82) | 26910.22 (18675.83-35667.04) | 26960.4 (18695.59-35791.16) | 27194.49 (18963.96-35921.11) | 27652.17 (19462.85-36506.54) | 28143.82 (19897.44-37078.67) | 28466.49 (20095.41-37413.16) | 28397.77 (20411.05-36772.34) | 26812.69 (19875.32-34173.22) |
| Singapore | 18753.99 (13387.89-24528.64) | 18660.2 (13399.53-24428.79) | 18665.82 (13468.84-24414.99) | 18725.63 (13560.79-24428.14) | 18783.47 (13636.91-24544.3) | 19334.18 (13999.39-25038.06) | 20507.7 (14755.53-26667.1) | 21709.89 (15146.25-28408.1) | 22297.67 (15237.24-29551.46) | 22188.19 (15133.46-29564.45) | 22335.82 (15297.5-29493.75) |
| Haiti | 29116.58 (20458.7-37964.22) | 29095.76 (20528.99-37915.64) | 29079.63 (20490.9-37991.5) | 29109.64 (20485.62-38189.64) | 29156.04 (20534.2-38398.38) | 29212.03 (20590.8-38320.95) | 29255.88 (20685.02-38192.72) | 29282.14 (20690.88-38475.73) | 29282.77 (20667.25-38651.22) | 27866.9 (21575.54-34197.87) | 26287.78 (17870.35-35177.28) |
| Israel | 27612.77 (19392.35-35946.16) | 27557.85 (19355.12-35862.03) | 27471.32 (19323.94-35641.88) | 27375.25 (19258.6-35445.58) | 27312.36 (19255.19-35518.51) | 27271.29 (19235.98-35399.32) | 27227.04 (19195.95-35523.15) | 27207.1 (19171.72-35457.6) | 27236.46 (19229.48-35597.23) | 27462.24 (19547.45-35254.87) | 27435.95 (19279.23-35275.52) |
| Qatar | 29255.94 (21673-37292.96) | 29328.42 (21674.7-37436.58) | 29439.62 (21737.03-37636.09) | 29559.85 (21823.97-37881.14) | 29665.44 (21851.11-38214.41) | 29740.25 (21950.47-38104.29) | 29784.5 (22114.77-38012.23) | 29819.38 (22168.44-37826.36) | 29866.45 (22134.39-37787.68) | 30083.71 (22009.88-38421.64) | 30066.29 (22022.45-38088.18) |
| Republic of Korea | 34690.66 (27629.71-42486.13) | 34197.94 (27194.52-41995.55) | 33658.35 (26465.72-41566.01) | 33171.01 (25775.99-41523.43) | 32731.23 (25186.57-41640.58) | 32197.27 (24763.08-41071.68) | 41549.05 (33108.2-51429.73) | 40957.77 (32370.94-51056.37) | 40587.64 (31942.36-50889.47) | 40409.62 (31421.23-50914.08) | 39939.32 (31260.37-50299.43) |
| Italy | 16818.71 (12006.89-22222.99) | 16792.91 (11957.37-22184.39) | 16760.15 (11856.65-22079.87) | 16735.38 (11782.14-21963.66) | 16751.33 (11772.15-21984.78) | 17068.74 (12006.56-22520.16) | 17735.77 (12578.78-23563.81) | 18431.1 (12950.77-24700.72) | 18826.29 (13134.39-25408.19) | 18915 (13274.91-25621.48) | 18895.14 (13286.82-25346.72) |
| Morocco | 24532.59 (16390.48-33790.31) | 24605.2 (16422.37-33801.41) | 24695.28 (16411.07-34032.66) | 24786.75 (16417.42-34315.9) | 24859.4 (16372.66-34540.82) | 24923.48 (16442.77-34670.92) | 24993.11 (16472.83-34877.22) | 25057.34 (16444.68-35224.35) | 25105.38 (16327.28-35434.5) | 25005.07 (16426.72-34350.19) | 25076.98 (16486.59-34476.43) |
| Lithuania | 42751.33 (36665.09-48634.32) | 42948.69 (37027.96-48392.4) | 43058.57 (37140.75-48157.54) | 43083.08 (37021.23-48017.63) | 43051.72 (36794.56-47957.63) | 43020.34 (36835.53-47880.61) | 58134.57 (51893.84-62182.54) | 58008.57 (51932.04-62095.34) | 57773.51 (51539.22-62054.31) | 55308.98 (45366.91-65541.67) | 46985.7 (35537.71-59538.09) |
| United Arab Emirates | 28936.19 (24016.75-34355.54) | 28397.07 (23490.87-33901.57) | 27665.01 (22683.73-33240.16) | 27041.93 (21933.95-32676.67) | 26857.94 (21571.57-32505.95) | 27476.89 (22008.9-33403.32) | 28707.04 (22800.76-35036.7) | 30004.01 (23724.82-36682.01) | 30804.2 (24215.11-37700.51) | 31048.77 (24527.32-37744.9) | 29773.61 (22626.04-37158.34) |
| Algeria | 24290.81 (16031.38-33236.19) | 24287.27 (16202.99-32984.56) | 24287.3 (16145.59-32840.09) | 24303.61 (16058.89-32784.35) | 24346.43 (16070.23-32794.98) | 24416.61 (16237.18-32905.66) | 24507.06 (16309.52-33141.24) | 24612.87 (16363.06-33300.35) | 24724.79 (16426.92-33480.28) | 24994.9 (16291.82-34532.67) | 24994.87 (16529.09-34017.23) |
| Egypt | 23969.58 (15841.68-32710.35) | 23998.96 (15916.73-32653.32) | 24116.69 (16047.67-32884.46) | 24273.53 (16154.35-33207.47) | 24430.86 (16155.01-33452.58) | 24578.26 (16223.78-33722.53) | 24710.54 (16289.94-34132.64) | 24814.24 (16329.42-34464.83) | 24880.12 (16383.36-34680.49) | 24682.19 (16481.53-33749.92) | 24981.24 (16564.79-34042.51) |
| Iraq | 24840.98 (16565.41-33342.01) | 24851.61 (16530.58-33431.93) | 24838.75 (16473.24-33733.48) | 24837.06 (16369.62-33920.97) | 24871.96 (16380.82-34058.63) | 24885.49 (16473.04-34225.92) | 24843.34 (16436.84-33801.49) | 24785.5 (16368.85-33654.88) | 24766.27 (16262.91-33586.54) | 25143.53 (16705.05-34553.06) | 25027.85 (16546.73-34174.48) |
| Portugal | 19044.5 (12938.75-26620.11) | 19160.95 (12892.5-26818.57) | 19280.97 (12830.68-27041.6) | 19309.93 (12791.92-27104.42) | 19221.44 (12720.29-27177.91) | 19084.45 (12725.85-26983.79) | 18992.98 (12772.75-26877.21) | 18964.94 (12796.92-26827.12) | 18981.91 (12783.17-26934.66) | 19019.11 (12797.59-26611.46) | 19011.43 (12812.65-26384.75) |
| Afghanistan | 25530.23 (17022.4-34815.81) | 25438.49 (16980.27-34812.51) | 25342.15 (16942.97-34864.4) | 25263.15 (16839.11-34905.24) | 25216.94 (16766.53-34925.1) | 25216.38 (16879.49-34849.73) | 25229.41 (16930.28-34776.12) | 25240.55 (16875.15-34762.43) | 25253.71 (16813.39-34787.64) | 25285.54 (16783.91-34231.92) | 25351.86 (16839.16-34592.84) |
| Paraguay | 30686.19 (22365.11-39940.81) | 30654.55 (22288.88-39777.29) | 30623.04 (22220.5-39582.1) | 30585.52 (22098.4-39593.53) | 30533.79 (21961.75-39453.04) | 30320.33 (21859.92-39167.84) | 29927.7 (21521.1-39057.56) | 29537.02 (21139.72-38774.14) | 29327.13 (20925.34-38401.86) | 29272.73 (21116.67-37809.49) | 29248.8 (20820.57-37913.8) |
| Serbia | 34595.58 (26906.78-42889.71) | 34702.19 (26928.52-43156.25) | 34664.57 (26779.57-43106.69) | 34524.7 (26509.71-43078.57) | 34384.69 (26247.94-42933.27) | 34265.6 (26293.05-42682.29) | 46090.95 (36898.99-55665.44) | 45882.36 (36749.99-55210.64) | 45665.31 (36628.32-55163.83) | 33818.89 (26003.16-42815.82) | 33826.24 (25623.63-43062.8) |
| Romania | 37138.83 (28257.39-46212.99) | 37249.8 (28300.23-46248.3) | 37227.6 (28285.8-46234.67) | 37084.35 (28243.66-46006.28) | 36909.26 (27966.48-45888.16) | 36805.97 (27991.43-45789.15) | 50480.52 (39985.58-61228.35) | 50502.16 (39991.39-61350.52) | 50537.85 (40011.18-61661.35) | 50456.21 (39794.05-61045.82) | 50819.97 (40177.99-61233.35) |
| Libya | 24608.97 (16298.33-34022.15) | 24696.5 (16347.5-34173.94) | 24794.66 (16350.45-34087.98) | 24887.24 (16384.08-34006.64) | 24956.22 (16450.75-34017.71) | 24994.88 (16488.19-34107.44) | 25012.8 (16577.01-34072.75) | 25018.65 (16676.59-34152.3) | 25034.23 (16709.93-34333.32) | 25256.12 (16599.82-34488.99) | 25268.23 (16705.15-34333.81) |
| Brazil | 23013.18 (15878.36-30750.04) | 23115.77 (15960.42-30914.12) | 23262.21 (16008.89-31063.86) | 23426.64 (16081.01-31246.58) | 23581.82 (16154.69-31403.34) | 23780.55 (16296.14-31697.06) | 24048.61 (16476.41-32036.35) | 24318.24 (16613.92-32334.06) | 24520.61 (16706.83-32615.14) | 24719.56 (16763.16-32920.74) | 26833.36 (18111.14-35680.58) |
| Jordan | 25128.57 (19089.57-32689.55) | 25432.72 (19058.46-32714.54) | 25792.99 (18861.52-33652.47) | 26074.77 (18177.22-34752.1) | 26200.75 (17336.96-35890.02) | 26200.59 (17438.07-35856.64) | 26144.1 (17396.74-35910.29) | 26065.01 (17374.39-35909.08) | 26011.15 (17362.45-35767.38) | 26090.86 (17196.67-35430.56) | 26042.95 (17142.15-35575.53) |
| India | 29307.32 (20703.66-38215.37) | 29256.18 (20676.87-38149.16) | 29191.56 (20622.25-38062.02) | 29117.73 (20549.79-38028.3) | 29058.85 (20502.6-37988.29) | 29002.72 (20546.93-37809.58) | 28934.17 (20571.35-37605.65) | 28872.37 (20600.79-37426.27) | 28835 (20608.69-37330.92) | 28526.2 (21098.59-36367.49) | 27088.17 (20608.9-33826.85) |
| Saint Lucia | 28982.12 (20256.23-37808) | 28955.93 (20295.92-37782.12) | 28934.86 (20341.97-38022.85) | 28923.25 (20372.35-38177.78) | 28917.84 (20251.14-38506.75) | 28884.01 (20293.24-38263.91) | 28811.96 (20268.08-37904.52) | 28733.69 (20242.34-37826.75) | 28682.52 (20223.41-37777.13) | 28711.86 (20022.24-37512.87) | 27546.61 (21148.88-34117.05) |
| Bahamas | 27235.55 (19042.77-35842.59) | 27504.02 (19416.44-36170.33) | 27747.67 (19613.34-36343.39) | 27905.24 (19656.42-36660.66) | 27949.81 (19441.84-37020.65) | 27935.8 (19579.59-36872.51) | 27925.67 (19640.06-36704.17) | 27899.9 (19574.84-36629.82) | 27866.48 (19429.44-36536.55) | 27927.83 (19378.14-36813.73) | 27838.86 (19205.95-36817.75) |
| Palestine | 23959.61 (18464.76-30157.41) | 24478.02 (19724.29-29754.65) | 25178.05 (20788.94-29908.63) | 25960.73 (21326.92-30814.01) | 26742.94 (21352.21-32354.6) | 27744.78 (22109.17-33315.84) | 28969.56 (22868.06-34749.73) | 30009.91 (23411.24-36074.2) | 30458.21 (23433.91-36761.06) | 23593.64 (15419.36-32865.03) | 23440.39 (15254.39-32103.52) |
| Peru | 27349.92 (19092.29-35767.14) | 27282.23 (18979.23-35605.69) | 27213.59 (18845.53-35471.32) | 27170.15 (18773.97-35631.86) | 27161.8 (18725.58-35680.08) | 27162.11 (18729.61-35538.4) | 27145.44 (18692.62-35385.75) | 27125.96 (18607.25-35546.59) | 27130.69 (18587.22-35835.09) | 27294.54 (18761.22-36282.4) | 27168.12 (18672.27-36110.87) |
| Malta | 19794.07 (12507.4-28133.18) | 19879.96 (12513.7-28170.97) | 19972.2 (12526.67-28375.79) | 20034.42 (12482.97-28676.94) | 20047.02 (12434.97-28813.43) | 20024.11 (12497.63-28667.52) | 20003.71 (12580.76-28531.83) | 19990.76 (12654.9-28623.28) | 19984.31 (12648.37-28644.78) | 19864.53 (12457.37-28141.7) | 19909.17 (12532.92-28274.62) |
| Niue | 31343.2 (22208.65-40626.84) | 31214.9 (22154.12-40540.31) | 31105.46 (22066.74-40543.55) | 31023.3 (21943.32-40519.41) | 30967.29 (21928.48-40431.66) | 30923.22 (21863.67-40370.65) | 30888.95 (21834.7-40547.28) | 30877.05 (21815.66-40618.11) | 30869.88 (21804.83-40639.6) | 30976.56 (21727.44-40554.5) | 30931.49 (21679.69-40167.63) |
| Bolivia (Plurinational State of) | 27614.9 (19130.29-36870.37) | 27553.85 (19158.92-36706.85) | 27492.7 (19068.25-36619.06) | 27447.58 (18981.44-36661.89) | 27436.33 (19013.99-36644.75) | 27436.41 (18984.45-36401.94) | 27424.01 (19053.7-36152.59) | 27414.77 (19117.6-36112.74) | 27425.28 (19154.98-36232.22) | 27686.29 (19240.57-36935.75) | 27359.19 (18876.44-36109.06) |
| Ghana | 24647.08 (16399.69-34067.87) | 24583.18 (16368.48-33894.3) | 24512.45 (16301.13-33695.18) | 24455.34 (16177.06-33734.88) | 24432.44 (16090.06-33614.67) | 24439.74 (16066.46-33702.09) | 24457.57 (16097.55-33673.55) | 24480.74 (16134.73-33772.54) | 24503.04 (16129.45-33766.25) | 24337.91 (15891.84-33328.25) | 24509.1 (15974.42-33618.72) |
| Grenada | 28738.04 (20345.32-38079.93) | 28730.21 (20289.17-37863.48) | 28705.54 (20207.74-37679.23) | 28680.54 (20127.25-37615.51) | 28673.15 (19953.56-37803.95) | 28676.75 (20089.7-37522.89) | 28674.15 (20069.49-37677.27) | 28663.96 (20138.54-37617.64) | 28651.09 (20039.06-37594.88) | 28677.23 (20113.13-38464.05) | 28721.09 (20141.49-38053.16) |
| Zambia | 26634.56 (18035.59-35947.01) | 26603.74 (18022.79-35999.59) | 26577.4 (17989.3-35991.08) | 26568.35 (17961.05-36044.21) | 26590.38 (17941.05-36193.39) | 26658.03 (18133.97-36091.06) | 26753.72 (18279.49-36153.93) | 26844.65 (18285.14-36101.46) | 26899.31 (18208.55-36036.1) | 26600.7 (17927.16-35795.68) | 26928.55 (18063.34-36424.28) |
| Antigua and Barbuda | 28678.5 (20007.89-37457.86) | 28683.27 (20060.99-37695.97) | 28651.08 (20054.5-37868.44) | 28594.47 (20029.92-37785.77) | 28539.06 (19950.75-37886.74) | 28511.86 (19904.25-37693.69) | 28508.78 (19839.59-37805.39) | 28507.6 (19812.01-37681.93) | 28489.93 (19788.43-37683.21) | 28386.58 (19947.9-37458.65) | 28432.77 (19879.21-37492.67) |
| Seychelles | 29875.56 (20833.32-39121.44) | 29807.54 (20812.83-38970.08) | 29717.06 (20783.91-38889.33) | 29631.88 (20672.51-39062.76) | 29572.84 (20595.71-39104.6) | 29556.14 (20624.82-39014.65) | 29578.06 (20592.92-38946.92) | 29626.25 (20546.88-39061.76) | 29669.08 (20575.17-39170.39) | 29620.19 (20659.62-39341.72) | 29630.15 (20427.8-39357.95) |
| Dominica | 28991.36 (20417.97-37870.71) | 29034.76 (20401.25-37989.86) | 29143.73 (20392.89-38131.78) | 29290.15 (20420.53-38381.51) | 29429.2 (20405.72-38761.95) | 29516.34 (20644.63-38802.55) | 29532.39 (20769.18-38673.38) | 29470.06 (20657.62-38566.59) | 29362.33 (20547.67-38576.5) | 29311.74 (20764.42-38666.91) | 29149.55 (20358.04-38209.23) |
| Bahrain | 25741.32 (17265.63-34794.18) | 25849.21 (17300.45-34810.77) | 25942.45 (17311.39-35291.88) | 25994.13 (17301.2-35421.96) | 26036.93 (17277.68-35572.73) | 26122.97 (17346.5-35715.61) | 26244.16 (17448.15-35859.07) | 26354.63 (17576.17-36115.8) | 26431.83 (17684.3-36157.64) | 26550.3 (17497.26-36516.1) | 26556.72 (17726.4-36581.84) |
| Turkey | 28915.51 (21958.6-36338.32) | 28773.27 (21800.87-36050.3) | 28639.18 (21528.6-36114.42) | 28538.68 (21292.07-36077.29) | 28495.79 (21133.26-36096.38) | 28497.18 (21058.83-36034.61) | 28513.03 (21107.92-36010.29) | 28530.62 (21160.44-36083.46) | 28550.82 (21225.91-36313.12) | 28643.89 (20969.18-36511.01) | 27016.74 (17941.83-37478.69) |
| Mauritania | 24619.38 (16384.96-33721.21) | 24602.76 (16396.12-33593.36) | 24587.37 (16366.12-33517.72) | 24578.83 (16308.35-33392.71) | 24580.75 (16223.28-33338) | 24582.11 (16252.57-33469.8) | 24575.05 (16265.43-33675.35) | 24569.71 (16233.85-33878.11) | 24574.33 (16203.34-34207.42) | 24701.28 (16199.61-33963.82) | 24633.26 (16126.82-33974.47) |
| El Salvador | 25383.6 (17049.76-34454.83) | 25382.48 (17047.95-34322.18) | 25387.96 (16987.53-34300.11) | 25409.05 (16948.25-34332.31) | 25447.55 (16982.47-34518.93) | 25481.6 (17105.21-34370.9) | 25498.17 (17112.55-34336.19) | 25502.61 (17100.85-34492.74) | 25505.39 (17082.03-34622.63) | 25570.59 (16865.44-34934.14) | 25691.97 (16765.47-34606.94) |
| Uganda | 23857.63 (17673.01-30542.6) | 25082.33 (18687.04-31805.95) | 26551.16 (19609.8-33562.46) | 27801.77 (20232.35-35424.38) | 28362.31 (20204.39-36614.86) | 28029.04 (19952.61-36127.43) | 27211.83 (19027.11-35606.12) | 26370.25 (17965.33-35421.68) | 25975.64 (17275.4-35530.66) | 26029.53 (17274.44-35485.35) | 26092.79 (17089.88-35248.14) |
| Luxembourg | 19611.04 (12308.38-27759.46) | 19598.25 (12289.75-27687.26) | 19580.95 (12281.35-27679.95) | 19587.88 (12266.14-27772.44) | 19623.14 (12298.8-28022.61) | 19633.43 (12352.68-27893.52) | 19598.53 (12293.42-27812.74) | 19537.98 (12216.21-27918.82) | 19499.23 (12089.54-27721.4) | 19616.98 (12276.2-28117.67) | 19395.48 (12175.68-27808.65) |
| Guatemala | 25654.21 (17043.24-34574.15) | 25578.59 (16986.52-34510.22) | 25498.5 (16897.33-34543.01) | 25463.32 (16916.35-34811.69) | 25488.45 (16929.43-34968.02) | 25558.45 (16988.11-35064.1) | 25644.12 (16994.55-35144.97) | 25714.7 (16889.45-35201.84) | 25752.6 (16895.03-35050.07) | 25605.66 (16959.27-35007.48) | 25732.21 (16815.94-35028.15) |
| Republic of Moldova | 32205.32 (22695.87-42197.49) | 32218.94 (22853.94-42236.34) | 32220.94 (22886.66-42291.83) | 32218.92 (22799.46-42397.94) | 32227.7 (22803.74-42620.6) | 32230.89 (22880.6-42486.53) | 32214.1 (22884.99-42262.66) | 32191.72 (22848.36-42295.95) | 32185.48 (22861.26-42471.51) | 32389.11 (22682.71-42599.35) | 32463.14 (22843.37-42369.53) |
| United States Virgin Islands | 28077.22 (19775.47-36999.19) | 28087.07 (19847.39-36786.15) | 28101.26 (19832.53-36698.78) | 28116.95 (19831.23-36794.78) | 28135.97 (19776.92-36926.91) | 28166.73 (19762.05-36900.26) | 28215.23 (19712.84-36812.93) | 28282.63 (19726-37027.1) | 28365.6 (19744.07-37440.52) | 28672.1 (20062.38-37425.02) | 28392.05 (19675.33-37602.91) |
| Belize | 28923.73 (20287.14-37982.72) | 28909.99 (20272.47-37902.67) | 28901.67 (20324.07-37881.31) | 28899.29 (20282.36-37924.22) | 28901.66 (20183.67-38302.64) | 28882.63 (20243.03-37985.5) | 28832.56 (20346.49-37808.1) | 28777.15 (20211.63-37673.01) | 28744.87 (20110.9-37795.92) | 28880.7 (20185.27-38379.15) | 28751.45 (20097.21-37825.21) |
| Costa Rica | 25267.26 (16914.57-34604.36) | 25221.02 (16848.48-34504.11) | 25158.72 (16762.01-34395.22) | 25105.91 (16693.29-34362.03) | 25083.1 (16635.75-34356.16) | 25106.16 (16691.6-34531.22) | 25168.87 (16816.34-34622.87) | 25271.1 (16878.19-34857.68) | 25413.72 (16922.63-34924.66) | 25521.86 (17044.35-35046.09) | 25736.02 (17246.37-35446.97) |
| Suriname | 37456.78 (27696.05-48019.82) | 37547.87 (27769.41-48234.46) | 37655.89 (27957.04-48719.76) | 37741.29 (28045.47-49062.02) | 37762.62 (28050.63-49127.82) | 36734.18 (27152.48-46611.77) | 34469.05 (25191.13-43810.52) | 32119.61 (23051.3-41160.07) | 30833.09 (21710.32-40177.93) | 30677.81 (21716.72-39465.72) | 30645.68 (21562.73-39825.64) |
| Uruguay | 28227.9 (19578.34-37507.03) | 27878.1 (19404.01-37155.01) | 27536.39 (19077.16-36590.16) | 27390.09 (18923.58-36273.62) | 27471.31 (18917.09-36333.55) | 27720.9 (19186.75-36830.53) | 28037.37 (19412.93-37337.72) | 28322.98 (19630.97-37682.34) | 28488.39 (19713.65-37869.79) | 28515.93 (19770.19-37874.82) | 28170.53 (19571.42-37619.88) |
| Uzbekistan | 30895.94 (22197.11-40207.69) | 30895.21 (22191.25-40212.77) | 30882.01 (22260.97-40087.34) | 30866.25 (22314.65-40046.13) | 30861.24 (22350.59-40102.33) | 30865.98 (22245.98-39903.43) | 30870.91 (22233.01-39824.21) | 30876.23 (22215.45-39860.64) | 30894.75 (22313.49-40191.85) | 30944.31 (22317.71-40273.37) | 31027.5 (22354.35-40994.45) |
| San Marino | 19835.85 (12454.36-28007.7) | 19841.86 (12442.93-28068.37) | 19839.81 (12452.49-28128.31) | 19836.76 (12459.23-28305.98) | 19839.38 (12431.67-28555.8) | 19833.91 (12494.95-28344.91) | 19810.12 (12518.54-28114.37) | 19779.67 (12452.81-27955.77) | 19756.06 (12377.95-27772.2) | 19915.52 (12552.94-28267.82) | 19887.93 (12378.14-28393.6) |
| Tuvalu | 31543.29 (22616.22-40985.33) | 31519.51 (22536.59-40916.48) | 31491.83 (22584.4-40861.9) | 31459.19 (22630-40845.34) | 31420.8 (22573.19-40860.5) | 31357.48 (22546-40819.96) | 31269.99 (22457.29-40643.75) | 31185.8 (22339.16-40663.83) | 31134.61 (22145.54-40720.19) | 31207.26 (22202.56-40677.47) | 31375.83 (22402.34-40678.06) |
| Guyana | 28773.27 (20144.04-37654.53) | 28721.98 (20087.23-37577.29) | 28677.18 (20033.87-37437.25) | 28648.92 (20023.14-37374.19) | 28645.71 (20041.09-37415.47) | 28659.17 (20103.92-37417.96) | 28679.14 (20117.77-37407.38) | 28674.03 (20092.78-37555.42) | 28655.81 (19977.99-37717.56) | 28633.64 (20017.31-37578.81) | 28673.13 (20025.58-37613.36) |
| Jamaica | 28986.03 (20233.02-38371.36) | 29055.73 (20376.05-38288.66) | 29128.49 (20415-38376.66) | 29179.34 (20413.79-38348.91) | 29190.6 (20415.88-38350) | 29154.4 (20435.29-38134.8) | 29089.47 (20464.65-37810.12) | 29022.34 (20521.48-37693.81) | 28978.96 (20406.95-37802.09) | 28989.15 (20258.59-38092.6) | 28935.6 (19885.65-38247.98) |
| Tunisia | 26479.5 (17575.55-35961.33) | 26544.68 (17578.82-36100.24) | 26632.46 (17532.42-36342.5) | 26720.68 (17518.66-36651.46) | 26787.28 (17620.07-36720.27) | 26812.08 (17613.87-36672.61) | 26807.84 (17710.45-36565.4) | 26801.68 (17782.74-36512.7) | 26823.98 (17760.24-36555.88) | 27038.09 (18157.21-36834.3) | 27131.65 (18143.04-36987.82) |
| Albania | 0 (0-0) | 0 (0-0) | 0 (0-0) | 0 (0-0) | 0 (0-0) | 0 (0-0) | 5877.85 (1729.48-10581.91) | 5885.33 (1749.32-10593.57) | 5897.86 (1773.82-10660.35) | 5981.18 (1790.81-11042.46) | 5928.74 (1660.84-11020.94) |
| American Samoa | 0 (0-0) | 0 (0-0) | 0 (0-0) | 0 (0-0) | 0 (0-0) | 0 (0-0) | 6001.75 (1725.24-11149.45) | 5959.27 (1713.81-11078.43) | 5885.56 (1688.11-10881.6) | 1662.41 (429.94-3581.56) | 1671.89 (412.45-3660.45) |
| Angola | 0 (0-0) | 0 (0-0) | 0 (0-0) | 0 (0-0) | 0 (0-0) | 0 (0-0) | 5536.51 (1591.3-10315.16) | 5522 (1582.04-10286.8) | 5493.42 (1556.88-10173.29) | 3445.51 (1367.3-6104.34) | 1377.79 (264.91-3475.87) |
| Bangladesh | 0 (0-0) | 0 (0-0) | 0 (0-0) | 0 (0-0) | 0 (0-0) | 0 (0-0) | 6388.77 (1871.28-11728.18) | 6435.24 (1932.04-11797.83) | 6452.27 (1948.09-11857.21) | 6308.43 (1860.76-11400.34) | 6371.94 (1855.33-11473.31) |
| Barbados | 0 (0-0) | 0 (0-0) | 0 (0-0) | 0 (0-0) | 0 (0-0) | 0 (0-0) | 5863.72 (1633.13-11524.24) | 5852.63 (1643.2-11245.63) | 5841.93 (1660.6-11184.51) | 5805.85 (1692.52-10611.65) | 5872.62 (1681-10945.12) |
| Belgium | 0 (0-0) | 0 (0-0) | 0 (0-0) | 0 (0-0) | 0 (0-0) | 0 (0-0) | 5083.63 (1092.91-9780.89) | 5063.35 (1069.17-9858.37) | 5058.21 (1071.84-9875.66) | 5169.11 (1143.87-9986.61) | 5093.67 (1128.57-9781.59) |
| Benin | 0 (0-0) | 0 (0-0) | 0 (0-0) | 0 (0-0) | 0 (0-0) | 0 (0-0) | 5583.89 (1540.68-10554.97) | 5528.31 (1517.17-10466.13) | 5498.29 (1503.93-10381.96) | 5605.63 (1650.43-10329.24) | 5497.54 (1563.29-9979.95) |
| Bermuda | 0 (0-0) | 0 (0-0) | 0 (0-0) | 0 (0-0) | 0 (0-0) | 0 (0-0) | 5786.97 (1700.03-10837.43) | 5826.08 (1708.62-10968.45) | 5848.65 (1700.23-11136.24) | 5841.69 (1676.23-11477.26) | 5883.79 (1644.02-11163.94) |
| Bosnia and Herzegovina | 0 (0-0) | 0 (0-0) | 0 (0-0) | 0 (0-0) | 0 (0-0) | 0 (0-0) | 6132.01 (1804.9-11321.31) | 6133.02 (1768.38-11264.8) | 6130.96 (1703.08-11281.41) | 6159.89 (1654.44-11113.93) | 6151.12 (1819-11254.14) |
| Botswana | 0 (0-0) | 0 (0-0) | 0 (0-0) | 0 (0-0) | 0 (0-0) | 0 (0-0) | 5529.65 (1578.38-10044.22) | 5583.02 (1573.2-10197.21) | 5612.05 (1570.03-10389.12) | 5515.41 (1535.65-10373.86) | 5591.32 (1562.87-10483.7) |
| Bulgaria | 0 (0-0) | 0 (0-0) | 0 (0-0) | 0 (0-0) | 0 (0-0) | 0 (0-0) | 6087.76 (1724.56-11354.88) | 6119.17 (1764.31-11335.66) | 6129.55 (1766.63-11276.77) | 6016.78 (1730.68-11027.74) | 6038.29 (1611.62-11239.32) |
| Burkina Faso | 0 (0-0) | 0 (0-0) | 0 (0-0) | 0 (0-0) | 0 (0-0) | 0 (0-0) | 2898.45 (1563.31-5045.43) | 1895 (707.23-3971.79) | 1378.78 (247.98-3507.38) | 1312.29 (245.24-3253.66) | 1289.16 (233.48-3157.67) |
| Cabo Verde | 0 (0-0) | 0 (0-0) | 0 (0-0) | 0 (0-0) | 0 (0-0) | 0 (0-0) | 5479.39 (1568.39-10021.74) | 5477.46 (1567.36-9948.79) | 5478.74 (1536.93-10029.38) | 5510.6 (1583.03-10186.52) | 5552.48 (1558.05-10424.07) |
| Cameroon | 0 (0-0) | 0 (0-0) | 0 (0-0) | 0 (0-0) | 0 (0-0) | 0 (0-0) | 5570.03 (1534.48-10373.02) | 5536.65 (1519.14-10275.96) | 5517.89 (1513.47-10398.3) | 5599.55 (1526.34-10850.45) | 5480.2 (1511.61-9920.41) |
| Central African Republic | 0 (0-0) | 0 (0-0) | 0 (0-0) | 0 (0-0) | 0 (0-0) | 0 (0-0) | 1418.97 (280.18-3596.05) | 1424.43 (292.99-3577.79) | 1427.13 (293.4-3605.6) | 1434.44 (295.29-3586.65) | 1420.26 (292.04-3488.57) |
| Chad | 0 (0-0) | 0 (0-0) | 0 (0-0) | 0 (0-0) | 0 (0-0) | 0 (0-0) | 5628.67 (1570.51-10425.19) | 5626.72 (1551.08-10552.96) | 5624.38 (1538.68-10610.87) | 5608.95 (1551.74-10433.08) | 5608.35 (1573.67-10579.5) |
[truncated: 1,061,623 more chars]
