# Supplementary material for: Global and regional burden and inequalities of oral conditions in children, adolescents, and young adults (0–39 years), 1990 to 2021
Source: PLOS Glob Public Health. 2025 Oct 9;5(10):e0005274. doi: 10.1371/journal.pgph.0005274 (PMC12510561; doi:10.1371/journal.pgph.0005274)
Supplement: S4 Text — (DOCX) [file pgph.0005274.s004.docx]

# S4 Text: Supplementary Results

## Oral Disorder Incidence and Prevalence among Young Populations in 2021

Globally, untreated caries in deciduous teeth was the most incident oral disorder among young populations in 2021, with an estimated ASIR of 62261.07 (41905.15 to 89708.76) (per 100,000 populations). Untreated caries in permanent teeth was the most prevalent and contributed the largest share of YLDs in 2021, with an estimated ASPR of 28989.1 (20361.45-39942.86) and ASYLDR of 28.86 (11.38-57.95) (per 100,000 populations) (**Table 1**). Edentulism was the only oral disorder that exhibited a gender disparity in the burden, with the indicators being higher for females than for males (**Table 1, Fig 1 and Fig B-E in S3 Text**). The burden of untreated caries in deciduous teeth was lowest in High-SDI quintiles and highest in Middle- and High-Middle-SDI quintiles. The ASIR of untreated caries in permanent teeth was roughly equal across all locations, but the ASPR decreased with increasing SDI across regions. For periodontal diseases, the burden peaked in low-middle-SDI quintiles, and then decreased with increasing SDI. The ASIR of edentulism was highest in low- and high-SDI quintiles, while the ASPR was highest in low-SDI quintiles (**Table 1**).

## Drivers of Oral Disorder Epidemiology

The result of decomposition analysis indicated that population growth emerged as the primary factor driving this increase across all oral disorders, which accounted for 204.59%, 76.79%, 74.94%, and 80.81% of the increased incidence of untreated caries of deciduous teeth, untreated caries of permanent teeth, periodontal disease, edentulism, respectively. In low-, low-middle-, high-middle-, and high-SDI quintiles, population growth predominantly contributed to the incidence increase for untreated caries of deciduous teeth. In low-, low-middle-, and middle-SDI quintiles, population growth predominantly contributed to the incidence increase for untreated caries of permanent teeth, periodontal disease, and edentulism. Its impact in high-middle- and high-SDI quintiles varied, being not evident (the absolute value less than 10%) for periodontal disease and edentulism, negative for untreated caries of permanent teeth in high-middle-SDI quintile (-90.53%), positive for untreated caries of permanent teeth in high-SDI quintile (71.63%) (**Fig K in S3 Text**). Changes in population age structure led to an increase in the incidence of untreated caries of deciduous teeth (3.7%), untreated caries of permanent teeth (1.55%), periodontal disease (11.39%), and edentulism (9.55%). For untreated caries of deciduous teeth, the impact of age structure was not evident in low- and high-SDI quintiles and was -21.32% 23.9%, and 15.84% for low-middle-, middle-, and high-middle-SDI quintiles. For untreated caries of permanent teeth, the impact of age structure was evident in low-middle- and high-SDI quintiles, and was 8.12% and -99.28%, respectively. Regarding periodontal disease and edentulism, the age structure contributed significantly to incidence changes in middle (28.4% for periodontal disease and 16.62% for edentulism), high-middle- (105.68% for periodontal disease and 106.48% for edentulism), and high-SDI (60.29% for periodontal disease and 13.81% for edentulism) quintiles, less so in the low-middle SDI quintile (5.42% for periodontal disease and 4.52% for edentulism), and nearly negligibly in the low-SDI quintile. The epidemiological change resulted in a decreased incidence of untreated caries of deciduous teeth (-108.3%) and an increased incidence of untreated caries of permanent teeth (21.66%), periodontal disease (13.67%), and edentulism (9.94%). Its negative influence on incidence was observed in low- (-25.65%), low-middle- (-218.66%), middle- (-77.32%), and high-middle-SDI (-10.28%) quintiles and positive influence was observed in high-SDI quintile (19.57%) for untreated caries of deciduous teeth. The impact of epidemiological change on the incidence of untreated caries of permanent teeth was positive in middle- (46.7%) and high-middle-SDI (-214.55%) quintiles, negative in high-SDI quintile (-72.35%), and nearly negligible in the low- and low-middle-SDI quintiles. For periodontal disease, the epidemiological change had a negative impact on the low- (-62.54%) quintile and a positive impact on low-middle- (2.33%), middle- (16.84%), high-middle- (17.01%), and high-SDI (15.04%%) quintiles. For edentulism, the epidemiological change had a negative impact on incidence in low- (-6.5%), low-middle- (-32.56%), and high-middle-SDI (-46.43%) quintiles and a positive impact on middle- (18.76%) and high-SDI (74.91%) quintiles. Population growth emerged as the primary factor driving this increase across all oral disorders, which accounted for 204.59%, 76.79%, 74.94%, and 80.81% of the increased incidence of untreated caries of deciduous teeth, untreated caries of permanent teeth, periodontal disease, and edentulism, respectively. In low-, low-middle-, high-middle-, and high-SDI quintiles, population growth predominantly contributed to the incidence increase for untreated caries of deciduous teeth. In low-, low-middle-, and middle-SDI quintiles, population growth predominantly contributed to the incidence increase for untreated caries of permanent teeth, periodontal disease, and edentulism. Its impact in high-middle- and high-SDI quintiles varied, being not evident for periodontal disease and edentulism, negative for untreated caries of permanent teeth in the high-middle-SDI quintile (-90.53%), positive for untreated caries of permanent teeth in the high-SDI quintile (71.63%) (**Fig K in S3 Text**). Changes in population age structure led to an increase in the incidence of untreated caries of deciduous teeth (3.7%), untreated caries of permanent teeth (1.55%), periodontal disease (11.39%), and edentulism (9.55%). For untreated caries of deciduous teeth, the impact of age structure was not evident in low- and high-SDI quintiles and was -21.32% 23.9%, and 15.84% for low-middle-, middle-, and high-middle-SDI quintiles. For untreated caries of permanent teeth, the impact of age structure was evident in low-middle- and high-SDI quintiles, and was 8.12% and -99.28%, respectively. Regarding periodontal disease and edentulism, the age structure contributed significantly to incidence changes in middle (28.4% for periodontal disease and 16.62% for edentulism), high-middle- (105.68% for periodontal disease and 106.48% for edentulism), and high-SDI (60.29% for periodontal disease and 13.81% for edentulism) quintiles, less so in the low-middle SDI quintile (5.42% for periodontal disease and 4.52% for edentulism), and nearly negligibly in the low-SDI quintile. The epidemiological change resulted in a decreased incidence of untreated caries of deciduous teeth (-108.3%) and an increased incidence of untreated caries of permanent teeth (21.66%), periodontal disease (13.67%), and edentulism (9.94%). Its negative influence on incidence was observed in low- (-25.65%), low-middle- (-218.66%), middle- (-77.32%), and high-middle-SDI (-10.28%) quintiles and positive influence was observed in high-SDI quintile (19.57%) for untreated caries of deciduous teeth. The impact of epidemiological change on the incidence of untreated caries of permanent teeth was positive in the middle- (46.7%) and high-middle-SDI (-214.55%) quintiles, negative in high-SDI quintile (-72.35%), and nearly negligible in the low- and low-middle-SDI quintiles. For periodontal disease, the epidemiological change had a negative impact on the low- (-62.54%) quintile and a positive impact on low-middle- (2.33%), middle- (16.84%), high-middle- (17.01%), and high-SDI (15.04%%) quintiles. For edentulism, the epidemiological change had a negative impact on incidence in low- (-6.5%), low-middle- (-32.56%), and high-middle-SDI (-46.43%) quintiles and a positive impact on middle- (18.76%) and high-SDI (74.91%) quintiles.

Population growth emerged as the primary driver for all oral disorders, which accounted for 231.56%, 102.69%, 64.81%, 72.3%, and 85.47% of the increased prevalence of untreated caries of deciduous teeth, untreated caries of permanent teeth, periodontal disease, edentulism, and other oral disorders, respectively. Population growth predominantly contributed to the prevalence increase for untreated caries of deciduous teeth in all locations except middle-SDI (-37.82%) quintile (being 122.76%, 259.94%, -78.05%, and -58.27% in low-, and low-middle-, high-middle-, and high-SDI quintiles). In low-, low-middle-, and middle-SDI quintiles, population growth predominantly contributed to the prevalence increase for untreated caries of permanent teeth, periodontal disease, and edentulism. Its impact in high-middle- and high-SDI quintiles varied, being not evident (the absolute value less than 10%) for periodontal disease and edentulism, negative for untreated caries of permanent teeth in high-middle-SDI quintile (-59.53%), and nearly negligible for untreated caries of permanent teeth in high-SDI quintile (**Fig K in S3 Text**). For other oral disorders, population growth predominantly contributed to the prevalence increase in low- (94.71%), low-middle- (83.79%), middle- (65.82%), and high-middle-SDI (227.89%) quintiles, which is a secondary driver only in the high-SDI quintile (-155.29%). Changes in population age structure led to led to a decrease in the prevalence of untreated caries of deciduous teeth (-37.33%) but resulted in increases for untreated caries of permanent teeth (7.67%), periodontal disease (17.9%), edentulism (15.06%), and other oral disorders (13.76%). For untreated caries of deciduous teeth, the impact of age structure was not evident in low-, high-middle-, and high-SDI quintiles, and was -55.53% and -51.86% for low-middle- and middle-SDI quintiles. For untreated caries of permanent teeth, the impact of age structure was evident in low-middle-, middle-, and high-SDI quintiles, and was 13.48%, -16.12%, and 21.83%, respectively. Regarding periodontal disease and edentulism, the age structure contributed significantly to prevalence changes in low-middle- (10.83% for periodontal disease and 9.72% for edentulism), middle (35.92% for periodontal disease and 26.65% for edentulism), high-middle- (90.11% for periodontal disease and 129.87% for edentulism), and high-SDI (97.84% for periodontal disease and 15.12% for edentulism) quintiles, being nearly negligible in the low-SDI quintile. For other oral disorders, the age structure contributed 4.81%, 15.76%, 32.4%, 122.04%, and 252.3% to low-, low-middle-, middle-, high-middle-, and high-SDI quintiles. The epidemiological change resulted in decreased prevalence for untreated caries of deciduous teeth (-94.23%) and untreated caries of permanent teeth (-10.36%) and resulted in increased prevalence for periodontal disease (17.29%) and edentulism (12.64%). Its negative influence on prevalence was observed in all SDI quintiles for untreated caries of deciduous teeth (-16.72%, -104.41%, -10.32%, -16.98%, and -32.49 for low-, low-middle-, middle-, high-middle-, and high-SDI quintiles) and in low-, low-middle-, middle-, high-middle-SDI quintiles for untreated caries of permanent teeth (-5.4%, -12.69%, -13.23%, and -50.25%, respectively). The contribution of epidemiological change to the high-SDI quintile for untreated caries of permanent teeth is 125.86%. For periodontal disease, the epidemiological change had a negative impact on low- (-66.19%) and high-SDI (-25.3%) quintiles and a positive impact on low-middle- (4.38%), middle- (20.18%), and high-middle-SDI (24.38%) quintiles. For edentulism, the epidemiological change had a negative impact on prevalence in low- (-7.59%), low-middle- (-33.18%), and high-middle-SDI (-61.3%) quintiles and a positive impact on middle- (16.46%) and high-SDI (75.4%) quintiles. For other oral disorders, the impact of epidemiological change for all SDI quintiles was nearly negligible (the absolute value was less than 10%).

## SDI-related Inequality in the Age-standardized Incidence and Prevalence Rate of Oral Disorders

The findings from the slope index of inequality and concentration index of inequality indicated that there was no significant absolute or relative SDI-related inequality existed in the ASIR revealed that the burden of untreated caries in deciduous teeth was heavier in poorer regions in 1990 and heavier in wealthier regions in 2021, untreated caries in permanent teeth burden was heavier in wealthier regions in both 1990 and 2021, and the burden of edentulism was greater in poorer regions in both years (**Fig L in S3 Text**). However, the concentration index suggested no significant relative SDI-related inequality in the ASIR of untreated caries and edentulism (**Fig M in S3 Text**). For periodontal diseases, the distribution of SDI-related absolute and relative inequalities in ASIR mirrored that of ASYLDR. The sociodemographic attribution analysis partially supported the findings of ASIR, indicating that in 2021, 5.16% (about 62.92 million, 5.14% to 5.18%) incident cases of untreated caries in deciduous teeth, -2.20% (about -37.39 million, -2.21% to -2.18%) of untreated caries in permanent teeth, 29.33%, (about 10.47 million, 29.26% to 29.41%) of periodontal diseases, and -16.92% (about -0.36 million, -17.29% to -16.55%) of edentulism, could be attributable to cross-country sociodemographic inequality.

The findings from SII and CII indicated that there was no significant absolute or relative SDI-related inequality existed in the ASPR of untreated caries of deciduous teeth and untreated caries of permanent teeth (**Fig M-N in S3 Text**). And there was no significant difference between the results of 1990 and 2021. The burden of ASPR of untreated caries of deciduous teeth in wealthier countries became heavier than in poorer countries in 2021, while there was no such difference between them in 1990 [SII: 317.45 (-1456.01 to 2090.92) in 1990; 1542.23 (-450.44 to 3534.89) in 2021]. In the case of periodontal disease, the SII results showed that the burden of ASPR in poorer countries was heavier than in wealthier countries in both 1990 and 2021, and the absolute inequality gap between wealthier and poorer countries decreased in 2021 [SII: -9380.82 (-11663.89 to -7097.76) in 1990; -2556.70 (-4587.27 to -526.3) in 2021]. The results of CII supported those of SII, indicating a disproportionate concentration of burden of ASPR among poorer countries in both 1990 and 2021 [CII: -0.2 (-0.24 to -0.16) in 1990; -0.17 (-0.21 to -0.13) in 2021]. In the case of edentulism, the SII results showed that the burden of ASPR in poorer countries was heavier than in wealthier countries in both 1990 and 2021, and there was no significant difference between the results of 1990 and 2021 [SII: -632.21 (-874.02 to -390.40) in 1990; -584.26 (-802.10 to -366.43) in 2021]. The results of CII were not satisfied for the poor fitting results (**Fig M in S3 Text**), which indicated that there was no significant relative SDI-related inequality existed in the ASPR of edentulism [CII: -0.03 (-0.12 to -0.05) in 1990; -0.09 (-0.17 to -0.02) in 2021]. The findings from SII and CII indicated that there was no significant absolute or relative SDI-related inequality existed in the ASPR and ASYLDR of other oral disorders (**Fig L-M in S3 Text**). And there was no significant difference between the results of 1990 and 2021.

The sociodemographic attribution analysis partially supported the findings of ASIR, indicating that in 2021, 5.16% (about 62.92 million, 5.14% to 5.18%) incident cases of untreated caries in deciduous teeth, -2.20% (about -37.39 million, -2.21% to -2.18%) of untreated caries in permanent teeth, 29.33%, (about 10.47 million, 29.26% to 29.41%) of periodontal diseases, and -16.92% (about -0.36 million, -17.29% to -16.55%) of edentulism, could be attributable to cross-country sociodemographic inequality.

## Burden and Temporal Trends in age-standardized prevalence and YLD rates from 1990-2021 of Other Oral Disorder

Globally, other oral disorder was the least prevalent and contributed the least share of YLDs in all oral disorders among children, adolescents, and young adults in 2021, with an estimated ASPR of 1606.79 (1415.88 to 1801.16) per 100,000 populations and an estimated ASYLDR of 47.16 (28.52 to 71.02) per 100,000 populations. For other oral disorder, ASPR and ASYLDR increased with age after 4 years old and peaked at age 35-39 y. The burden of other oral disorder was roughly equal across all sexes and SDI quintiles.

The burden of other oral disorders in 2021 remained relatively unchanged in the period from 1990 to 2021 (**Table 1, Fig D-F in S3 Text)**. Regarding specific temporal trends, the ASPR and ASYLDR of other oral disorders exhibited a similar pattern of incline during 1990-1993 and 2007-2019 and decreased during 1993-2007. The ASPR increased from 2019 to 2021 while the ASYLDR decreased in this period **(Fig G in S3 Text)**.
