# Supplementary material for: Global and regional burden and inequalities of oral conditions in children, adolescents, and young adults (0–39 years), 1990 to 2021
Source: PLOS Glob Public Health. 2025 Oct 9;5(10):e0005274. doi: 10.1371/journal.pgph.0005274 (PMC12510561; doi:10.1371/journal.pgph.0005274)
Supplement: Attachment — Submitted filename: Response to reviewers.docx [file pgph.0005274.s006.docx]

**Rebuttal letter**

**Manuscript ID:** PGPH-D-25-02129

**Manuscript Title:** Global and Regional Burden and Inequalities of Oral Conditions in Children, Adolescents, and Young Adults (0-39 years), 1990 to 2021

Sep 15, 2025

Dr. Nicola Hawley

Academic Editor
*PLOS Global Public Health*

Dear editor,

We sincerely appreciate you and all reviewers for the valuable feedback that we have used to improve the quality of our manuscript. We responded to each comment in a point-by-point manner. The revisions in the main text of our manuscript are highlighted in red in the file labelled 'Revised Manuscript with Track Changes'.

Requirements and comments from the Editorial Office and reviewers are shown below in *italics*. Our response is given in normal font and blue text.

Thanks again for your contribution to this manuscript.

***Journal Requirements:***

*1. We have amended your Competing Interest statement to comply with journal style. We kindly ask that you double check the statement and let us know if anything is incorrect.*

**Response:**

Thank you for addressing the Competing Interest statement of this manuscript and for making the necessary amendments. We have double-checked the statement and confirm that no competing interests exist. Additionally, we have updated the statement in the manuscript (**Page 28, line 481**) to comply with the journal's requirements.

**Revisions:**

**Competing interests:** The authors deny any conflicts of interest related to this study.

*2. Some material included in your submission may be copyrighted. According to PLOS’s copyright policy, authors who use figures or other material (e.g., graphics, clipart, maps) from another author or copyright holder must demonstrate or obtain permission to publish this material under the Creative Commons Attribution 4.0 International (CC BY 4.0) License used by PLOS journals. Please closely review the details of PLOS’s copyright requirements here: PLOS Licenses and Copyright. If you need to request permissions from a copyright holder, you may use PLOS's Copyright Content Permission form.*

*Please respond directly to this email or email the journal office and provide any known details concerning your material's license terms and permissions required for reuse, even if you have not yet obtained copyright permissions or are unsure of your material's copyright compatibility.*

*Potential Copyright Issues: Figure 2: please (a) provide a direct link to the base layer of the map (i.e., the country or region border shape) and ensure this is also included in the figure legend; and (b) provide a link to the terms of use/license information for the base layer image or shapefile. We cannot publish proprietary or copyrighted maps (e.g. Google Maps, Mapquest) and the terms of use for your map base layer must be compatible with our CC-BY 4.0 license.*

*Note: if you created the map in a software program like R or ArcGIS, please locate and indicate the source of the basemap shapefile onto which data has been plotted.*

*If your map was obtained from a copyrighted source please amend the figure so that the base map used is from an openly available source. Alternatively, please provide explicit written permission from the copyright holder granting you the right to publish the material under our CC-BY 4.0 license.*

*Please note that the following CC BY licenses are compatible with PLOS license: CC BY 4.0, CC BY 2.0 and CC BY 3.0, meanwhile such licenses as CC BY-ND 3.0 and others are not compatible due to additional restrictions.*

**Response:**

Thank you for highlighting the important copyright concerns regarding the map used in this work. The base layer of our maps is sourced from the Resource and Environment Science and Data Platform, Institute of Geographic Sciences and Natural Resources Research, Chinese Academy of Sciences (Direct link: https://www.resdc.cn/data.aspx?DATAID=205). The map of global national administrative boundaries is an open-access resource available on this platform (<https://www.resdc.cn/Introduction.aspx?WebShieldSessionVerify=O6FthJA883s7OiMfx7hY>). We have included the links in the figure legends (**Page 13, lines 232-233**) and in the “Availability of data and materials” section (**Page 28, lines 476-479**). We appreciate your guidance and believe that these revisions will enhance the quality of our submission.

**Revisions:**

**Figure Legends:** …The link to the base layer of the map: <https://www.resdc.cn/data.aspx?DATAID=205>

**Availability of data and materials:** ……The map of global national administrative boundaries used in this work is from the Resource and Environment Science and Data Platform, Institute of Geographic Sciences and Natural Resources Research, Chinese Academy of Sciences, and is publicly available at https://www.resdc.cn/data.aspx?DATAID=205

*3. If the reviewer comments include a recommendation to cite specific previously published works, please review and evaluate these publications to determine whether they are relevant and should be cited. There is no requirement to cite these works unless the editor has indicated otherwise.*

**Response:**

Thanks for the reminder. Reviewers did not recommend citing any works. The references cited in this work were selected carefully.

*4.Please review your reference list to ensure that it is complete and correct. If you have cited papers that have been retracted, please include the rationale for doing so in the manuscript text, or remove these references and replace them with relevant current references. Any changes to the reference list should be mentioned in the rebuttal letter that accompanies your revised manuscript. If you need to cite a retracted article, indicate the article’s retracted status in the References list and also include a citation and full reference for the retraction notice.*

**Response:**

Thanks for your kind request regarding the reference list of this manuscript. In response to it, we have thoroughly reviewed all citations in our manuscript. We have carefully checked each reference for correctness and completeness. We would like to confirm that we did not cite any retracted references in our manuscript. Therefore, there was no need to include a rationale for any retracted articles. We have ensured that all references are current and relevant to our study. We appreciate your guidance on this matter.

***Reviewer(s)' Comments to Author:***

***Reviewer #1：***

*This manuscript is scientifically rigorous, fills a critical evidence gap in youth oral health burden research, and provides actionable insights for equitable public health interventions. No revisions are needed—I strongly recommend direct acceptance for publication in PLOS Global Public Health.*

**Response:**

We would like to express our sincere gratitude to Reviewer #1 for the positive feedback on the manuscript. We are delighted to hear that the reviewer found our work to be scientifically rigorous and valuable in addressing the evidence gap in the oral health burden of youth. The recognition of the actionable insights provided for equitable public health interventions is particularly encouraging.

Thanks again for the reviewer’s thoughtful consideration of our work and recommendation for direct acceptance.

***Reviewer #2：***

*Oral diseases are frequently overlooked when considering the global burden of noncommunicable disease, despite being a major public health concern. The authors of this manuscript, “Global and Regional Burden and Inequalities of Oral Conditions in Children, Adolescents, and Young Adults (0-39 years), 1990 to 2021,” utilize Global Burden of Diseases, Injuries, and Risk Factors Study (GBD) data to identify temporal trends (via joinpoint regression) and drivers (via decomposition analysis) of non-fatal oral diseases over three decades for young populations. Years lived with disability (YLDs), incidence, and prevalence for deciduous caries, permanent caries, periodontal disease, edentulism, and other oral disorders and sociodemographic index (SDI) quintile for countries and territories were obtained from GBD. Age-standardized rates were calculated for all phenotypes, and relative (concentration index) and absolute (slope index) inequalities in oral disease burden as well as the burden attributable to inequality in sociodemographic development were assessed. Periodontal disease was responsible for the most YLDs of the diseases present in the study, and population growth was identified as the major driver of YLDs globally. SDI-related inequalities were observed for periodontal disease only. A major strength of this study is the inclusion of stratified analyses for age (children, 0-9 years; adolescents, 10-19 years; young adults, 20-39 years) and SDI quintile (low, low-middle, middle, high-middle, high), which highlighted differences in the burden of disease and their temporal trends across these demographics. This study is helpful in explaining the global landscape of oral diseases over the last 30 years and illustrates the impacts of COVID-19 on oral health at a population level, particularly for children aged 0-9 years. These findings will inform public health strategies for oral disease prevention.*

**Response:**

We would like to express our appreciation to Reviewer #2 for the thorough and insightful evaluation of our manuscript. We are grateful for the reviewer’s recognition of the significance of oral diseases within the context of noncommunicable diseases and the thoughtful summary of our findings. The acknowledgment of the study's strengths, particularly the stratified analyses and the implications for public health strategies, is encouraging. We appreciate the time and effort the reviewer invested in providing such detailed feedback, which reinforces the importance of our research in informing oral disease prevention strategies. Thanks once again for the constructive feedback and support.

***Minor comments:***

*1. A maximum of 5 joinpoints was allowed for in the joinpoint regression, but no justification is provided for this parameter.*

**Response:**

Thank you for highlighting this methodological concern. According to the National Cancer Institute, the algorithmic recommendations indicate a default maximum number of 5 joinpoints when the number of data points ranges from 27 to 31 (<https://surveillance.cancer.gov/help/joinpoint/setting-parameters/method-and-parameters-tab/number-of-joinpoints>). In our study, the focused year range is from 1990 to 2021, resulting in 32 data points (32 years), which is approximately equal to 31. Therefore, we set the maximum number of joinpoints to 5. We have added the justification for this parameter to the “Materials and methods” section (**Page 10, lines 169-172**). We appreciate your critique and hope this clarification addresses your concerns.

**Revisions:**

A maximum of five joinpoints was allowed for the algorithmic recommendations from the National Cancer Institute (<https://surveillance.cancer.gov/help/joinpoint/setting-parameters/method-and-parameters-tab/number-of-joinpoints>), with model selection conducted using the Monte Carlo Permutation method.

*2. Table 1 is impossibly small and unreadable.*

**Response:**

Thank you for your insightful critique regarding the readability of Table 1. We acknowledge that the table contains a substantial amount of data, which limits the font size in an A4 layout. In the revised manuscript, we have increased the font size and adjusted the page orientation to landscape to provide more width for improved readability for reviewers. For the final presentation to readers, we have referenced similar studies published in *PLOS Glob Public Health* with large data tables (1, 2), and believe that optimal readability can be achieved through the journal's presentation of tables with separate links and the production team's specialized arrangement. We appreciate your constructive feedback and are confident that these revisions will enhance the quality of our submission.

**References:**

1.	Karp C, Tumlinson K, Bullington BW, Zimmerman LA, Senderowicz L. Keeping the "R" in LARC (long-acting reversible contraception): Measuring client-centered implant removal services in sub-Saharan Africa. PLOS Glob Public Health. 2025;5(7):e0004478.

2.	Vecino-Ortiz AI, Roberton T, López-Hernández A, Noonan CM, Vega Landaeta AP, Maceira D, et al. Bending the curve: Modeling the impact of reducing risk factors for noncommunicable diseases to control future health expenditures in Latin America and the Caribbean. PLOS Glob Public Health. 2025;5(7):e0004791.

*3. Figure 2 is fantastic, but the legends correspond to different age-standardized YLD rates (ASYLDRs), making comparisons across different age groups and among the various oral diseases challenging. Consider using the same scale for all plots in this figure.*

**Response:**

Thank you for your valuable feedback regarding the presentation of Figure 2. In response, we have unified the scale across all plots in a new version of Figure 2:

This uniform scale facilitates comparisons across different age groups and oral diseases. However, the variation in burden magnitudes among subgroups and diseases may obscure differences between countries in certain plots. For instance, when using the scale for periodontal diseases in young adults as the standard, the burden of deciduous caries in adolescents appears incomparable across all countries in one separate map due to the significant disparity in absolute ASYLDR values, resulting in a map dominated by dark blue. **Therefore, we have retained the original Figure in the main text and included the new version in the Supporting Information as Figure S4 (Supporting Information, Page 19) for interested readers**.

Additionally, during the revision process, we uploaded all figure files to the Preflight Analysis and Conversion Engine (PACE) digital diagnostic tool (https://pacev2.apexcovantage.com/) to ensure compliance with PLOS requirements.

Overall, we express our gratitude to the editors and reviewers for your careful evaluation and valuable comments. We hope our revisions have improved the clarity of our manuscript. We appreciate your time and look forward to your response.

Sincerely yours,

Liu Liu, DDS, PhD
State Key Laboratory of Oral Diseases & Department of Orthodontics, West China Hospital of Stomatology, Sichuan University
14# Third Section, Renmin Nan Road, 610041 Chengdu, China.

Hu Long, DDS, PhD
State Key Laboratory of Oral Diseases & Department of Orthodontics, West China Hospital of Stomatology, Sichuan University
14# Third Section, Renmin Nan Road, 610041 Chengdu, China.
